# Developing generic templates to shape the future for conducting integrated research platform trials

**DOI:** 10.1186/s13063-024-08034-8

**Published:** 2024-03-21

**Authors:** Madhavi Gidh-Jain, Tom Parke, Franz König, Cecile Spiertz, Peter Mesenbrink, On behalf of EU-PEARL (Europe [EU] Patient-cEntric clinicAl tRial pLatforms) an Innovative Medicines Initiative (IMI) initiative Work Package 2 (WP 2), Fabienne Baffert, Robert Patrizi

**Affiliations:** 1 55 Corporate Drive, Bridgewater, NJ 08807 USA; 2 Berry Consultants, Suite3, 5 East Saint Helen Street, Abingdon, OX14 5EG UK; 3 Center for Medical Statistics, Informatics, and Intelligent Systems, Medical University of Vienna, Vienna, Austria; 4 Exscientia, Oxford, UK; 5 Novartis Pharmaceuticals Corporation, One Health Plaza, East Hanover, NJ 07936 USA

**Keywords:** Master Protocol Template, Integrated research platform trials, EU Patient-cEntric clinicAl tRial pLatforms

## Abstract

**Background:**

Interventional clinical studies conducted in the regulated drug research environment are designed using International Council for Harmonisation (ICH) regulatory guidance documents: ICH E6 (R2) Good Clinical Practice—scientific guideline, first published in 2002 and last updated in 2016. This document provides an international ethical and scientific quality standard for designing and conducting trials that involve the participation of human subjects. Recently, there has been heightened awareness of the importance of integrated research platform trials (IRPs) designed to evaluate multiple therapies simultaneously. The use of a single master protocol as a key source document to fulfill trial conduct obligations has resulted in a re-examination of the templates used to fulfill the dynamic regulatory and modern drug development environment challenges.

**Methods:**

Regulatory medical writing, biostatistical, and other members of EU Patient-cEntric clinicAl tRial pLatforms (EU-PEARL) developed the suite of templates for IRPs over a 3.5-year period. Stakeholders contributing expertise included academic hospitals, pharmaceutical companies, non-governmental organizations, patient representative groups, and small and medium-sized enterprises (SMEs).

**Results:**

The suite of templates for IRPs based on TransCelerate’s Common Protocol Template (CPT) and statistical analysis plan (SAP) should help authors navigate relevant guidelines as they create study design content relevant for today’s IRP studies. It offers practical suggestions for adaptive platform designs which offer flexible features such as dropping treatments for futility or adding new treatments to be tested during a trial. The EU-PEARL suite of templates for IRPs comprises a preface, followed by the actual resource. The preface clarifies the intended use and underlying principles that inform resource utility. The preface lists references contributing to the development of the resource. The resource includes TransCelerate CPT guidance text, and EU-PEARL-derived guidance text, distinguished from one another using shading. Rationale comments are used throughout for clarification purposes. In addition, a user-friendly, functional, and informative Platform Trials Best Practices tool to support the setup, design, planning, implementation, and conduct of complex and innovative trials to support multi-sourced/multi-company platform trials is also provided. Together, the EU-PEARL suite of templates and the Platform Trials Best Practices tool constitute the reference user manual.

**Conclusions:**

This publication is intended to enhance the use, understanding, and dissemination of the EU-PEARL suite of templates for designing IRPs. The reference user manual and the associated website (http://www.eu-pearl) should facilitate the designing of IRP trials.

**Supplementary Information:**

The online version contains supplementary material available at 10.1186/s13063-024-08034-8.

## Background

EU-PEARL (EU Patient-cEntric clinicAl tRial pLatforms) was a strategic partnership between the public and private sectors to shape the future of clinical drug development. This innovative consortium aimed to create a framework for patient-centric integrated research platforms (IRPs), through which novel techniques and treatments developed by multiple companies and organizations could be evaluated in platform trials. To achieve this goal, EU-PEARL promoted collaboration between pharmaceutical companies, researchers, clinicians, and patients and encouraged knowledge sharing as well as open discussion among all stakeholders. This sustainable and reusable systematic approach to IRP trials conceived to test multi-sourced treatment is supported by a structure designed by EU-PEARL which will be able to meet complex regulatory, ethical, legal, statistical, and data requirements [1].

EU-PEARL was a key part of the overall approach of the European Commission (EC) of improving the landscape of clinical trials in Europe (EU). The new European Commission (EC), the Heads of Medicines Agencies (HMA), and the European Medicines Agency (EMA) have launched an initiative to transform how clinical trials are initiated, designed, and run, referred to as Accelerating Clinical Trials in the EU (ACT EU) [2]. Building on the application of the Clinical Trials Regulation (CTR) (Clinical Trials Regulation—Regulation EU No 536/2014) [3] and the launch of the Clinical Trials Information System (CTIS) on 31 January 2022, ACT EU will strengthen the European environment for clinical trials, while maintaining the high level of protection of trial participants, data robustness, and transparency that EU citizens expect.

The EU-PEARL project has received funding from the Innovative Medicines (http://www.imi.europa.eu) Initiative 2 Joint Undertaking (JU) under grant agreement no. 853966. The JU receives support from the European Union’s Horizon 2020 research and innovation program and European Federation of Pharmaceutical Industries and Associations (EFPIA), Children’s Tumor Foundation, Global Alliance For Tuberculosis (TB) Drug Development Non-Profit Organisation, Springworks Therapeutics Inc.

The objective of this project was to create an integrated set of methods, tools, and standard procedures to support all stakeholders to navigate relevant guidelines as they conduct today’s IRP trials. The main resources are based on input from clinical research professionals who report clinical studies using the ICH guidelines, and extensive regulatory review, followed by a structured approach to develop internationally based consensus.

The set of generic platform trial templates consists of the Master Protocol Template (MPT) which governs the entire study and includes the common key study design elements; the sub-protocols or alternately termed Intervention-Specific Appendices (ISAs), dedicated to focus on topics that are specific to the intervention cohort. The suite of templates includes a statistical analysis plan (SAP) template which covers all the statistical analyses that are planned for all interventions as well as those statistical analyses that are planned to be conducted at the sub-study or ISA level and a guidance document for the EU CTR cover letter (Additional files 1, 2, 3, and 4). A separate template for the SAP of the ISA was not created because the SAP template was created with enough flexibility such that the analyses described could be performed for all interventions or the content could be adapted and applied at the intervention level. The suite of templates is completed with a cross-functional Platform Trials Best Practices tool (Additional file 5) to assist in the setup of platform trials to ensure key operational tasks are planned for and completed. A Data Monitoring Committee (DMC) Charter template was also created that serves as a central basis for the ongoing of all cohorts that are active at any point in time in the study. The suite of templates was developed from the templates for protocol and SAP from TransCelerate [4] as these TransCelerate templates had a structure that is currently aligned with ICH standards (as well as the Food and Drug Administration [FDA]/National Institutes of Health [NIH] template), and these templates are widely implemented within the pharmaceutical industry. Utilization of this known and accepted format allowed for the development of more advanced and detailed template versions within the allocated development timeline.

## Methods

### Composition of EU-PEARL

EU-PEARL is a multi-stakeholder collaborative research project that was started in 2019 and ended in 2023, consisting of 36 partners from academic hospitals, pharmaceutical companies, non-governmental organizations, patient representative groups, and small and medium-sized enterprises (SMEs). As a strategic public–private alliance, its aim was to transform the current approach of conducting single-compound clinical trials into cross-company collaborative, multi-compound, adaptive platform trials, centered around patients, to be able to test multiple drug development compounds at the same time and potentially accelerate drug development. This would be particularly beneficial for those people living with high unmet clinical and health needs.

In November 2019, EU-PEARL convened the Work Package 2 (WP2), a group of clinical research experts, to address the current limitations of conducting clinical platform studies and offer potential statistical, regulatory, and operational methodology solutions. EU-PEARL was designing frameworks, tools, and guidance material, to promote this new approach to accelerate the efficient set-up of IRPs. The authors of this publication are WP2 members. The WP2 comprises of experts in biostatistics, medical writing, regulatory affairs, safety, information technology, and clinical operations. These individuals are employees of a pharmaceutical company, contract research organizations, technology vendors, and academia, brought together to represent the range of perspectives of professionals commonly engaged in authoring clinical regulatory documents.

All authors gave their time and expertise to this project as representatives of their organizations in EU-PEARL, in the belief that an open-access user templates and tools to support conducting platform clinical studies would benefit today’s healthcare industry. Further information on individual author contributions is included in the “Authors’ information” section.

### Project plan

During the 3.5-year project, EU-PEARL took a pragmatic and responsive approach to challenges presented by a dynamic environment for platform trials. The original project plan [5] described the intention to establish clear communication and standardized terminology, as well as continuous awareness on the design of platform trial components, benefits, and challenges. The authoring and development were done internationally based on the involvement of relevant stakeholders. The planning and conduct of this project referred to available EU [6, 7] and FDA guidance [8, 9]. Lessons from COVID-19 platform trials were also incorporated [10].

**Fig. 1:**
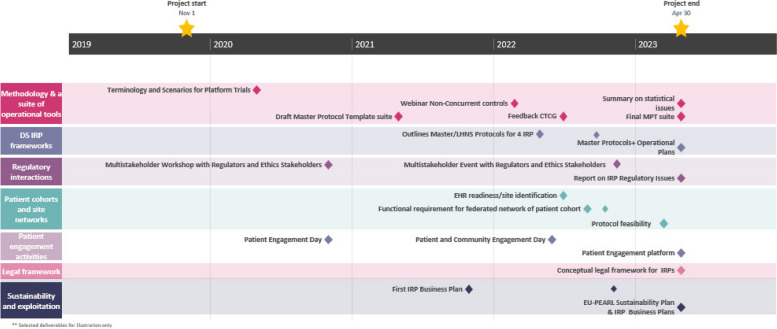
Process map of the EU-PEARL project

The 3.5-year roadmap (Fig. 1) summarizes the EU-PEARL’s planned and conducted oversight reviews. The stakeholders included representatives from global industry associations, regulatory agency, patient advocacy groups, and academic and contract research organizations, who reviewed the templates and tools, and provided insights and analysis over the period of November 2019 to April 2023.

Multiple, extensive, and rigorous regulatory interactions were conducted throughout the 3.5-year project to support the broad aim of integrating relevant global and regional (EU and United States of America [USA]) regulatory guidance received on use cases into the resource. Due diligence was exercised throughout and to the best ability of the EU-PEARL.

### Development of the generic templates

After deciding that EU-PEARL wished to incorporate certain elements of the TransCelerate templates, in August 2020, EU-PEARL contacted the TransCelerate Clinical Content & Reuse (CC&R) Project Management for approval to base their Master Protocol Templates on TransCelerate’s Common Protocol template Version 8.0, copyright TransCelerate Biopharma Inc. 2015–2020 and the statistical analysis plan (SAP) on the Statistical Analysis Plan template Version 3.0, copyright TransCelerate Biopharma Inc. 2018–2020. TransCelerate’s CC&R assets are publicly available, and all users are invited to build upon them with appropriate acknowledgment. It is recognized by EU-PEARL that the TransCelerate CPT and SAP templates are updated regularly to accommodate the latest international clinical trial standards and standard practices. EU-PEARL decided to use the CPT version 8.0 and SAP version 3.0 as they were the most recently issued versions at the time of the launch of the work plan.

Continued periodic connections with TransCelerate over the past 2 years were made while EU-PEARL was developing solutions. TransCelerate was not involved formally in asset development. However, two CC&R TransCelerate team members (medical writing and statistics) participated in the development of the generic templates and were members of the EU-PEARL WP2 team. TransCelerate CC&R shared experiences with EU-PEARL regarding asset development and deployment of key themes, release and storage of assets, creation of guidance vs. separate templates, communication/rollout experience, sustainability considerations, point-in-time asset versus ongoing maintenance, and feedback options.

In December 2020, the first version of the templates was launched and broadly shared within the EU-PEARL consortium and presented to operational experts of the disease-specific work packages (DSWPs). The DSWPs for major depressive disorder (MDD), tuberculosis (TB), non-alcoholic steatohepatitis (NASH), and neurofibromatosis (NF) used the EU-PEARL templates as a source of reference when writing their disease-specific master protocol.

The feedback from the DSWPs was integrated into the templates leading to version 2 with the following additional sections: informed consent, data collection, data sharing and disclosure, and considerations on patient engagement. In June 2021, version 2 was submitted to IMI. Between the IMI submission and the Clinical Trials Facilitation Group (CTFG) review, the DSWPs reviewed the templates by using them for their own disease-specific protocols. The feedback from them was integrated. In February 2022, version 3 was sent to CTFG for review. Further enhancements were made based on CTFG feedback.

The final version 4 was released in April 2023 and was based on the latest review by all partners in WP2, independent reviewers from other work packages and the EU-PEARL Steering Committee and Project Management Office. The period from March 2023 to the end of May 2023 was spent finalizing (including quality control and proofreading steps) the templates, website, and this publication.

To allow rapid dissemination of periodic project updates, the updates on the planned templates were presented at the following:

- EU-PEARL 1st Stakeholder Event, October 2020 [virtual]
- EU-PEARL 2nd Stakeholder Event, November 2022, [in-person/virtual] Amsterdam, the Netherlands
- EU-PEARL Patient and Community Engagement Day, June 2022 [virtual]
- DIA Clinical Trials and Data Science Conference, October 2022, Amsterdam, the Netherlands
- MCP Conference - 12th International Conference on Multiple Comparison Procedures September 2022 [in-person/virtual], Bremen, Germany
- PSI (Statisticians in the Pharmaceutical Industry) Annual Conference, June 2022, Gothenburg, Sweden
- DIA Global Annual Meeting, June 2022, Chicago, USA

The idea for a website to house the resource and support its utility, and logos to brand the EU-PEARL resource was planned. Many of the EU-PEARL web features for downloadable templates were influenced by the TransCelerate website.

The broad aims set out at the start of this project have been fulfilled through the final published resource, and the planned 3.5-year project timeline has been met. EU-PEARL Templates are available at http://www.eu-pearl.eu. This publication describes the project and the launch of EU-PEARL generic templates and Platform Trials Best Practices tool and is intended to enhance the use, understanding, and dissemination of these materials.

### Contributors to the development of suite of templates—Work Package 2

#### Key stakeholder

Clinical Trials Facilitation Group (CTFG): Monique AL submitted the consolidated opinion from CTFG on behalf of the Chair and Vice-Chair of the CTFG, Dr. Marianne Lunzer and Dr. Greet Munch.

## Results

### Broad principles

EU-PEARL developed five templates for sponsors developing their own platform trials: a Master Protocol Template (MPT), an Intervention Specific Appendix (ISA) or sub-protocol template, a template for the statistical analysis plan (SAP), a data monitoring committee (DMC) charter template adjusted for the purpose of conducting a platform trial, and a guidance for supplementary information to the CTR Cover Letter. A spreadsheet which is intended to be a Platform Trials Best Practices tool that can be used to assist in the operational planning of a collaborative platform trial is also provided.

EU-PEARL templates are provided as a PDF in a user manual (Additional files 1, 2, 3, and 4). The separate Platform Trials Best Practices tool is provided in Excel format to support its utility in a user manual (Additional file 5).

EU-PEARL templates present content suggestions and best practices that add value for creating ICH-compliant protocols and SAPs, but these come with the caveat that they may not work in all situations. IRP authors should use their judgment and, above all, make sensible structuring choices based on their specific IRP.

In addition, the goal was for EU-PEARL to be globally relevant, and the publication includes links to relevant regional guidances and other useful resources where possible, with explanation, to maximize utility of the resource. Consultation with the relevant regulatory health authority is highly recommended in cases where there is doubt.

The EC, EMA, and CTFG have jointly issued in 2022 “Complex Clinical Trials—Questions and Answers” document for which “Recommendation Paper on the Initiation and Conduct of Complex Clinical Trials” issued in 2019 by the former Clinical Trials Coordination Group (CTCG) has served as a basis. This document provides clarification or additional information and lays out certain considerations regarding scientific aspects, planning and set-up, submission for obtaining CT authorization (CTA), conduct, reporting and transparency, analysis, and interpretation of Complex Clinical Trials (CCTs) under the EU Clinical Trials Regulation 536/2014 (CTR) and EU In Vitro Diagnostic Medical Devices Regulation 2017/746 (IVDR) [11], as well as their use in submissions for marketing authorization.

In March 2022, The Food and Drug Administration (FDA or agency) announced the availability of a final guidance for the industry entitled “Master Protocols: Efficient Clinical Trial Design Strategies to Expedite Development of Oncology Drugs and Biologics.” Master protocols use a single infrastructure, trial design, and protocol to simultaneously evaluate multiple drugs and/or disease populations in multiple sub-studies, allowing for efficient and accelerated drug development.

The EU-PEARL templates are written in Microsoft Word and start with reader instruction. Different font colors are used to explain the intended use: instructional text, common and suggested text, variable text, example text, and highlighted yellow text. The yellow highlighted text in the EU-PEARL templates shows modifications that were made to the TransCelerate CPT V8 to make the template suitable for the master protocol approach. Deletions from the CPT are not shown. Instructional and example text are integrated into the templates through discrete color-coded fonts. These should help authors make informed choices as they navigate the evolving and complex area of IRPs.

Platform Trials Best Practices tool is an Excel spreadsheet divided over several tabs, each tab applicable to a particular clinical operations sub-team involved in the planning of a platform trial. This tool may not be all-inclusive due to the variety and complexity of platform trials, different organizational procedures, and national/local requirements. Therefore, reference to the appropriate governance committee’s guidance on relevant standard operating procedures (SOPs), company/organizational work instructions, etc. for applicable related procedures/requirements is recommended.

### Generic templates

The use of templates to develop regulatory documents is a standard practice and is well accepted by health authorities, industry, and academia. For clinical studies, the use of a protocol template provides a common structure that might benefit sponsors, investigators, sites, vendors, and regulators. It supports consistency, clarity, and ease of use and offers further efficiencies. Platform trials often have a structure of a master protocol and associated ISAs or sub-protocols.

**Fig. 2:**
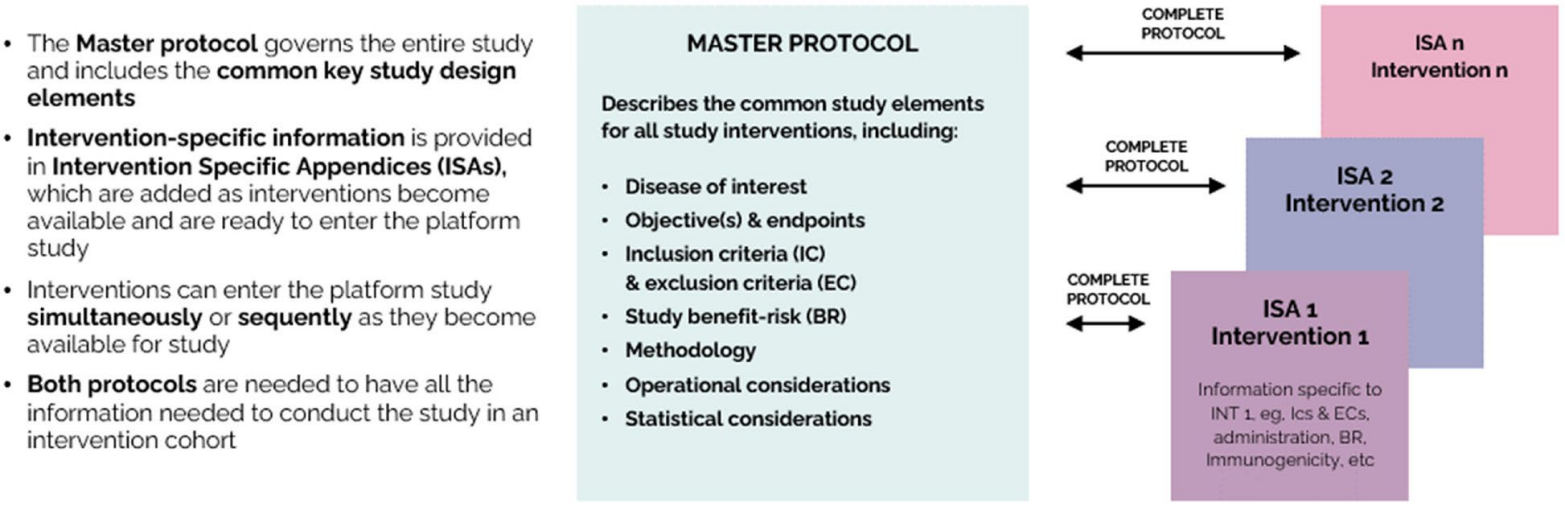
Outline of the master protocol and ISA or sub-protocol

EU-PEARL developed five templates for sponsors developing their own platform trials: a Master Protocol Template (MPT) and an Intervention Specific Appendix (ISA) or sub-protocol template (Fig. 2), a template for the statistical analysis plan (SAP), a data monitoring committee (DMC) Charter template adjusted for the purpose of conducting a platform trial, and a guidance for supplementary information to the CTR Cover Letter.

The core of the study setup is determined by the master protocol. The ISA is the appendix to the related master protocol which describes: the specific features of the intervention and treatment of participants assigned to that specific intervention or the control group to which it is compared, as well as intervention-specific assessments and procedures (e.g., administration of study medication when there are different dosing frequencies and routes of administration, and any special laboratory evaluations/biomarkers, endpoints, and risks that are intervention-specific). The ISA is a simplification of the concept of a domain-specific appendix which could be used to support platform trials like REMAP-CAP [12].

The templates allow the users to focus on the science and strategic development of the integrated research platforms without having to be concerned about the flow of content within the protocol that they are developing. In addition, the structure of the master protocol and ISA template use consistent section numbering to allow for easy cross-referencing across the two templates. When new intervention cohorts are started in a platform trial, both the master protocol and new ISA need to be submitted to the Clinical Trials Information System (CTIS) since each ISA will have a unique EUDRA-CT number.

Additional elements in the master protocol include the following:

- Interventional Medicinal Product (IMP) or Intervention Selection Committee, including independent experts (which may have different types of expertise, such as scientific, ethics, supply chain expertise, depending on specific needs), to advise on the inclusion of new IMPs into the trial. This committee will continue to evaluate potential arms for the trial and prioritize their importance based on newly emerging preclinical and clinical data. The IMP or Intervention Selection Committee Charter will provide details on the decision rules and what decision criteria should be considered.
- Two-step informed consent: The informed consent process may vary across different types of platform studies. The informed consent must be signed before the first study-related activity, which would typically be for the master protocol (e.g., master protocol informed consent form [ICF]). After consent is signed for the master protocol, the informed consent process for the intervention cohort(s) may depend on the number of intervention cohort(s) in a platform study. If only a single intervention cohort is open in a platform study, the master protocol and ISA ICFs may be signed at the same time. If multiple intervention cohorts are open in a platform study and participants are to be allocated or randomized among intervention cohorts after screening for the master protocol, the ICFs may be signed sequentially, e.g., the master protocol ICF would be signed to permit screening procedures for allocation/randomization to an intervention cohort, followed by the applicable intervention-specific ICF. If there are specific screening or eligibility criteria that determine the intervention cohort that participants will be allocated or randomized to, these screening/eligibility criteria should be in the master protocol and the master ICF. Intervention-specific screening/eligibility criteria should only be in the ISA protocol and the ISA ICF.
- Participant input into design: overall scientific integrity and regulatory compliance of the study. This section should describe the methodology used to collect patient inputs. If applicable, include a justification of why there is no engagement of patients required. For additional guidance and definition refer to https://eupati.eu/resources/patient-engagement-roadmap or https://patientfocusedmedicine.org/pem-suite.
- Compliance with EU CTR in accordance with Clinical Trials Regulation (EU No. 536/2014), procedures for reporting SUSARs, urgent safety measures, serious breaches, and change risk/benefit due to unexpected events were added.

The guidance and considerations in the cover letter are intended to help the reviewers and readers navigate and understand the master protocol across the entire platform trial and across all ISAs. The cover letter also contains ideas and suggestions for different tables and charts. For example, it contains a platform trial tracker, which is a table that provides an overview to keep track of the different versions of the key documents, the amendments, and the current status (e.g., approved or filed), so that they can be easily understood and navigated by its users. Using a graphical visualization in the cover letter depicting all closed, current, and planned sub-protocols/arms (e.g., status overview) as proposed in the EU-PEARL guidance for the cover letter is also encouraged in the Q&A on Complex Clinical Trials by ACT EU.

More details regarding the different templates, as well as their creation and their internal and external review process, are covered in the publicly available report on “Deliverable D2.6—Final Generic Master Protocol Template and Appendix for IRPs” on the EU-PEARL website.

The Platform Trials Best Practices tool spreadsheet is divided into several tabs, each tab applicable to a particular clinical operations sub-team involved in the planning of a platform trial. The tabs are as follows: index, instructions, definitions are covered in the first three tabs, clinical operations, electronic health records (eHR), data management, medical writing, clinical supplies, governance, safety, statistics, regulatory topics to be considered for preparing the submission dossiers, Data Monitoring Committee (DMC) and Data and Safety Monitoring Board (DSMB), patient engagement, data protection, data sharing, and communications. Each tab is organized as a table. There is a row for each task that has been identified. The column headings are as follows: Task, which is a general description of the activity; Platform considerations, a summary of the considerations which the function will consider particularly for the planning of a master protocol; and Name of responsible person that the task is assigned to, which is an empty column to remind the sponsor team to complete while planning for the roles and responsibilities in a collaborative platform trial/integrated research platform and facilitate team dynamics.

More details regarding the creation and the internal and external review process for the tool are covered in the publicly available report on “Deliverable D2.10 Final Report on Clinical Operations Best Practices” on the EU-PEARL website.

### Statistical considerations

The use of Complex Clinical Trials, especially platform trials results not only in operational but also in statistical challenges. Statistical challenges specific to platform trials include but are not limited to multiplicity issues due to multiple treatment arms and a common control, the use of concurrent and non-concurrent controls, the use of simulations for trial planning and patient allocation approaches [13–21].

To allow aid in the interpretation of trial results originating from a (potentially perpetual) integrated platform trial, the master protocol should describe which the type of error control(s) to be implemented on the platform and sub-study level. If some hypotheses are considered inferentially independent, a framework should be given to (i) decide based on which criteria hypotheses are defined as related and unrelated, (ii) how to define the families within the platform for which error rates such as family-wise error rate (FWER) should be controlled, and (iii) whether changes of the testing strategy are needed when adding new arms in an ongoing trial.

The large flexibility in platform designs with respect to the addition of new study arms, allocation ratio, and concurrent vs. non-concurrent control data usage, may not allow for a conventional sample size calculation to determine the total platform trial size. The master protocol may only provide an approximation based on a set of assumptions defined in the planning stage, and it should describe the guiding principles in sample size calculation for individual interventions instead.

Similarly, the implementation of interim analyses in platform trials typically differs from conventional clinical trials. To minimize interim analyses being conducted with limited data to inform decisions on whether to stop or not to stop and advance an investigational treatment, the interim analyses in platform trials may need to be triggered by the master protocol, instead of intervention-specific appendices. This will require statistical procedures on the sub-study level, which may handle flexibility in the timing of interim analyses. How to address this is explained within the SAP template as there is not a separate SAP template to be used for the ISAs.

### The use of non-concurrent controls

Platform trials offer the opportunity to add and drop treatment arms during an ongoing trial. When using a shared control, this will lead to non-concurrent and concurrent control patients. Non-concurrent controls refer to trial participants allocated to the control group, who were recruited in periods of time where the “concurrent” experimental treatment was not yet part of the available treatment options. The statistical power can be substantially increased if non-concurrent controls are used in the comparisons of treatment arms with the control comparisons. The later an arm enters the trial, the larger the potential gain in power by including non-concurrent control data. However, a major concern is a bias introduced by time trends that can lead to a change in the response. Examples are temporal changes in the patient population, the disease, the standard of care, or endpoint assessment, etc. [22]. In a publication on the use of external controls [23], of EU-PEARL members together with external authors, the different sources of bias potentially resulting from external controls are elaborated and the relevance of these issues for non-concurrent controls in platform trials is discussed.

### Regulatory feedback

Two interactions with EMA were sought through discussions with the Innovation Task Force (ITF). These discussion meetings provide opportunities for early and product-agnostic engagement on general trial design and methodology topics with regulators (see https://www.ema.europa.eu/en/human-regulatory/research-development/innovation-medicines). Similarly to the FDA, the Critical Path Innovation Meeting (CPIM) (ref added) is also available.

A consultation with the EMA ITF was held on 31 January 2022 with EU-PEARL representatives from the WP4 (MDD). The EMA did not have any issues with the templates. The EMA were supportive of many of the proposed statistical approaches in the study design, including the following:

- Two-step randomization process where participants are first randomized to one of the routes of administration (domain) and then randomly assigned to one of the experimental arms within that domain or its control arm with a participant opt-out feature for domain
- Intra-domain blinding
- The use of a range of placebo allocation ratios and concurrent controls (limited to 35 to 50% because of the risk of inflating the placebo response in MDD if the chance of being allocated to placebo is low)

On whether to permit re-entry and re-randomization of participants into a platform trial, EMA highlighted that further consideration needs to be given to the potential for changes in the patient population over time as this may lead to an increasingly higher rate of resistant participants. The option for a participant re-entering within the same domain or arm should be avoided.

A further consultation with the EMA ITF was held on 9 November 2022 with EU-PEARL representatives from the WP6 (NASH). The EMA was supportive of many of the proposed clinical and statistical features of the study design:

- A randomization process that accounts for patient choice with respect to which cohorts they would be willing to participate in if they meet the eligibility criteria for the platform trial
- Three-tiered level of evidence linked to clinically meaningful effect size as part of the Bayesian decision rules for stopping early for futility or overwhelming efficacy
- The use of concurrent controls across cohorts if the accrual rate allows such data sharing to be performed
- The leveraging of an independent data monitoring committee for decision-making.

There were concerns raised with respect to the controlling of the type I error even in a phase 2 setting if dependence exists between treatment arms (multiple doses of the same investigational treatment or investigational treatments with similar mechanisms of action) where controlling type I error in phase 2b would be justified especially if only a single confirmatory phase 3 study is planned. In addition, the EMA experts raised the point that there is some risk of multiple false positives in case a shared control arm is used. Concerns were also raised on the use of non-concurrent controls and not being able to fully characterize time trends. However, it was noted that, if necessary, the range of potential time trends could be simulated to understand how such trends would impact decision-making.

### FDA interactions

A consultation with the FDA during a Critical Path Innovation Meeting was held on 28 January 2022 with EU-PEARL representatives from the WP6 (NASH). The FDA shared its view that multiplicity control is not required for a phase 2 design across the entire platform trial or within intervention cohorts. In the situation where the sharing of control data across cohorts within a platform trial is being proposed, the FDA recommended attention to the following topics that are relevant for consideration in the use of the suite of templates:

- The expected effect on the main outcome of interest (efficacy or safety) and other/secondary outcomes of interest should be considered. For example, it would not be reasonable to look at safety analyses for injection site reactions for treatments that differ in route of administration (oral vs. injectable).
- The comparison between an investigational drug and the control arm should include the inclusion of only the control participants in a comparison that could have been randomized to that drug (i.e., comparable baseline characteristics and would have been eligible to enter the intervention cohort where the treatment is being evaluated).
- The potential bias created by participant selection to participate in a specific intervention cohort should be considered.
- Any concurrent control should be justified even if the participants would be eligible for multiple cohorts as it is conceivable that the standard of care could change even during the time period of overlap where a concurrent control would be applicable.

During 2022, feedback was obtained from CTCG as part of a formal review of the Master Protocol Template, ISA Template, and guidance for supplementary information to the CTR cover letter.

During the 2nd stakeholder session in 2022, CTCG members added the importance of a consolidated opinion in terms of templates and other aspects. Multinational trials are necessary, but the way how clinical trials are assessed is changing with the new CTR, leading to a multinational assessment. So, the harmonization among regulators, between the different member states, is very important. To get complete high-quality applications for assessment by regulators, it was recommended to have upfront advice, as envisioned by ACT-EU.

The following were the key highlights from their feedback:

1. Providing separate appendices for new interventions is only one option for maintaining the master protocol of the platform study and alternatively, this could be done as one protocol document.
2. Updates needed and were made to fully align with the final EU CTR.
  - Reference to Clinical Trials Regulation (EU No 536/2014).
  - Introduction modified to include guidance for the scientific and social relevance of the trial.
  - New section/text added 4.2.1. Participant input into design. This sub-section elaborates further on participant Input for example design, choice of endpoints, communication during the conduct of the trial, and study results (including informing patients on (interim) study results). This section also includes a justification if there is no engagement of patients.
  - Sect. 8.3.4. Regulatory Reporting Requirements for serious adverse events (SAEs). Provided guidance on the need for clear agreement between sponsors, co-sponsors, involved pharmaceutical companies, and investigators about roles and responsibilities.
  - New section/text added for overdose, medication errors, and misuses or abuses of the medicinal product: Sect. 8.3.9 Overdose, medication errors, and misuses or abuses of the medicinal product.
  - Clarification of data protection and publication policy.
  - Guidance on storage of biological samples.

Changes will need to be made once ICH M11 is finalized as part of a sustainability plan.

### Patient and community engagement

The EU-PEARL patient and community engagement group (PAG) has provided insights into the work of the EU-PEARL suite of templates. PAG was involved in some activities such as defining trial terminology: person vs. patient reviewing and commenting on the Master Protocol Template, and review of the EU-PEARL dictionary in lay terms. Lastly, the benefits of participation of PAG were the inclusion in the planning phase of a project, suiting resources; monitoring and evaluation of activities; and mutual learning process which requires time and confidence. An additional section on participant input was added to the Master Protocol Template.

The relevance of patient engagement in NF is based on a long-term condition with variable disease burden and a rare condition. The variability in disease manifestations requires to first prioritize manifestations. The results of including input from patient representatives in the final selection of manifestations were shown by WP7. WP7 gained patient input in a very early stage, which substantially influenced the NF platform trial design. Some improvements for a future study would be defining the target population and considering trade-offs for scoring.

It is important to get patients and community voices involved in an early research phase. Sometimes, it does not make sense to involve patients at a very early stage, but it is important to get their opinion in what stage they would want to give input. It would be interesting for future projects to have these insights right in the beginning. If patients’ community members are approached very early on, they feel that they are being taken more seriously in their input.

### Sustainability

A sustainability plan for the generic templates was developed. The plan includes the review of the suite of Master Protocol Templates on a regular basis and communication to the user community at least on a yearly basis and when the ICH M11 Protocol Template guidance is finalized at a future date. TransCelerate will incorporate learnings from the EU-PEARL templates into the future versions of the CPT.

## Discussion

Complexity occurs in two dimensions in platform clinical trials: the protocol and the operations. Trials that are complex in any one of these dimensions require special flexibility to easily adapt to variability and change, including in the technologies built to support the trial.

IRP protocol complexity covers specifics in the study design and can be related to the treatment, patient flow through the study, and point-in-time complexities. Protocol complexities might include multiple treatment arms, variable visit schedules, or personalized medicines. Studies that are single- or double-blind require re-randomization or adaptive randomization schemes or add new disease types to the study as it progresses also have significant protocol complexity. The EU-PEARL suite of templates is set up to support these IRP trials build in enough flexibility, visibility, and control to be ready to respond to the variability of the platform trials.

The concept of IRPs is relatively new in clinical development and regulators have only recently started to develop guidelines on challenges like safety oversight of the trials, data transparency and integrity, and control of type 1 errors. Conscious of these challenges, the EU-PEARL Consortium places regulatory endorsement as a key aspect for the successful and effective uptake of any IRP. EU-PEARL primarily engaged with representatives from the European Medicines Agency (EMA) and from European national health authorities (HA), but also with representatives from the Food and Drug Administration (FDA), to foster the global dialog and share insights on the latest guidance on the role of IRPs in regulatory evaluation and approval of new medicinal products. No prototype for such templates for IRPs exists and this suite of templates will facilitate the development of such novel designs.

Furthermore, the suite of templates should increase the quality and enhance consistency within and between sponsors. It may also benefit systematic reviewers in their review of platform trials, which will also contribute to the development of a trust-enhanced environment. The website (http://www.eu-pearl) is fitted with separate download counters for suite of templates and the Platform Trial Best Practice Tool. Although this will enable us to monitor resource downloads, it is less easy to monitor the use of the templates in practice. We therefore encourage user feedback. The suite of templates is open for comments from its publication date. Comments may be submitted via the link in the EU-PEARL website. To maintain its relevance, surveillance of the evolving regulatory and operational landscapes should support future updates.

### Supplementary Information

**Additional file 1.** EU-PEARL Master Protocol Template.

**Additional file 2.** EU-PEARL Intervention Specific Appendix template.

**Additional file 3.** EU-PEARL Statistical Analysis Plan template.

**Additional file 4.** EU-PEARL Guidance to CTR Cover letter.

**Additional file 5.** EU-PEARL Platform Trial Best Practices Tool.

## Data Availability

Not applicable.

## Abbreviations

ACT EU: Accelerating Clinical Trials in the EU
CC&R: Clinical Content & Reuse
CCT: Complex Clinical Trials
CPT: Common Protocol Template
CTA: CT authorization
CTCG: Clinical Trials Coordination Group
CTFG: Clinical Trials Facilitation Group
CTIS: Clinical Trials Information System
CTR: Clinical Trials Regulation
DMC: Data Monitoring Committee
DSMB: Data and Safety Monitoring Board
EC: European Commission
EFPIA: European Federation of Pharmaceutical Industries and Associations
eHR: Electronic health records
EMA: European Medicines Agency
EU: Europe
EU-PEARL: EU Patient-cEntric clinicAl tRial pLatforms
FDA: Food and Drug Administration
FWER: Family-wise error rate
HMA: Heads of Medicines Agencies
ICH: International Council for Harmonisation
IMI: Innovative Medicines Initiative
IMP: Interventional Medicinal Product
IRPs: Integrated research platform trials
ISAs: Intervention Specific Appendices
ITF: Innovation Task Force
IVDR: In Vitro Diagnostic Medical Devices Regulation 2017/746
JU: Joint undertaking
MDD: Major depressive disorder
MPT: Master Protocol Template
NASH: Non-alcoholic steatohepatitis
NIH: National Institutes of Health
NF: Neurofibromatosis
PAG: Patient and Community Engagement Group
SAE: Serious adverse events
SAP: Statistical analysis plan
SMEs: Small and medium-sized enterprises
TB: Tuberculosis
USA: United States of America
WP: Work Package

## References

[1] ICH E6 (R2) Good Clinical Practice - scientific guideline. 1 December 2016. https://www.ema.europa.eu/en/documents/scientific-guideline/ich-guideline-good-clinical-practice-e6r2-step-5_en.pdf.

[2] Accelerating Clinical Trials in the EU (ACT EU). Delivering an EU clinical trials transformation initiative 13 January 2022 https://www.ema.europa.eu/en/Accelerating Clinical Trials in the EU (ACT EU).

[3] Clinical trials - Regulation EU No 536/2014 16 June 2014 https://www.ema.europa.eu/en/Clinical trials - Regulation EU No 536/2014.

[4] TransCelerate Common Protocol Template: https://www.transceleratebiopharmainc.com/assets/clinical-content-reuse-solutions .

[5] L Cash-Gibson, JM Pericàs, C Spiertz, E van de Ketterij, E Molero, F Patalano, D Kalra, A Ussi, A Van Dessel, J Genescà, EU-PEARL: changing the paradigm of clinical trials in Europe, European Journal of Public Health, Volume 31, Issue Supplement_3, October 2021, ckab165.657. 10.1093/eurpub/ckab165.657.

[6] CTFG (2019) Recommendation paper on the initiation and conduct of Complex Clinical Trials, https://www.hma.eu/ctfg.html#c4141 .

[7] Complex Clinical Trials – questions and answers version 2022–05–23 https://health.ec.europa.eu/system/files/2022-06/medicinal_qa_complex_clinical-trials_en.pdf .

[8] Woodcock J, LaVange LM (2017). Master protocols to study multiple therapies, multiple diseases, or both. N Engl J Med.

[9] FDA guidance (2022) Master protocols: efficient clinical trial design strategies to expedite development of oncology drug and biologics guidance for industry https://www.fda.gov/media/120721/download.

[10] Randomized, Embedded, Multifactorial Adaptive Platform trial for Community Acquired Pneumonia (REMAP-CAP): core protocol https://www.remapcap.org/protocol-documents 10.1513/AnnalsATS.202003-192SD PMC7328186 32267771

[11] The European Union In Vitro Diagnostics Regulation (EU) 2017/746 (EU IVDR) 26 May 2022 The European Union In Vitro Diagnostics Regulation – Regulation (EU) 2017/746 (EU IVDR).

[12] Angus DC,  Berry S, Lewis RJ, Al-Beidh F, Arabi Y, van Bentum-Puijk W,  Bhimani Z, Bonten M, Broglio  K, Brunkhorst F, Cheng AC, Chiche JD, De Jong M, Detry M, Goossens H, Gordon A, Green C, Higgins AM, Hullegie SJ, Kruger P, Lamontagne F, Litton E, Marshall J, McGlothlin A, McGuinness S,  Mouncey P, Murthy S, Nichol A, O’Neill GK, Parke R, Parker J, Rohde G,  Rowan K, Turner A, Young P, Derde L, McArthur C, Webb SA (2020). The REMAP-CAP (Randomized Embedded Multifactorial Adaptive Platform for Community-acquired Pneumonia) Study. Rationale and Design. Ann Am Thorac Soc..

[13] Ballarini NM, Burnett T, Jaki T, Jennison C, König F, Posch M (2021). Optimizing subgroup selection in two-stage adaptive enrichment and umbrella designs. Stat Med.

[14] Bofill Roig M, Burgwinkel C, Garczarek U, et al. On the use of non-concurrent controls in platform trials: a scoping review. Trials. 2023;24:408. 10.1186/s13063-023-07398-7. 10.1186/s13063-023-07398-7 PMC10268466 37322532

[15] Bofill Roig M, König F, Meyer E, Posch M. Commentary: Two approaches to analyze platform trials incorporating non-concurrent controls with a common assumption. Clin Trials. 2022;19(5):502-503. 10.1177/17407745221112016. 10.1177/17407745221112016 PMC9523805 35993540

[16] Bofill Roig M, Krotka P, Burman CF, Glimm E, Gold SM, Hees K, Jacko P, Koenig F, Magirr D, Mesenbrink P, Viele K, Posch M (2022). On model-based time trend adjustments in platform trials with non-concurrent controls. BMC Med Res Methodol.

[17] Bofill Roig, M., Melis, G. G., Posch, M., & Koenig, F. (2022). Adaptive clinical trial designs with blinded selection of binary composite endpoints and sample size reassessment. arXiv preprint arXiv:2206.09639. 10.1093/biostatistics/kxac040 PMC10939415 36150142

[18] Collignon, O., Schiel, A., Burman, C., Rufibach, K., Posch, M., & Bretz, F. (2022). Estimands and complex innovative designs. Clinical Pharmacology & Therapeutics, 0(0). 10.1002/cpt.2575 Yes 10.1002/cpt.2575 PMC9790227 35253205

[19] Meyer, E. L., Mesenbrink, P., Dunger-Baldauf, C., Glimm, E., Li, Y., König, F., & EU-PEARL (EU Patient-cEntric clinicAl tRial pLatforms) Consortium (2022). Decision rules for identifying combination therapies in open-entry, randomized controlled platform trials. Pharm Stat.

[20] Robertson, D. S., Wason, J., König, F., Posch, M., & Jaki, T. (2022). Online error control for platform trials. arXiv preprint arXiv:2202.03838. 10.1002/sim.9733 PMC7614610 37005003

[21] Sridhara R, Marchenko O, Jiang Q, Pazdur R, Posch M, Berry S, Lu C (2022). Use of nonconcurrent common control in master protocols in oncology trials: report of an American statistical association biopharmaceutical section open forum discussion. Statistics in Biopharmaceutical Research.

[22] Dodd L, Freidlin B (2021). Platform trials—beware the noncomparable control group April 22, 2021. N Engl J Med.

[23] Burger HU, Gerlinger C, Harbron C, Koch A, Posch M, Rochon J, Schiel A (2021). The use of external controls: to what extent can it currently be recommended?. Pharm Stat.

