# Supplementary material for: Developing generic templates to shape the future for conducting integrated research platform trials
Source: Trials. 2024 Mar 21;25:204. doi: 10.1186/s13063-024-08034-8 (PMC10956223; doi:10.1186/s13063-024-08034-8)
Supplement: Supplementary file 1 — Additional file 1. EU-PEARL Master Protocol Template. [file 13063_2024_8034_MOESM1_ESM.docx]

| 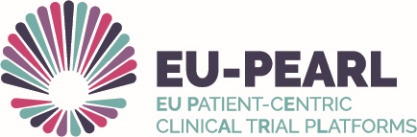 | 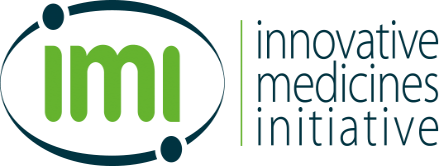 |
| --- | --- |

**Document title:**

**Generic Master Protocol Template**

| **Document history and plan** |
| --- |
| This updated document (V4 25April2023) is based on the **EU-PEARL** **D2.3** ‘Provisional Generic Master Protocol Template and Appendix for IRPs’ (V2 30 April 2021). |
| D2.3 was based on TransCelerate Common Protocol Template CPT V8.0, copyright TransCelerate Biopharma Inc. 2015-2020. |
| Input received from the review committee was assessed for incorporation in the final deliverable D2.6 which is made publicly available at the end of the project (April 2023). |

| Authors | Madhavi Gidh-Jain, Yingwen Dong (Sanofi); Peter Mesenbrink, Ekkehard Glimm, Ian Carbans, Fabienne Baffert (Novartis); Kathy Hersh, Cecile Spiertz, Tobias Mielke, Heidi De Smedt, Eva-Maria Didden, Tom Reijns, Ingela Larsson, Robert Patrizi (Janssen); Tom Parke (Berry); Franz Koenig (MUW); Clelia Di Serio, Paola Rancoita (USR); Olga Sanchez Maroto (VHIR); Burc Aydin, Christine Kubiak, Jacques Demotes (ECRIN); Edwin van de Ketterij (EATRIS) |
| --- | --- |
| Contact person EU-PEARL | ECRIN; Peter Mesenbrink (Novartis) |
| Document version | Version 4 |
| Date | 25April2023 |

The EU-PEARL project has received funding from the Innovative Medicines Initiative 2 Joint Undertaking (JU) under grant agreement No 853966. The JU receives support from the European Union’s Horizon 2020 research and innovation programme and EFPIA and CHILDREN'S TUMOR FOUNDATION, GLOBAL ALLIANCE FOR TB DRUG DEVELOPMENT NON PROFIT ORGANISATION, SPRINGWORKS THERAPEUTICS INC.

**Disclaimer**

These materials are provided AS IS WITHOUT WARRANTY OF ANY KIND, EITHER EXPRESSED OR IMPLIED, INCLUDING, BUT NOT LIMITED TO, THE IMPLIED WARRANTIES OF MERCHANTABILITY, FITNESS FOR A PARTICULAR PURPOSE, OR NONINFRINGEMENT.

EU-PEARL and its members do not accept any responsibility for any loss of any kind including loss of revenue, business, anticipated savings or profits, loss of goodwill or data, or for any indirect consequential loss whatsoever to any person using these materials or acting or refraining from action as a result of the information contained in these materials. Any party using these materials bears sole and complete responsibility for ensuring that the materials, whether modified or not, are suitable for the particular use and are accurate, current, commercially reasonable under the circumstances, and comply with all applicable laws and regulations.

Nothing in this template should be construed to represent or warrant that persons using this template have complied with all applicable laws and regulations. All individuals and organizations using this template bear responsibility for complying with the applicable laws and regulations for the relevant jurisdiction.


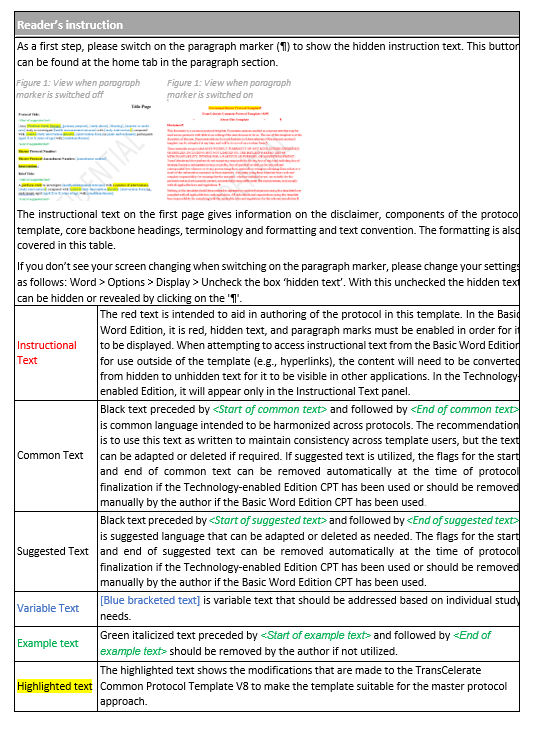


**Provisional Master Protocol Template**

**TransCelerate Common Protocol Template v8.0**

**About This Template**

**Disclaimer**

This document is a common protocol template. It contains sections marked as common text that may be used across protocols with little to no editing if the user chooses to do so. The use of this template is at the discretion of the user. Recommendations for modifications in future releases of the common protocol template can be submitted at any time and will be reviewed on a routine basis.

These materials are provided AS IS WITHOUT WARRANTY OF ANY KIND, EITHER EXPRESSED OR IMPLIED, INCLUDING, BUT NOT LIMITED TO, THE IMPLIED WARRANTIES OF MERCHANTABILITY, FITNESS FOR A PARTICULAR PURPOSE, OR NONINFRINGEMENT. TransCelerate and its members do not accept any responsibility for any loss of any kind including loss of revenue, business, anticipated savings or profits, loss of goodwill or data, or for any indirect consequential loss whatsoever to any person using these materials or acting or refraining from action as a result of the information contained in these materials. Any party using these materials bears sole and complete responsibility for ensuring that the materials, whether modified or not, are suitable for the particular use and are accurate, current, commercially reasonable under the circumstances, and comply with all applicable laws and regulations.

Nothing in this template should be construed to represent or warrant that persons using this template have complied with all applicable laws and regulations. All individuals and organizations using this template bear responsibility for complying with the applicable laws and regulations for the relevant jurisdiction.

Throughout this protocol template, the yellow highlighted text shows the modifications that are made to the TransCelerate Common Protocol Template V8 to make the template suitable for the master protocol approach.

**Components of the Protocol Template**

- The **Core Backbone** contains protocol information common to all phases, study populations, and therapeutic areas. The core backbone is streamlined and focused on the sites’ needs.
- **Libraries** group and store content that will be inserted into the core backbone and contain specific information related to therapeutic area, study intervention, country, and study population (eg, participant, healthy volunteer). For pediatric or adult/pediatric studies, include the content contained in the pediatric library.
- **Appendices** provide additional information that can be accessed when needed (eg, abbreviations, standard content regarding adverse event [AE] definitions).

**Core Backbone Headings**

- Level 1 and 2 headings should be consistent across protocols that use the CPT for reference and mapping purposes. The structure of this template aligns with the structure of the United States National Institutes of Health (NIH) and Food and Drug Administration (FDA) Clinical Trials Protocol Template.
- Level 1 and 2 headings should not be deleted. If they are not relevant to the study, not applicable should be inserted so that the numbering of subsequent sections is not changed.
- Level 3 and subsequent headings are suggested and can be deleted/added/modified as needed with the exception of those in Section 8.3 relating to Adverse Events, which are International Council on Harmonisation (ICH)-/regulatory agency-required wording and must be included.

**Terminology**

- The following terminology has been selected for use within TransCelerate common templates (protocol, statistical analysis plan [SAP], and clinical study report [CSR]) and is considered to be appropriate for all phases, study populations, and therapeutic areas.
  - *Participant* is used rather than subject, healthy volunteer, or patient.
  - *Study intervention* is used rather than study drug. Study intervention covers all types of investigational and noninvestigational products including medical devices and vaccines.
    - Study intervention is defined as investigational intervention(s), marketed product(s), placebo, or medical device(s) intended to be administered to a study participant per protocol.
  - Efficacy is used for drugs.
- The following terminology has been selected for use within EU PEARL common templates:
  - The master protocol is the document which describes the overall clinical study design applicable to all related interventions, such as the clinical study rationale, objectives, endpoints, benefit-risk assessment, shared procedures regarding safety monitoring and reporting, and a common screening platform dictating participant eligibility and/or treatment allocation (CTFG, 2019).
  - The Intervention Specific Appendix (ISA) is the appendix to the related master protocol which describes the specific features of the intervention and treatment of participants assigned to that intervention or the control group to which it is compared (IMI call). Each intervention will have a separate ISA. Together, a master protocol and an ISA define all the elements needed to conduct a study. Instead of ‘Intervention Specific Appendix’ alternatively the term sub- protocol or, if the specification is preferred, ‘Intervention Specific Protocol’, could be used.

**Formatting and Text Conventions**

- Common Text: Black font preceded by *<Start of common text>* and followed by *<End of common text>* is common language intended to be harmonized across protocols. The recommendation is to use this text as written to maintain consistency across template users, but the text can be adapted or deleted if required. If suggested text is utilized, the flags for the start and end of common text can be removed automatically at the time of protocol finalization if the Technology-enabled Edition CPT has been used or should be removed manually by the author if the Basic Word Edition CPT has been used.
- Suggested Text: Black text preceded by *<Start of suggested text>* and followed by *<End of suggested text>* is suggested language that can be adapted or deleted as needed. The flags for the start and end of suggested text can be removed automatically at the time of protocol finalization if the Technology-enabled Edition CPT has been used or should be removed manually by the author if the Basic Word Edition CPT has been used.
- Variable Text: Blue bracketed text is variable text that should be addressed based on individual study needs.
- Example Text: Green italicized text preceded by *<Start of example text>* and followed by *<End of example text>* should be removed by the author if not utilized.
- Instructional Text: Is intended to aid in authoring of the protocol in this template. In the Basic Word Edition, it is red, hidden text, and paragraph marks must be enabled in order for it to be displayed. When attempting to access instructional text from the Basic Word Edition for use outside of the template (eg, hyperlinks), the content will need to be converted from hidden to unhidden text for it to be visible in other applications. In the Technology-enabled Edition, it will appear only in the Instructional Text panel.
- It is necessary that all documents are identified with:
  - A common identifier (e.g. an acronym) in the title to be used for the master protocol and all sub- protocols
  - A specific identifier (e.g. a number) following the common identifier for each sub-protocol
  - These identifiers should also be used in the names of the electronic documents (e.g. pdf). When submitting substantial modifications, reference should be made to relevant sub-protocol common and specific identifier(s).
- Similar information is provided in more than one chapter: e.g. description study population, DMC and rationale.

Title Page

**Protocol Title:**

Protocol Title: The protocol should have a descriptive title that identifies the study sufficiently to ensure it is immediately evident what the study is investigating and on whom, and to allow retrieval from literature or internet searches.

<Start of suggested text>

[A(n) [Platform Study Design] [primary purpose], [study phase], [blinding], [number or multi-arm] study to investigate [health measurement/outcome] with [study interventions] compared with [control/study intervention/placebo] [intervention form] in [male and/or female] participants [aged X to X years of age] with [condition/disease]

<End of suggested text>

**Example:**

A Multicenter, Adaptive, Randomized, Double-Blind Phase 2/3 Standard-of-Care Controlled Platform Study of the Safety and Efficacy of Investigational Therapeutics evaluating the speed of recovery for male and female participants aged 18 years and older with COVID-19.

A structured title should contain details of participants, interventions (and acronyms if relevant), comparison groups, outcomes, and study design. Use of the terms below will ensure alignment with Clinical Trials Registry Data Element Definitions.

Enter values from the list given for each of the indicated fields to complete.

**Intervention Model:**

- Platform study

**Primary Purpose:**

- Treatment: One or more interventions are being evaluated for treating a disease, syndrome, or condition.
- Prevention: One or more interventions are being assessed for preventing the development of a specific disease or health condition.

**Study Phase:**

See definitions under heading for Study Phase and enter same phase in each place.

**Blinding**

Insert/copy definition from Overall Design section.

**Number of intervention cohorts**:

Numeric value for the maximum number of intervention cohorts in the study, overall and at any one time, if applicable.

**Health Measurement/Outcome:**

What is the primary outcome as given in the objectives being examined to determine the effect from the intervention? This should be included in the protocol title written in lay language, eg, measure the reduction in bad cholesterol; other potential terms: treat, delay, confirm, predict, identify, reduce, correct, reverse, lower, decrease, increase, or improve. Ensure this is written as an action/verb.

**Intervention Name**:

Refer to the applicable intervention specific appendix (ISA)

**Intervention Form:**

See applicable ISA

**Participant Sex:**

eg, male, female, male and female

**Participant Age Range:**

eg, 18-65 years of age, 10-18 years of age

**Condition/Disease:**

The disease, disorder, syndrome, illness, or injury, etc that is being studied

**Master Protocol Number:**

Sponsor specific decision depending on whether each ISA is registered separately.

**Master Protocol Amendment Number:** [amendment number]

**Intervention:**

Each intervention will be described in the applicable ISA.

**Brief Title:**

<Start of suggested text>

A platform study to investigate [health measurement/outcome] with a number of interventions [study interventions] compared with [control/study intervention/placebo] [intervention form] in participants aged [aged X to X years of age] with [condition/disease]

<End of suggested text>

Example:

Therapeutics for Inpatients with COVID-19 (TICO)

Short title should be sufficiently detailed to make clear to a lay reader what the study is about and suitable for use as the Brief Title in ClinicalTrials.gov and for use with informed consents and ethics committee submissions.

Based on NIH expectations and participant preferences, the optimal Brief Title on ClinicalTrials.gov includes the following data elements: condition/disease, health measurements/observations, intervention name, intervention form, participant age range, and participant sex.

Definitions of these terms are in the guidance following the additional details section.

Additional details:

- Reference to *participants* as the preferred term
- All abbreviations are defined.
- Does not end with a period
- Technical study design terms are avoided.
- Limited to 300 characters

Study Phase: [study phase]

Please select one of the values for this field:

- N/A: for studies without phases (eg, studies of behavioral interventions)

- Early Phase 1: exploratory studies, involving very limited human exposure, with no therapeutic or diagnostic intent (eg, screening studies, microdose studies)

- Phase 1: includes initial studies to determine the metabolism and pharmacologic actions of drugs in humans, the side effects associated with increasing doses, and to gain early evidence of effectiveness; may include healthy participants and/or participants with the disease of interest

- Phase 1/Phase 2: for studies that are a combination of Phases 1 and 2

- Phase 2: includes controlled clinical studies conducted to evaluate the effectiveness of the intervention for a particular indication or indications in participants with the disease or condition under study and to determine the common short-term side effects and risks

- Phase 2/Phase 3: for studies that are a combination of Phases 2 and 3

- Phase 3: studies conducted after preliminary evidence suggesting effectiveness of the intervention has been obtained, and are intended to gather additional information to evaluate the overall benefit-risk relationship of the intervention

- Phase 4: studies of FDA-approved interventions to delineate additional information including risks, benefits, and optimal use

**[Acronym]**:

Acronym or abbreviation used publicly to identify the clinical study, if any.
Limit: 14 characters. Delete if not applicable.

**Sponsor Name:**

List the sponsor responsible for the management of the platform study.

The companies who are providing its investigational medicinal product/study drug (IMP) (IMP Owner or intervention owner) will be listed in the ISAs. Typically, in a platform trial there are multiple (often unaffiliated) intervention owners contributing interventions to different (or the same in the case of combination treatment trials) ISAs of the platform trial.

Legal Registered Address:

The sponsor name and legal registered address must be included.

In some countries, the clinical study sponsor may be the local affiliate company (or designee). If applicable, the details of the alternative sponsor and contact person in the territory should be provided to the relevant regulatory authority as part of the clinical study application and should not be included in the protocol.

Regulatory Agency Identifier Number(s):

Include all numbers that are applicable for the study and available at the time of protocol or amendment finalization, eg, investigational new drug (IND) number (include the center number, IND/IDE number, serial number), World Health Organization (WHO) universal trial number, CTIS number (EU trial number), ClinicalTrials.gov. Add type and number as applicable.

Refer to ISA and adjust text above to reflect how IND and EudraCT will be handled. Add IND number for common control arm if included in master.

| **Registry** | **ID** |
| --- | --- |
|  |  |

**Approval Date:**

Sponsor Signatory:

| **[Name]**  **[Title]** |  | **Date** |
| --- | --- | --- |

Medical Monitor Name and Contact Information [will be provided separately OR can be found in XX]

The investigator signature page is generated internally as a stand-alone document and should be provided to the investigator for signature alongside the final protocol. The investigator should retain the original in the site study files and return a copy to the sponsor for archiving in the trial master file (TMF). In case of a protocol amendment, ensure that the protocol version is noted on the investigator signature page.

This also needs to be done for each ISA that the investigator participates in.

Protocol Amendment Summary of Changes Table

**Delete this section if this is not an amendment.**

The master protocol and the ISA can be amended separately. Therefore, the amendment numbers for the master protocol and the ISAs may not align. The amendment history for the master protocol will be in the master protocol and the amendment history for each ISA will be in the applicable ISA.

Protocols should not be developed with the intent to amend; however, if an amendment is required, the following process and template is recommended. Companies should modify this process as appropriate (eg, naming conventions, designation of substantial/nonsubstantial amendment status) to ensure alignment with their internal processes and systems.

**GENERAL INSTRUCTIONS:**

- Protocols should be amended by making the changes directly within the protocol.
- In addition to the summary of changes table, incorporate the changes made as a result of the amendment into the respective CPT sections and create
  - a new clean version
  - a new version with the changes highlighted (ie, tracked changes) to be provided to the health authorities, if required.
- NOTE: For substantial amendments: use the tracked-changes version of the protocol to create a separate document with a tabular listing detailing section changed, initial wording, amended or new wording, reason/justification for change, and reason for substantial amendment as this is now required by many health authorities.
- Include the heading Protocol Amendment Summary of Changes in the table of contents (TOC) as a non-numbered heading.
- Modify the protocol number as appropriate throughout the document as specific to the company (eg, title page, page headers) to designate status as an amendment.
- See Appendix 10, Protocol Amendment History for further instructions and examples for completing this section.
- The common text section titled Document History should be completed for each amendment.
- Amendments should appear in reverse chronological order with the most recent at the top (eg, Amendment 3, 2, 1).
- The Protocol Amendment Summary of Changes table for the current amendment should be maintained directly in front of the TOC.
- The Protocol Amendment Summary of Changes Table(s) for the previous amendment(s) should be moved to Appendix 10, Protocol Amendment History.
- Group changes by rationale and list rationales by order of importance, with the rationale for the most important study design changes listed first. Under each rationale, list changes in order of occurrence in the protocol.

Relevant changes may have been made to the protocol template since the original protocol or last amendment was issued. Check the template change control documentation and discuss with the team to ensure all relevant changes have been added to the protocol and included in the Protocol Amendment Summary of Changes Table.

***NAMING CONVENTIONS*** for differentiation of types of amendments (eg, global, country-specific, site‑specific):

Use International Organization for Standardization (ISO)-Alpha 3 Codes from the United Nations Statistics Department for 3-letter codes to represent country or area name in country-specific amendments: https://www.nationsonline.org/oneworld/countrycodes.htm

Examples can be found in Appendix 10, Protocol Amendment History.

***NUMBERING CONVENTIONS***

- Global amendments should be sequentially numbered (eg, Amendment 1, Amendment 2, Amendment 3, etc).
- Country-specific amendments should list the 3-digit ISO-Alpha 3 Codes (link above) with sequential numbering (eg, for France, the 3-digit code is FRA. The first country-specific amendment for France should be numbered Amendment FRA-1. If a second amendment is required with content specific to France, it would be Amendment FRA-2.).
- When adding an amendment ensure that the country-specific changes are maintained with each global update.
  - A country-specific amendment to a global amendment

or

- - A global amendment to a country-specific amendment.

Examples can be found in Appendix 10, Protocol Amendment History.

***DOCUMENT HISTORY***

- The Document History table should be inserted at the beginning of each amendment and contain the document number and date for each amendment.
- Global amendments should not list the country- or site-specific amendments in the table.
- Country- and site-specific amendments should list the global amendments.
- Country-specific amendments should not list the site-specific amendments.
- Site-specific amendments should only list country-specific amendments for that specific country.
- If an amendment with identical changes is needed for multiple countries/areas/sites, they may be named as:
  - Region 1 (list country/area codes as ISO-Alpha 3 Codes from the United Nations Statistics Department as noted above)
  - Region 2 (list country/area codes as ISO-Alpha 3 Codes the from United Nations Statistics Department as noted above)
  - Site-specific SS-1 (site numbers)

The rationale for not including the entire list of amendments in the Document History table is that the global amendments apply to all countries and sites, while the country- and site-specific amendments are just that, specific, and therefore do not apply to all.

Examples can be found in Appendix 10, Protocol Amendment History.

Amendment history for each ISA will be described within the applicable ISA.

List dates of original protocol and all amendments in reverse chronological order.

Use table below for Document history. Alternately, the table can be removed and a structured cover letter capturing platform study history may be used.

Strongly recommended not to have country-specific or site-specific amendments, as country-specific or site-specific amendments will add complexity to platform study and constraint the ability to analyze data.

<Start of common text>

| DOCUMENT HISTORY | |
| --- | --- |
| Document | Date |
| [Amendment X] | [Day-Mon-Year] |
| [Amendment X] | [Day-Mon-Year] |
| [Amendment X] | [Day-Mon-Year] |
| Original Protocol | [Day-Mon-Year] |

Amendment [X] (Day-Month-Year)

Include the following statement if this amendment will be implemented in any European Union (EU) member state. Include the last phrase for nonsubstantial amendments only.

<Start of suggested text>

This amendment is considered to be [substantial] [nonsubstantial] based on the criteria set forth in Article 10(a) of Directive 2001/20/EC and Regulation (EU) No 536/2014 of the European Parliament and the Council of the European Union [because it neither significantly impacts the safety or physical/mental integrity of participants nor the scientific value of the study].

Health Authority and Institutional Review Board/Ethics Committee approval is needed before implementation of changes if the amendment is substantial.

<End of suggested text>

Overall Rationale for the Amendment:

The overall rationale (one primary driver) for the changes implemented in the protocol amendment should be provided. In addition, provide a high-level description of the change(s) and a brief scientific rationale for specific items outlined in the table provided (eg, changes to individual inclusion/exclusion criteria). See Appendix 10, Protocol Amendment History for examples of format and green text for sample content.

[INSERT rationale statement]

| Section # and Name | Description of Change | Brief Rationale |
| --- | --- | --- |
| [INSERT] | [INSERT] | [INSERT] |
| [INSERT] | [INSERT] | [INSERT] |
| [INSERT] | [INSERT] | [INSERT] |
|  |  |  |
|  |  |  |

<End of common text>

Table of Contents

[1. Protocol Summary 19](#_Toc127288275)

[1.1. Synopsis 19](#_Toc127288276)

[1.2. Schema 24](#_Toc127288277)

[1.3. Schedule of Activities (SoA) 26](#_Toc127288278)

[2. Introduction 30](#_Toc127288279)

[2.1. Study Rationale 31](#_Toc127288280)

[2.2. Background 32](#_Toc127288281)

[2.3. Benefit/Risk Assessment 32](#_Toc127288282)

[2.3.1. Risk Assessment 32](#_Toc127288283)

[2.3.2. Benefit Assessment 33](#_Toc127288284)

[2.3.3. Overall Benefit Risk Conclusion 33](#_Toc127288285)

[3. Objectives, Endpoints, and Estimands 34](#_Toc127288286)

[4. Study Design 36](#_Toc127288287)

[4.1. Overall Design 36](#_Toc127288288)

[4.2. Scientific Rationale for Study Design 37](#_Toc127288289)

[4.2.1. Participant Input into Design 37](#_Toc127288290)

[4.3. Justification for Dose 37](#_Toc127288291)

[4.4. End of Study Definition 37](#_Toc127288292)

[5. Study Population 39](#_Toc127288293)

[5.1. Inclusion Criteria 39](#_Toc127288294)

[5.2. Exclusion Criteria 40](#_Toc127288295)

[5.3. Lifestyle Considerations 40](#_Toc127288296)

[5.3.1. Meals and Dietary Restrictions 41](#_Toc127288297)

[5.3.2. Caffeine, Alcohol, and Tobacco 41](#_Toc127288298)

[5.3.3. Activity 41](#_Toc127288299)

[5.4. Screen Failures 41](#_Toc127288300)

[5.5. Criteria for Temporarily Delaying [Enrollment/Randomization/Administration of Study Intervention] 42](#_Toc127288301)

[6. Study Intervention(s) and Concomitant Therapy 43](#_Toc127288302)

[6.1. Study Intervention(s) Administered 43](#_Toc127288303)

[6.1.1. Medical Devices 43](#_Toc127288304)

[6.2. Preparation, Handling, Storage, and Accountability 44](#_Toc127288305)

[6.3. Measures to Minimize Bias: Randomization and Blinding 44](#_Toc127288306)

[6.4. Study Intervention Compliance 47](#_Toc127288307)

[6.5. Dose Modification 47](#_Toc127288308)

[6.5.1. Retreatment Criteria 47](#_Toc127288309)

[6.6. Continued Access to Study Intervention after the End of the Study 48](#_Toc127288310)

[6.7. Treatment of Overdose 48](#_Toc127288311)

[6.8. Concomitant Therapy 48](#_Toc127288312)

[6.8.1. Rescue Medicine 49](#_Toc127288313)

[7. Discontinuation of Study Intervention and Participant Discontinuation/Withdrawal 50](#_Toc127288314)

[7.1. Discontinuation of Study Intervention 50](#_Toc127288315)

[7.1.1. Liver Chemistry Stopping Criteria 50](#_Toc127288316)

[7.1.2. QTc Stopping Criteria 50](#_Toc127288317)

[7.1.3. Temporary Discontinuation 51](#_Toc127288318)

[7.1.4. Rechallenge 51](#_Toc127288319)

[7.2. Participant Discontinuation/Withdrawal from the Study 51](#_Toc127288320)

[7.3. Lost to Follow up 52](#_Toc127288321)

[8. Study Assessments and Procedures 53](#_Toc127288322)

[8.1. [Efficacy and/or Immunogenicity] Assessments 53](#_Toc127288323)

[8.2. Safety Assessments 54](#_Toc127288324)

[8.2.1. Physical Examinations 54](#_Toc127288325)

[8.2.2. Vital Signs 54](#_Toc127288326)

[8.2.3. Electrocardiograms 54](#_Toc127288327)

[8.2.4. Clinical Safety Laboratory Tests 55](#_Toc127288328)

[8.2.5. Pregnancy Testing 56](#_Toc127288329)

[8.2.6. Suicidal Ideation and Behavior Risk Monitoring 56](#_Toc127288330)

[8.3. Adverse Events (AEs) Serious Adverse Events (SAEs), and Other Safety Reporting 56](#_Toc127288331)

[8.3.1. Time Period and Frequency for Collecting AE and SAE Information 57](#_Toc127288332)

[8.3.2. Method of Detecting AEs and SAEs 57](#_Toc127288333)

[8.3.3. Follow-up of AEs and SAEs 57](#_Toc127288334)

[8.3.4. Regulatory Reporting Requirements for SAEs 57](#_Toc127288335)

[8.3.5. Pregnancy 58](#_Toc127288336)

[8.3.6. Cardiovascular and Death Events 59](#_Toc127288337)

[8.3.7. Disease-related Events and/or Disease-related Outcomes Not Qualifying as AEs or SAEs 59](#_Toc127288338)

[8.3.8. Adverse Events of Special Interest 60](#_Toc127288339)

[8.3.9. Overdose, medication errors, misuses or abuses of medicinal product 60](#_Toc127288340)

[8.3.10. Medical Device Deficiencies 61](#_Toc127288341)

[8.4. Pharmacokinetics 62](#_Toc127288342)

[8.5. Genetics 63](#_Toc127288343)

[8.6. Biomarkers 63](#_Toc127288344)

[8.7. Immunogenicity Assessments 64](#_Toc127288345)

[8.8. [Health Economics OR Medical Resource Utilization and Health Economics] 64](#_Toc127288346)

[8.11 Use of Biological Samples and Data for Future Research 65](#_Toc127288347)

[9. Statistical Considerations 67](#_Toc127288348)

[9.1. Statistical Hypotheses 67](#_Toc127288349)

[9.1.1. Multiplicity Adjustment 68](#_Toc127288350)

[9.2. Analysis Sets 68](#_Toc127288351)

[9.3. Statistical Analyses 70](#_Toc127288352)

[9.3.1. General Considerations 70](#_Toc127288353)

[9.3.2. Primary Endpoint(s)/Estimand(s) Analysis 71](#_Toc127288354)

[9.3.3. Secondary [Endpoint(s)/Estimand(s)] Analysis 71](#_Toc127288355)

[9.3.4. [Tertiary/Exploratory/Other] [Endpoint(s)/Estimand(s)] Analysis 72](#_Toc127288356)

[9.3.5. [Other] Safety Analyses 72](#_Toc127288357)

[9.3.6. Other Analyses 73](#_Toc127288358)

[9.4. Interim Analysis 74](#_Toc127288359)

[9.5. Sample Size Determination 75](#_Toc127288360)

[10. Supporting Documentation and Operational Considerations 78](#_Toc127288361)

[10.1. Appendix 1: Regulatory, Ethical, and Study Oversight Considerations 78](#_Toc127288362)

[10.1.1. Regulatory and Ethical Considerations 78](#_Toc127288363)

[10.1.2. Financial Disclosure 79](#_Toc127288364)

[10.1.3. Informed Consent Process 80](#_Toc127288365)

[10.1.4. Data Protection 81](#_Toc127288366)

[10.1.5. Committees Structure 82](#_Toc127288367)

[10.1.6. Dissemination of Clinical Study Data 83](#_Toc127288368)

[10.1.7. Data Quality Assurance 83](#_Toc127288369)

[10.1.8. Source Documents 84](#_Toc127288370)

[10.1.9. Study and Site Start and Closure 84](#_Toc127288371)

[10.1.10. Publication Policy 85](#_Toc127288372)

[10.2. Appendix 2: Clinical Laboratory Tests 86](#_Toc127288373)

[10.3. Appendix 3: AEs and SAEs: Definitions and Procedures for Recording, Evaluating, Follow-up, and Reporting 89](#_Toc127288374)

[10.3.1. Definition of AE 89](#_Toc127288375)

[10.3.2. Definition of SAE 91](#_Toc127288376)

[10.3.3. Recording and Follow-Up of AE and/or SAE 92](#_Toc127288377)

[10.3.4. Reporting of SAEs 94](#_Toc127288378)

[10.4. Appendix 4: Contraceptive and Barrier Guidance 95](#_Toc127288379)

[10.4.1. Definitions 95](#_Toc127288380)

[10.4.2. Contraception Guidance 95](#_Toc127288381)

[10.5. Appendix 5: Genetics 96](#_Toc127288382)

[10.6. Appendix 6: Liver Safety: Suggested Actions and Follow-up Assessments [and Study Intervention Restart/Rechallenge Guidelines] 97](#_Toc127288383)

[10.7. Appendix 7: AEs, ADEs, SAEs, SADEs, USADEs and Device Deficiencies: Definitions and Procedures for Recording, Evaluating, Follow-up, and Reporting in Medical Device Studies 98](#_Toc127288384)

[10.7.1. Definition of Medical Device AE and ADE 98](#_Toc127288385)

[10.7.2. Definition of Medical Device SAE, SADE and USADE 99](#_Toc127288386)

[10.7.3. Definition of Device Deficiency 99](#_Toc127288387)

[10.7.4. Recording and Follow-Up of AE and/or SAE and Device Deficiencies 100](#_Toc127288388)

[10.7.5. Reporting of SAEs 102](#_Toc127288389)

[10.7.6. Reporting of SADEs 103](#_Toc127288390)

[10.8. Appendix 8: Country-specific Requirements 104](#_Toc127288391)

[10.9. Appendix 9: Abbreviations [and Definitions] 105](#_Toc127288392)

[10.10. Appendix 10: Protocol Amendment History 106](#_Toc127288393)

[10.11. APPENDIX 10: Collection, storage and future use of data and human biological samples 109](#_Toc127288394)

[10.11.1. Compliance with Member State applicable rules for the collection, storage and future use of human biological samples (Article 7.1h) 109](#_Toc127288395)

[10.11.2. Compliance with Member State applicable rules for the collection, storage and future use of (personal) data (article 7 (1 d) of EU Regulation 536/2014) 109](#_Toc127288396)

[10.12. Appendix 11: Additional Appendices (if needed) 109](#_Toc127288397)

[11. References 110](#_Toc127288398)

# Protocol Summary

## Synopsis

The protocol synopsis is a short (1 to 2 pages) summary of the key points of the protocol. This section of the protocol should be completed after the main text to ensure consistency with the main text.

The purpose of the protocol synopsis is to provide a concise outline of the key aspects of the study. It may be used for European Union (EU) clinical trial applications (CTAs) and for other external bodies such as institutional review boards [IRBs]/independent ethics committees [IECs]). Its level of detail should not dissuade/discourage the investigator from referring to the main text of the protocol.

**Protocol Title:**

Ensure wording here matches the title page.

**Brief Title:**

Ensure wording here matches the title page.

Rationale:

The synopsis text should be taken from the main text.

Objectives, Endpoints, and Estimands:

State the primary and secondary objectives and associated endpoints. Be consistent with the main text of the protocol in text and format.

Endpoints and estimands: This should be a high-level description.

| Objectives | Endpoints |
| --- | --- |
| Primary |  |
|  |  |
| Secondary |  |
|  |  |

Overall Design:

<Start of common text>

A platform study is defined by the master protocol, which describes the overall clinical study design applicable to all related interventions.  The intervention-specific appendix (ISA) is the appendix to the related master protocol which describes the specific features of the intervention and treatment of participants randomized to that intervention or to the control group to which the intervention will be compared. The master protocol together with an ISA has all the information needed to conduct an intervention cohort.

<End of common text>

Be sure the text included in this section is consistent with the text in other sections such as inclusion/exclusion criteria and concomitant medications.

Briefly state:

- Type of design and control method (eg, placebo, active comparator, low dose, historical, or none [uncontrolled]), single or multicenter. Include the kind of control group to be used, if any.
- High-level description of the study population (eg, participants with acute lung injury, etc). Main inclusion and exclusion criteria.
- Level and method of blinding (eg, open-label, single-blind, double-blind, double-blind [sponsor unblinded], matching placebo, double-dummy) and the methods to be used to minimize bias on the part of participants, investigators, and analysts.
- Study intervention assignment method (eg, randomization, stratification, both). Do NOT state block size. If assignment to intervention is by randomization, describe when randomization occurs relative to screening.
- Refer to use of an independent data monitoring committee, dose-escalation committee, or some other pre-specified independent committee who will have accountability for the stopping of interventions for futility, toxicity, or superior efficacy.
- A separate independent group will provide oversight on the adding of intervention(s) to the platform study.
- The Sponsor will be responsible for determining any revisions required to the overall platform study design based on the adding of intervention(s) and whether an amendment of the platform study protocol is necessary.

**Brief Summary:**

Brief Summary is a short description of the clinical study, including a brief statement of the clinical study’s hypothesis. It should be sufficiently detailed to make clear to a lay reader what the study is about and can be used for informed consents and ethics committee submissions as well as the Brief Summary in ClinicalTrials.gov.

Based on NIH expectations and participant preferences, the optimal Brief Summary on ClinicalTrials.gov includes the following data elements: condition/disease, study duration, treatment duration, health measurement/observation, and visit frequency.

Additional details:

- Complete sentences
- All abbreviations are defined.
- Formatting includes paragraphs and/or bullets.
- Bibliographic references are avoided, as well as any reference to external documents.
- Limit: 5000 characters

**Example:**

The purpose of this study is to determine the optimal care for participants with SARS-CoV-2 virus-induced pneumonia with or without cytokine release syndrome (CRS)

*For Text highlighted in Blue, provide in each ISA and remove from MP if not applicable*

**Health Measurement/Observation**: What is the primary outcome as given in the objectives being examined to determine the effect from the intervention. This should be included in the protocol title written in lay language, eg, measure the reduction in bad cholesterol; other potential terms: treat, delay, confirm, predict, identify, reduce, correct, reverse, lower, decrease, increase, or improve.

**Study Intervention and Intervention Form:**

Text for this field should be taken from the Intervention table in Section 6.1 (if applicable).

**Condition/Disease**:

The disease, disorder, syndrome, illness, or injury etc. that is being studied included in the protocol title written in lay language. Refer to <https://hso.research.uiowa.edu/medical-terms-lay-language>.

**Study Duration**:

The maximum length of time a participant can be in the study

**Treatment Duration**:

The length of time the intervention will be provided or administered; examples: 3 weeks, 12 months

**Visit Frequency**:

The number of times or how often study visits will take place during the study duration; examples: every 3 weeks, 5 continuous days in the hospital

Complete the suggested text paragraph with values from the list given for each of the indicated fields.

Example:

The purpose of this study is to measure the safety and decrease in agitation with Addiryn tablets compared with placebo tablets in participants with Alzheimer’s dementia. Study details include:

- Study duration: 24 weeks
- Treatment duration: 12 weeks
- Visit frequency: every 3 weeks

<Start of suggested text>

The purpose of this study is to measure [health measurement/observation] with [study interventions] [intervention form] [compared with OR in combination with] [study interventions] [intervention form] in [participants with [disease/condition]/].

Study details include:

- The study duration will be up to [numerical value, eg, days, weeks, months].
- The treatment duration will be up to [numerical value, eg, days, weeks, months].
- The visit frequency will be [X].

<End of suggested text>

Number of Participants:

- In most platform studies, the eventual total number of participants will not be known at study start. Details for number of participants per intervention cohort will be provided in applicable ISA.
- To comply with registry disclosure requirements, a maximum number of participants expected should be provided. This may be driven by the maximum number of investigation cohorts that will potentially be evaluated in the platform study.
- Adaptive design only: If the sample for an intervention cohort is not fixed, describe the range of potential sample sizes for each investigational arm, i.e., the minimum sample size an ISA can be stopped (for futility/efficacy) and the maximum sample size.
- Cross reference Section 9.5 Sample Size Determination and ensure that section clearly explains how screening failures and nonevaluable participants are defined.
- Choose one of the two options listed and modify as appropriate

<Start of example text>

Existing simulations indicate that when a single investigational treatment is beneficial, the power to be determined superior to control will be greater than [X%] when there is at least a [X%] decrease in the risk of <primary efficacy endpoint> for the funded sample size of [X] participants. The timing of these conclusions of superiority have a median time of less than [X] participants. The probability that an investigational treatment will be determined superior to the control when in truth the two are equal (Type I error) will be typically less than [X%].

**OR**

Approximately [X] participants will be screened to achieve [X] [randomly assigned/enrolled] to a minimum number of participants to each study intervention in the platform study and [X] evaluable participants for an estimated minimum total of [X] evaluable participants per intervention group in the platform study.

**OR**

A maximum of [X] participants will be [randomly assigned/enrolled] to study intervention in the platform study such that approximately [X] evaluable participants will complete the platform study if at least [X] interventions are evaluated in the platform study.

**Note**: *Enrolled* means a participant’s or their legally acceptable representative’s agreement to participate in the platform study following completion of the informed consent process [and screening]. Potential participants who are screened for the purpose of determining eligibility for the platform study, but do not participate in the platform study, are not considered enrolled, unless otherwise specified by the protocol. A participant will be considered enrolled if the informed consent is not withdrawn prior to participating in any study activity after screening.

<End of example text>

Intervention Groups and Duration:

Details in applicable ISA.

Data Monitoring/Other Committee: [Yes/No]

Use of an independent data monitoring committee, dose-escalation committee, or similar review group. Details should be included in Appendix 10.1.5 Committees Structure.

Sponsor

The group or organization who is responsible and accountable for the conduct of the study either globally or regionally.

Describe who is the Sponsor of the overall platform study and if the Sponsor is not from Industry describe the governance process between those pharmaceutical companies providing study treatments to the platform study and how they will interact with the Sponsor.

<Start of suggested text>

Study Management Group

A Study Management Group (SMG) will be formed comprising the Principal Investigator, other co-investigators and the Sponsor organization responsible for the monitoring of the platform study. The membership of the SMG may be expanded if other groups wish to participate. The SMG will be responsible for the day-to-day running and management of the study and will meet by teleconference at least every 3 months.

Steering Committee

A Study Steering Committee (SSC) will be formed to provide overall supervision for the study and provide advice through its independent chair. The ultimate decision for the continuation of the study lies with the SSC. The SSC will meet regularly as agreed to in the SSC charter (available as a separate document).

IMP or Intervention Selection Committee

IMP or Intervention Selection Committee, including independent experts (which may have different types of expertise, such as scientific, ethics, supply chain expertise, depending on specific needs), to advise on the inclusion of new IMPs into the trial. This committee will continue to evaluate potential arms for the trial and prioritize their importance based upon newly emerging preclinical and clinical data. The Charter provides details on the decision rules and what decision criteria should be considered.

Endpoint Committee:

An independent group will be established to evaluate the endpoints that have been observed in the platform study and determine if there is substantive evidence to confirm that a clinical event has occurred. The details of the scope of this committee will be described in the Endpoint Committee Charter.

Adjudication Committee (if applicable):

An independent group of at least three individuals who will have responsibility for confirming efficacy or safety events by reviewing of data and participants records on a specific case that triggers review by the committee. These individuals will often have expertise in a specific clinical or scientific area that qualifies them for reviewing cases and background data and rendering a decision. The scope of the adjudication committee for the events that they are responsible for and how they will review cases will be describe in the adjudication charter for the events for which they are responsible.

Data Monitoring Committee:

A [data monitoring committee (DMC)] has been appointed for this platform study. The [DMC] is a group of independent scientists who are appointed to monitor the safety and scientific integrity of a human research intervention, and to make recommendations to the sponsor regarding the stopping of an intervention cohort or a study for efficacy, for harms, or for futility. The composition of the committee is dependent upon the scientific skills and knowledge required for monitoring the particular study.

An independent DMC will be commissioned for each ISA.

<End of suggested text>

## Schema

<Start of example text>

Example


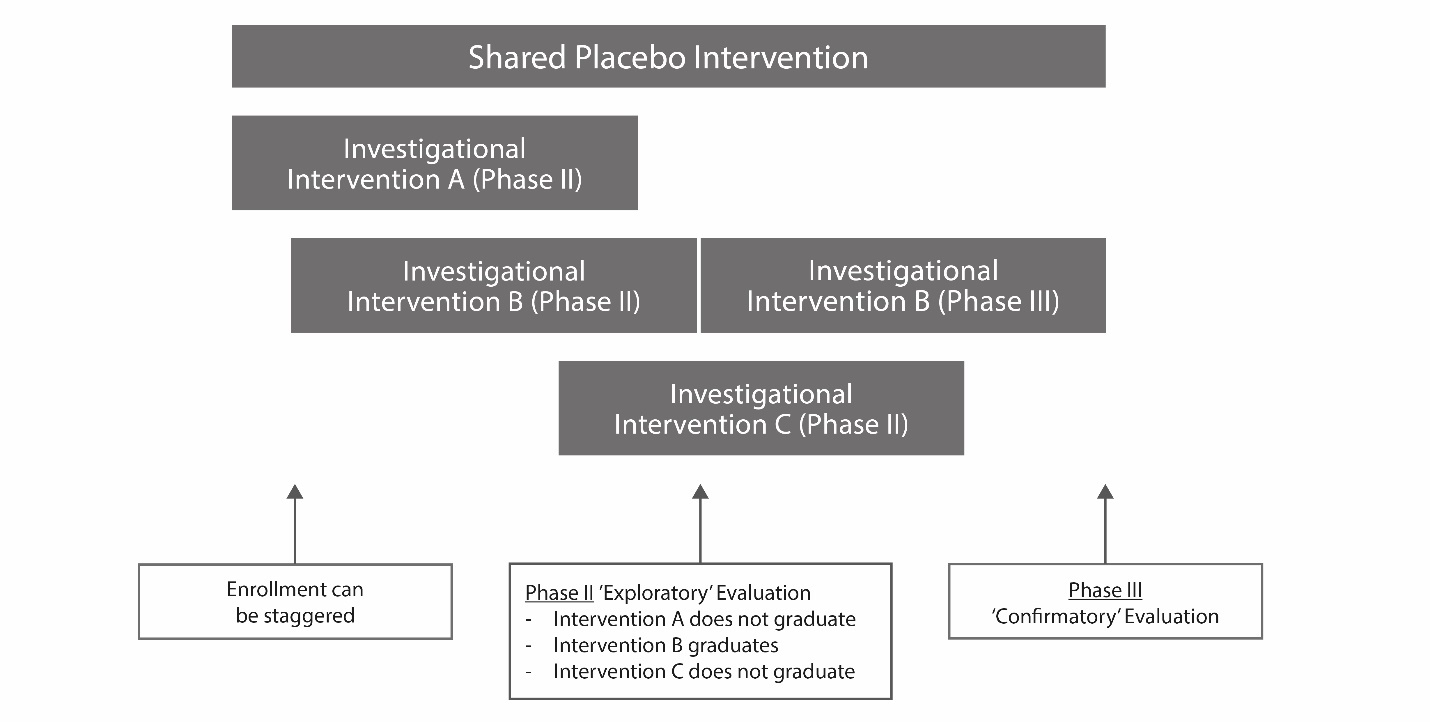


Example


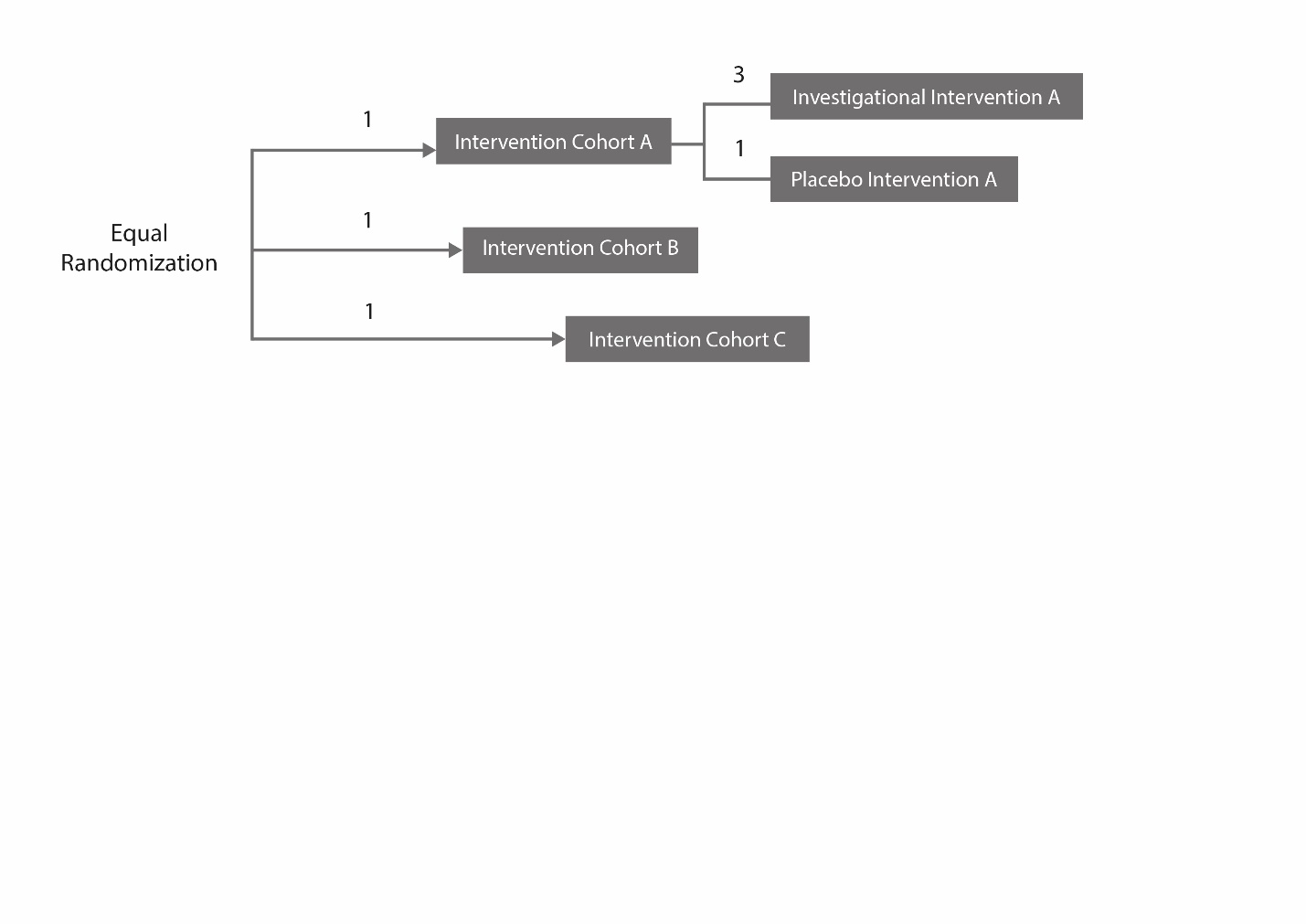


<End of example text>

## Schedule of Activities (SoA)

General information:

- Ensure that only essential data are collected. The schedule of activities (SoA) is the primary location for specifying the timing of assessments at each stage of the study. Do not repeat the SoA schedule in the main text.
- Visit windows may be necessary for the collection of efficacy or safety data. The acceptable windows can be indicated on the SoA by adding ± days or hours/minutes to the visit day or timepoint row.
- If applicable, specify the order of assessments (eg, performing participant-recorded assessments before other assessments to reduce bias or performing electrocardiograms [ECG] or measuring vital signs before blood draws).
- Notes/footnotes (relating to specific procedures) should be minimal, brief, and include key information. If additional details are needed, the notes should refer to the section in the protocol main text where details are provided. Note that Day 0 should not be used as a timepoint.
- Combine assessments on consecutive weeks if they are identical and consider separate tables for separate periods of the study (eg, screening, intervention days, and follow-up). For a multiple-part study, one SoA table for each part of the study is recommended.
- The informed consent process may vary across different types of platform studies. Is it acknowledged that informed consent must be signed before the first study-related activity, which would typically be for the master protocol (eg, master protocol informed consent form [ICF]). After consent is signed for the master protocol, the informed consent process for the intervention cohort(s) may depend on the number of intervention cohort(s) in a platform study. If only a single intervention cohort is open in a platform study, the master protocol and ISA ICFs may be signed at the same time. If multiple intervention cohorts are open in a platform study and participants are to be allocated or randomized among intervention cohorts after screening for the master protocol, the ICFs may be signed sequentially, eg, the master protocol ICF would be signed to permit screening procedures for allocation/randomization to an intervention cohort, followed by the applicable intervention-specific ICF. If there are specific screening or eligibility criteria that determine the intervention cohort that participants will be allocated or randomized to, these screening/eligibility criteria should be in the master protocol and the master ICF. Intervention-specific screening/eligibility criteria should only be in the ISA protocol and the ISA ICF.
- Any intervention-specific assessments will be described in the appropriate ISA. Minimum criteria or assessments are provided in the SoA master.
- An example of a SoA table is included. Modify as required.

<Start of example>

| Procedure | Screening  (up to X days before Day 1) | Baseline | Intervention Period [Visits, etc.] | E/D | Follow-up (Visit after last dose) | Notes  E/D = Early Discontinuation |
| --- | --- | --- | --- | --- | --- | --- |
|  |  | Day1 |  |  |  |  |
| Informed consent | X |  |  |  |  |  |
| Inclusion and exclusion criteria | X |  |  |  |  |  |
| Demography | X |  |  |  |  |  |
| Full physical examination including height and weight | X |  |  |  |  |  |
| Medical history (includes substance usage [and family history of premature CV disease]) | X |  |  |  |  | Substances: [drugs, alcohol, tobacco, and caffeine] |
| Past and current medical conditions | X |  |  |  |  |  |
| [Highly sensitive serum OR urine] pregnancy test (WOCBP only) | X | X |  | X | X | [refer to section 8.2.5 – pregnancy testing for instruction on timepoints] |
| [HIV, Hepatitis B, and C screening] | X |  |  |  |  |  |
| Laboratory tests (include liver chemistries) | X |  | List common tests eg, Liver function tests on Visit 3, 5, 8, 11  + Follow applicable ISA |  |  |  |
| 12-lead ECG | X |  |  |  |  |  |
| Vital signs | X |  | Daily until discharge |  |  |  |
| [Randomization] if applicable |  | X |  |  |  |  |
| Genetic sample |  |  |  |  |  | [Pre-dose/baseline], ICF for genetic sampling should be added per sponsor process (e.g., part of ICF or separate ICF). |
| Study intervention |  | X | Follow applicable ISA |  |  |  |
| AE review |  | X | 🡨=============================🡪 |  |  |  |
| [Solicited Administration site events] if applicable |  | X | 🡨=============================🡪 | X | X | [Pain, redness or swelling]  Consider separate tables for days with multiple assessments |
| [Unsolicited AEs] if applicable |  | X | 🡨=============================🡪 | X | X | See Appendix 3 for definitions  Consider separate tables for days with multiple assessments |
| SAE review |  | X | 🡨=============================🡪 | X | X |  |
| Concomitant medication review |  | X | 🡨=============================🡪 | X | X |  |
| [Study specific assessments (e.g., PK, efficacy)] |  | X | Follow applicable ISA |  |  | Consider separate tables for days with multiple assessments |

# Introduction

- Overall, this section should be short (recommend 2 to 3 pages) and may be started with an overview description of the study population.
- Any intervention specific information should be included in the applicable ISAs.
- Consider that the entire protocol will be subject to public disclosure and be succinct.
- As much as possible, reference the investigator’s brochure (IB), investigational directions for use (IDFU), package insert, and other relevant documents; do not duplicate information available elsewhere.
- The results of pre-clinical studies with potential clinical significance and of clinical trials or studies relevant to the proposed research should be summarized.
- Convincing arguments should be given that there is not sufficient knowledge available to explain the problem and for the need to test what is known.
- This section should also explain why the research needs to be conducted with the selected population. This is particularly important where minors or incapacitated adults or other vulnerable subjects are to be included as participants.
- When writing a master protocol for a specific condition, disease, or indication, include a summary of the specifications of the condition, disease, or indication to clearly indicate the eligible participant population. This could include, but is not limited to participant age (eg, pediatric, adult), gender, clinical and/or laboratory parameters, prior or concomitant medications, morbidity and comorbidities, and unmet medical need.

*<Start of common text>*

A platform study is defined by the master protocol, which describes the overall clinical study design applicable to all related interventions.  The intervention specific appendix (ISA) is the appendix to the related master protocol which describes the specific features of the intervention and treatment of participants randomized to that intervention. The master protocol together with an ISA has all the information needed to conduct an intervention cohort study.

*<End of common text>*

<Start of suggested text>

The following terms are used throughout the master protocol and ISA and are defined below:

- Sponsor is the organization with overall responsibility for the platform study.
- Intervention owner is the organization that provides the investigational treatment and possibly the matching control for an interventional cohort
- A platform study is a study with multiple targeted therapies investigated in a single disease in a perpetual manner, with therapies allowed to enter or leave the platform on the basis of a decision algorithm (Woodcock and Lavange, 2017). A platform study will typically consist of a master protocol and 1 or more ISAs.
- The master protocol is the document which describes the overall clinical study design applicable to all related interventions, such as the clinical study rationale, objectives, endpoints, benefit-risk assessment, shared procedures regarding safety monitoring and reporting, and a common screening platform dictating participant eligibility and/or treatment allocation (CTFG, 2019).
- The ISA is the appendix to the related master protocol which describes the specific features of the intervention and treatment of participants assigned to that intervention or the control group to which it is compared (IMI call). Each intervention will have a separate ISA. Together, a master protocol and an ISA define all the elements needed to conduct a study.
- Intervention cohort refers to the group of participants who receive a specific investigational intervention or an intervention-specific comparator (ie, placebo and/or an active comparator) and in whom that intervention is evaluated. In the event of a shared comparator group used across all intervention cohorts described in the master protocol, the intervention cohort refers to the group of participants who receive the investigational intervention (EU-PEARL). The intervention cohort is described in the ISA.
- The investigational treatment arm refers to the group of participants in an intervention cohort who receive the investigational intervention of interest being evaluated in the ISA (EU-PEARL).

<End of suggested text>

**Master protocol and ISA relationship**

Describe very briefly what is mandated by the Master Protocol and what can be varied in the ISA. Describe how the control arm(s) will be organized:

<Start of suggested text>

This Master Protocol defines the common study inclusion/exclusion criteria, common visit schedule and data collection scheme, randomization, follow-up time, primary endpoint and key secondary endpoints, and the recommended sample size, primary analysis, and success and futility criteria.

An ISA may flexibly define additional inclusion/exclusion criteria (safety only), additional visits and data assessments, additional endpoints including biomarkers, an alternative analysis and success or futility thresholds, and a different sample size.

Control Arm(s): Apart from a placebo arm, other types of control arm are possible, i.e. one in which the participants are on a standard (already commercialized) treatment.

or

This study will not have control arms. An intervention will be compared to pre-defined success threshold determined from historical response rates or intervention specific target product profiles.

<End of suggested text>

<Start of example text>

The prevalence of obesity is increasing worldwide. The World Health Organization (WHO) estimated that worldwide obesity has nearly tripled since 1975, affecting more than 650 million adults (WHO 2017). In the United States (US) in 2015-2016, 39.8% of adults and 18.5% of children and adolescents were obese, representing a significantly increasing trend compared to the prevalence of 30.5% and 13.9%, respectively, reported in the 1999-2000 period (Hales 2017). The US Centers for Disease Control and Prevention predict that obesity-related deaths could soon overtake smoking-related illnesses as the leading cause of mortality in the US.

<End of example text>

## Study Rationale

- Present a 2- to 3-sentence, coherent, scientific description of the rationale for the study with respect to the purpose of the study. The rationale for the study design appears in Section 4.2.
- Include a brief description of the reasons for doing the study (the aim of the study) and for doing it at this time. For example, include any key issues for the platform study that are being addressed.
- This section should be aligned with the overall disease area.
- This rationale should be based on the results of previous studies (if relevant) and the characteristics of the disease entity and should be of scientific merit.
- Provide the rationale for conducting a platform study instead of an independent study for each intervention.

<Start of example text>

This adaptive design platform study will evaluate the efficacy and safety of several different potential treatments which are being developed for [disease/condition].

The platform study design enables multiple study interventions to be evaluated in a clinical study in a simultaneous manner. Platform study designs have specific operating characteristics which need to be carefully considered and balanced against the complexity which is introduced. For this study, the design is anticipated to provide a more efficient means of evaluating novel therapies in [disease/condition].

Using common design elements in this master protocol confers the following advantages:

XXXX

XXXX

In summary, the use of a platform study design will potentially allow for: 1) utilizing a shared control arm for multiple ISAs reducing the number of participants that need to be evaluated to make decisions on the effectiveness of interventions; 2) consistency in data collection and a potential reduction of variability by using a common infrastructure and participant pool; and 3)  lower costs conduct by using the same sites, vendors, and governance, and by sharing the control arm, thus containing the number of participants.

<End of example text>

## Background

To avoid duplication of information and unnecessary protocol amendments, any background on specific intervention-related background information should be described in the applicable ISA.

Describe the standard of care at present in the disease area being evaluated in the platform study.

This section will describe current treatments for [disease/condition], issues and/or limitations with current therapies, and the rationale for developing new therapeutics.

## Benefit/Risk Assessment

- Provide a brief assessment of the benefits and risks of study participation.
- Consider the known and expected benefits and potential risks of the study intervention(s), any significant risks associated with study procedures (biopsies, etc) or design (placebo arm, etc), and any measures to control the risks. Cross reference Section 4 Study Design for details of study procedures, dose, and study design justification.
- The benefit/risk assessment may include a description of the types of events anticipated in the specific study population (eg, hypoglycemic events are anticipated in a Type 1 diabetes participant, and arrhythmias are anticipated in a participant with Class III/IV heart failure).
- Outcomes of discussions with regulatory authorities as related to benefit/risk and reporting may be summarized here if they provide useful insights for the investigator.
- Focus on the benefit/risk in general for the interventions being studied in the platform study relative to placebo/standard of care at the time of study start. If the standard of care changes during the course of the platform study, amend section to reflect any changes to benefit/risk relative to the standard of care at that point in time.
- Any benefit/risk relative to specific interventions should be presented in the applicable ISA.
- Add the benefit/risk from the point of view of the participant (e.g. fewer participants will be treated with placebo).
- Add standard of care information and the benefit on improving on the standard of care.

<Start of suggested text>

For each study intervention, refer to the ISA for details on the source of information for the benefit/risk assessment for the intervention being evaluated.

<End of suggested text>

### Risk Assessment

**Risk Summary and Mitigation Strategy**

For additional guidance and definition refer to: [https://www.ncbi.nlm.nih.gov/pmc/articles/PMC8498219/](https://www.transceleratebiopharmainc.com/assets/clinical-content-reuse-solutions/?domain=urldefense.com) and Guideline for good clinical practice E6(R2) EMA/CHMP/ICH/135/1995 Page 31/68 5.0.3.

[Study-specific Discussion of Procedure Risks and Mitigations]

Trial Procedures – Consider risks associated with the design (for example, placebo arm) and procedures specific to this trial (for example, biopsies), and any measures to control the risks. Provide a brief description of strategies to mitigate identified risks or provide a cross-reference to the relevant protocol section. This is not intended to be an exhaustive list of all possible risks associated with trial procedures but should focus on the unique risks inherent in the design or less common or high-risk procedures. As above, provide a brief description of strategies to mitigate identified risks or provide a cross-reference to the relevant protocol section.

Other – Consider risks associated with other items (for example, comparators, challenge agents, imaging agents, medical devices). Insert a line for each, as needed.

Table format given in example is recommended to address all requirements of EU reg Annex 1, Section D, 17d are met.

Should only include risks related to study conduct and those applicable to all interventions in the platform study.

| **Potential Risk of Clinical Significance**  Briefly summarize only the relevant key risks for THIS study. | **Summary of Data/Rationale for Risk** | **Mitigation Strategy**  For applicable cells in this column, provide a brief description of strategies to mitigate identified risks or provide a cross reference to the relevant protocol section (eg, inclusion/exclusion criteria, participant monitoring, withdrawal criteria, dose selection, comparison to nonclinical no effect levels, duration of dosing, etc). |
| --- | --- | --- |
|  | | |
| Nonclinical risks.  Clinical risks –.  . |  |  |
| **Study Procedures** | | |
| Consider risks associated with the study design and procedures. |  |  |
| **Other** | | |
| Consider risks associated with comparators, challenge agents, imaging agents, , etc. |  |  |

### Benefit Assessment

The benefit assessment should be written relative to what the interventions may offer in general compared to the standard care for the disease being studied in the platform study. All other intervention-specific benefits should be presented in the applicable ISA.

The benefit assessment should be written from the perspective of an individual participant.

### Overall Benefit Risk Conclusion

Provide a succinct concluding statement on the perceived balance between risks that have been identified from cumulative safety data, protocol procedures and anticipated efficacy/benefits within the context of the proposed study. Risks need to be weighed against the benefits for the **individual participant** independent of the intervention(s) received. Intervention-specific content on benefit/risk will be presented in the applicable ISA.

<Start of example text>

Considering the measures taken to minimize risk to participants participating in this study, the potential risks identified in association with conduct of platform study are justified by the anticipated benefits that may be afforded to participants with [indication].

<End of example text>

# Objectives, Endpoints, and Estimands

This section will only contain information that is pertinent to all intervention cohorts. Any information that is specific to an intervention should be described in the applicable ISA.

The master protocol should have an overarching scientific hypothesis and the primary objective should not be changed during the conduct of the trial.

The protocol is the primary source of information for protocol endpoint registration on public registers (eg, ClinicalTrials.gov).

*Objectives*:

- Objectives and endpoints for specific therapeutic areas may be accessed in the therapeutic area libraries. List each scientific objective of the study, clearly and concisely, differentiating between primary, secondary, and tertiary (or other/exploratory) objectives. The objectives should present the questions that the study is designed to answer (which can include predefined safety parameters). Secondary objectives should not merely reiterate the secondary endpoints of the study.
- The objectives should be stated with sufficient specificity that the reader can easily understand the intended context (eg, Superiority of [intervention X] vs control when the intervention is taken as directed). Avoid objectives that use vague terms such as assess or evaluate. Objective text should link to the statistical output (eg, compare, estimate). Consultation or review by a statistician is recommended.

*Endpoints:*

- Ensure that there is an endpoint with associated timepoint or timeframe for each study objective including exploratory objectives, if applicable, and that there are no endpoints without a corresponding objective.
- Be specific and selective when choosing and describing primary and secondary endpoints because results for all primary and secondary endpoints are required to be listed on the ClinicalTrials.gov website and other public registers.
- In a study designed to establish efficacy, a primary endpoint should measure a clinically meaningful therapeutic effect or be a surrogate or other endpoint with a demonstrated ability to predict clinical benefit.
- Avoid vague descriptions of primary and secondary endpoints that encapsulate a large number of measures and timepoints, such as “safety and tolerability as determined by AE reporting, laboratory values, vital signs, and ECGs.” In a typical clinical study this could equate to hundreds of endpoints, all of which must be registered on ClinicalTrials.gov. and other public registers. Consider including such general objectives and endpoints in the Tertiary/Exploratory/Other section, which will not be registered on public registers.
- If there is a safety measure of special relevance, this can be included as a specific primary or secondary endpoint (eg, a specific lab measurement or an adverse event of special interest).
- It is recommended not to include study procedures as an endpoint; only the data resulting from the procedure should be an endpoint. For example, abnormal physical examination should not be designated as an endpoint unless it is clear where this endpoint is captured and how it will be summarized in the clinical study report. Details of procedures should be placed in Section 8 Study Assessments and Procedures. Consider whether the desired endpoints will be achievable in case of unexpected findings, technical/equipment issues, or personnel failure.
- If a clinical outcome assessment (COA) is included in the platform study, mention the concept being measured (eg, fatigue) as well as the instrument (eg, fatigue as measured by the fatigue scale in the Functional Assessment of Cancer Therapy-Anemia [FACT-An]). Avoid the term quality of life and use a more specific term such as physical functioning or vitality.
- If additional tertiary/exploratory/other endpoints (eg, pharmacodynamic endpoints) may be explored, consider addition of a general statement giving an indication of the types of endpoints that might be explored. For example, in cardiovascular studies, a statement such as “Additional atherosclerotic biomarkers may be explored” may be appropriate. The study procedures section of the protocol should clarify, to the extent possible, how and when the additional endpoints will be selected, the types of endpoints that may be assayed (eg, protein, messenger ribonucleic acid), whether existing or additional samples would be used for these assays, and how any changes to exploratory endpoints will be documented.
- Identify surrogate markers if used as study endpoints.

*Estimands:*

- It is recommended that objectives and endpoints be presented together in a table (see example) to ensure all endpoints are aligned with an objective. Further, it is recommended to include the definition(s) of the estimands below the table.
- Estimands include 5 attributes: the treatment condition of interest, the population, variable (or endpoint), details of how to account for intercurrent events (ICEs), and the population-level summary for the variable.
- The estimands should be clear and include sufficient detail on each attribute.
- Estimands are mandatory for confirmatory studies, but it is also strongly recommended to include estimands in other types of studies.
- Define one or more estimands for the primary and key secondary objectives. An estimand description is not required for tertiary/exploratory objectives.
- Include the clinical question(s) of interest that drives the estimand(s) and provide a rationale for the chosen estimand(s), eg, per regulatory guidance or per clinical justification such as estimating the treatment effect without the confounding effect of rescue intervention or the treatment effect including the effect of rescue medication to reflect clinical practice. Make sure to address all ICEs and all other attributes of the estimand and justify accordingly.
- If including more than one estimand for the primary objective, one of them should be named primary.
- If for a particular study different primary estimands/endpoints are required for different regulatory authorities, it should be specified which one is considered primary for each regulatory authority.

Indicate not applicable or remove the section if no estimand is defined for the study.

| Objectives | Endpoints |
| --- | --- |
| Primary |  |
|  |  |
| Secondary |  |
|  |  |
| [Tertiary/Exploratory/Other] |  |
|  |  |

**Primary estimand/coprimary estimands**

<Start of example text>

The primary clinical question of interest is: What is the intervention difference in [health measurement/outcome] after [duration] of intervention in participants with [condition/disease] regardless of intervention discontinuation for any reason and regardless of initiation of rescue intervention or change in background intervention (dose and product)?

The estimand is described by the following attributes:

Treatment: the treatment of interest (individual intervention or in combination) and the alternative treatment condition, with or without [rescue medication or change in background medication].

Population: participants with [condition/disease] as described by the inclusion and exclusion criteria.

Endpoint: variable to be obtained for each participant to address clinical question. For example, change from baseline to [timepoint] in [health measurement/outcome]

Intercurrent Events (ICE)

The list of ICEs and their management strategies such as treatment policy, hypothetical or while on treatment, need to be defined depending on disease and clinical research questions.

Examples of ICEs:

- Participants withdraw from study before the completion of a specified follow-up period for non-treatment related reason
- Participants withdraw from study before the completion of a specified follow-up period for treatment related reason
- Initiation of rescue medication
- Non-compliance to the background intervention
- Discontinuation of background treatment.
- Change in dose regimen of background treatment
- Study treatment discontinuation (e.g. discontinuation by the investigator if the investigator judges that treatment is no longer appropriate, if the participant’s clinical condition is worsening, or for AE)
- Initiation of medications not allowed by protocol.
- Study treatment non-compliance
- Deaths
- Symptomatic COVID-19 infection
- Death due to COVID-19

Population-level summary or the variable (endpoint) to provide a basis for comparison between two groups. For example, difference in mean changes between intervention conditions

Rationale for estimand: [rationale].

**Secondary estimand(s)**

The clinical question of interest is for the secondary objective regarding [target of intervention]: What is the intervention difference in the percentage of participants achieving [health measurement/outcome] after [duration] of intervention in participants with [condition/disease] regardless of intervention discontinuation for any reason and regardless of initiation of any interventions affecting [outcome], eg, [medication and/or surgery]?

The estimand is described by the following attributes:

Population: participants with [condition/disease]. Further details can be found in Section 5.

Endpoint: achievement of [health measurement/outcome] at [timepoint]

Treatment condition: the randomized intervention with or without any other [interventions] (treatment policy strategy). Further details on study interventions and concomitant, including rescue, interventions can be found in Section 6.

The 2 intercurrent events intervention discontinuation for any reason and any interventions affecting [X] are both addressed by the treatment condition of interest attribute. There are no remaining intercurrent events.

Population-level summary: difference in percentage of participants achieving [health measurement/outcome] at [timepoint] between intervention conditions

Rationale for estimand: [rationale]

<End of example text>

# Study Design

## Overall Design

- Do not include study schema.
- Do not include the SoA here.
- Use bullets rather than lengthy text, if possible.
- Include a brief summary of the platform study design (eg, open entry) and control method (eg, , historical, common control or none [uncontrolled]), multicenter. Include the kind of control group to be used, if any.
- Include a high-level description of the study population (eg, participants with F2-F3 fibrosis and diagnosis of non-alcoholic steatohepatitis).
- Level and method of blinding if it is the same across all interventions being evaluated (eg, open-label, single-blind, double-blind, matching placebo, double-dummy), and the methods to be used to minimize bias on the part of participants, investigators, and analysts.
- Study intervention assignment method and how it may be adapted when new interventions are added to the platform study (eg, response adaptive, randomization, stratification, both). Do NOT state block size. If assignment to intervention requires response adaptive randomization, describe at a high level how randomization will be adapted when interim decisions are made and how randomization will be done within cohorts and across the entire study. Do not put sample size justification here. This is covered in Section 9 Statistical Considerations.
- Refer to any use of an independent data monitoring committee, adaptations/interim analysis committee, or similar review group and cross-reference Appendix 10.1.5 Committees Structure.
- Total duration of study participation for each participant with sequence and duration of study periods (eg, screening, run-in, fixed dose/titration, follow up/washout periods) if it consistent across all interventions in the platform study. Otherwise, content should be described in the ISA.
- Include any plans to obtain long-term follow-up information regarding the participant’s safety or survival status as noted in the ICF and assent form if the plan is consistent across all interventions in the platform study. Otherwise, details on this should appear in the ISA.
- See therapeutic area libraries for additional guidance for studies in specific therapeutic areas.
- A protocol deviation is related to a data point or process identified in the protocol or documents referenced in the protocol (eg, laboratory manual). When designing the study, limit items that may generate deviations whenever possible. Reduce the number of reference documents to those essential for the conduct of the study.
- When writing a master protocol for a specific condition, disease, or indication, include specifications of the condition, disease, or indication to clearly indicate the eligible participant population. This could include, but is not limited to participant age (eg, pediatric, adult), gender, clinical and/or laboratory parameters, prior or concomitant medications, morbidity and comorbidities, and unmet medical need. Any details required to clearly define the participant population should be included here and align with the eligibility criteria in Section 5.

<Start of suggested text>

This is a [Phase 2a, Phase 2b, Phase 2a/b, Phase 3, Phase 2/3] platform study to investigate the [purpose (eg, efficacy and safety)] of multiple study interventions simultaneously or sequentially in cohorts of [provide high-level description of the study population].

This master protocol describes the framework for the general study population and the common study elements of the platform study. The accompanying ISA(s) will present the intervention-specific information, including intervention-specific objectives, the justification for the study intervention dose(s), the number of participants to be assigned to an intervention cohort, any additional inclusion and exclusion criteria, and any additional study elements for the study intervention(s) as applicable.

The study will be conducted in several phases: [provide high-level description of the study phases]. The duration of individual participation will be approximately [specify number of weeks or months].

[Randomization across ISAs will be performed if at site level there is overlapping recruitment for at least 2 ISAs, the ISAs are focused on recruiting the same population (i.e., no conflicting in/exclusion criteria), and the interventions have a similar benefit-risk ratio.]

[Participants who do not meet all screening criteria for a particular ISA and agree to participate in another ISA may need to repeat some of the screening activities for the Master protocol if they are outside the screening window as detailed in the applicable ISA.]

[At screening, each participant will receive a core participant number (x number of digits) that will remain the same during participation in the platform. This number will be prefixed with x number of digits: the first x numbers will indicate the ISA in which the participant is currently enrolled and the next x numbers will be incremented by +1 with each additional screening (and re-screening, if applicable) at the Platform and ISA level.(e.g., 2346 (Core study [master protocol])-0001 (1st participant enrolled in the core study), for a participant # of 2346-0001. ISA numbers would then be added)

The numbering scheme is intended to easily track participants through the Platform via the core participant number while knowing which ISA they are participating as well as their (re)screening history.]

[In general, participants who have enrolled in a given ISA will have the option to re-enroll in a different or subsequent ISA (if available) only after they have completed participation to the first ISA, and if they meet the new intervention-specific eligibility criteria.]

Throughout the study [efficacy, safety, pharmacokinetics, pharmacodynamics, pharmacogenomics, biomarkers, immunogenicity, health economics] will be assessed at the timepoints indicated in the applicable ISA Schedule of Activities.

A database lock is planned to occur after the completion of each intervention cohort.

[Additional database locks may be added for an individual intervention cohort if appropriate and will be specified in the ISA].

[A Data Monitoring Committee will be commissioned to review safety data periodically.]

[Within each intervention cohort, interim analyses maybe performed, if applicable, at pre-specified time points. An Interim Analysis/Adaptations Committee will review the interim efficacy analysis results.]

[An intervention arm within a given ISA or the complete ISA may be terminated if it is determined to be efficacious or futile based on interim analysis results or due to safety concerns].

<End of suggested text>

## Scientific Rationale for Study Design

- Provide scientific rationale for any features of the platform study design. Include any key ethical issues.
- Provide justification for why conducting a platform study is appropriate in the specific disease area. What are the advantages and efficiencies gained with such a design compared to a traditional study design evaluating a single intervention? Explain the potential for rationale effective interventions and how many interventions could be evaluated as part of the platform study. Is there a maximum number of interventions (overall/at any point of time) that can be evaluated?
- Explain what the current standard care/control arm is at present and under what conditions the control arm could change during the study.
- Describe why the primary endpoint is clinically relevant and how it provides a reliable and valid measurement of the intended intervention effect.
- Discuss how the primary endpoint measures direct benefit in how the participant feels, functions, or survives and what would constitute a clinically meaningful effect.
- If a measure of direct benefit is not proposed, describe how a proposed surrogate endpoint substitutes for how a participant feels, functions, or survives, based on epidemiologic, therapeutic, pathophysiologic, or other evidence to predict benefit.
- If applicable, provide a scientific rationale that including a vulnerable study population (eg, pediatric participants or participants requiring emergency care) has the potential to produce a clinically relevant benefit for all interventions.
- Provide justification for the sex and age allocation of participants and if a specific sex or age group is excluded from or underrepresented in the study, an explanation of the reasons and justification for these exclusion criteria.
- Describe the intermediate endpoints by which interim decision will be performed and how these intermediate endpoints are related to the key endpoint(s).
- Provide rationale for other design elements (e.g., pharmacogenomics, biomarker collection/storage).

<Start of suggested text>

All participants meeting the eligibility criteria for the master protocol can be screened for eligibility for one or more ISAs. Additional eligibility criteria will be specified in the applicable ISA.

The common outcomes that will be assessed for all ISAs are based on [clinical assessments] that have been used for decision-making in the development of treatments for the disease.

Any additional intervention-specific scientific rationale will be described in the applicable ISA.

<End of suggested text>

### Participant Input into Design

Describe any participant involvement in the design of the clinical study and any participant suggestions implemented.

This subsection should describe any additional patient and/or their representatives input on study design and operations, such as the schedule of activities, inclusion and exclusion criteria, the types of procedures, number and flexibility of the visits, choice of endpoints, PROs, communication during and after of the study, study results (including informing the participants of (interim) study results) while maintaining the overall scientific integrity and regulatory compliance of the study.

This section should describe the methodology used to collect patient inputs.

If applicable, include a justification of why there is no engagement of patients required.

For additional guidance and definition refer to: <https://eupati.eu/resources/patient-engagement-roadmap> or https://patientfocusedmedicine.org/pem-suite.

## Justification for Dose

Justification for the doses of each intervention will be described in the applicable ISA.

## End of Study Definition

Distinguish between the end of the study (EU definition) and study completion (US CT Registry definition: final date on which data were or are expected to be collected) if they are not the same.

Distinguish between participant completion, intervention cohort completion, and platform study completion.

<Start of suggested text>

**Participant Completion**

A participant will be considered to have completed an intervention cohort if the participant has completed assessments through [Week XX of the blinded phase] [the final follow-up visit] for that intervention cohort or discontinued earlier.

**Intervention Cohort Completion**

The end of the intervention cohort is defined as the date of the last visit for the last participant in that intervention cohort.

**Platform Study Completion**

The end of the platform study is defined as the date of the last visit of the last participant in the last ISA.

The platform study stopping determination will be made by the Steering committee based upon a determination as to any further interventions are planned to be entered into the study and/or whether there is scientific validity to continue the platform study.

<End of suggested text>

# Study Population

Criteria should be numbered according to company process.

<Start of common text>

All participants must satisfy all the inclusion and exclusion criteria in this Master protocol and an ISA, if applicable, to enroll in the platform study. Any additional intervention-specific criteria will be described in the applicable ISA.

Prospective approval of protocol deviations to recruitment and enrollment criteria, also known as protocol waivers or exemptions, is not permitted.

<End of common text>

## Inclusion Criteria

General Points:

- List the criteria necessary for participation in the study. Ensure that each criterion can be easily assessed on the basis of measurable data and answered with yes/no responses.
- When choosing inclusion criteria, consider that study participants should be representative of the participant population to which the results will be generalized.
- The choice of the study population in a Phase 2 or Phase 3 clinical study should reflect the intended use of the intervention. This is particularly relevant for planning multiregional studies and for the range of subgroups that may be relevant to evaluate.
- If measures to enrich the study population for prespecified subgroups of interest are used, they should be described here and further details of how this will be handled in the analysis should be presented in Section 9: Statistical Methodology, if it is applicable to all intervention cohorts otherwise, such details will be described in the applicable ISA.
- In general, laboratory results required for eligibility should be listed as inclusion criteria rather than exclusion criteria.
- The use of double negatives should be avoided (eg, no indication of prior noncompliance with the intervention regimen).

Participants are eligible to be included in the study only if all of the following criteria apply:

| Age |
| --- |
| <Start of suggested text>   1. Participant must be [X] [or the legal age of consent in the jurisdiction in which the study is taking place] to [Y] years of age inclusive, at the time of signing the informed consent. |
| Type of Participant and Disease Characteristics |
| State whether rescreening will be allowed and the circumstances under which rescreening can occur (eg, laboratory value range) and cross-reference Section 5.4 if appropriate:  Participants who are [insert disease specific criteria]  <End of suggested text>  For studies in participants with the disease of interest, provide disease‑related considerations: standard, accepted diagnostic criteria (consider supplying laboratory reference ranges or clinical diagnostic criteria in an appendix). Include duration/severity of disease or disorder if appropriate.  When appropriate, specify a realistic and pragmatic inclusion range for each test or marker of interest. Take into consideration any known assay variance or error rate as well as biological variation to avoid creating protocol violation issues.  State whether rescreening will be allowed and the circumstances under which rescreening can occur (eg, laboratory value range) and cross-reference Section 5.4, if appropriate.  Check whether additional information associated with the disease area can be found in the therapeutic area libraries. |
| Weight |
| Consider whether any restriction on weight or BMI is needed for this study intervention/stage of development and delete if not required.  <Start of suggested text>   1. Body weight within [insert range including units] and body mass index (BMI) within the range [X – X] kg/m^2^ (inclusive)   <End of suggested text> |
| Sex and Contraceptive/Barrier Requirements |
| 1. [male and/or female]   <Start of common text>  If there are no contraceptive requirements in the study, remove the statement “Contraceptive use by men and women …”. Modify as appropriate based upon inclusion of men and women.  Contraceptive use by [men and women] should be consistent with local regulations regarding the methods of contraception for those participating in clinical studies.   1. Male participants:   See participant libraries for common text to include here.   1. Female participants:   See participant libraries for common text to include here.  Check local requirements. For reference see CTCG guidance on contraception and pregnancy testing in clinical trials: https://www.hma.eu/fileadmin/dateien/Human_Medicines/01- About_HMA/Working_Groups/CTFG/2020_09_HMA_CTFG_Contraception_guidance_Version_1.1_updated.  <End of common text> |
| Informed Consent  If randomization across ISAs is not applicable, the platform study will utilize a 1-step consent process, i.e., a modular approach obtaining the master protocol consent and the ISA consent at the same time. Once a participant has signed both Informed Consent Forms (ICF) (i.e., Master and ISA ICF), the screening activities can commence.  In case of randomization across ISAs that are conducted in an overlapping way, the platform study will utilize a 2-step consent process, i.e., the master protocol consent and the ISA consent will be obtained separately. Upon having signed the Master ICF, participants will be assigned to an ISA (based on data registered into the IWRS) for which they will sign the corresponding ISA ICF and will subsequently undergo screening activities at that same time. This process will avoid participants being allocated to ISAs in which they may not qualify.  The modular protocol approach utilizing a Master protocol and an ISA supports the need to have a consistent approach to providing and collecting ICFs. The master protocol ICF details the platform study design and procedures while the ISA ICF will describe the following in lay language:  • Background information on the study intervention(s)  • Intervention-specific assessments and procedures  • Intervention-specific and procedural risk language  The two ICFs will provide the participant with a clear distinction between the common study elements and the intervention-specific study elements to allow for the ICF process to occur in a participant-centric manner and support participant understanding. A participant can withdraw consent at any time.  Any change in the risk- benefit assessment for the patient (example: in case of change in standard of care and the control arm), may result in a protocol amendment, and the patient will be asked to reconsent. |
| <Start of common text>   1. Capable of giving signed informed consent as described in Appendix 1 which includes compliance with the requirements and restrictions listed in the informed consent form (ICF) and in this protocol. Participants must consent at 2 levels, and sign an ICF for both the master protocol and the applicable ISA.   <End of common text> |
| Other Inclusions |
|  |

## Exclusion Criteria

Exclusion criteria: See participant and therapeutic area libraries for suggested text. Numbering will start again for exclusion criteria or be continued from the inclusion criteria dependent on company practice or requirements of technology solutions.

Any exclusion criteria named in the master protocol apply to all ISAs, whereas any exclusion criteria that are specific to an intervention cohort will be described in the applicable ISA. Definition of master protocol exclusion criteria should be carefully evaluated to avoid amendments to address needs for subsequent ISAs.

Participants are excluded from the study if any of the following criteria apply:

| **Medical Conditions** |
| --- |
| 1. [ ] |
| **Prior/Concomitant Therapy** |
| 1. [ ] |
| **Prior/Concurrent Clinical Study Experience** |
| 1. [ ] |
| **Diagnostic Assessments** |
| 1. [ ] |
| **Other Exclusions** |
| 1. [ ] |

## Lifestyle Considerations

- [ ]
- [ ]

If this section is not applicable, include a statement that no restrictions are required. Do not omit section.

If there are difference across interventions, then the details should be described in the applicable ISA.

If applicable, describe any of the lifestyle considerations (diet, smoking habits, alcohol, or recreational drug consumption, etc) that could be of relevance for the study and any restrictions during any of the study periods. For example, include a statement about exposure to sunlight for study interventions with photosensitivity potential.

Level 3 headings may not be applicable for all studies (eg, vaccines).

### Meals and Dietary Restrictions

- Any details on meal and dietary restrictions that can be applied across all interventions should be described here. Intervention-specific details should be described in the applicable ISA.

### Caffeine, Alcohol, and Tobacco

- Any details on caffeine, alcohol, and tobacco that can be applied across all interventions should be described here. Intervention-specific details should be described in the applicable ISA.

<Start of suggested text>

- During each dosing session, participants will abstain from ingesting caffeine- or xanthine-containing products (eg, coffee, tea, cola drinks, and chocolate) for [X hours] before the start of dosing until after collection of the final pharmacokinetic (PK) and/or pharmacodynamic sample.
- During each dosing session, participants will abstain from alcohol for 24 hours before the start of dosing until after collection of the final PK and/or pharmacodynamic sample.
- Participants who use tobacco products will be instructed that use of nicotine-containing products (including nicotine patches) will not be permitted while they are in the clinical unit. [OR] Use of tobacco products will not be allowed from [screening/the start of dosing] until after the final follow-up visit.

<End of suggested text>

### Activity

Any details on activity that can be applied across all interventions should be described here. Intervention-specific details should be described in the applicable ISA.

Study-specific restrictions may apply depending on the nature and frequency of assessments, e.g., activity may be further restricted by ensuring participants remain in bed for 4 to 6 hours after dosing, or for studies with interventions known to cause photosensitivity, activities such as sunbeds may be restricted.

<Start of suggested text>

1. Participants will abstain from strenuous exercise for [X hours] before each blood collection for clinical laboratory tests. Participants may participate in light recreational activities during studies (e.g., watching television, reading).

<End of suggested text>

## Screen Failures

Participants must meet all screening criteria in the master protocol to be screened for an intervention cohort. If a participant meets the criteria of the master protocol but does not meet the screening criteria for an intervention cohort, determine if they are eligible to screen for a different intervention cohort in the same program, depending on the study design and study intervention.

<Start of suggested text>

Participants who meet the entry criteria for inclusion per the master protocol but do not meet the entry criteria for participation in this intervention cohort [may/may not] be rescreened to another intervention cohort.

<End of suggested text>

Participants should be screened for all ISAs that are recruiting participants at the time of their screening.

The protocol must describe how it will be determined which intervention cohort a participant will be randomized to based on their eligibility.

<Start of common text>

A screen failure occurs when participants who consents to participate in the clinical study is not subsequently [randomly assigned to study intervention/entered in the study]. A minimal set of screen failure information is required to ensure transparent reporting of screen failure participants to meet the Consolidated Standards of Reporting Trials (CONSORT) publishing requirements and to respond to queries from regulatory authorities. Minimal information includes demography, screen failure details, eligibility criteria, and any serious adverse event (SAE).

<End of common text>

State whether rescreening is permitted. If rescreening is permitted, state the entry criteria/parameters that can be reassessed for individuals who previously failed screening and the time period for repeating procedures/rescreening. Individual inclusion/exclusion criteria may also state whether a repeat procedure is allowed without being considered a rescreen.

<Start of suggested text>

Individuals who do not meet the criteria for participation in active ISAs (screen failure) [may/may not] be rescreened. [Rescreened participants should be assigned a new participant number for every screening/rescreening event.]

<End of suggested text>

## Criteria for Temporarily Delaying [Enrollment/Randomization/Administration of Study Intervention]

Insert suggested text from participant or therapeutic libraries if relevant to all interventions, otherwise, details should be provided in the applicable ISA.

# Study Intervention(s) and Concomitant Therapy

<Start of common text>

Study intervention is defined as any investigational intervention(s), marketed product(s), or placebo, intended to be administered to a study participant according to the study protocol. Study intervention information will be described in the applicable ISAs. However, if there is a common control arm, details are provided in the applicable sections below.

<End of common text>

## Study Intervention(s) Administered

<Start of sample text>

In this platform study, <placebo/standard of care/active comparator xxx>, will be used as the common control arm for all intervention cohorts. If any investigational treatment evaluated in the platform study demonstrate superiority and is established as the new standard of care, the platform study steering committee will discuss and decide whether or not a new control arm will be established for future intervention cohorts.

The platform study steering committee will discuss and decide whether or not a new intervention will be introduced to the platform study. The steering committee will make this decision based on the criteria for potential new interventions as outlined below.

- - - Sound rationale, including a robust scientific hypothesis and compelling clinical research evidence of activity that strongly identifies a need to assess a research approach in the setting studied.
    - Intervention owners are willing to collaborate and contribute to the study.
    - Successful peer review of the potential new intervention.
    - Introduction of the new intervention must not jeopardize the completion of the ongoing intervention cohorts.

<End of sample text>

Details provided in the applicable ISA.

Add guidance and sample text for studies where a common control agent/formulation (e.g. placebo or active comparator) is used across ISAs

Keep deleted TransClerate Common Protocol template text if control arm is in master. Add Section 6.1 from TransClerate Common Protocol template for study interventions in control arm.

### Medical Devices

- This section is required for medical devices and can be deleted for non-device protocols. A device user manual can be included as an appendix to the protocol.
- Describe any sponsor-provided medical device(s) including any materials that will be in contact with tissues or body fluids. Include details of any medicinal products, human or animal tissues or their derivatives, or other biologically active substances.
- **Consult with Regulatory Affairs of sponsor** if use of a device is required for the study because not all devices are defined as medical devices and different regions have different definitions for a medical device.
- Examples of sponsor medical devices include, but are not limited to, the following: metered dose inhaler, autoinjector, inhalation spacers, measuring cups, measuring spoons, pediatric oral syringes, and dry-powder inhalers.
- For devices, if a control product is used, ensure that description of its use is consistent with the applicable DFU in countries where the study will be conducted.
- Specifically note whether the device is cleared by the FDA cleared or not. If any diagnostic tests have not been approved or cleared for the indications the protocol is designed to investigate, a detailed test protocol (including specimen collection/storage/processing/testing procedures, result interpretation guide, the number and name(s) of US and non-US testing sites) and a summary/report of test validation studies should accompany the protocol submission.
- The detection and documentation procedures described in this protocol apply to all sponsor medical devices provided for use in the study.

<Start of suggested text>

1. The [sponsor] manufactured medical devices (or devices manufactured for [sponsor] by a third party) provided for use in this study are [list here].
2. Other medical devices (not manufactured by or for [sponsor]) provided for use in this study are [list here].
3. Instructions for medical device use are provided [cross reference the location of such information].
4. All device deficiencies (including malfunction, use error and inadequate labelling) shall be documented and reported by the investigator throughout the clinical investigation (see Section 8.3.9) and appropriately managed by the sponsor.

<End of suggested text>

## Preparation, Handling, Storage, and Accountability

Instructions for the preparation of study interventions, , should be provided (e.g., reconstitution, mixing) in the applicable ISA.

Keep if control arm in master.

<Start of common text>

Refer to the applicable ISA for information regarding study intervention preparation, handling, storage, and accountability.

<End of common text>

## Measures to Minimize Bias: Randomization and Blinding

- Describe method of assigning participants to study interventions.
- Briefly describe the randomization procedures (e.g., central randomization procedures), the method used to generate the randomization schedule (e.g., computer generated), the source of the randomization schedule (e.g., sponsor, investigator, or other), and whether or not an Interactive Voice/Web Response System (IVRS/IWRS) will be used. To maintain the integrity of the blind, do NOT include the block size.
- If adaptive randomization to intervention is to be used or if other methods of covariate balancing/minimization are employed, provide a cross link to the methods of analysis in Section 9 Statistical Considerations.
- State any other study-specific rules (e.g., once a randomization number has been assigned, it must not be re-assigned).
- Include details of how and when a participant is allocated a participant number and the participant numbering convention, if relevant, in the Study Reference Manual.
  - Discuss any bias-reducing procedures if randomization is not used.
  - Include the stratification process and stratification variables, if applicable.
- Define enrichment and adaptation strategies. Outline any pre-specified enrichments or adaptations to randomization.
- Intervention eligibility - whether an ISA can limit its accrual to specific subgroups.
- Early stopping – whether an ISA’s enrolment of a particular subgroup can be stopped early for success or futility (adaptive enrichment).
- Re-allocation of study participants – Interim analysis decisions may lead to changes in the randomization ratios across the intervention cohorts of the platform study
- Response adaptive randomization – whether response adaptive randomization will be used within subgroups to preferentially allocate better performing interventions based on a participant’s subgroup.
- Balanced randomization – whether stratification will be used to balance the allocation of sub-groups to the different interventions in the study.

<Start of suggested text>

[Participants will be randomly allocated to the available intervention cohorts to avoid imbalances across intervention cohorts and potential resulting bias.]

[A placebo/standard of care control will be used to establish the frequency and magnitude of changes in clinical endpoints that may occur in the absence of active intervention.] [An active control will be used to determine the sensitivity of the clinical endpoints to change due to intervention in this study.]

[Randomization will be used to minimize bias in the assignment of participants to intervention arms, to increase the likelihood that known and unknown participant attributes (eg, demographic and baseline characteristics) are evenly balanced across intervention arms, and to enhance the validity of statistical comparisons across intervention arms.]

[Blinded intervention will be used to reduce potential bias during data collection and evaluation of clinical endpoints.] [When pooling participants assigned to control arms from multiple ISAs, blinding should be maintained as long as the studies per respective ISAs are still ongoing. To this end, for these ongoing ISAs only the efficacy data from the specified time points together with dummy participant identifiers will be provided.]

<End of suggested text>

<Start of example text>

**Example for Response Adaptive Randomization**

| **Type of Study** | Example text to use |
| --- | --- |
| **Study using IVRS/IWRS** | All participants will be centrally assigned to randomized study intervention using an interactive voice/web response system (IVRS/IWRS). Before the study is initiated, the telephone number and call-in directions for the IVRS and/or the log-in information and directions for the IWRS will be provided to each site.  Study intervention will be dispensed at the study visits as summarized in the applicable ISA specific SoA.  Returned study intervention should not be redispensed to the participants. |
| **Study using precoded randomization provided to site** | On Day [X], participants will be assigned a unique number (randomization number) in ascending numerical order at each study site. The randomization number encodes the participant’s assignment to one of the [X] arms within one of the intervention cohorts of the study, according to the randomization schedule generated prior to the study by the statistics department at [sponsor/designee]. Each participant will be dispensed blinded study intervention, labeled with his/her unique randomization number, throughout the study. |

<End of example text>

- *Masking* or another appropriate synonym may be used in place of *blinding* if considered more appropriate in the context of the study or study population (eg, studies involving visually impaired participants), but maintain consistent use within the protocol.
- If study includes blinding, ensure to include details to whom the intervention is blinded for: *Single-blind* refers to studies in which participants are blinded to study intervention but site personnel (eg, monitors and investigators) and sponsor personnel are not. *Double-blind* refers to studies in which both participants and site personnel are blinded to study intervention.
- If someone involved in conducting the study is not blinded (eg, the site pharmacist or the sponsor’s clinical study material group), describe the methods used to preserve the blinding of the other personnel conducting the study within an intervention cohort and across intervention cohorts when they are participating in more than one.
- Provide a description of the specific blinding procedures, if any, to be used. If blinding will not be used, include a statement to that effect.
- Describe how any blinding will be achieved and any impact on bias/randomization.
- Include the circumstances in which the blind will be broken for an individual or for all participants (eg, for SAEs), the procedures to be implemented to do this, and a description of who has access to participant codes. If the study allows for some investigators to remain unblinded (eg, to allow them to adjust medication), the methods of shielding other investigators should be explained.

<Start of example text>

| **Type of Study** | Example text to use |
| --- | --- |
| **Open-label, no blinding at site level** | This is an open-label study; potential bias will be reduced by the following steps: [central randomization, adjudications]. |
| **Open-label using central randomization via IVRS/IWRS** | This is an open-label study; however, the specific intervention to be taken by a participant will be assigned using an IVRS/IWRS. The site will contact the IVRS/IWRS prior to the start of study intervention administration for each participant. The site will record the intervention assignment on the applicable case report form, if required. Potential bias will be reduced by the following steps: [central randomization, adjudications]. |
| **Blind break (IVRS/IWRS)** | This is a double-blind study in which [participants/care providers/investigators/outcomes assessors, etc] are blinded to study intervention. The IVRS/IWRS will be programmed with blind-breaking instructions. In case of an emergency, the investigator has the sole responsibility for determining if unblinding of a participants’ intervention assignment is warranted. Participant safety must always be the first consideration in making such a determination. If the investigator decides that unblinding is warranted, the investigator should make every effort to contact the sponsor prior to unblinding a participant’s intervention assignment unless this could delay emergency treatment for the participant. If a participant’s intervention assignment is unblinded, the sponsor must be notified within 24 hours of this occurrence. The date and reason that for the unblinding must be recorded. |
| **Open-label using blinded randomization** | This is an open-label study; however, the specific intervention to be taken by a participant will be assigned using randomization envelopes. The site will receive blinded randomization envelopes that will be opened in ascending numerical order immediately prior to the start of study intervention administration for each participant. The site will record the date and time the envelope was opened.  **Note for open label**: This is not an approach to be supported from a statistical perspective. Open-label randomized studies need to use central randomization. If envelopes are preassigned to the site, the randomization must be blocked at the site level, which introduces selection bias risk whether or not the randomization codes are blinded in envelopes. |
| **Blind break (envelopes)** | This is a double-blind study in which [participants/care providers/investigators/outcomes assessors, etc] are blinded to study intervention. A sealed envelope that contains the study intervention assignment for each participant will be provided to the investigator. The sealed envelope will be retained by the investigator (or representative) in a secured area. In case of an emergency, the investigator has the sole responsibility for determining if unblinding of a participant’s intervention assignment is warranted. Participant safety must always be the first consideration in making such a determination. If the investigator decides that unblinding is warranted, the investigator should make every effort to contact the sponsor prior to unblinding a participant’s intervention assignment unless this could delay emergency treatment for the participant. If a participant’s intervention assignment is unblinded, the sponsor must be notified within 24 hours of this occurrence. Once the study is complete, all envelopes (sealed and opened) must be inventoried and returned to the sponsor. |
| **Blinded study with unblinded third party who is dispensing intervention** | Participants will be randomly assigned in a [1:1] ratio to receive study intervention. Investigators will remain blinded to each participant’s assigned study intervention throughout the course of the study. To maintain this blind, an otherwise uninvolved third party will be responsible for the reconstitution and dispensation of all study intervention and will endeavor to ensure that there are no differences in time taken to dispense following randomization.  This third party will instruct the [participant/participant’s parent(s) or legally authorized representative] to avoid discussing the taste, dosing frequency, or packaging of the study intervention with the investigator.  In the event of a quality assurance audit, the auditor(s) will be allowed access to unblinded study intervention records at the site(s) to verify that randomization/dispensing has been conducted accurately. |

<End of example text>

<Start of suggested text>

[Sponsor safety staff may unblind the intervention assignment for any participant with an SAE. If the SAE requires that an expedited regulatory report be sent to one or more regulatory agencies, a copy of the report, identifying the participant’s intervention assignment, may be sent to investigators in accordance with local regulations and/or sponsor policy.]

<End of suggested text>

## Study Intervention Compliance

Procedures to be used for study intervention compliance should be described in the applicable ISA.

<Start of common text>

Refer to the applicable ISA for information regarding study intervention compliance.

<End of common text>

## Dose Modification

Procedures to be used for selecting/modifying each participant's dose of study intervention should be described in the applicable ISA.

<Start of suggested text>

Refer to the applicable ISA for information regarding study intervention dose modification.

<End of suggested text>

### Retreatment Criteria

Should be described in the applicable ISA.

<Start of suggested text>

See section in the applicable ISA.

<End of suggested text>

## Continued Access to Study Intervention after the End of the Study

Include planned extension studies or possibilities for continued access to study intervention, if any, beyond completion of the study. Continued access should be clearly defined – differentiate between study-level and participant-level access.

If there is no intervention following the end of the study, then text should be included to state that this is the case. Describe any additional care that will be provided to participants after they complete or discontinue the study if this differs from what is normally expected for their condition.

Should be included only if it applies to all intervention cohorts, otherwise, this should be described in the applicable ISA as appropriate.

<Start of suggested text>

Refer to the applicable ISA for information regarding continued access to the study intervention after the end of the study.

<End of suggested text>

## Treatment of Overdose

Should be described in the applicable ISA.

<Start of suggested text>

- An overdose (accidental or intentional) with the IMP/AxMP is an event suspected by the Investigator or spontaneously notified by the participant and defined as at least twice the intended dose within the intended therapeutic interval, adjusted according to the tested drug.
- Infusion: increase of at least 30% of the dose to be administered in the specified duration or if the dose is administered in less than half the recommended duration of administration.
- Injectable administration: at least twice the dose during the planned intervals

[Sponsor does not recommend specific treatment for an overdose] OR [The antidote to study intervention is YYY] and may be used in case of an overdose.

<End of suggested text>

<Start of suggested text>

In the event of an overdose, the Investigator should:

- Closely monitor the participant for any AE/SAE and laboratory abnormalities [as medically appropriate and at least until the next scheduled follow-up].
- Evaluate the participant to determine, if possible, whether study intervention should be interrupted or whether the dose should be reduced.
- [Obtain a plasma sample for PK analysis as soon as possible].
- Document appropriately in the CRF.

<End of suggested text>

## Concomitant Therapy

- Describe which interventions or procedures will be allowed before and during the platform study across interventions and any other specific rules and procedures related to permitted or prohibited concomitant therapy across all interventions. Any concomitant relevant to specific interventions should be described in the applicable ISA. If this list is lengthy consider including details in an appendix and cross-referencing here.
- Minimize use of concomitant therapies or other co-interventions that can affect critical outcome measures to reduce potential imbalances in such co-interventions across intervention groups.
- Outline expectations for recording the use of concomitant therapies.
- Mention any nonstudy interventions, such as background therapy or rescue medication, as applicable.
- Include sponsor guidance, if any, on the management of study-specific conditions (eg, hyperkalemia, blood pressure control, edema, glucose control) that may need to be treated during the study.
- Review therapeutic area libraries for additional guidance.
- Consider whether rescue therapy will be allowed and provide details if appropriate.
- Additional intervention-specific concomitant therapy considerations should be included in the ISA(s) as applicable.

<Start of suggested text>

Any [medication or vaccine (including over-the-counter or prescription medicines, recreational drugs, vitamins, and/or herbal supplements) or other specific categories of interest] that the participant is receiving at the time of enrollment or receives during the study must be recorded along with:

- [Reason for use
- Dates of administration including start and end dates
- Dosage information including dose, route, and frequency]

The medical monitor should be contacted if there are any questions regarding concomitant or prior therapy.

Participants must abstain from taking prescription or nonprescription drugs (including vitamins, recreational drugs, and dietary or herbal supplements) within 7 days (or 14 days if the drug is a potential enzyme inducer) or 5 half-lives (whichever is longer) before the start of study intervention until completion of the follow-up visit, unless, in the opinion of the investigator and sponsor, the medication will not interfere with the study.

Other concomitant medications may be considered on a case-by-case basis by the [investigator in consultation with the] medical monitor [if required].

<End of suggested text>

### Rescue Medicine

If rescue therapy is permitted, consider using the suggested text provided consider using the suggested text below if applicable across all interventions, otherwise the details should be provided in the applicable ISA.

The efficacy section should address when endpoints (eg, pain scores) are to be assessed with respect to dosing of rescue medication if relevant.

<Start of suggested text>

The study site [will/will not] supply [specify type] rescue medication that will be [provided by the sponsor/obtained locally]. The following rescue medications may be used:

- [X]
- [X]

Although the use of rescue medications is allowable [at any time during the study], the use of rescue medications should be delayed, if possible, for at least [insert timeframe] following the administration of study intervention. The date and time of rescue medication administration as well as the name and dosage regimen of the rescue medication must be recorded.

<End of suggested text>

# Discontinuation of Study Intervention and Participant Discontinuation/Withdrawal

Details should be kept at a minimum here. Include any actions to be taken if certain events are observed in an appendix and cross-reference that appendix as needed. Use schematics/algorithms if possible.

<Start of common text>

Discontinuation of specific sites or of the study as a whole are detailed in Appendix 1.

<End of common text>

## Discontinuation of Study Intervention

Describe the criteria for discontinuation of the participant from study intervention if applicable across all interventions, otherwise, details will be provided in the applicable ISA. See the SoA for data to be collected at the time of discontinuation of study intervention. Specify if participants who discontinue study intervention can or cannot continue the study (ie, continue with study visits).

As appropriate, consider using subheadings.

For single-dose studies, this section is not relevant. In such cases, state *not applicable* under Section 7.1.

The following criteria should be considered for inclusion in each of the subsections:

- Liver chemistry – see Section 7.1.1 Liver Chemistry Stopping Criteria
- Cardiac changes (eg, QTc) – see Section 7.1.2 QTc Stopping Criteria
- Pregnancy: cross-reference Section 8.3.5. Pregnancy
- Other safety criteria (eg, AE, PK criteria) – cross-reference Section 6.5 Dose Modification if relevant and do not replicate information provided there
- Disease-state criteria (eg, progressive disease)

<Start of common text>

In rare instances, it may be necessary for a participant to permanently discontinue study intervention. If study intervention is permanently discontinued, the participant [will/will not] remain in the study to be evaluated for [X]. See the SoA for data to be collected at the time of discontinuation of study intervention and follow-up and for any further evaluations that need to be completed.

<End of common text>

### Liver Chemistry Stopping Criteria

Liver injury – see participant libraries for proposed algorithm and text. Guidelines in the libraries are general and may be applicable to most clinical studies. Protocol authors should carefully evaluate if the liver-related stopping criteria are appropriate for the participant population and class of therapy evaluated in the clinical study and modify them, if needed.

If interventions being studied have specific stopping criteria due to known hepatotoxicities, these will be described in the applicable ISA.

<Start of common text for liver injury>

Discontinuation of study intervention for abnormal liver tests is required by the investigator when a participant meets one of the conditions outlined [in the algorithm] or in the presence of abnormal liver chemistries not meeting protocol-specified stopping rules if the investigator believes that it is in best interest of the participant.

Insert appropriate algorithm from relevant library.

<End of common text for liver injury>

### QTc Stopping Criteria

Insert appropriate text from relevant library if applicable to all interventions, otherwise, specific text should be included in the applicable ISA.

<Start of common text for cardiac changes>

If a clinically significant finding is identified (including, but not limited to, changes from baseline in QT interval corrected using [Bazett’s formula [QTcB] or Fridericia’s formula [QTcF]]) after enrollment, the investigator or qualified designee will determine if the participant can continue in the study and if any change in participant management is needed. This review of the ECG printed at the time of collection must be documented. Any new clinically relevant finding should be reported as an AE.

<End of common text for cardiac changes>

### Temporary Discontinuation

Include specifics around criteria for interrupting study intervention, what to do if the participant needs to stop study intervention, whether they will continue in the study, and whether all or specify which evaluations will be performed for the stated duration of the study should be described in the applicable ISA.

### Rechallenge

Specifics around rechallenge, as appropriate, should be described in the applicable ISA.

#### Study Intervention Restart or Rechallenge After Liver Stopping Criteria Are Met

This section should be presented in the applicable ISA unless applicable to all interventions.

<Start of common text if restart/rechallenge is NOT allowed>

Study intervention restart or rechallenge after liver chemistry stopping criteria are met by any participant in this study are not allowed.

<End of common text if restart/rechallenge is NOT allowed>

<Start of common text if restart/rechallenge IS allowed>

Study intervention [restart/rechallenge] after liver chemistry stopping criteria are met is allowed in this study. If the participant meets liver chemistry stopping criteria, do not [restart/rechallenge] the participant with study intervention unless:

- [Intervention-owner steering committee] approval **is granted**
- Ethics and/or IRB approval is obtained, if required, and
- Separate consent for intervention [restart/rechallenge] is signed by the participant

NOTE: If study intervention was interrupted for suspected intervention-induced liver injury, the participant should be informed of the risk of death, liver transplantation, hospitalization, and jaundice and reconsented before resumption of dosing.

Refer to Appendix 6 Liver Safety: Suggested Actions and Follow-up Assessments [and Study Intervention Restart/Rechallenge Guidelines] for details on the [restart/rechallenge] process.

If [Intervention-owner steering committee] approval to restart/rechallenge the participant with study intervention is **not granted**, then the participant must permanently discontinue study intervention and may continue in the study for protocol-specified follow-up assessments.

<End of common text if restart/rechallenge IS allowed>

## Participant Discontinuation/Withdrawal from the Study

Describe the criteria for withdrawal of participants from the study.

<Start of common text>

- Participant can withdraw from one intervention cohort and be screened/randomized to another cohort.
- A participant may withdraw from the study at any time at his/her own request or may be withdrawn at any time at the discretion of the investigator for safety, behavioral, or compliance reasons. This is expected to be uncommon.
- At the time of discontinuing from the study, if possible, an early discontinuation visit should be conducted, as shown in the SoA. See SoA for data to be collected at the time of study discontinuation and follow-up and for any further evaluations that need to be completed.
- The participant will be permanently discontinued from the study intervention and the study at that time.
- If the participant withdraws consent for disclosure of future information, the sponsor may retain and continue to use any data collected before such a withdrawal of consent.
- If a participant withdraws from the study, the participant may request destruction of any samples taken and not tested, and the investigator must document this in the site study records.

<End of common text>

## Lost to Follow up

Include a brief section on how the study will define and address participants who were lost to follow-up to help limit the amount and impact of missing data. Describe the nature and duration of follow-up, including follow‑up after discontinuation of intervention, as appropriate.

Include the final bullet of common text when follow-up status of a participant is critical to study outcomes

<Start of common text>

A participant will be considered lost to follow-up if the participant repeatedly fails to return for scheduled visits and is unable to be contacted by the study site.

The following actions must be taken if a participant fails to return to the clinic for a required study visit:

- The site must attempt to contact the participant and reschedule the missed visit as soon as possible, counsel the participant on the importance of maintaining the assigned visit schedule and ascertain whether the participant wishes to and/or should continue in the study.
- Before a participant is deemed lost to follow-up, the investigator or designee must make every effort to regain contact with the participant (where possible, [3] telephone calls, and if necessary, a certified letter to the participant’s last known mailing address or local equivalent methods). These contact attempts should be documented in the participant’s medical record.
- Should the participant continue to be unreachable, the participant will be considered to have withdrawn from the study.
- [Site personnel, or an independent third party, will attempt to collect the vital status of the participant within legal and ethical boundaries for all participants randomized, including those who did not get study intervention. Public sources may be searched for vital status information. If vital status is determined as deceased, this will be documented, and the participant will not be considered lost to follow-up. Sponsor personnel will not be involved in any attempts to collect vital status information.]

<End of common text>

# Study Assessments and Procedures

- Describe the assessments and procedures required during each phase of the study (eg, screening, Week 1). This should be consistent across all interventions. If any assessments and procedure are intervention-specific, these should be provided in ISA for the platform study. The full schedule of assessments will be presented in the applicable ISA (common and intervention-specific).
- Give all details that are not obvious from the SoA (eg, time of admission to the study site).
- Specify how unscheduled visit(s) will be handled and documented if not noted in the SoA.
- If the study includes qualitative interviews (or exit interviews), describe these evaluations.
- Describe methods/training to ensure consistency across centers, use of participant diaries, instructions on timing/conditions of assessments, and if a specifically qualified person (eg, physician, psychologist) should be performing these assessments. Specify that the same person should perform each assessment whenever possible. The procedures used, including means of maintaining the blind and centralized readings and measurements, should be described fully.
- Specify if the study allows for standard-of-care procedures as baseline assessments.
- All clinical outcome assessment (COA) parameters should be fully integrated into the appropriate sections of the protocol.
- COA is an umbrella term encompassing different types of outcomes:
  - Patient-reported outcome (PRO) measures – reported directly from the participant without interpretation by a clinician or anybody else
  - Clinician-reported outcome (ClinRO) measures
  - Observer-reported outcome (ObsRO) measures
  - Performance outcome (PerfO) measures
- If COA measures are used, include instructions for the investigators regarding the following:
  - Training and instructions provided to participants related to completing the questionnaires
  - Participant supervision during COA administration
  - Processes and rules for questionnaire review for completeness
  - Documentation of how and when data are filed, stored, and transmitted to or from the study site should be noted in the protocol or provided in a separate document.
- Details for maximum volume for blood draw, individual blood draws, and volumes required should be included if appropriate to the study.
- If a participant diary (paper or an electronic device) will be used to capture participant- or investigator-reported data, describe the steps to be taken to ensure that participants and/or investigators make entries according to the study design and not, for example, just before a study‑site visit when their reports will be collected.

<Start of common text>

- Potential participants will be fully informed of the risks and requirements of the study, and, during the study, participants will be given any new information that may affect their decision to continue participation. They will be told that their consent to participate in the study is voluntary and may be withdrawn at any time with no reason given and without penalty or loss of benefits to which they would otherwise be entitled. Only participants who are fully able to understand the risks, benefits, and potential AEs of the study, and provide their consent voluntarily will be enrolled.
- Study procedures and their timing are summarized in the SoA. See applicable ISA for intervention-specific assessments. Protocol waivers or exemptions are not allowed.
- Immediate safety concerns should be discussed with the intervention owner immediately upon occurrence or awareness to determine if the participant should continue or discontinue study intervention.
- Adherence to the study design requirements, including those specified in the SoA, is essential and required for study conduct.
- All screening evaluations must be completed and reviewed to confirm that potential participants meet all eligibility criteria. The investigator will maintain a screening log to record details of all participants screened and to confirm eligibility or record reasons for screening failure, as applicable.
- Procedures conducted as part of the participant’s routine clinical management (eg, blood count) and obtained before signing of the ICF may be utilized for screening or baseline purposes provided the procedures met the protocol-specified criteria and were performed within the timeframe defined in the SoA.
- [Safety/laboratory/analyte results] that could unblind the study will not be reported to investigative sites or other blinded personnel [until the study has been unblinded]. For such parameters that would lead to unblinding, please refer to Table.

<End of common text>

Include the maximum amount of blood collected from each participant over the duration of the study and if any repeat or unscheduled samples may be taken, as appropriate.

[The maximum amount of blood collected from each participant over the duration of the study, including any extra assessments that may be required, will not exceed [X] mL.]

[Repeat or unscheduled samples may be taken for safety reasons or for technical issues with the samples.]

## [Efficacy and/or Immunogenicity] Assessments

- Clearly list and define the specific measurements and assessments (including tools, equipment, instruments/questionnaires, laboratory tests, etc, with calibration methods if appropriate) that will be used for assessing and recording the efficacy data.
- The results of each primary and secondary outcome measure are required to be reported in the CT Registry; therefore, each measure and associated visits at which it will be evaluated across all interventions in the platform study should be clearly identified (visits should be captured in the SoA). For each measure, give the reference (where possible) and describe how scoring will be accomplished. If any outcome measures are specific to a certain intervention these should be described in the applicable ISA.
- Instructions or protocols for specialized tests may be presented in an appendix; however, do not use copies of case report forms (CRFs), published questionnaires, or rating scales as an appendix, as these will need to be redacted before disclosure. Any of these documents used in the study should be included in the CRF or annotated CRF with those not owned by the sponsor in a separate section for ease of redaction.
  - If any scale/questions are to be included in an appendix, it should be representative of what will be used in the CRF as it may change prior to study initiation or throughout the study. Consider including the following language in this section to address this circumstance, “Appendix [X] provides a representative example of the [scale] [questions] [specify other] that will be used in this study”.
- Any definitions used to characterize outcomes (eg, criteria for determining occurrence of acute myocardial infarction, designation of the location of the infarction, characterization of a stroke as thrombotic or hemorrhagic, distinction between transient ischemic attack and stroke, assignment of cause of death) should be explained in full if they are common to all interventions in the platform study.
- If categorical responses (global scales, severity scores, responses of a certain size) will be used in analyzing responses, they should be clearly defined.

Planned timepoints for all [efficacy and/or immunogenicity] assessments that are applicable to all interventions in the platform study are provided in the SoA.

## Safety Assessments

For each safety assessment:

- Specify how to perform, collect, and record each assessment (including tools, equipment, instruments/questionnaires, laboratory tests, etc, with calibration methods if appropriate); any limitations on personnel performing the assessment (eg, qualifications and training needed to conduct/interpret assessments, if an attempt should be made for the same individual to conduct that assessment throughout the study); and any definitions used to characterize outcomes.
- Specify methods used to standardize and/or interpret the assessment (eg, use of central laboratory, Holter monitoring, central ECG reader). Details can be provided in a separate document if they do not impact participant safety.
- Identify any noninvestigator party responsible for evaluation of laboratory or other safety assessments (eg, sponsor or external independent data monitoring committee) and describe any procedures used, including centralized reading/measurement.
- Include any questionnaires and rating scales used to classify laboratory or other safety assessments. Cross-reference Section 7 if linked with stopping criteria. Use validated scales. Reference the publication of the validation of the scale.
- Instructions or protocols for specialized tests may be presented in an appendix; however, do not use copies of CRFs, published questionnaires, or rating scales as an appendix, as these will need to be redacted before disclosure. Any of these documents used in the study should be included in the CRF or annotated CRF with those not owned by the sponsor in a separate section for ease of redaction.
  - If any scale/questions are to be included in an appendix, it should be representative of what will be used in the CRF as it may change prior to study initiation or throughout the study. Consider including the following language in this section to address this circumstance, “Appendix [X] provides a representative example of the [scale] [questions] [specify other] that will be used in this study”.
- Include guidelines for the management of relevant laboratory or other safety assessment abnormalities.
- Carefully evaluate inclusion/exclusion and withdrawal criteria to ensure any assessments required are included in the list of required tests. For example, Child-Pugh assessment requires measurement of albumin for calculation; thus, albumin needs to be included in the clinical chemistry parameters.
- Pregnancy testing and ECG monitoring should be included in safety evaluations regardless of whether they are collected only at baseline to determine eligibility or if they are repeated throughout the study.

Planned timepoints for all safety assessments that are applicable to all interventions in the platform study are provided in the SoA.

### Physical Examinations

- Provide content of the physical examination that will be applied to all participants in the platform study. Any examination needed for a specific intervention should be defined in the applicable ISA.
- Consider further specifications (eg, for height and weight measurements, the participant is allowed to wear indoor, daytime clothing with no shoes) if appropriate for the study.
- Include special instructions for assessing weight changes that may require dose adjustments. If the dose will be adjusted based on weight, provide details in Section 6 Study Intervention(s) and Concomitant Therapy.

<Start of suggested text>

- A complete physical examination will include, at a minimum, assessments of the [cardiovascular, respiratory, gastrointestinal, and neurological] systems. Height and weight will also be measured and recorded.
- A brief physical examination will include, at a minimum, assessments of the [skin, lungs, cardiovascular system, and abdomen (liver and spleen)].
- Investigators should pay special attention to clinical signs related to previous serious illnesses.

<End of suggested text>

### Vital Signs

- Carefully consider which vital signs (if any) should be measured to ensure that only essential data are collected.
- Provide content of vital sign measurements that will be performed in all participants in the platform study. Any measurement needed for a specific intervention should be defined in the applicable ISA.
- Include any specific instructions with respect to the collection and interpretation of vital signs. If orthostatic vital signs will be assessed, include instructions for supine and standing blood pressure and pulse measurements.
- Select the standard methods of vital sign collection as appropriate for the countries in which the study will be conducted.
- For studies requiring sensitive blood pressure monitoring (eg, if blood pressure decrease or increase is an anticipated effect), include details on device calibration requirements or frequency of measuring.

<Start of suggested text>

- [Oral] [Tympanic] [Rectal] [Axillary] [Skin] [Temporal artery] temperature, pulse rate, respiratory rate, and blood pressure will be assessed.
- Blood pressure and pulse measurements will be assessed [specify participant’s position, if applicable] with a completely automated device. Manual techniques will be used only if an automated device is not available.
- Blood pressure and pulse measurements should be preceded by at least 5 minutes of rest for the participant in a quiet setting without distractions (eg, television, cell phones).
- Vital signs (to be taken before blood collection for laboratory tests) will consist of 1 pulse and 3 blood pressure measurements (3 consecutive blood pressure readings will be recorded at intervals of at least 1 minute). The average of the 3 blood pressure readings will be recorded.

OR

- Vital signs will be measured in a [specify participant’s position, if applicable] position after 5 minutes rest and will include temperature, systolic and diastolic blood pressure, and pulse [and respiratory rate]. [Three readings of blood pressure and pulse will be taken. The first reading should be rejected. The second and third readings should be averaged to give the measurement to be recorded.]

<End of suggested text>

### Electrocardiograms

- Specify if the ECG is for screening purposes only.
- Provide content of ECG parameters/procedures that will be performed in all participants in the platform study. Any parameters/procedures needed for a specific intervention should be defined in the applicable ISA.
- Include any specific instructions for the collection and interpretation of ECGs (eg, time points relative to dosing with study intervention or other evaluations).
- Indicate whether single or triplicate ECGs will be collected at each time point. If triplicate ECGs will be collected, provide necessary details.
- If ECGs will be analyzed at a central laboratory, instructions for the collection (eg, equipment), transmission, and archiving of ECG data should be agreed upon with the central laboratory and summarized. The turnaround time for safety alerts from the central laboratory to the study site should be specified.
- If ECGs will be read locally, indicate if digital ECG waveforms will be centrally archived and in what format. If the digital waveforms will be archived, there is no need to retrieve paper ECGs from the study sites.
- Include instructions with respect to local review of ECG tracings for safety findings, even if ECGs will be analyzed at a central laboratory, and any actions to be taken in response to ECG findings.
- Consider consultation with the relevant sponsor cardiovascular safety committee.
- High-quality ECG data should be collected if the goal is to assess the effects of study intervention on ECG intervals such as the QT interval. Such ECG data may be required to meet regulatory authority expectations for a thorough ECG assessment (eg, as outlined in ICH E14) or to better assess a cardiac conduction signal from previous nonclinical or clinical studies. High‑quality ECGs are typically performed more frequently and in a more rigorous and more standardized fashion than routine ECGs. High-quality ECGs are typically recorded and archived in digital format using a central ECG vendor and analyzed by a specialized central laboratory.
- The frequency and timing of high-quality ECGs should reflect the PK of the study intervention and any metabolites. In general, ECGs should be conducted around key PK timepoints including the following: predose, maximum observed concentration (Cmax) after the first dose, and steady‑state Cmax. Additional measurements should be performed to account for potential PK differences between participants, unanticipated drug metabolites, delays between peak plasma/serum and tissue concentrations, and a PK lag effect. When possible, time-matched measurements should be considered (eg, collection of predose and postdose ECGs at a similar time of day) to minimize the effects of diurnal variation in ECG intervals.
- If high-quality ECG data are not collected in the study, ECG data must still be collected for routine safety monitoring of participants, at least until important study intervention effects on cardiac conduction or cardiac function have been sufficiently excluded in clinical studies. For studies investigating long-term dosing, ECGs should be obtained throughout the course of the study (eg, after each cycle of study intervention or monthly) as well as at the completion of the study.
- Ensure the correction formula listed here is consistent with that listed in the QTc exclusion and withdrawal criteria.
- Include the second bullet of suggested text if triplicate ECGs are to be obtained.

<Start of suggested text>

- [Triplicate OR Single] 12-lead ECG(s) will be obtained as outlined in the SoA (see Section 1.3) using an ECG machine that automatically calculates the heart rate and measures PR, QRS, QT, and [QTc] intervals. Refer to Section 7.1.2 for [QTc] withdrawal criteria and any additional [QTc] readings that may be necessary.
- [At each timepoint at which triplicate ECGs are required, 3 individual ECG tracings should be obtained as closely as possible in succession, but no more than 2 minutes apart.]

<End of suggested text>

### Clinical Safety Laboratory Tests

- Provide content of laboratory tests that will be performed in all participants in the platform study. Any laboratory tests needed for a specific intervention should be defined in the applicable ISA.
- For platform study, make every effort to ensure routine laboratory safety tests are performed by a central laboratory. If local laboratory tests are required, these must be stated clearly in the master protocol or applicable ISA. Provisions should be in place to allow for the acceptance of local laboratory data (even if a central laboratory is used). Sponsor databases should be set up appropriately for the reporting of data from both central and local laboratories. Consult with the data management representative for language to be included on how data should be reported to the sponsor if a local laboratory is used.
- Specify if the use of local laboratories is allowed in cases where initiation of study intervention or safety follow-up is time sensitive and the central laboratory results will not be available before the need to begin study intervention or other actions that need to be taken for safety reasons. These details should be described in the applicable ISA.
- Specify any special instructions for screening samples.
- Specify which laboratory parameters should be included in each panel (eg, for hematology, chemistry, urinalysis). List only those that will be analyzed for the study. Confirm lists and blood volumes before finalizing the protocol.
- See therapeutic area libraries for additional guidance.

<Start of common text>

- See Appendix 2 for the list of clinical laboratory tests to be performed and the SoA (Section 1.3) for the timing and frequency.
- The investigator must review the laboratory report, document this review, and record any clinically significant changes occurring during the study as an AE. The laboratory reports must be filed with the source documents.
- [Abnormal laboratory findings associated with the underlying disease are not considered clinically significant unless judged by the investigator to be more severe than expected for the participant’s condition].
- All laboratory tests with values considered clinically significantly abnormal during participation in the study or within [insert timeframe] after the last dose of study intervention should be repeated until the values return to normal or baseline or are no longer considered clinically significant by the investigator or medical monitor.
  - [If clinically significant/any] values do not return to normal/baseline within a period of time judged reasonable by the investigator, the etiology should be identified, and the sponsor notified.
  - All protocol-required laboratory tests required for all interventions in the platform study, as defined in Appendix 2, must be conducted in accordance with the laboratory manual and the SoA (Section 1.3).
  - If laboratory values from non-protocol-specified laboratory tests performed at the institution’s local laboratory require a change in participant management or are considered clinically significant by the investigator (eg, SAE or AE or dose modification), then the results must be recorded.

<End of common text>

### Pregnancy Testing

- Include the defined timepoints for pregnancy testing in WOCBP in the SoA. Timepoints should be based upon a risk assessment of the potential for genotoxicity and teratogenicity/fetotoxicity of the intervention(s) in the study. Risk should be determined for each intervention with input from the company’s nonclinical safety assessment group. Determination of risk for a marketed compound should also consider the risks outlined in the product label. Further guidance can be found in International Council on Harmonization [ICH] Guideline M3(R2) and Clinical Trial Facilitation Group (CTFG). As a minimum, a pregnancy test should be performed at screening to confirm absence of pregnancy and at the end of relevant systemic exposure. Additional testing may be required between the screening visit and the first dose of study intervention on Day 1. Consider additional pregnancy testing if the interval is:
  - ≤ 4 days, a repeat highly sensitive serum pregnancy test usually is not required
  - > 4 days, a repeat serum pregnancy test should be obtained
- For studies that have requirements for multiple pregnancy tests, add additional criteria as needed (eg, if there is a requirement for a test to be performed within a proximal timeframe prior to first dose, specify as inclusion criteria; if at a specified visit, or at the end of study intervention, note in the SoA and provide any necessary details here.
- A serum pregnancy test may diagnose pregnancy ~6 to 10 days after fertilization; a urine pregnancy test, because it is less sensitive, will diagnose pregnancy a few days after a serum pregnancy test. As serum pregnancy tests have a lower detection limit and will detect pregnancy closer to the date of conception, serum testing is the preferred test if there is a requirement to know pregnancy status within a few days of the first dose of study intervention.
- Decide if local or central testing will be standard for the protocol. Highly sensitive serum testing is mandatory if required by local regulations or the IRB/IEC, or to resolve an indeterminate test or confirm a positive urine test.

Insert library content here.

### Suicidal Ideation and Behavior Risk Monitoring

Clinical studies meeting either of the following 2 criteria must include appropriate assessments (eg, Columbia-Suicide Severity Rating Scale [C-SSRS]) to enable the prospective monitoring of suicidal ideation and behavior (SIB) in individual participants:

1. Participant or healthy volunteer studies using compounds that:

- are known to be active in the human central nervous system (CNS), or
- are being studied for CNS activity, or
- are being developed for any psychiatric or neurologic indication, or
- may affect mood, cognition, or behavior via their effects on the CNS (directly or indirectly), or
- are pharmacologically similar to medicines that have had SIB reported in association with their use, which is considered to be at least possibly causally associated (eg, isotretinoin and other tretinoins, beta blockers, reserpine, smoking cessation medicines and medicines for weight loss).

2. Studies including any participant population with an elevated risk of SIB, which may manifest during the study, and for which monitoring of SIB is considered to be in the best interest of participant safety and/or science.

Notes:

- Determination and documentation is made at a program level on a company-specific basis according to their practices. Assessment of SIB is difficult in participants with cognitive impairment of a degree that interferes with understanding of the concept of suicide (eg, Alzheimer’s disease, other dementias, learning disability, autism), and in participants who are terminally/critically ill. It is therefore reasonable to omit in these circumstances. If omission of SIB assessment is being considered for studies in challenging populations that would otherwise meet the criteria for monitoring, regulatory authority approval should be sought prior to protocol approval.
- Young children may not have reached sufficient cognitive maturity to understand the concept of death. As there is also no validated instrument for the prospective monitoring of SIB in children less than 7 years of age, all proposed studies in children less than 7 years that meet the criteria for monitoring of SIB should therefore be referred for regulatory and company’s internal review/advisory board for approval prior to protocol approval.
- It should be recognized that in uncontrolled studies, scientific interpretation of the results of monitoring of SIB may be difficult or impossible. Even so, if monitoring is important for participant safety it may be included.

<Start of example text>

[STUDY INTERVENTION/ACTIVE COMPARATOR] is considered to be a CNS-active intervention.

AND/OR:

[STUDY INTERVENTION/ACTIVE COMPARATOR] is related to products with an increased risk of suicidal ideation or behavior.

AND/OR:

Participants with [CONDITION] may occasionally develop suicidal ideation or behavior.

<End of example text>

<Start of suggested text>

Participants being treated with [study intervention X] should be monitored appropriately and observed closely for suicidal ideation and behavior (SIB) or any other unusual changes in behavior, especially at the beginning and end of the course of intervention, or at the time of dose changes, either increases or decreases. Participants who experience signs of SIB should undergo a risk assessment. All factors contributing to SIB should be evaluated and consideration should be given to discontinuation of the study intervention.

If study design calls for family and caregiver input, specify the need to communicate to these parties. For wording to be used in pediatric studies, see the pediatric participant library.

Specify how, if in the event of suicidal ideation or behavior, information will be shared with the legal guardian or others, including mental health professionals (local regulations should be followed). Address in the informed consent and assent forms as appropriate.

When informed consent or assent has been given, families and caregivers of participants being treated with [study intervention X] should be alerted about the need to monitor participants for the emergence of unusual changes in behavior, as well as the emergence of suicidal ideation and behavior and to report such symptoms immediately to the study investigator.

[Baseline assessment of suicidal ideation and behavior/intervention-emergent suicidal ideation and behavior] will be monitored during [study identifier] using [name of scale].

<End of suggested text>

## Adverse Events (AEs) Serious Adverse Events (SAEs), and Other Safety Reporting

- The means of obtaining AE data should be described (volunteered, checklist, or questioning) as should any specific rating scales used and any specifically planned follow-up procedures for specific AEs or any planned rechallenge procedures in case study intervention is discontinued because of an AE.
- If the study requires solicited and unsolicited adverse events to be collected then include the optional text in Section 8.3.1 and the optional table of definitions in Appendix 3.
- Consider whether there are any protocol-specific events that may need expedited reporting, or alternatively, are not required to be reported. Provide guidance for investigators. If there is a specific AE that will be of special interest it should be described in Section 8.3.8.

**NOTE: Level 3 headings** **in this section from 8.3.1 to 8.3.5 are common text and must be maintained to ensure the elements required by ICH and regulators are included in the protocol.**

<Start of common text>

The definitions of adverse events (AEs) and serious adverse events (SAEs) can be found in Appendix [3/7].

[The definitions of unsolicited and solicited adverse events can be found in Appendix 3].

AEs will be reported by the participant (or, when appropriate, by a caregiver, surrogate, or the participant’s legally authorized representative).

The investigator and any qualified designees are responsible for detecting, documenting, and recording events that meet the definition of an AE or SAE and remain responsible for following up [all AEs OR AEs that are serious, considered related to the study intervention or study procedures, or that caused the participant to discontinue the [study intervention] [study]] (see Section 7).

The method of recording, evaluating, and assessing causality of AEs and SAEs and the procedures for completing and transmitting SAE reports are provided in Appendix [3/7].

### Time Period and Frequency for Collecting AE and SAE Information

The first 2 paragraphs may be combined if the collection interval is the same for AEs and SAEs.

All SAEs will be collected from the [signing of the informed consent form (ICF) OR start of intervention] until [the follow-up visit] at the timepoints specified in the SoA (Section 1.3).

All AEs will be collected from the [signing of the ICF OR start of intervention] until [the follow‑up visit] at the timepoints specified in the SoA (Section 1.3).

[Unless considered as related to study conduct, medical occurrences that begin before the start of study intervention but after obtaining informed consent will be recorded as medical history/current medical conditions, not as AEs].

All SAEs will be recorded and reported to the sponsor or designee immediately and under no circumstance should this exceed 24 hours, as indicated in Appendix [3/7]. The investigator will submit any updated SAE data to the sponsor within 24 hours of it being available.

Investigators are not obligated to actively seek information on AEs or SAEs after conclusion of the study participation (defined as up to [insert timeframe] after the last dose of study intervention or last study procedure). However, if the investigator learns of any SAE, including a death, at any time after a participant has been discharged from the study, and the participant considers the event to be reasonably related to the study intervention or study participation, the investigator must promptly notify the sponsor.

### Method of Detecting AEs and SAEs

Care will be taken not to introduce bias when detecting AEs and/or SAEs. Open-ended and nonleading verbal questioning of the participant is the preferred method to inquire about AE occurrences.

For some studies, participants are not always able to provide valid verbal responses to open ended questions. In these circumstances, another method of detecting AEs and SAEs must be specified.

### Follow-up of AEs and SAEs

After the initial AE/SAE report, the investigator is required to proactively follow each participant at subsequent visits/contacts. All SAEs [and AEs of special interest (as defined in Section 8.3.8)] will be followed until resolution, stabilization, the event is otherwise explained, or the participant is lost to follow-up (as defined in Section 7.3). Further information on follow-up procedures is provided in Appendix [3/7].

### Regulatory Reporting Requirements for SAEs

For all studies except those using medical devices also include the last bullet.

- Prompt notification by the investigator to the sponsor or designee of an SAE is essential so that legal obligations and ethical responsibilities towards the safety of participants and the safety of a study intervention under clinical investigation are met.
- The sponsor or designee has a legal responsibility to notify both the local regulatory authority and other regulatory agencies about the safety of a study intervention under clinical investigation. The sponsor or designee and/or study conduct organization will comply with country-specific regulatory requirements relating to safety reporting to the regulatory authority, institutional review boards (IRBs)/independent ethics committees (IECs), and investigators.
- An investigator who receives an investigator safety report describing an SAE or other specific safety information (eg, summary or listing of SAEs) from the sponsor or designee will review and then file it along with the [IB/IDFU/package insert or state other documents] and will notify the IRB/IEC, if appropriate according to local requirements.
- [Investigator safety reports must be prepared for suspected unexpected serious adverse reactions (SUSARs) according to local regulatory requirements and sponsor policy and forwarded to investigators as necessary.]

NOTE: There may be incidences where events are potential efficacy endpoints, eg, in MACE studies. It is standard practice to exclude them from SAE reporting unless deemed possibly related to study intervention as long as this has been agreed up front with all of the relevant regulatory agencies. These events should be detailed in Section 8.3.7.

<End of common text>

### Pregnancy

- Define the time period for collecting pregnancy information for female participants or female partners of male participants as appropriate. This should align with the time period for postintervention contraception as described in Section 5.1.
- Do not collect pregnancy information for female participants known to be pregnant during the screening phase or before exposure to study intervention unless these participants enter the study, in which case consider whether pregnancy history needs to be collected.
- Specify any additional actions required (discontinuation of study intervention, withdrawal from the study), and any assessments that need to be performed.

<Start of common text>

- Details of all pregnancies in [female participants and, if indicated, female partners of male participants] will be collected after the start of study intervention and until [time period for reporting pregnancies should align with the time period for postintervention contraception determined in Section 5.1 or applicable ISA]. For female partners, consent to obtain pregnancy follow up information will be sought.
- If a pregnancy is reported, the investigator will record pregnancy information on the appropriate form and submit it to the sponsor [within 24 hours] of learning of the [female participant or female partner of male participant (after obtaining the necessary signed informed consent from the female partner)] pregnancy.
- While pregnancy itself is not considered to be an AE or SAE, any pregnancy complication or elective termination of a pregnancy for medical reasons will be reported as an AE or SAE.
- Abnormal pregnancy outcomes (eg, spontaneous abortion, fetal death, stillbirth, congenital anomalies, ectopic pregnancy) are considered SAEs and will be reported as such.
- The [participant/pregnant female partner] will be followed to determine the outcome of the pregnancy. The investigator will collect follow-up information on the [participant/pregnant female partner] and the neonate and the information will be forwarded to the sponsor.
- Any poststudy pregnancy-related SAE considered reasonably related to the study intervention by the investigator will be reported to the sponsor as described in Section 8.3.4. While the investigator is not obligated to actively seek this information in former [study participants/pregnant female partner], the participant may learn of an SAE through spontaneous reporting.

Any female participant who becomes pregnant while participating in the study [will discontinue study intervention or be withdrawn from the study] OR [may request continuation of study intervention.]

<End of common text>

Should a female participant become pregnant during the course of a study, under certain circumstances the study design may allow for the continuation of study intervention. In these instances, ICH guidelines and local regulations must be observed, and appropriate justification given in Section 4.2 Scientific Rationale for Study Design. In the absence of such justification female participants who become pregnant must be discontinued from study intervention.

Justification for continuation of study intervention may include the following circumstances:

1. The study intervention has an approved label that indicates it can be used safely in pregnant females

OR

1. The participant has a high mortality disease and the investigator determines the participant is benefiting from study participation and there is no other alternative treatment for her.

If continuation of study intervention following pregnancy is justified, the protocol should include details regarding what must occur prior to allowing continuation for that participant:

<Start of suggested text>

Prior to continuation of study intervention following pregnancy, the following must occur:

- The sponsor and the relevant IRB/IEC give written approval.
- The participant gives signed informed consent.
- The investigator agrees to monitor the outcome of the pregnancy and the status of the participant and her offspring.

<End of suggested text>

### Cardiovascular and Death Events

### Disease-related Events and/or Disease-related Outcomes Not Qualifying as AEs or SAEs

Specify if applicable any disease-related events (DREs) and/or disease-related outcomes that do not need to be reported as AEs or SAEs.

The following disease-related events (DREs) are common in participants with [disease, condition under study] and can be serious/life threatening:

- [Event A
- Event B
- Event C
- Event D]

Because these events are typically associated with the disease under study, they will not be reported according to the standard process for expedited reporting of SAEs even though the event may meet the definition of an SAE. These events will be recorded within [the appropriate timeframe]. [These DREs will be monitored by a/an [independent data monitoring committee, safety review committee, safety review team, other] on a routine basis. See Section 10.1.5]

NOTE: However, if either of the following conditions applies, then the event must be recorded and reported as an AE/SAE (instead of a DRE):

The event is, in the investigator’s opinion, of greater intensity, frequency, or duration than expected for the individual participant.

OR

The investigator considers that there is a reasonable possibility that the event was related to study intervention.

### Adverse Events of Special Interest

Consult the appropriate medically qualified team member if unsure if this section is applicable for a particular protocol.

The description should include the following:

- The definition of the event
- Is it a measurable quantity? If yes, how will the measurement be done?
- If it is a clinical event, how will it be confirmed?

<Start of suggested text>

The following are considered as AESI for all participants in this platform study:

- [A]
- [B]

For additional AESI applicable in some cohorts, see applicable ISA.

<End of suggested text>

### Overdose, medication errors, misuses or abuses of medicinal product

All reports of overdose, medication error, misuse or abuse in relation to the IMP with or without an AE must be recorded on the corresponding page(s) of the CRF and transmitted to the Sponsor’s representative following standard processes. The Investigator will assess whether or not the overdose, medication error, misuse or abuse event has to be reported together with an AE or SAE.

An overdose definition is given in [Section](#_Ref107922613) 6.8.

A medication error is an unintended failure in the drug treatment process (ie, mistake in the process of prescribing, storing, dispensing, preparing, or administering medicinal products in clinical practice) that leads to, or has the potential to lead to harm to the participant.

A misuse refers to situations where the medicinal product is intentionally and inappropriately used, ie, not in accordance with the terms of the marketing authorization or outside what is foreseen in the protocol, by the participant for a therapeutic purpose.

An abuse corresponds to the persistent or sporadic, intentional excessive use of a medicinal product, which is accompanied by harmful physical or psychological effects, ie, intentional non-therapeutic use of a medicinal product by a participant for a perceived reward or desired non-therapeutic effect including, but not limited to, “getting high”(euphoria).

This includes situations in which a participant was involved or not (eg, even if the error was recognized and intercepted before the participant received or used the product), and whether it resulted in harm to the participant or not. Of note, if a medication error or misuse meets the protocol definition of an overdose, it will be recorded in the overdose page of the CRF.

### Medical Device Deficiencies

This section is required for a study in which a medical device is provided for use in the study (ie, there are medical devices listed in Section 6.1.1 that are manufactured by the sponsor or by a third party for the sponsor). If Section 6.1.1 only includes nonsponsor medical devices, then this section is not needed.

- Instructions for documenting medical device deficiencies are provided in Appendix 7.

<Start of common text>

Medical devices are being provided for use in this study as the study intervention. To fulfill regulatory reporting obligations worldwide, the investigator is responsible for the detection and documentation of events meeting the definitions of device deficiency that occur during the study with such devices.

The definition of a medical device deficiency can be found in Appendix 7.

NOTE: Deficiencies fulfilling the definition of an AE/SAE will follow the processes outlined in Appendix 7 of the protocol.

#### Time Period for Detecting Medical Device Deficiencies

- Medical device deficiencies that result in an incident will be detected, documented, and reported during all periods of the study in which the medical device is used.
- If the investigator learns of any device deficiency at any time after a participant has been discharged from the study, and such a deficiency is considered reasonably related to a medical device provided for the study, the investigator will promptly notify the sponsor.

The method of documenting medical device deficiencies is provided in Appendix 7.

#### Follow-up of Medical Device Deficiencies

- Follow-up applies to all participants, including those who discontinue study intervention.
- The investigator is responsible for ensuring that follow-up includes any supplemental investigations as indicated to elucidate the nature and/or causality of the deficiency.
- New or updated information will be recorded on the originally completed form with all changes signed and dated by the investigator.

#### Prompt Reporting of Device Deficiencies to the Sponsor

- Device deficiencies will be reported to the sponsor within [24 hours] after the investigator determines that the event meets the protocol definition of a medical device deficiency.
- The medical device deficiency report form will be sent to the sponsor by [method]. If [method] is unavailable, then [alternative method] should be utilized.
- The sponsor will be the contact for the receipt of device deficiency reports.

#### Regulatory Reporting Requirements for Device Deficiencies

- The investigator will promptly report all device deficiencies occurring with any medical device provided for use in the study in order for the sponsor to fulfill the legal responsibility to notify appropriate regulatory authorities and other entities about certain safety information relating to medical devices being used in clinical studies.
- The investigator, or responsible person according to local requirements (eg, the head of the medical institution), will comply with the applicable local regulatory requirements relating to the reporting of device deficiencies to the IRB/IEC.

<End of common text>

## Pharmacokinetics

- Based on the study design, double-blind or open-label, the feasibility of the PK plan at the platform study-level will be determined. Depending on the design, PK may only be defined within each ISA.
- If population PK will be included for all interventions evaluated in the platform study, provide appropriate text. If PK will not be part of the study, include a statement to this effect.
- . Samples of plasma, urine, or other fluids may be taken for the purpose of measuring compliance, adjusting dose, or determining if a therapeutic window exists. Information that is specific to an intervention will be described in the ISA.
- information about sampling times, sample volume, sample handling procedures, assay methods, etc. Specific sample collection and processing including retention time instructions will be described in the ISA.
- Indicate definitions for the PK parameters (eg, area under the curve [AUC], maximum observed concentration [C_max_], time to C_max_ [T_max_], half-life [T_½_], volume of distribution [V_d_], clearance [CL]) of interest and how they will be calculated. Consult with the PK representative for this information.
- Do not reiterate the details given in the SoA or other sections of the protocol. Use cross-references as needed.

<Start of suggested text>

- [PK parameters are not evaluated in this study].

<End of suggested text>

OR

<Start of suggested text>

Refer to the applicable ISA for information regarding pharmacokinetics.

<End of suggested text>

<Start of suggested text>

- [Plasma/serum/whole blood/urine] samples of approximately [X] mL will be collected for measurement of [plasma/serum/whole blood/urine] concentrations of [study intervention/other] as specified in the SoA (Section 1.3) [specify timepoints only if not obvious from the SoA].
- A maximum of [X] samples may be collected at additional timepoints during the study if warranted and agreed upon between the investigator and the sponsor. The timing of sampling may be altered during the course of the study based on newly available data (eg, to obtain data closer to the time of peak plasma concentrations) to ensure appropriate monitoring.
- Instructions for the collection and handling of biological samples will be provided by the sponsor. The actual date and time (24-hour clock time) of each sample will be recorded.
- Samples will be used to evaluate the PK of the different study intervention(s) evaluated as part of the platform study. Each [plasma/serum/whole blood] sample will be divided into [X] aliquots (1 each for [PK, other analyses, and a backup]). Samples collected for analyses of [study intervention (plasma/serum/whole blood)] concentration may also be used to evaluate safety or efficacy aspects related to concerns arising during or after the study.
- Genetic analyses will not be performed on these [plasma/serum/whole blood] samples [unless consent for this was included in the informed consent]. Participant confidentiality will be maintained. At visits during which [plasma/serum/whole blood/etc] samples for the determination of [multiple aspects] of [study intervention] will be taken, one sample of sufficient volume can be used.

<End of suggested text>

## Genetics

- If this will not be part of the platform study across all interventions being evaluated, include a statement to this effect. If this is being done for certain interventions a detailed description will be provided in the applicable ISA.
- Contact the appropriate sponsor functional area representatives to ensure that appropriate generic genetic study design text is included throughout the protocol.
- See the appropriate guidelines/templates from the sponsor functional area representatives (eg, standard attachments for shipping and handling of laboratory samples). Dependent upon the volume of these attachments, they may be added to the ISA or provided in supplementary documents that will accompany the protocol.

<Start of suggested text>

Genetics are not evaluated in this study.

<End of suggested text>

<Start of suggested text>

Refer to the applicable ISA.

<End of suggested text>

OR

<Start of suggested text>

A [X] mL [blood OR saliva] sample for DNA isolation will be collected from participants in each cohort of the platform study who have consented to participate in the genetic analysis component of the platform study. Participation is optional. Participants who do not wish to participate in the genetic research may still participate in the study.

In the event of DNA extraction failure, a replacement genetic blood sample may be requested from the participant. Signed informed consent will be required to obtain a replacement sample unless it was included in the original consent.

[See Appendix 5 Genetics] for information regarding genetic research]. Details on processes for collection and shipment and destruction of these samples can be found in [specify location].

<End of suggested text>

## Biomarkers

If biomarkers will not be evaluated, include a statement to this effect. Do not delete the heading.

<Start of suggested text>

Biomarkers are not evaluated in this study.

<End of suggested text>

OR

<Start of suggested text>

Refer to the applicable ISA.

<End of suggested text>

OR

If biomarkers will be evaluated describe those that will be collected across all cohorts in the platform study. Biomarkers that are specific to an intervention will be described in the applicable ISA:

- Indicate the biological samples that will be collected (eg, serum, plasma, etc) across all cohorts.
- Indicate the types of biomarkers that will be studied across all cohorts otherwise, these will be included in the applicable ISA. For primary/secondary endpoints, specify biomarkers to be assessed. For exploratory biomarkers, there does not need to be a complete list of every biomarker, but the nature of the markers that may be measured, eg, inflammatory biomarker, including but not limited to TNFa, IL-6 etc. NOTE: Each matrix/endpoint will require its own paragraph as shown in the suggested text.
- Specify the retention time for the samples (this must match the information in the ICF; typically 15 years although country-level legislation may limit this) and that samples may be used for further research if consent is provided.
- If RNA-sequencing (RNA-Seq) is planned, ensure that this is specified in the consent.
- If instructions for collection of samples are complex, then consider including them in an appendix or laboratory manual rather than the main text of the protocol.

<Start of suggested text>

- DNA and biomarker samples may be used to enable the development of safer, more effective, and ultimately individualized therapies. Analyses of the samples will be performed at the discretion of the intervention owner, will always be under the intervention owner’s supervision, and may be reported separately. Any intervention-specific details will be described in the ISA.
- Any additional PD analyses or additional biomarkers associated with the study interventions will be presented in the applicable ISA. The goal of the pharmacogenomic component is to collect DNA to allow for the identification of genetic factors that may influence the PK, PD, efficacy, safety, or tolerability of the study intervention, and to identify genetic factors associated with the disease. Biomarker samples will be collected to examine the biological response to platform study interventions and to identify biomarkers that are relevant to the study intervention mechanism of action and/or the disease, or that may help to explain inter-individual variability in clinical outcomes, or may help to identify population subgroups that respond differently to a study intervention.
- [The total blood volume to be collected will be presented in each ISA.]
- [Specify type of sample, eg, plasma] samples will be collected to [protocol-specific objective]. Biomarkers will include [biomarker names]. Samples will be collected according to the schedule described in the SoA and as detailed in [laboratory manual provided separately to sites].
- [Specify type of sample, eg, blood] samples will be collected to [protocol-specific objective]. Biomarkers will include [biomarker names]. Samples will be collected according to the schedule described in the SoA and as detailed in [laboratory manual provided separately to sites].
- [Sponsor] may store samples for up to [X] years after the end of the platform study to achieve study objectives. Additionally, with participants’ consent, samples may be used for further research by [sponsor] or others such as universities or other companies to contribute to the understanding of [specify disease targeted in protocol] or other diseases, the development of related or new treatments, or research methods.

<End of suggested text>

## Immunogenicity Assessments

<Start of suggested text>

Refer to the applicable ISA.

<End of suggested text>

## [Health Economics OR Medical Resource Utilization and Health Economics]

If this section is not applicable, include a statement to this effect.

- Include this section only for any value evidence and outcomes assessment not included in either the efficacy or safety sections that are being evaluated for all interventions being studied in the platform study.
- Briefly describe the health outcome measures, collection method (eg, diary, physician interview), and participant burden.

<Start of suggested text>

Health economics OR Medical resource utilization and health economics parameters are not evaluated in this study.

<End of suggested text>

OR

<Start of suggested text>

For all participants throughout the study, the investigator and study site personnel will collect data about health care resource utilization associated with medical encounters.

The data collected will

- Include the reasons and duration of hospitalizations and emergency room visits and
- Exclude procedures, tests, and encounters mandated by the protocol.

The sponsor may use the collected data to conduct economic analyses.

<End of suggested text>

## 8.11 Use of Biological Samples and Data for Future Research

This section is mandatory and cannot be removed from the protocol. This Section corresponds to Part 2 of the core ICF template entitled “Information about future research”. The optional text ‘biological samples’ will be deleted if no biological samples are collected.

Where local law or IRB/IEC prohibits use of biological samples or data for future research, local ICF must be adapted and related changes documented centrally. See details on sample processing in relevant sections. Results from future research will not be entered in the study database. Protocol will not be amended if a country refuses future use.

<Start of common text>

Future research (research not prespecified by the project team when the protocol is developed and not included in the “drug development program”) may help further the understanding of disease and the development of new medicines. Reuse of coded data and biological samples (leftover and additional) will be limited to future scientific research conducted under a research plan for the purpose of diagnosing, preventing or treating diseases. The future research projects will be conducted under the Sponsor’s and/or, if applicable, the partner of the Sponsor which has licensed the study drug to the Sponsor or which is co-developing the study drug with the Sponsor’s control, acting alone or in collaboration with research partners such as universities, research institutions or industrial partners with whom the coded data may be shared.

Data and biological samples will be stored and used for future research only when consented to by participants and, when applicable, further information on the future research has been provided to the study participant, unless prohibited by local laws or IRBs/IECs (in such case, consent for future use of sample will not be included in the local ICF). The conditions for reuse will be adapted locally with the appropriate language in the ICF.

In any case, a specific consent will be collected for the performance of genetic analyses on leftover and/or additional samples.

**Data protection – Processing of coded clinical data**

The study participant will be provided will all mandatory details of the data processing in Section 2 of the core ICF.

The Sponsor adopts safeguards for protecting participant confidentiality and personal data (see Section 10.1.4).

**Use of leftover samples and additional samples for future research**

Remaining leftover samples will be used only after the study ends, ie, end of study as defined in the study protocol. Additional/extra samples can be collected and used during the study conduct at a given timepoint (eg, at randomization visit) as defined in the study protocol.

The study participant will be provided with all mandatory details of the use of the human biological samples (leftover and additional) in Section 2 of the Core ICF.

Relating data will be stored for up to 25 years for regulatory purposes and future research. Biological samples for future use will be stored for up to 25 years after the end of the study. Any samples remaining at the end of retention period will be destroyed. If a participant requests destruction of his/her samples before the end of the retention period, the Investigator must notify the Sponsor (or its contract organization) in writing. In such case, samples will be destroyed and related coded data will be anonymized unless otherwise required by applicable laws.

<End of common text>

# Statistical Considerations

<Start of suggested text>

A statistical analysis plan that encompasses the entire platform study is available for the master protocol. Statistical analysis for each intervention cohort will be performed by the intervention owner or under the authority of the sponsor. A general description covering the main aspects of the statistical analysis of the platform study is given below. When analyses for intervention cohort deviate from the overall statistical analysis plan, a separate/joint statistical analysis plan (SAP) will be developed. For each intervention, details will be available in the applicable ISA and in the corresponding SAP, as applicable, and will be finalized before the first unblinding of efficacy data (as required by ICH E9).

<End of suggested text>

## Statistical Hypotheses

- Clearly articulate the statistical hypotheses that will be the subject of statistical testing related to each key estimand that are applicable to all interventions in the platform study. In case no hypotheses are planned to be tested state this in this section.
- Any statistical hypotheses that are intervention-specific should be specified in Section 9.1 of the relevant ISA
- Describe the statistical hypotheses for the primary and secondary objectives of the platform study. Even when a statistical hypothesis is not applicable for the platform study, the study objective should still be translated into a statistical statement, such as “… estimate the 95% confidence interval…”, and “…to generate the descriptive statistics for exploratory purpose …”, etc.
- If a frequentist statistical hypothesis test is used, clearly state the null and alternative hypothesis and the significance level (one-sided or two-sided and include a rational for the choice).
- In the Bayesian framework, if a general set of decision rules are being made based on posterior probabilities that are applicable to all interventions in the platform study then this would be described here. If such rules are intervention specific, they will be described in the ISA, as appropriate.
- If a study is intended to generate new hypotheses then the criteria by which this done should be described here accordingly (e.g. if in the 1st stage of a multi-arm multi-stage study (MAMS), biomarkers are identified to select participants for inclusions in subsequent stages of the design).
- Please reference back to the Estimands Section of the Protocol (Section 3) for the definition of endpoints and estimands that are connected to these statistical hypotheses adding any details that are necessary to allow the reader to understand the endpoints to which the hypotheses are being applied.

<Start of example text>

Example 1:

The primary objective is to demonstrate that intervention is superior to [control] in achieving [outcome] at [timepoint]. Thus, the null hypothesis to be tested in relation to the primary estimand is as follows:

- Null hypothesis: Intervention is not different from [control] with respect to the achievement of [outcome] at [timepoint].

The null hypotheses corresponding to the secondary estimands are as follows:

1. Intervention is not different from [control] with respect to the achievement of [outcome/endpoint] at [timepoint]
2. Intervention is not different from [control] with respect to change from baseline to [timepoint] in [health measurement/outcome]

Example 2:

For the primary estimand with primary endpoint, change from baseline to [timepoint] in [health measurement or observation]_,_ the following 2 (confirmatory) 1-sided hypotheses are planned to be tested for [intervention x] versus [intervention y]. Let the mean treatment difference be defined as μ = ([intervention x] minus [intervention y]).

Noninferiority (noninferiority margin is [noninferiority margin])

H0: μ ≥ [noninferiority margin] against Ha: μ < [noninferiority margin]

Superiority

H0: μ ≥ 0.0 [unit] against Ha: μ < 0.0 [unit]

Operationally the hypotheses will be evaluated by 2-sided tests.

<End of example text>

### Multiplicity Adjustment

- Clearly state the method for controlling overall type I error.
- State not applicable if multiplicity control is not relevant for the study.
- If in the platform study, multiplicity is being controlled only in specific ISAs refer to details in Section 9.1.1 of the ISA
- If Bayesian decision rules are used, multiplicity could be discussed in term of frequentist error rates.
- In a platform study, different set of families of hypotheses might be defined, e.g., for each intervention-control comparison including a pre-defined set of primary and secondary endpoints. A rational must be given. If the same adjustment methods are applied across all intervention cohorts in the master protocol, describe the details in the master protocol itself. If multiplicity adjustment strategies differ between intervention cohorts (due to different populations/treatments), describe which error rates should be controlled for which family of hypotheses, but you may provide more technical details in Section 9.1.1 of the corresponding ISA.

<Start of example text>

Example 1 (linked to example 1 in the statistical hypotheses):

The statistical comparisons for the primary efficacy endpoint and the key secondary endpoints will be carried out in the hierarchical order as indicated in Section 9.1 for each intervention cohort. This means that statistically significant results for the comparison in the higher rank (primary, then ranked secondary variables) are required to initiate the testing of the next comparison in the lower rank. Since a step-down procedure is used, each comparison will be tested at a significance level of 0.05 and an overall alpha level of 0.05 will be preserved.

Example 2 (linked to example 2 in the statistical hypotheses):

The type I error will be controlled in the strong sense using a hierarchical (fixed sequence) testing procedure. This is based on priority ordering of the null hypotheses and testing them in this order using the 2-sided 95% confidence interval approach until an insignificant result appears. Consequently, the second null hypothesis will only be tested if the first null hypothesis has been rejected in favor of [intervention x].

The steps in the hierarchical testing procedure are as follows:

Step 1: [health measurement or observation] noninferiority of [intervention x] versus [intervention y]

Step 2: [health measurement or observation] superiority of [intervention x] versus [intervention y]

<End of example text>

## Analysis Sets

- In studies with estimands explicitly defined (mandatory for confirmatory studies), the analysis sets can be derived from the estimand description. However, for transparency, it is useful to specify in detail which participants and which data points are to be included in each analysis set used to estimate each estimand defined in the protocol. Note, naming of the sets of selected participants and not only the full data set (participants and data points) is recommended for ease of programming of the ADaM data set.
- The analysis sets should at minimum be defined at the participant level and the data points to be included can be specified, if deemed relevant.
- Definitions that apply to entire platform study should be described in the master protocol. Any definitions that are intervention-specific should be described in Section 9.3 of the relevant ISA.

Three examples are provided for illustration purposes only. Other formats, other definitions, and different naming conventions can be used. The first example is for a study with no estimands explicitly defined and the second and the third examples address a study with estimands explicitly defined.

<Start of example text>

Example 1:

For the purposes of analysis, the following analysis sets are defined:

| **Participant Analysis Set** | **Description** |
| --- | --- |
| Full analysis set | - All randomized participants. Participants will be included in the analyses according to the planned intervention. |
| Safety analysis set | - All participants who are exposed to study intervention. Participants will be included in the analyses according to the intervention they actually received. |

The full analysis set is used to analyze endpoints related to the efficacy objectives and the safety analysis set is used to analyze the endpoints and assessments related to safety.

Example 2:

The following analysis data sets are defined to estimate the estimands defined in the protocol and to address safety.

| **Analysis Data Sets** | **Description** |
| --- | --- |
| Analysis set 1 for the primary estimand and for the secondary estimand for the secondary objective 1 | - PAS1: All randomized participants. Participants will be included in the analyses according to the planned intervention. - For participants who discontinue study intervention and/or receive rescue therapy, post-discontinuation or post-rescue observations will not be included. |
| Analysis set 2 for the additional estimand for the primary objective | - PAS1: All randomized participants. Participants will be included in the analyses according to the planned intervention. - For participants who discontinue study intervention and/or receive rescue therapy, all post-discontinuation or post-rescue observations will be included in the analysis set. |
| Safety analysis set 1 to be used for safety assessments with a long lag-time | - PAS2: All participants who are exposed to study intervention. Participants will be included in the analyses according to the intervention they actually received. - All observed data will be included in the analysis set. |
| Safety analysis set 2 to be used for safety assessments with an acute onset | - PAS2: All participants who are exposed to study intervention. Participants will be included in the analyses according to the intervention they actually received. - All observed data until discontinuation of intervention will be included in the analysis set. |

PAS = participant analysis set

Example 3:

The following participant analysis sets are defined:

| **Participant Analysis Set** | **Description** |
| --- | --- |
| Full analysis set (FAS) | - All randomized participants. Participants will be included in the analyses according to the planned intervention. |
| Safety analysis set | - All participants who are exposed to study intervention. Participants will be included in the analyses according to the intervention they actually received. |

The following data points sets are defined:

| **Defined Data Points Sets** | **Description** |
| --- | --- |
| DPS1 | - For participants who discontinue study intervention and/or receive rescue therapy, post-discontinuation or post-rescue observations will not be included. |
| DPS2 | - For participants who discontinue study intervention and/or receive rescue therapy, all post-discontinuation or post-rescue observations will be included in the analysis set. |
| DPS3 | - All observed data will be included in the analysis set. |
| DPS4 | - All observed data until discontinuation of intervention will be included in the analysis set. |

FAS and DPS1 are used to estimate the primary estimand and the secondary estimand for secondary objective 1.

FAS and DPS2 are used to estimate the additional estimand for the primary objective.

Safety analysis set and DPS3 are used to present safety data with a long lag-time.

Safety analysis set and DPS4 are used to present safety data with an acute onset.

<End of example text>

## Statistical Analyses

<Start of suggested text>

The master statistical analysis plan for the platform study will be finalized prior to [un-blinding/FPFV/DBL] of the first intervention evaluated and it will include a more technical and detailed description of the statistical analyses that will apply to all interventions, described in this section. This section is a summary of the planned statistical analyses of the most important endpoints including primary and key secondary endpoints that are evaluated for all interventions in the platform study. Any key endpoints that are specific to an intervention will be described in the applicable ISA, if required.

<End of suggested text>

### General Considerations

This section should describe general methods and definitions that do not need to be repeated in the subsequent sections. For example, a general statement that all treatment group comparisons for all categorical analyses will be tested using Fisher’s exact test does not need to be repeated for each categorical analysis described in later subsections. The same would be true for analysis of variance information and the model used. If different definitions are required for specific analyses, these should be stated in the relevant section. Suggested topics to be included in this section, if appropriate for the study, are provided. If subsections are used, this section can have the section heading only, with no text required.

- Decision criteria, such as nominal significance levels, 1- or 2-sided tests, and confidence interval probabilities if not already specified in Section 9.1
- Common definitions of baseline
- For randomized studies, describe stratification factors if applicable, and if not specified in Section 4.1
- General methods, such as handling of wrong stratification, wrong intervention assignment, handling of values below lower limit of quantification, continuous variables will be summarized with min, max, mean, median, std, quantiles, etc
- General choice of analysis sets for analyses
- Pooling strategies for countries/regions, sites, etc
- Intervention grouping strategy, eg, combining all active dose arms versus control
- Handling of missing baseline values, if this is planned to be handled in the same way across analyses
- Definition of study periods if needed
- For studies with estimands, describe how intercurrent events will be summarized (number by intervention group and timing).
- Definition of which study intervention contrasts will be provided.
- Any definitions that are applicable to specific interventions should be described in Section 9.3.1 of the ISA.
- Definition of how the control group will be defined across all of the interventions, any adaptations or sharing that will be broadly required should be outlined here. Intervention grouping strategy, eg, combining all active dose arms versus control.

<Start of suggested text>

The statistical analysis will be performed in alignment with the following standards and regulations: [fill in as appropriate, for example ICH-E9 standards]. All available data will be used in the analysis [otherwise, specify here the exceptions]. The following paragraphs describe the statistical analysis for the evaluation of the primary and secondary endpoints and other analysis that will be performed at the end of the study. Interim analysis are described in Section 9.4.

Participant demographics and baseline characteristics will be summarized on the XXX set, overall and by randomized treatment group, by means of summary descriptive statistics.

For qualitative variables (e.g. sex), absolute (n= ) and relative frequencies will be calculated per treatment group. Data will be visualized by bar plots. For quantitative data (e.g. age), the number of valid observations (n = ), mean, standard deviation, standard error, median, interquartile range, minimum and maximum will be calculated for each randomized group.

Data will be visualized by [write here the more relevant graphical representation with respect to the aim of the descriptive statistics (e.g. histogram or boxplot) and eventually described whether reference/normative values will be plotted].

<End of suggested text>

### Primary Endpoint(s)/Estimand(s) Analysis

- State how the primary endpoint(s) will be defined/calculated/derived and used to address the primary objective.
- Refer to the analysis set to be used in the main analytical approach(es).
- Refer to the Statistical Hypotheses section (Section 9.1 of this template) regarding the hypothesis to be tested, if applicable.
- Refer to estimand(s) in Section 3 and ICH E9 (R1) if applicable. In case of more than one primary estimand due to different requirements across different regulatory agencies, describe the analysis of the primary endpoint for all primary estimands and indicate which estimands are required by which authorities if this is not clear from Section 3. If no estimands are defined, please describe how important anticipated protocol deviations will be handled. If for some reason primary estimand is not applicable across intervention cohorts of the platform study, the primary estimand for that intervention should be described in Section 9.3.2 of the relevant ISA.
- Describe the main analytical approach(es) (aligned to the primary estimand[s], if applicable), including description of assumptions, including assumptions on the missing data mechanism. Describe how missing data will be handled and describe (if applicable) factors, covariates, stratification factors, etc to be included in the analysis model. If imputation methods differ across different intercurrent events, describe how. In case of multiple imputation, state imputation model, number of datasets, and seed. Specify how datasets will be combined.
- Describe the planned sensitivity analyses and how the sensitivity analyses will target the assumptions behind the main analytical approach. Pay special attention to assumptions regarding the missing data mechanism.
- Describe any supplementary analyses. This could be estimation of additional estimands defined in Section 3 for the primary objective. If additional estimands are not mentioned in Section 3 and supplementary analyses are planned, the estimands should be defined in this section. If not described in Section 9.2 Analysis Sets, describe which participants and data points are included in the analysis set to be used to estimate each of the estimand(s) related to supplementary analyses. Consider if supplementary analyses including estimand definitions related to them can be moved to the SAP. Only those supplementary analyses deemed appropriate and relevant to all interventions in the platform study should be presented. Any supplementary analyses that are intervention-specific should be presented in Section 9.3.2 of the relevant ISA.

<Start of suggested text>

The [main] primary endpoint of the study is [cite the main primary endpoints with its formal definition in terms of type of variables (e.g. presence/absence outcome or survival outcome or continuous endpoints)]. The evaluation of this endpoint will be done by [summarize here the statistical method used for testing and estimation, specifying the variables and the eventual considered covariates, the eventual needed assumptions, whether the analysis will be done also on specific subsets, how missing data will be handled].

Discuss the analysis set.

- In a platform study it is important to discuss if the comparison is made against a control group vs concurrent controls or non-concurrent controls [add some more details on platform studies]. The ISA analyses on the primary endpoint include participants allocated to the intervention and equivalent participants contemporaneously allocated to the control arm while the intervention is open to recruitment.

[Add a similar paragraph for any additional primary endpoint].

<End of suggested text>

### Secondary [Endpoint(s)/Estimand(s)] Analysis

- Key/confirmatory secondary endpoint(s)/estimand(s) (eg, for which a label claim is pursued) are part of the confirmatory hypotheses where the type 1 error is controlled (via multiplicity adjustment). It is recommended to describe the analysis of such endpoints to the same level of detail as the primary endpoint(s)/estimand(s) being described in Section 9.3.2. If the same methodology/analytical approach is taken for these endpoints, it will be sufficient to add a cross-reference to Section 9.3.2 to avoid redundancy. If there are any deviations to the control of the type I error rate or other error rates being controlled across interventions that are unique to a specific intervention cohort this information should be described in Section 9.3.2 of the ISA.
- Analysis of other (supportive) secondary endpoint(s)/estimand(s) need not be described with the same level of detail as the key secondary endpoints. A description of supportive secondary endpoints/estimands can be omitted from the protocol section with a reference made to the SAP.

<Start of suggested text>

The [first] secondary endpoint of the study is [cite the first secondary endpoint with its formal definition in terms of type of variables (e.g. presence/absence outcome or survival outcome or continuous endpoints)]. The evaluation of this endpoint will be done by [summarize here the statistical method used, specifying the variables and the eventual considered covariates, the eventual needed assumptions, whether the analysis will be done also on specific subsets, how missing data will be handled].

[Add a similar paragraph for any additional secondary endpoint].

<End of suggested text>

### [Tertiary/Exploratory/Other] [Endpoint(s)/Estimand(s)] Analysis

It is optional if a description will be provided in the protocol section or in the SAP. If the description is provided in the SAP only a reference to the SAP should be made in this section. If the description is provided in the protocol section, it is recommended to describe the analyses to the same level of detail as the supportive secondary endpoints/estimands. For example, no sensitivity or supplementary analyses need to be specified for tertiary/exploratory/other endpoints/estimands. The content included should here should only be for those endpoints/estimands that are applicable across all intervention cohorts. Any tertiary/exploratory endpoints/estimands that are unique to a specific intervention should be described in Section 9.3.4 of the ISA.

### [Other] Safety Analyses

- Describe at a high level how the safety data will be analyzed if not already described in either Section 9.3.2, Section 9.3.3, or Section 9.3.4. Refer to the SAP for details. Any analyses of safety data that are intervention-specific should be described in Section 9.43.5 of the relevant ISA.
- Specify the analysis set to be used or refer to Section 9.2.
- Specify estimands, if applicable.

<Start of suggested text>

*All safety analyses across all interventions in the platform study will be based on the Safety analysis dataset including all randomized participants who are exposed to study intervention(s). Participants will be analyzed according to the intervention(s) they actually receive.*

[*In addition, similar analyses will be performed on the Expanded Safety analysis dataset including all randomized participants who are exposed to the investigational intervention and participants who are randomized and exposed to the control(s) across available intervention cohorts.*]

**Adverse events**

*The verbatim terms used in the CRF by investigators in the platform study to identify adverse events will be coded using the Medical Dictionary for Regulatory Activities (MedDRA). Intervention-emergent adverse events are adverse events with onset during the* [lead-in, intervention, follow-up] *phase or that are a consequence of a pre-existing condition that has worsened since baseline. All reported adverse events will be included in the analysis. For each adverse event, the number (percentage) of participants who experience at least 1 occurrence of the given event will be summarized by intervention arm. AEs of special interest to any specific study intervention will be evaluated.*

*Summaries, listings, datasets, or participant narratives may be provided, as appropriate, for those participants who die, who discontinue intervention due to an adverse event, or who experience a severe or a serious adverse event.*

[Parameters with predefined National Cancer Institute Common Terminology Criteria for Adverse Events (NCI-CTCAE) toxicity grades will be summarized. Change from baseline to the worst adverse event grade experienced by the participant during the study will be provided as shift tables.]

**Clinical Laboratory Tests**

*Laboratory data will be summarized by type of laboratory test. Reference ranges will be used in the summary of laboratory data. Descriptive statistics will be calculated for each laboratory analyte at baseline and for observed values and changes from baseline at each scheduled time point. Results will be presented in pre- versus post-intervention cross-tabulations (with classes for below, within, and above normal ranges). Frequency tabulations of the abnormalities will be made. A listing of participants with any laboratory results outside the reference ranges will be provided.*

[*The laboratory abnormalities will be determined according to the criteria specified in the DAIDS Toxicity Grading Scale3 and in accordance with the normal ranges of the clinical laboratory if no gradings are available.]*

[*Markedly abnormal laboratory results specified in the Statistical Analysis Plan, will be summarized and a listing of participants with any markedly abnormal laboratory result will be provided.]*

Any analyses specific to an intervention in the platform study should be described in the ISA.

**Electrocardiogram**

*Electrocardiogram data will be summarized by ECG parameter. Descriptive statistics will be calculated at baseline and for observed values and changes from baseline at each scheduled time point. Frequency tabulations of the abnormalities will be made.*

*The ECG variables that will be analyzed are heart rate, PR interval, QRS interval, QT interval, and corrected QT (QTc) interval using the following correction method:* [*QT corrected according to Bazett's formula (QTcB), QT corrected according to Fridericia's formula (QTcF)*.]

[*Descriptive statistics of QTc intervals and changes from baseline will be summarized at each scheduled time point. The number (percentage) of participants with QTc interval >450 milliseconds, >480 milliseconds, or >500 milliseconds will be summarized, as will the number (percentage) of participants with QTc interval increases from baseline >30 milliseconds or >60 milliseconds.*]

Any analyses specific to an intervention in the platform study should be described in the ISA.

**Vital Signs**

*Descriptive statistics of* [*temperature, pulse/heart rate, respiratory rate, and blood pressure (systolic and diastolic)*] *values and changes from baseline will be summarized at each scheduled time point. The number (percentage) of participants with values beyond clinically important limits will be summarized.*

Any analyses specific to an intervention in the platform study should be described in the ISA.

**Physical Examination**

*Physical examination results and findings will be summarized.*

Any analyses specific to an intervention in the platform study should be described in the ISA.

**Suicidal Ideation and Behavior Risk Monitoring**

[*Columbia Suicide Severity Rating scale (C-SSRS) data from participants with suicidal ideation or behavior at any time during the study will be summarized.*]

Any analyses specific to an intervention in the platform study should be described in the ISA.

<End of suggested text>

### Other Analyses

A description can be omitted from the protocol section and a reference made to the SAP. Alternatively, a high-level description can be provided and further detailed in the SAP, if not fully detailed in the study protocol.

Analysis of other variables and/or parameters and subgroup analyses belong in this section.

**Other variables and/or parameters:**

Other analyses may include analyses of assessments or derived parameters, which are not defined as endpoints but need to be prespecified in either the protocol or SAP. Examples include but are not limited to immunogenicity, biomarkers, PK/PD/population PK parameters, health care utilization variables, and health technology assessment-related variables. State if these will be reported in a separate document. Other analyses that are not being applied across all interventions should be described in the applicable ISA.

Subsections may be used for different topics.

It is recommended that the variables used in the analyses should be clearly defined and the analyses should be described at the same level of detail as the supportive secondary endpoint(s)/estimand(s).

The definition and derivation may be specified in a table format.

Specify estimands if applicable.

**Subgroup analyses:**

Note: Often individual standard clinical studies (excluding large outcomes or safety studies) are not designed to allow for statistically meaningful subgroup analyses because of too small sample sizes. Also, subgroup analyses are not commonly included in the set of multiplicity-controlled analyses and are therefore subject to multiplicity issues.

It is recommended to consider addressing the following topics, if applicable:

- Define the endpoints subject to subgroup analysis – can be for either efficacy and safety, or both.
- Define subgroups (may include stratification factor, if relevant).
- Provide the purpose (consistency, hypothesis) of each subgroup analysis. The subgroup analyses should preferably be further substantiated, eg, biological plausibility of anticipated differential effect, regulatory/payer requirement.
- Specify any rules to define the minimum size of a subgroup in order to carry out the analysis.
- Specify analysis sets/estimand(s), as applicable.
- Specify the subgroup analysis methods including how missing data are handled.
- Specify the level of significance for the test of the treatment-by-subgroup interaction, if applicable.
- Assess consistency across regions and subpopulation(s) for multiregional clinical studies, as specified in ICH E17.
- Describe how results will be presented. It is recommended to focus on estimates and confidence intervals rather than p-values. It is often useful to display the results in a forest plot.

<Start of example text>

Subgroup analyses of the primary endpoint and confirmatory secondary endpoints will be made to assess consistency of the intervention effect across the following subgroups:

- Age group: < 65 vs ≥ 65 years
- Sex: female vs male
- Race: white vs black vs. other

If the number of participants is too small (less than [10%]) within a subgroup, then the subgroup categories may be redefined prior to unblinding the study. Further details on the statistical analysis will be provided in the SAP.

<End of example text>

## Interim Analysis

If an interim analysis is planned for the platform study for at least one intervention cohort, , describe if any type of data monitoring committee will be established to evaluate the interim analyses (the safety data and/or the critical efficacy endpoints) in accordance with ICH E9. Also describe the role of the committee (eg, making recommendation to the sponsor whether to continue, modify, or stop a study or intervention cohort). Full details of the committee including any charters should be included in Appendix 10.1.5 Committees Structure. Details that are intervention-specific should be described in the applicable ISA, as appropriate.

The following information belongs in this section:

- Reason for conducting interim analyses and the impact on the conduct of the study (e.g. is the interim analysis administrative? Is the interim analysis being conducted to assess futility of an investigational treatment being evaluated? Is the interim analysis being conducted to assess superiority of an investigational treatment and advance it to the next stage of development? Are interim analyses being performed to assess futility and superiority?)
- Timing of interim analysis relative to intervention cohort recruitment and participant follow-up (e.g. will it be milestone-based or time frequency based as such that certain criteria are met (events, number of participants, follow-up time) this would trigger specific interventions to be included as part of the interim analysis.
- Endpoints to be included in the interim analyses
- Timing of the interim analyses (eg, approximate number of participants enrolled, approximate number of participants completing a certain number of visits, number of events, calendar time)
- (Adaptive) making criteria and stopping rules (e.g., for efficacy, binding/unbinding futility rules) and impact on operating characteristics. Discuss if stopping rules have been addressed in the sample size calculation), if applicable to all interventions.
- Any actions resulting from an interim analysis such as sample size re-estimation or stopping rules, if the rules are applicable to all interventions
- If adaptations are envisaged: type of adaptive design with details of the pre-planned study adaptations and the statistical information informing the adaptations (e.g., will adaptations be based on primary endpoint and/or secondary endpoints)
- If response adaptive designs are used: give details on burn-in period and if and how long control group allocation is fixed, algorithm to modify allocation ratio and information to be used for updates, if this can be done generically at the platform study level. Otherwise, such text should be described in the ISA.
- Discuss how interim analysis impacts statistical testing and estimation across all interventions being evaluated in the platform study.
- Multiplicity considerations relating to the interim and final analyses
- Blinding/ unblinding strategy

<Start of example text>

For each ISA, an interim analysis for futility and superiority will be conducted to address uncertainty on effect assumptions and to maintain efficiency in resource allocation across the platform. The interim analysis on the primary endpoint will be performed by the independent data monitoring committee (IDMC) consisting of [X] clinicians and 1 statistician who are independent experts not otherwise involved in the study when approximately [X] primary events have occurred. The analysis method for the primary efficacy endpoint described in Section 9.3.2 Primary Endpoints/Estimands will be used for the interim analysis. Based on the group sequential design with the [O’Brian Fleming] alpha spending approach, a 2-sided alpha of [X] will be allocated to the interim analysis. In addition, if the conditional power for the final analysis (based on the original assumption for the remaining study) is [X] or lower, the study may be stopped for futility.

The interim analysis will be conducted such that the ongoing study integrity is maintained. Only the independent statistical support group, who is responsible for providing the interim analysis results to the IDMC will be unblinded to the individual treatment group assignments. Interim analysis results will not be shared with investigators, participants, or the study team who are involved in the conduct of the study before the final database lock.

The statistical analysis plan will describe the planned interim analyses in greater detail.

Type of adaptive design used, with justification (i.e. uncertainties to be addressed (effect, dose, population), resource allocation to be optimized across platform) and details of the pre-planned study adaptations and the statistical information informing the adaptations

Multiple interim analyses will be conducted by DMC/IDMC/DSMB to monitor efficacy and safety of the study interventions. Interim analyses will be conducted for the following:

for the full platform study / for each ISA separately at time points [X/Y/Z] / when [X/Y/Z] participants have been enrolled / when [X/Y/Z] participants reach the endpoint for decision making / when [X/Y/Z] participants have a least [X] weeks of follow up.

• at time points specified in the applicable ISA.

Based on the available interim data, the DMC/IDMC/DSMB will recommend continuing the study as planned, or propose adaptations to the ISA design and future ISAs as outlined in the DMC/IDMC/DSMB charter, including [X/Y/Z] (e.g. population enrichment, sample size adjustment, futility, success), or recommendation of additional study activities.

Additional interim analyses may be performed depending on recommendations from the DMC/IDMC/DSMB or based on the enrollment rate to guide further development and to support interactions with Health Authorities.

**Corrections for the adaptive design**

Exploratory with no corrections / Potentially Bayesian

Due to the exploratory nature of this platform study, no formal corrections in inference for the adaptive design will be considered. Simulations have been used to assess operating characteristics of the study design. The expected type-1 error /FWER is controlled at [X] for the considered adaptive design.

With correction within ISA

Appropriate statistical methodology will be implemented to control the type-1 error rate/FWER within ISAs. Adaptive group-sequential design methodology will be applied to control the type-1 error rate/FWER in presence of early stopping and other prospectively planned study design adaptations.

The [X] alpha-spending function approach will be used for sequential success monitoring, with expected number of and relative information at interim analyses as defined above. The exact timing and number of interim analyses will be utilized in calculation of the stage-wise alpha-spending.

Combination tests will be utilized to control the type-1 error /FWER in presence of study design adaptations. The calculation of combination weights is prospectively defined to depend on the relative information up to the time of the adaptive study design modification and will not depend on any unblinded efficacy estimates.

The family wise error rate will be controlled using an adaptive closed testing approach with preplanned intersection testing strategy as described in detail in the SAP.

Adjusted estimates, repeated confidence intervals and repeated p-values will be calculated, taking the prospectively planned study design adaptations into account.

The Statistical Analysis Plan and the DMC/IDMC/DSMB charter will describe the planned interim analyses in greater detail.

Simulations involved in determining interim stopping boundaries or decisions rules are defined in <SAP/DMC Charter >

<End of example text>

## Sample Size Determination

- In a platform study, different ISAs may target different populations and different interventions may have different decision rules and effect assumptions. The master protocol text on sample size may require generic language to allow specification within the ISAs, if required.
- The sample size for platform studies is not fixed and will depend also on operational aspects of the study design (e.g. time of entry of ISA). Simulations will help to evaluate different scenarios. In this case, a high-level summary of simulation results may be added to the protocol, referring for detailed results to an appendix. Alternatively, simplified calculations may allow one to approximate the required sample size.
- Potential stratification factors of importance, defining sub-populations of interest might need to be included. This is of relevance, if powering of subgroups is considered.
- Re-randomization to multiple ISAs may apply for some platform studies. This might have implications on the total sample size. Specifically, for platform studies in rare indications, this may limit the total number of participants to be enrolled into the platform. Corrections for re-randomization of participants might need to be added, if not included into simulations or ballpark sample size calculation.
- Simulations involved in determining sample size will be defined in <SAP/DMC Charter>
- State the expected number of participants to be screened, randomized/enrolled, and expected to be evaluable, if known and when applicable. Consistent with the estimand chosen (where applicable) state the assumptions for intercurrent events: frequency per intervention group and the assumed impact these may have on the effect size and variation. for each expected intervention in the platform study (e.g. if a large number of dropouts are expected over time, how this will impact the approximation of the expected sample size).
- When applicable, ensure this section clearly explains how screening failures and nonevaluable participants are defined.
- Provide justification of the maximum sample size in accordance with the primary and/or other relevant statistical analysis and study objectives/estimands.
- State if any historical or borrowing of data from other cohorts is being used and how amount of data to be borrowed will be determined (e.g. using Bayesian hierarchical models)
- Assumptions and methodology for determining the maximum sample size required for an intervention and the minimum required number of participants in the control required for making scientifically rigorous comparisons and for making the necessary references. State if borrowing from historical data or other study cohorts is allowed, and how the amount of borrowed data will be calculated (mainly Bayesian models). The justification for this should be provided with references. The expected sample size per intervention cohort for each intervention will be described in the ISA. In the Bayesian framework of most platform studies, any initial estimates are likely to be adapted over time. The sensitivity to these assumptions should be investigated by presenting different scenarios based on varying assumptions.
- The actual sample size reached in the study will rarely be exactly equal to the target sample size if anticipated event rate or assumptions differ. Therefore, please add the word approximately in the text when stating the target sample size. This ensures that the protocol covers the potential to slightly over- or underenroll.
- Include power calculations and level of significance to be used as appropriate and any posterior probabilities that are being used to demonstrate effectiveness of intervention, if applicable across the entire study, if details vary please describe in the ISA.
- If the sample size is not based on statistical considerations, as outlined above, provide a justification. An alternative to providing a statistical justification for the sample size is to state that the sample size is not based on statistical considerations and then discuss the statistical implications of the chosen sample size.

<Start of suggested text>

*The sample size of this platform study is driven by the total number of ISAs included into the study, the individual effect assumptions and the design specifics of the underlying ISAs.*

If data between ISAs to be shared, e.g. concurrent control

*Due to the sharing of concurrent data, operating characteristics of the platform study will depend on the time of inclusion of ISAs and the enrollment rate into the different ISAs.*

Clause adding flexibility on effect assumptions due to new information

*The available information on the endpoints used for decision making is expected to change over the duration of the platform study and will be considered in the confirmation of the required sample size of newly added ISAs.*

Endpoint to be used/Decision rule:

*Unless specified differently in the specific ISAs, decision making for an ISA in this platform study will be based on (pick one of the below)*

- *frequentist testing at one-sided significance level alpha of* [X]
- *"Bayesian inference", targeting the posterior probability of no effects below alpha*
- *the location of 1-sided 1-alpha-confidence/credibility intervals relative to targeted effects*
- *other*

*on the endpoint "x", as described in the section [X] of the master protocol.*

Approximate sample size text (Based on worst case scenario):

*Assuming a minimum clinically relevant effect of* [X] *and a randomization ratio of* [X] [*and an inflation of the control group size per ISA by [X] due to the use of concurrent control data*], *up to* [X] *subjects will need to be evaluable per ISA to assert a* (pick one of the below)

- *probability of success of at least [X] for the ISA*
- *probability of inconclusive results of at most [X] for the ISA.*

A total sample size for the platform study of [X] *evaluable participants will allow evaluation of* [X] *ISAs under these assumed common effect assumptions.*

*Based on the common effect assumptions and assuming the sample size of [X] per ISA and a total number of* [X] *ISAs, the probability to detect at least one intervention as effective is:* [X].

Simulation guided sample size text

*The required sample size of this platform study has been estimated based on clinical study simulations. Given the simulation results, at most* [X] participants *are required to be randomized for the evaluation of* [X] *ISAs. A total sample size of* [X] *is expected to be required for the platform study. Sample size and operation characteristics of the platform study will depend on the actual entry times of ISAs, enrollment into the ISAs and on the treatment effects. The simulation report summarizes operating characteristics of the study design under various scenarios.*

No sample size text – defer to ISA

*The required sample size of the platform study will be driven by the design and the objectives of the specific ISAs and the predicted performance of the planned analyses on the endpoints of interest, including effect assumptions for the ISAs.*

*Details on the sample size calculation will be provided in the respective intervention cohorts.*

If subpopulations of special interest exist / Enrichment

*The following stratification factors will be considered in the randomization:*

*To allow for testing of subgroup effects in stratum* [X], *at least* [X]% *of the enrolled participants per ISA shall be enrolled from stratum* [X]. *Any enrichment for a specific intervention will be described in this section of the ISA*.

Corrections for evaluable participants/ re-randomizations

*Approximately* [X] *participants will be randomly assigned to each ISA such that approximately* [X] evaluable participants will complete the study.

*Study participants may be eligible of sequentially randomization into other open ISAs, upon completion of previous ISAs. Assuming that [X]% of participants will be re-randomized to multiple ISAs, the total number of required participants for the platform is* [*X*].

<End of suggested text>

# Supporting Documentation and Operational Considerations

Information that is too lengthy and could detract from the reader’s comprehension if included in the body of the protocol should be included in an appendix.

The order of the sections is determined by the order in which they are first referenced in the protocol text.

Modify, delete, or add sections as needed.

## Appendix 1: Regulatory, Ethical, and Study Oversight Considerations

<Start of common text>

### Regulatory and Ethical Considerations

The text below should be modified as appropriate depending on the jurisdiction of the study sites, in consultation with Health Authority guidance.

- This study will be conducted in accordance with the protocol and with the following:
  - Consensus ethical principles derived from international guidelines including the Declaration of Helsinki and Council for International Organizations of Medical Sciences (CIOMS) international ethical guidelines
  - Applicable ICH Good Clinical Practice (GCP) guidelines [such the Declaration of Taipei, General Data Protection Regulation (GDPR), etc.’]
  - Applicable laws and regulations
- The protocol, protocol amendments, ICF, investigator’s brochure, [IDFU], and other relevant documents (eg, advertisements) must be submitted to an IRB/IEC by the investigator and reviewed and approved by the IRB/IEC before the study is initiated.
- Any amendments to the protocol will require IRB/IEC approval before implementation of changes made to the study design, except for changes necessary to eliminate an immediate hazard to study participants.
- Protocols and any substantial amendments to the protocol will require health authority approval prior to initiation except for changes necessary to eliminate an immediate hazard to study participants.
- The investigator will be responsible for the following:
  - Providing written summaries of the status of the study to the IRB/IEC in accordance with the requirements, policies, and procedures established by the IRB/IEC (the sponsor and/or study conduct organization will provide updates on the study as a whole, to which the Investigator should add center-specific information). (Note: Providing written summaries of the status of the study to the IRB/IEC annually is not required under CTR. See also CTR Q&A 1.5 on Eudralex Volume 10: https://ec.europa.eu/health/system/files/2022- 02/regulation5362014_qa_en_1.pdf)
  - Notifying the responsible body such as IRB/IEC of SAEs or other significant safety findings as required by IRB/IEC procedures (the sponsor or study conduct organization will provide notifications about events across the study, while the Investigator may need to provide additional center-specific information). (Note: There are no legal requirements in EU for notifying the IRB/IEC of SAEs.)
  - Providing oversight of the conduct of the study at the site and adherence to requirements of 21 CFR, ICH guidelines, the IRB/IEC, European regulation 536/2014 for clinical studies (if applicable), European Medical Device Regulation 2017/745 for clinical device research (if applicable), and all other applicable local regulations.
  - As applicable, according to requirements of the Regulation No536/2014 of the European Parliament and the Council of the European Union, the Sponsor will be responsible for obtaining approval from the Competent Authorities of the EU Member States and/or Ethics Committees, as appropriate, for any amendments to the clinical trial that are deemed as “substantial” (ie, changes which are likely to have a significant impact on the safety or physical or mental integrity of the clinical trial participants or on the scientific value of the trial) prior to their implementation.
  - According to the Regulation No 536/2014 of the European Parliament and the Council of the European Union, and as specified by the applicable regulatory requirements in non-EU/EEA countries, the clinical study Sponsor, needs to report to the concerned regulatory agency/ies serious breaches without undue delay but not later than 7 calendar days of becoming aware of that breach. A serious breach is defined as a deviation of the version of the protocol applicable at the time of the breach or the applicable clinical trial regulation that is likely to affect to a significant degree the safety and rights of a subject or the reliability and robustness of the data generated in the clinical trial.
  - The Sponsor shall ensure that all parties involved in the conduct of the clinical study promptly report any events that might meet the definition of a serious breach.
  - Therefore, Investigators shall within 48h after being aware of a deviation that might meet the definition of a serious breach, report to the Sponsor any suspected serious breach to enable the Sponsor to carry out the required assessment and notify the regulatory agency/ies in the event of a confirmed serious breach. To that extent, the principal Investigator must have a process in place to ensure that the site staff or service providers engaged by the principal Investigator/institution are able to identify the occurrence of a (suspected) serious breach and that a (suspected) serious breach is promptly reported to the Sponsor through the contacts (e-mail address or telephone number) provided by the Sponsor."

<End of common text>

### Financial Disclosure

IP financial disclosures will be done at the ISA level.

Include text related to financial disclosure if not included in another document.

<Start of suggested text>

[Investigators and subinvestigators will provide the sponsor with sufficient, accurate financial information as requested to allow the sponsor to submit complete and accurate financial certification or disclosure statements to the appropriate regulatory authorities. Investigators are responsible for providing information on financial interests during the course of the study and for 1 year after completion of the study.]

<End of suggested text>

<Start of common text>

CTR requires Declaration of Interest to be completed by all Investigators and to be submitted to Member State (part II CTA).

<End of common text>

### Informed Consent Process

Include the primary ethical concerns of this study that are applicable across the platform study and if known, within interventions. If the latter is not possible, ethical concerns should be described in the applicable ISA. Consider the key elements of the informed consent process, including any special concerns and how addressed (e.g., assent, capacity, legally acceptable representative).

<Start of common text>

- The investigator or his/her representative will explain the nature of the platform study, including the risks and benefits, to the participant [or their legally authorized representative [defined as [X]] and answer all questions regarding the study. The Declaration of Helsinki does not only require that the information is provided, but also that the investigator needs to make sure that the potential subject has understood the information (Art. 26).
- Participants must be informed that their participation is voluntary. Participants [or their legally authorized representatives] will be required to sign a statement of informed consent that meets the requirements of 21 CFR 50, CTR, local regulations, ICH guidelines, privacy and data protection (including GDPR) requirements, where applicable, and the IRB/IEC or study center.
- The medical record must include a statement that written informed consent was obtained before the participant was enrolled in the platform study and the date the written consent was obtained. The authorized person obtaining the informed consent must also sign the ICF.
- Any secondary use of participant data for additional research outside the scope of the platform study (clinical data, biological samples, images, biopsies, etc.) requires disclosure of the purpose of the research and additional consent of the participant.
- Participants must be reconsented to the most current version of the ICF(s) during their participation in the platform study unless the changes from the previous version are solely editorial in nature.
- A copy of the ICF(s) must be provided to the participant [or their legally authorized representative].

<End of common text>

If participants can be rescreened, add the text to state whether the participant needs to sign a new ICF for the platform study and intervention-specific rescreening.

<Start of suggested text>

[A participant who is rescreened is not required to sign another ICF if the rescreening occurs within (X) days from the previous ICF signature date.]

OR

[Participants who are rescreened are required to sign a new ICF.]

If participants will be asked to consent to optional exploratory research using the remainder of mandatory samples, include text that addresses the use of remaining mandatory samples for optional exploratory research.

[The ICF will contain a separate section that addresses the use of remaining mandatory samples for optional exploratory research. The investigator or authorized designee will explain to each participant the objectives of the exploratory research. Participants will be told that they are free to refuse to participate and may withdraw their consent at any time and for any reason during the storage period. A separate signature will be required to document a participant’s agreement to allow any remaining specimens to be used for exploratory research. Participants who decline to participate in this optional research will not provide this separate signature.]

<End of suggested text>

### Data Protection

Include all measures to be taken to comply with the applicable rules on protection of personal data and any relevant information on measures to be taken in case of a data security breach.

<Start of common text>

- Participants will be assigned a unique identifier by the sponsor. Any participant records or datasets that are transferred to the sponsor will contain the identifier only; participant names or any information which would make the participant identifiable will not be transferred.
- The participant must be informed that his/her personal platform study-related data will be used by the sponsor in accordance with local data protection law. The level of disclosure must also be explained to the participant who will be required to give consent for their data to be used as described in the informed consent
- The participant must be informed that his/her medical records may be examined by Clinical Quality Assurance auditors or other authorized personnel appointed by the sponsor, by appropriate IRB/IEC members, and by inspectors from regulatory authorities.

For studies conducted in the EU: To address EU-CTR, consider including the suggested bullets about the Sponsor/study-site responsibilities and use of secured information technology systems.

<Start of suggested text>

**Protection of participant data**

- The contract between Sponsor, Investigators, and study sites specifies responsibilities of the parties related data protection, including handling of data security breaches and respective communication and cooperation of the parties. Accordingly, the Investigator and the institution will promptly notify the Sponsor about any data security breaches and detail in the notification the nature of the breach, the categories (eg, Sponsor’s personnel, study participants or their relatives, healthcare professionals, etc.), the approximate number of subjects concerned, the type and approximate number of data records concerned and the likely consequences of the breach. The institution and/or Investigator will investigate the causes of the data security breach and take actions to minimize the effects of said breach. The institution and/or Investigator will record all information relating to the breach, including the results of their own investigations and investigations by authorities, as applicable, and will take all measures as necessary to prevent future data security breaches.
- Information technology systems used to collect, process, and store study-related data are secured by technical and organizational security measures designed to protect such data against accidental or unlawful loss, alteration, or unauthorized disclosure or access.

<End of suggested text>

- Participants must be informed that their study-related data will be used for the whole “drug development program”, ie, for this study as well as for the following steps necessary for the development of the investigational product, including to support negotiations with payers and publication of results.

<End of common text>

### Committees Structure

Briefly describe the administrative structure for the platform study (eg, internal review committee/internal review forum, central laboratories, steering committee, expert advisory committee, data monitoring committee or data safety monitoring board, contract research organization). Note that specific details are not required.

If a data monitoring committee is used, please include a section discussing any procedures relating to its operations (eg, charter, composition and schedule of meetings, etc). Consider the need for a closely monitored setting following initial dosing with the intervention.

#### [Early Safety Data Review AND/OR Committee]

Choose from the options provided and modify as required. Enter additional details as appropriate for the platform study design, eg, dose ranging. If the study does not include an early safety data review then only include the final bullet and delete the preceding ones. This last bullet should be deleted if an early safety data review is part of the platform study.

<Start of suggested text>

- Participant safety will be continuously monitored by the [sponsor’s internal or external] [safety review or insert others] committee, which includes safety signal detection at any time in an intervention cohort or during the platform study.
- In addition, an early aggregated safety data review will be performed, the goal of which is to allow for a cautious, stepwise approach to [intervention] administration. An initial safety review for this study is planned for the first [X participants/X% of participants] who are dosed and have provided safety data for [X] days after administration of Dose [X].
- All safety data collected will be summarized and reviewed by the [sponsor’s internal/external safety review or other committee] for agreement of next steps.
- In particular, data will be reviewed by the sponsor for identification of the following events that would potentially contribute to a requirement to [pause/stop] an intervention cohort or the platform study.
  - [Any deaths, regardless of causality]
  - [Any vaccine-related SAEs]
  - [Grade 3 fever reported in more than 2 participants (see table in Appendix [3/7]]
  - [Other]
- [Enrollment will be paused during the review]. If a [pausing/stopping] rule is met, a decision will be made, based on the review, as to whether enrollment in an intervention cohort or the platform study will be allowed to resume.
- Case unblinding may be performed for above reviews if necessary.

<End of suggested text>

### Dissemination of Clinical Study Data

Describe sponsor -specific or consortium-specific policy on provision of study results.

For studies conducted in the EU under Regulations EU 536/2014: Align on accountability of publishing results from within study and the timing of when this should be done across each of the interventions being evaluated.

Include how the following will be handled.

- Disclosure of CSRs, periodic safety reports, and clinical study summary reports after review by regulatory authorities. This includes access to CSRs from studies with negative outcomes and from terminated development programs.
- The posting of company-sponsored study information and tabular study results on the US National Institutes of Health’s website [www.clinicaltrials.gov](http://www.clinicaltrials.gov) and other publicly accessible sites
- Publication planning and other activities related to nonpromotional, peer-reviewed publications, to ensure the scientific integrity and credibility of publication activities performed by or on behalf of the company. The granting of access to analyzable datasets from clinical studies through a secure system, following an independent assessment of the scientific merit of a rigorously defined research question from a third party

### Data Quality Assurance

<Start of common text>

- Start-up meeting for all research coordinators and investigators will be held prior to platform study commencement to ensure consistency in procedures.
- All participant data relating to the study will be recorded on printed or electronic CRFs unless transmitted to the sponsor or designee electronically (eg, laboratory data). The investigator is responsible for verifying that data entries are accurate and correct by physically or electronically signing the CRF.
- Guidance on completion of CRFs will be provided in [specify location of information].
- The investigator must permit study-related monitoring, audits, IRB/IEC review, and regulatory agency inspections and provide direct access to source data documents.
- Quality tolerance limits (QTLs) will be predefined in the [state location(s)] to identify systematic issues that can impact participant safety and/or reliability of study results. These predefined parameters will be monitored during the study, and important deviations from the QTLs and remedial actions taken will be summarized in the clinical study report.
- Monitoring details describing strategy, including definition of study critical data items and processes (eg, risk-based initiatives in operations and quality such as risk management and mitigation strategies and analytical risk-based monitoring), methods, responsibilities, and requirements, including handling of noncompliance issues and monitoring techniques (central, remote, or on-site monitoring) are provided in the [monitoring plan] [contracts].
- The sponsor or designee is responsible for the data management of this study, including quality checking of the data.
- The sponsor assumes accountability for actions delegated to other individuals (eg, contract research organizations).
- Records and documents, including signed ICFs, pertaining to the conduct of this study must be retained by the investigator for [X] years after study completion unless local regulations or institutional policies require a longer retention period. No records may be destroyed during the retention period without the written approval of the sponsor. No records may be transferred to another location or party without written notification to the sponsor.

<End of common text>

### Source Documents

<Start of common text>

Describe procedures for the identification of data to be recorded directly on the CRF considered as source data.

- Source documents provide evidence for the existence of the participant and substantiate the integrity of the data collected. Source documents are filed at the investigator’s site.
- Data reported on the CRF or entered in the eCRF that are transcribed from source documents must be consistent with the source documents or the discrepancies must be explained. The investigator may need to request previous medical records or transfer records, depending on the study. Also, current medical records must be available.
- Definition of what constitutes source data and its origin can be found in [eg, source data acknowledgment or monitoring guidelines].
- The investigator must maintain accurate documentation (source data) that supports the information entered in the CRF.
- Study monitors will perform ongoing source data verification to confirm that data entered into the CRF by authorized site personnel are accurate, complete, and verifiable from source documents; that the safety and rights of participants are being protected; and that the study is being conducted in accordance with the currently approved protocol and any other study agreements, ICH GCP, and all applicable regulatory requirements.

<End of common text>

### Study and Site Start and Closure

**First Act of Recruitment**

<Start of suggested text>

The study start date is the date on which the clinical study will be open for recruitment of participants.

The first act of recruitment is the [first site open] OR [insert other] is considered the first act of recruitment] and will be the study start date.

<End of suggested text>

**Study/Site Termination**

<Start of common text>

The sponsor or designee reserves the right to close the study site or terminate the study at any time for any reason at the sole discretion of the sponsor. Study sites will be closed upon study completion. A study site is considered closed when all required documents and study supplies have been collected and a study-site closure visit has been performed.

The investigator may initiate study-site closure at any time, provided there is reasonable cause and sufficient notice is given in advance of the intended termination.

Reasons for the early closure of a study site by the sponsor or investigator may include but are not limited to:

For study termination:

- Discontinuation of further study intervention development

For site termination:

- Failure of the investigator to comply with the protocol, the requirements of the IRB/IEC or local health authorities, the sponsor’s procedures, or GCP guidelines
- Inadequate or no recruitment (evaluated after a reasonable amount of time) of participants by the investigator
- Total number of participants included earlier than expected

If the study is prematurely terminated or suspended, the sponsor shall promptly inform the investigators, the IECs/IRBs, the regulatory authorities, and any contract research organization(s) used in the study of the reason for termination or suspension, as specified by the applicable regulatory requirements. The investigator shall promptly inform the participant and should assure appropriate participant therapy and/or follow-up.

<End of common text>

### Publication Policy

The following information is required by ICH to be in the protocol if not addressed in another document. If addressed in site contracts, this section can be deleted.

<Start of common text>

- Publication of the results of this study will be governed by Publication/Dissemination Committee. Any presentation, abstract, or manuscript will be made available for review prior to submission.
- The results of this study may be published or presented at scientific meetings. If this is foreseen, the investigator agrees to submit all manuscripts or abstracts to the sponsor before submission. This allows the sponsor to protect proprietary information and to provide comments.
- The sponsor will comply with the requirements for publication of study results. In accordance with standard editorial and ethical practice, the sponsor will generally support publication of multicenter studies only in their entirety and not as individual site data. In this case, a coordinating investigator will be designated by mutual agreement.
- Authorship will be determined by mutual agreement and in line with International Committee of Medical Journal Editors authorship requirements.

<End of common text>

## Appendix 2: Clinical Laboratory Tests

- An example table is provided for listing laboratory tests. Modify as required for the study.
- Consider adding Level 3 headings for laboratory assessments for safety, and immunogenicity or biomarkers if relevant
- Abbreviations appearing in these tables do not need to be repeated in the abbreviations list.
- If any of the following tests are for screening purposes only, please specify.
- Indicate if the participants must be fasting (length of time) or nonfasting.

Procedure Notes:

- Hepatitis B and Hepatitis C screening:
  - For Phase 1 and Phase 2 studies, hepatitis B surface antigen (HBsAg) and hepatitis C virus (HCV antibody) testing may be required. For potent immunosuppressive agents, participants should also undergo testing for hepatitis B core antibody (HBcAb).
  - For Phase 3 studies, hepatitis testing may not be required unless immunosuppressive agents will be administered. Refer to exclusion criteria for additional guidance.

<Start of common text>

- The tests detailed in Table [X] will be performed [by the central laboratory] [by the local laboratory].
- [Local laboratory results are only required in the event that the central laboratory results are not available in time for either study intervention administration and/or response evaluation. If a local sample is required, it is important that the sample for central analysis is obtained at the same time. Additionally, if the local laboratory results are used to make either a study intervention decision or response evaluation, the results must be recorded.]
- [Intervention Specific requirements for inclusion or exclusion of participants are detailed in each ISA.]
- Additional tests may be performed at any time during the study as determined necessary by the investigator or required by local regulations.

Table X: Protocol-required Laboratory Tests

| Laboratory Tests | Parameters | | | | | |
| --- | --- | --- | --- | --- | --- | --- |
| Hematology | Platelet count | | RBC indices:  Mean corpuscular volume (MCV)  Mean corpuscular hemoglobin (MCH)  %Reticulocytes | | White blood cell (WBC) count with differential:  Neutrophils  Lymphocytes  Monocytes  Eosinophils  Basophils | |
|  | Red blood cell (RBC) count | |  |  |  |  |
|  | Hemoglobin | |  |  |  |  |
|  | Hematocrit | |  |  |  |  |
| Clinical chemistry^1^ | Blood urea nitrogen (BUN) | Potassium | | Aspartate aminotransferase (AST)/serum glutamic-oxaloacetic transaminase (SGOT) | | Total and direct bilirubin |
|  | Creatinine | Sodium | | Alanine aminotransferase (ALT)/serum glutamic-pyruvic transaminase (SGPT) | | Total protein |
|  | Glucose [indicate if fasting or nonfasting] | Calcium | | Alkaline phosphatase^2^ | |  |
| Routine urinalysis | - Specific gravity - pH, glucose, protein, blood, ketones, [bilirubin, urobilinogen, nitrite, leukocyte esterase] by dipstick - Microscopic examination (if blood or protein is abnormal) | | | | | |
| Pregnancy testing | - Highly sensitive [serum or urine] human chorionic gonadotropin (hCG) pregnancy test (as needed for women of childbearing potential)^3^ | | | | | |
| Other screening tests | - Follicle-stimulating hormone and estradiol (as needed in women of nonchildbearing potential only) - [Serum or urine] [alcohol and drug screen (to include at minimum: amphetamines, barbiturates, cocaine, opiates, cannabinoids and benzodiazepines)] - [Serology [(HIV antibody, hepatitis B surface antigen [HBsAg], and hepatitis C virus antibody)] [or specify other tests] [if applicable]   If a central laboratory is being used and protocol-required additional local tests are needed, include the following:   - [All study-required laboratory tests will be performed by a central laboratory, with the exception of [list the exceptions]:   - [SPECIFY REQUIRED TEST(S)] | | | | | |
| NOTES:  1 Details of liver chemistry stopping criteria and required actions and follow-up are given in Section [7.1.1 Liver Chemistry Stopping Criteria] and Appendix [6: Liver Safety: Suggested Actions and Follow-up Assessments [and Study Intervention Rechallenge Guidelines]]. All events of ALT [or AST] ≥ 3 × upper limit of normal (ULN) and total bilirubin ≥ 2 × ULN (> 35% direct bilirubin) or ALT [or AST] ≥ 3 × ULN and international normalized ratio (INR) > 1.5 (if INR measured), which may indicate severe liver injury (possible Hy’s law), must be reported to [sponsor] in an expedited manner (excluding studies of hepatic impairment or cirrhosis).  2 If alkaline phosphatase is elevated, consider fractionating.  3 A woman is considered of childbearing potential (fertile) from the time of menarche until becoming postmenopausal unless permanently sterile. Local urine testing will be standard for the protocol unless serum testing is required by local regulation or IRB/IEC | | | | | | |

Investigators must document their review of each laboratory safety report.

<End of common text>

## Appendix 3: AEs and SAEs: Definitions and Procedures for Recording, Evaluating, Follow-up, and Reporting

The definitions and procedures in Appendix 3 should be used for AEs and SAEs which do **not** involve sponsor-provided medical devices used in this study (see Section 6.1.1 for the list of sponsor medical devices). For events during the study that **do** involve the device, refer to Appendix 7 for definitions and reporting requirements for medical device AEs, SAEs, incidents, and deficiencies.

<Start of common text>

### Definition of AE

| AE Definition |
| --- |
| - An AE is any untoward medical occurrence in a clinical study participant, temporally associated with the use of study intervention, whether or not considered related to the study intervention. - NOTE: An AE can therefore be any unfavorable and unintended sign (including an abnormal laboratory finding), symptom, or disease (new or exacerbated) temporally associated with the use of study intervention. |

Add these definitions of unsolicited and solicited AEs to Appendix 3 AEs and SAEs: Definitions and Procedures for Recording, Evaluating, Follow-up, and Reporting] at the end of Section 10.3.1 if relevant, eg, for vaccine and pediatric studies.

| **Definition of Unsolicited and Solicited AE** |
| --- |
| - An unsolicited adverse event is an adverse event that was not solicited using a participant diary and that is communicated by a [participant/participant’s parent(s)/legally authorized representative (LAR)(s)] who has signed the informed consent. Unsolicited AEs include serious and nonserious AEs. - Potential unsolicited AEs may be medically attended (ie, symptoms or illnesses requiring a hospitalization, emergency room visit, or visit to/by a healthcare provider). The [participants/participant’s parent(s)/LAR(s)] will be instructed to contact the site as soon as possible to report medically attended event(s), as well as any events that, though not medically attended, are of [participant/participant’s parent(s)/LAR(s)] concern. Detailed information about reported unsolicited AEs will be collected by qualified site personnel and documented in the participant’s records. - Unsolicited AEs that are not medically attended nor perceived as a concern by the [participant/participant’s parent(s)/LAR(s)] will be collected during an interview with the [participants/participant’s parent(s)/LAR(s)] and by review of available medical records at the next visit. - Solicited AEs are predefined local [at the injection site] and systemic events for which the participant is specifically questioned, and which are noted by the participant in their diary. |

| Events Meeting the AE Definition |
| --- |
| For efficacy studies, include the penultimate bullet, and for nonefficacy studies involving marketed products in established indications, include the final bullet.   - Any abnormal laboratory test results (hematology, clinical chemistry, or urinalysis) or other safety assessments (eg, ECG, radiological scans, vital signs measurements), including those that worsen from baseline, considered clinically significant in the medical and scientific judgment of the investigator (ie, not related to progression of underlying disease) - Exacerbation of a chronic or intermittent pre-existing condition including either an increase in frequency and/or intensity of the condition - New condition detected or diagnosed after study intervention administration even though it may have been present before the start of the study. (In such cases, the AE onset date should be recorded as the retrospective date of first symptom, NOT the date a medical diagnosis was confirmed). - Signs, symptoms, or the clinical sequelae of a suspected intervention-intervention interaction - Signs, symptoms, or the clinical sequelae of a suspected overdose of either study intervention or a concomitant medication. Overdose per se will not be reported as an AE/SAE unless it is an intentional overdose taken with possible suicidal/self-harming intent. Such overdoses should be reported regardless of sequelae. - [Lack of efficacy or failure of expected pharmacological action per se will not be reported as an AE or SAE. Such instances will be captured in the efficacy assessments. However, the signs, symptoms, and/or clinical sequelae resulting from lack of efficacy will be reported as AE or SAE if they fulfill the definition of an AE or SAE.] - [The signs, symptoms, and/or clinical sequelae resulting from lack of efficacy will be reported as an AE or SAE if they fulfill the definition of an AE or SAE. Lack of efficacy or failure of expected pharmacological action also constitutes an AE or SAE.] |

| Events NOT Meeting the AE Definition |
| --- |
| - Any clinically significant abnormal laboratory findings or other abnormal safety assessments that are associated with the underlying disease, unless judged by the investigator to be more severe than expected for the participant’s condition - The disease/disorder being studied or expected progression, signs, or symptoms of the disease/disorder being studied, unless more severe than expected for the participant’s condition - Medical or surgical procedure (eg, endoscopy, appendectomy): the condition that leads to the procedure is the AE - Individual signs/symptoms contributing to a medical diagnosis: record the medical diagnosis as the AE - Situations in which an untoward medical occurrence did not occur (social and/or convenience admission to a hospital) - Anticipated day-to-day fluctuations of pre-existing disease(s) or condition(s) present or detected at the start of the study that do not worsen |

### Definition of SAE

| An SAE is defined as any untoward medical occurrence that, at any dose, meets one or more of the criteria listed: |
| --- |
| 1. **Results in death** |
| 1. **Is life threatening**   The term *life threatening* in the definition of *serious* refers to an event in which the participant was at risk of death at the time of the event. It does not refer to an event, which hypothetically might have caused death, if it were more severe. |
| 1. **Requires inpatient hospitalization or prolongation of existing hospitalization**  - In general, hospitalization signifies that the participant has been admitted (usually involving at least an overnight stay) at the hospital or emergency ward for observation and/or treatment that would not have been appropriate in the physician’s office or outpatient setting. Complications that occur during hospitalization are AEs. If a complication prolongs hospitalization or fulfills any other serious criteria, the event is serious. When in doubt as to whether hospitalization occurred or was necessary, the AE should be considered serious. - Hospitalization for elective treatment of a pre-existing condition that did not worsen from baseline is not considered an AE. |
| 1. **Results in persistent or significant disability/incapacity**  - The term disability means a substantial disruption of a person’s ability to conduct normal life functions. - This definition is not intended to include experiences of relatively minor medical significance such as uncomplicated headache, nausea, vomiting, diarrhea, influenza, and accidental trauma (eg, sprained ankle) that may interfere with or prevent everyday life functions but do not constitute a substantial disruption. |
| 1. **Is a congenital anomaly/birth defect** |
| 1. [Is a suspected transmission of any infectious agent via an authorized medicinal product] |
| 1. **Other situations:**  - Medical or scientific judgment should be exercised by the investigator in deciding whether SAE reporting is appropriate in other situations such as significant medical events that may jeopardize the participant or may require medical or surgical intervention to prevent one of the other outcomes listed in the above definition. These events should usually be considered serious.   - Examples of such events include invasive or malignant cancers, intensive treatment for allergic bronchospasm, blood dyscrasias, convulsions or development of intervention dependency or intervention abuse. |

Add other SAEs relevant per protocol/study intervention. Obtain agreement with the pharmacovigilance group on any protocol or project-specific SAEs. Such SAEs should be specified in the other situations part of the SAE definition.

Examples include:

- Grade 4 laboratory abnormalities
- [specify event] – see Section [X] for definition
- [specify event] leading to permanent discontinuation of study intervention

### Recording and Follow-Up of AE and/or SAE

| AE and SAE Recording |
| --- |
| - When an AE/SAE occurs, it is the responsibility of the investigator to review all documentation (eg, hospital progress notes, laboratory reports, and diagnostics reports) related to the event. - The investigator will then record all relevant AE/SAE information. - It is **not** acceptable for the investigator to send photocopies of the participant’s medical records to [X] in lieu of completion of the [X]/required form. - There may be instances when copies of medical records for certain cases are requested by [X]. In this case, all participant identifiers, with the exception of the participant number, will be redacted on the copies of the medical records before submission to [X]. - The investigator will attempt to establish a diagnosis of the event based on signs, symptoms, and/or other clinical information. Whenever possible, the diagnosis (not the individual signs/symptoms) will be documented as the AE/SAE. |
| Assessment of Intensity |
| The investigator will make an assessment of intensity for each AE and SAE reported during the study and assign it to one of the following categories:   - Mild: Asymptomatic or mild symptoms; clinical or diagnostic observations only; intervention not indicated. - Moderate: Minimal, local or noninvasive intervention indicated; limiting age-appropriate instrumental Activities of Daily Living (ADL). Instrumental ADL refers to preparing meals, shopping for groceries or clothes, using the telephone, managing money, etc. - Severe: Severe or medically significant but not immediately life-threatening; hospitalization or prolongation of hospitalization indicated; disabling, limiting self care ADL. Self care ADL refers to bathing, dressing and undressing, feeding self, using the toilet, taking medications, and not bedridden.   Other measures to evaluate AEs and SAEs may be used (eg, National Cancer Institute Common Terminology Criteria for Adverse Events [NCI-CTCAE]). |

| Assessment of Causality |
| --- |
| - The investigator is obligated to assess the relationship between study intervention and each occurrence of each AE/SAE. The investigator will use clinical judgment to determine the relationship. - A *reasonable possibility* of a relationship conveys that there are facts, evidence, and/or arguments to suggest a causal relationship, rather than a relationship cannot be ruled out. - Alternative causes, such as underlying disease(s), concomitant therapy, and other risk factors, as well as the temporal relationship of the event to study intervention administration, will be considered and investigated. - The investigator will also consult the investigator’s brochure (IB) and/or product information, for marketed products, in his/her assessment. - For each AE/SAE, the investigator **must** document in the medical notes that the participant has reviewed the AE/SAE and has provided an assessment of causality. - There may be situations in which an SAE has occurred and the investigator has minimal information to include in the initial report to [X]. However, it is very important that the investigator always make an assessment of causality for every event before the initial transmission of the SAE data to [X]. - The investigator may change his/her opinion of causality in light of follow-up information and send an SAE follow-up report with the updated causality assessment. - The causality assessment is one of the criteria used when determining regulatory reporting requirements. |

| Follow-up of AEs and SAEs |
| --- |
| - The investigator is obligated to perform or arrange for the conduct of supplemental measurements and/or evaluations as medically indicated or as requested by [X] to elucidate the nature and/or causality of the AE or SAE as fully as possible. This may include additional laboratory tests or investigations, histopathological examinations, or consultation with other health care professionals. - [If a participant dies during participation in the study or during a recognized follow-up period, the investigator will provide [X] with a copy of any postmortem findings including histopathology.]   Suggested bullet in variable blue text may not be required for studies where death is an endpoint.   - New or updated information will be recorded in the originally submitted documents. - The investigator will submit any updated SAE data to [X] within 24 hours of receipt of the information. |

### Reporting of SAEs

| SAE Reporting to [X] via an Electronic Data Collection Tool |
| --- |
| - The primary mechanism for reporting an SAE to [X] will be the electronic data collection tool. - If the electronic system is unavailable, then the site will use the paper SAE data collection tool (see next section) to report the event within 24 hours. - The site will enter the SAE data into the electronic system as soon as it becomes available. - After the study is completed at a given site, the electronic data collection tool will be taken offline to prevent the entry of new data or changes to existing data. - If a site receives a report of a new SAE from a study participant or receives updated data on a previously reported SAE after the electronic data collection tool has been taken offline, then the site can report this information on a paper SAE form (see next section) or to the [X/medical monitor/SAE coordinator] by telephone. - Contacts for SAE reporting can be found in [X]. |

| SAE Reporting to [X] via Paper Data Collection Tool | |
| --- | --- |
| - [Facsimile transmission of the SAE paper data collection tool is the preferred method to transmit this information to the [X/medical monitor or the SAE coordinator]. - [In rare circumstances and in the absence of facsimile equipment, notification by telephone is acceptable with a copy of the SAE data collection tool sent by overnight mail or courier service.] - Initial notification via telephone does not replace the need for the investigator to complete and sign the SAE data collection tool within the designated reporting timeframes. - Contacts for SAE reporting can be found in [X]. |  |

<End of common text>

## Appendix 4: Contraceptive and Barrier Guidance

Content for this appendix may be in the master protocol or the ISA as applicable. If this content is in the ISA, add the statement under common text below.

Delete appendix if not required.

Insert content for this appendix from the participant libraries as appropriate based upon the decision trees in Section 5.1.

<Start of common text>

Refer to the applicable ISA for contraception and barrier guidance.

<End of common text>

### Definitions

See participant libraries for common text to include here.

<

### Contraception Guidance

See participant libraries for common text to include here.

## Appendix 5: Genetics

Delete appendix if not required.

<Start of suggested text>

The following analyses may be conducted in alignment with HA and other regulations in the study site jurisdictions, and with participant consent.

Use/Analysis of DNA

- Genetic variation may impact a participant’s response to study intervention, susceptibility to, and severity and progression of disease. Variable response to study intervention may be due to genetic determinants that impact intervention absorption, distribution, metabolism, and excretion; mechanism of action of the intervention; disease etiology; and/or molecular subtype of the disease being treated. Therefore, where local regulations and IRB/IEC allow, a [blood/saliva] sample will be collected for DNA analysis from consenting participants.
- DNA samples will be used for research related to [study intervention] or [indication] and related diseases. They may also be used to develop tests/assays, including diagnostic tests related to [study intervention and/or interventions of this drug class] and [indication]. Genetic research may consist of the analysis of one or more candidate genes or the analysis of genetic markers throughout the genome [or analysis of the entire genome] (as appropriate).
- [DNA samples will be analyzed for [describe planned analyses]. [Additional] analyses may be conducted if it is hypothesized that this may help further understand the clinical data.]
- The samples may be analyzed as part of a multistudy assessment of genetic factors involved in the response to [study intervention] or study interventions of this class to understand the study disease or related conditions.
- The results of genetic analyses may be reported in the clinical study report (CSR) or in a separate study summary.
- The sponsor will store the DNA samples in a secure storage space with adequate measures to protect confidentiality.
- The samples will be retained while research on [study intervention or study interventions of this class or indication] continues but no longer than [X] years or other period as per local requirements.

<End of suggested text>

## Appendix 6: Liver Safety: Suggested Actions and Follow-up Assessments [and Study Intervention Restart/Rechallenge Guidelines]

This is disease/population specific information that should be added at the time of protocol development.

Delete appendix if not required.

See participant libraries for suggested common text.

## Appendix 7: AEs, ADEs, SAEs, SADEs, USADEs and Device Deficiencies: Definitions and Procedures for Recording, Evaluating, Follow-up, and Reporting in Medical Device Studies

Delete appendix if not required.

This appendix is required for a study in which a sponsor medical device is provided for use in the study (ie, there are medical devices listed in Section 6.1.1 that are manufactured by the sponsor or by a third party for the sponsor). If Section 6.1.1 includes only nonsponsor medical devices or is not applicable, then this appendix is not needed.

<Start of common text>

- The definitions and procedures detailed in this appendix are in accordance with ISO 14155 and the European Medical Device Regulation (MDR) 2017/745 for clinical device research (if applicable).
- Both the investigator and the sponsor will comply with all local reporting requirements for medical devices.
- The detection and documentation procedures described in this protocol apply to all sponsor medical devices provided for use in the study. See Section 6.1.1 for the list of sponsor medical devices.

### Definition of Medical Device AE and ADE

| Medical Device AE and ADE Definition |
| --- |
| - A medical device AE is any untoward medical occurrence in a clinical study participant, users, or other persons, temporally associated with the use of study intervention, whether or not considered related to the investigational medical device. An AE can therefore be any unfavorable and unintended sign (including an abnormal laboratory finding), symptom, or disease (new or exacerbated) temporally associated with the use of an investigational medical device. This definition includes events related to the investigational medical device or comparator and events related to the procedures involved except for events in users or other persons, which only include events related to investigational devices. - An adverse device effect (ADE) is defined as an AE related to the use of an investigational medical device. This definition includes any AE resulting from insufficient or inadequate instructions for use, deployment, implantation, installation, or operation, or any malfunction of the investigational medical device as well as any event resulting from use error or from intentional misuse of the investigational medical device. |

### Definition of Medical Device SAE, SADE and USADE

| A Medical Device SAE is an any serious adverse event that: |
| --- |
| 1. Led to death |
| 1. Led to serious deterioration in the health of the participant, that either resulted in:  - A life-threatening illness or injury. The term “life-threatening” in the definition of “serious” refers to an event in which the participant was at risk of death at the time of the event. It does not refer to an event, which hypothetically might have caused death if it were more severe. - A permanent impairment of a body structure or a body function. - Inpatient or prolonged hospitalization. Planned hospitalization for a pre-existing condition, or a procedure required by the protocol, without serious deterioration in health, is not considered an SAE. - Medical or surgical intervention to prevent life-threatening illness or injury or permanent impairment to a body structure or a body function. - Chronic disease (MDR 2017/745). |
| 1. Led to fetal distress, fetal death, or a congenital abnormality or birth defect |
| 1. [Is a suspected transmission of any infectious agent via a medicinal product] |
| SADE definition |
| - A SADE is defined as an adverse device effect that has resulted in any of the consequences characteristic of an SAE. - Any device deficiency that might have led to an SAE if appropriate action had not been taken, intervention had not occurred, or circumstances had been less fortunate. |
| **Unanticipated SADE (USADE) definition** |
| - An USADE (also identified as UADE in US Regulations 21 CFR 813.3), is defined as a serious adverse device effect that by its nature, incidence, severity, or outcome has not been identified in the current version of the risk analysis report (see Section 2.3). |

### Definition of Device Deficiency

| **Device Deficiency Definition** |
| --- |
| - A device deficiency is an inadequacy of a medical device with respect to its identity, quality, durability, reliability, safety, or performance. Device deficiencies include malfunctions, use errors, and inadequacy of the information supplied by the manufacturer. |

### Recording and Follow-Up of AE and/or SAE and Device Deficiencies

| AE, SAE, and Device Deficiency Recording |
| --- |
| - When an AE/SAE/device deficiency occurs, it is the responsibility of the investigator to review all documentation (eg, hospital progress notes, laboratory reports, and diagnostics reports) related to the event. - The investigator will then record all relevant AE/SAE/device deficiency information in the participant’s medical records, in accordance with the investigator’s normal clinical practice and on the appropriate form. - It is **not** acceptable for the investigator to send photocopies of the participant’s medical records to [X] in lieu of completion of the [X]/AE/SAE/device deficiency form. - There may be instances when copies of medical records for certain cases are requested by [X]. In this case, all participant identifiers, with the exception of the participant number, will be redacted on the copies of the medical records before submission to [X]. - The investigator will attempt to establish a diagnosis of the event based on signs, symptoms, and/or other clinical information. Whenever possible, the diagnosis (not the individual signs/symptoms) will be documented as the AE/SAE. - For device deficiencies, it is very important that the investigator describes any corrective or remedial actions taken to prevent recurrence of the deficiency.   - A remedial action is any action other than routine maintenance or servicing of a medical device where such action is necessary to prevent recurrence of a device deficiency. This includes any amendment to the device design to prevent recurrence. |
| Assessment of Intensity |
| The investigator will make an assessment of intensity for each AE/SAE/device deficiency reported during the study and assign it to one of the following categories:   - Mild: Asymptomatic or mild symptoms; clinical or diagnostic observations only; intervention not indicated. - Moderate: Minimal, local or noninvasive intervention indicated; limiting age-appropriate instrumental Activities of Daily Living (ADL). Instrumental ADL refers to preparing meals, shopping for groceries or clothes, using the telephone, managing money, etc. - Severe: Severe or medically significant but not immediately life-threatening; hospitalization or prolongation of hospitalization indicated; disabling, limiting self care ADL. Self care ADL refers to bathing, dressing and undressing, feeding self, using the toilet, taking medications, and not bedridden.   Other measures to evaluate AEs and SAEs may be used (eg, National Cancer Institute Common Terminology Criteria for Adverse Events [NCI-CTCAE]). |

| Assessment of Causality |
| --- |
| - The investigator is obligated to assess the relationship between study intervention and each occurrence of each AE/SAE/device deficiency. The investigator will use clinical judgment to determine the relationship. - A *reasonable possibility* of a relationship conveys that there are facts, evidence, and/or arguments to suggest a causal relationship, rather than a relationship, cannot be ruled out. - Alternative causes, such as underlying disease(s), concomitant therapy, and other risk factors, as well as the temporal relationship of the event to study intervention administration will be considered and investigated. - The investigator will also consult the [investigator’s brochure (IB) and/or IDFU or product information, for marketed products] in his/her assessment. - For each AE/SAE/device deficiency, the investigator must document in the medical notes that he/she has reviewed the AE/SAE/device deficiency and has provided an assessment of causality. - There may be situations in which an SAE has occurred and the investigator has minimal information to include in the initial report to [X]. However, it is very important that the investigator always make an assessment of causality for every event before the initial transmission of the SAE data to [X]. - The investigator may change his/her opinion of causality in light of follow-up information and send an SAE follow-up report with the updated causality assessment. - The causality assessment is one of the criteria used when determining regulatory reporting requirements. |

| Follow-up of AE/SAE/device deficiency |
| --- |
| - The investigator is obligated to perform or arrange for the conduct of supplemental measurements and/or evaluations as medically indicated or as requested by [X] to elucidate the nature and/or causality of the AE/SAE/device deficiency as fully as possible. This may include additional laboratory tests or investigations, histopathological examinations, or consultation with other health care professionals. - [If a participant dies during participation in the study or during a recognized follow-up period, the investigator will provide [X] with a copy of any post‑mortem findings including histopathology.] - Suggested bullet in variable blue text may not be required for studies where death is an endpoint. - New or updated information will be recorded in the originally completed form. - The investigator will submit any updated SAE data to [X] within 24 hours of receipt of the information. |

### Reporting of SAEs

| SAE Reporting to [X] via an Electronic Data Collection Tool |
| --- |
| - The primary mechanism for reporting an SAE to [X] will be the electronic data collection tool. - If the electronic system is unavailable, then the site will use the paper SAE data collection tool (see next table) to report the event within 24 hours. - The site will enter the SAE data into the electronic system as soon as it becomes available. - After the study is completed at a given site, the electronic data collection tool will be taken offline to prevent the entry of new data or changes to existing data. - If a site receives a report of a new SAE from a study participant or receives updated data on a previously reported SAE after the electronic data collection tool has been taken offline, then the site can report this information on a paper SAE form (see next table) or to the [X/medical monitor/SAE coordinator] by telephone. - Contacts for SAE reporting can be found in [X]. |

| SAE Reporting to [X] via Paper Data Collection Tool |
| --- |
| - [Facsimile transmission of the SAE paper data collection tool is the preferred method to transmit this information to the [X/medical monitor/SAE coordinator]]. - [In rare circumstances and in the absence of facsimile equipment, notification by telephone is acceptable with a copy of the SAE paper data collection tool sent by overnight mail or courier service.] - Initial notification via telephone does not replace the need for the investigator to complete and sign the SAE paper data collection tool within the designated reporting time frames. - Contacts for SAE reporting can be found in [X]. |

### Reporting of SADEs

| SADE Reporting to [X] |
| --- |
| NOTE: There are additional reporting obligations for medical device deficiencies that are potentially related to SAEs that must fulfill the legal responsibility to notify appropriate regulatory authorities and other entities about certain safety information relating to medical devices being used in clinical studies.   - Any device deficiency that is associated with an SAE must be reported to the sponsor within 24 hours after the investigator determines that the event meets the definition of a device deficiency. - The sponsor will review all device deficiencies and determine and document in writing whether they could have led to an SAE. These device deficiencies will be reported to the regulatory authorities and IRBs/IECs as required by national regulations. - Contacts for SAE reporting can be found in [X]. |

<End of common text>

## Appendix 8: Country-specific Requirements

Content for this appendix may be in the master protocol or the ISA as applicable. If this content is in the ISA, add the statement under common text below.

<Start of common text>

Refer to the applicable ISA for Appendix 8: Country-specific Requirements.

<End of common text>

Delete appendix if not required.

Do not use this appendix to create extensive lists of country-specific differences. Protocol requirements and specifications outlined in the body of the protocol should be authored using flexible language to accommodate local variation where permissible and within the parameters of the study design; this appendix should be used for requirements that cannot be addressed by flexible language.

Discuss with local regulatory groups whether country specific requirements need to be included in the appendix. The country-specific appendix may include a list (by country) of country-specific requirements in order that any requirements for a given country can be seen in one location.

Country-specific requirements listed in the appendix should also be clearly cross-referenced within the body of the document, within the sections they refer to, but details should not be included.

Countries where contraception requirements may differ: Australia, Japan

Korea: Local sponsor should be identified in addition to company sponsor on protocol agreement page.

## Appendix 9: Abbreviations [and Definitions]

This appendix may be in the master protocol and each ISA.

Delete appendix if not required.

- Generate a list while drafting the protocol to reflect the abbreviations used in the protocol.
- Only include those that are used more than once in the document. Once a term is abbreviated, it should be abbreviated in the rest of the document.
- Abbreviations are defined where first used in the document. If suggested text contains an abbreviation, the author can choose to retain the abbreviation or substitute the whole word(s).

## Appendix 10: Protocol Amendment History

Note that the master protocol and the ISA protocol can be amended separately. Therefore, the amendment numbers for the companion protocols may not align. Further, the amendment history for the ISA protocol will be in the ISA protocol and the amendment history for the master protocol will be in the master protocol.

Delete appendix if not required.

Example text is included in this appendix for the Protocol Amendment History located here and the Protocol Amendment Summary of Changes Table located before the table of contents.

<Start of common text>

The Protocol Amendment Summary of Changes Table for the current amendment is located directly before the table of contents (TOC).

See the instructions in the Protocol Amendment Summary of Changes Table located before the table of contents. Move all Protocol Amendment Summary of Changes Tables for previous amendments to this appendix.

Amendment [amendment number]: ([date])

This amendment is considered to be [substantial/nonsubstantial] based on the criteria set forth in Article 10(a) of Directive 2001/20/EC of the European Parliament and the Council of the European Union.

**Overall Rationale for the Amendment**

[Rationale]

| Section # and Name | Description of Change | Brief Rationale |
| --- | --- | --- |
|  |  |  |
|  |  |  |
|  |  |  |

<End of common text>

<Start of example text>

**Amendment 3: 30 March 2016**

This amendment is considered to be substantial based on the criteria set forth in Article 10(a) of Directive 2001/20/EC of the European Parliament and the Council of the European Union.

**Overall Rationale for the Amendment**

Current literature supports use of this class of interventions in a higher age range for this participant population.

| **Section # and Name** | **Description of Change** | **Brief Rationale** |
| --- | --- | --- |
| 5.1. Inclusion Criteria | Removed maximum age range | To better reflect the age of the participant population |
| Throughout | Minor editorial and document formatting revisions | Minor, therefore have not been summarized |

Example of Numbering Global and Country-specific Protocol Amendments

| Type of Protocol Amendment | Numbering | Type of changes |
| --- | --- | --- |
| Country-specific | Amendment 3/FRA-2 | Same changes specific to France added to global Amendment 3 (no new changes for France) |
| Global | Amendment 3 | New changes for all |
| Country-specific | Amendment 2/FRA-2 | Additional changes specific to France added to global Amendment 2 |
| Country-specific | Amendment 2/FRA-1 | Same changes specific to France added to global Amendment 2 (no new changes for France) |
| Global | Amendment 2 | New changes for all |
| Country-specific | Amendment 1/FRA-1 | Same changes specific to France added to global Amendment 1 (no new changes for France) |
| Global | Amendment 1 | New changes for all |
| Country-specific | Amendment FRA-1 | Changes specific to France added to original protocol |

Example of Numbering a Site-specific Protocol Amendment

| Type of Protocol Amendment | Numbering | Type of changes |
| --- | --- | --- |
| Site-specific | Amendment 2/SS-1 <<Insert Site Number(s)>> | Same changes specific to site(s) added to global Amendment 2 (no new changes for site[s]) |
| Global | Amendment 2 | New changes for all |
| Site-specific | Amendment 1/SS-1 <<Insert Site Number(s)>> | Changes specific to site(s) added to global amendment |
| Global | Amendment 1 | New changes for all |

Example of Document History Table for Global and Country-specific Protocol Amendments

| DOCUMENT HISTORY | |
| --- | --- |
| Document | Date of Issue |
| Amendment 2/FRA-1 | 1-Feb-2016 |
| Amendment 2 | 1-Feb-2016 |
| Amendment 1/FRA-1 | 1-Jan-2015 |
| Amendment 1 | 01-Dec-2015 |
| Original Protocol | 01-Oct-2015 |

Example of Document History Table for Site-specific Amendments to a Global Amendment

| DOCUMENT HISTORY | |
| --- | --- |
| Document | Date of Issue |
| Amendment 2/SS-1 | 1-Feb-2016 |
| Amendment 2 | 1-Feb-2016 |
| Amendment 1/SS-1 | 1-Jan-2015 |
| Amendment 1 | 01-Dec-2015 |
| Original Protocol | 01-Oct-2015 |

<End of example text>

## APPENDIX 10: Collection, storage and future use of data and human biological samples

Appendices to be provided for studies conducted in European countries.

### Compliance with Member State applicable rules for the collection, storage and future use of human biological samples (Article 7.1h)

This appendix is provided separately.

### Compliance with Member State applicable rules for the collection, storage and future use of (personal) data (article 7 (1 d) of EU Regulation 536/2014)

This appendix is provided separately.

## Appendix 11: Additional Appendices (if needed)

Add additional study-specific appendices in this content control.

If needed, several appendices can be added in this same content control with separate Level 2 headings for each

If there are no additional appendices, delete this heading and content control

# References

- See therapeutic libraries for key references to include.
- References to both internal and external documents and publications should be listed in alphabetical order. Do not reference internal reports in preparation.
- In the reference list, use the style and format published by the International Committee of Medical Journal Editors (ICMJE 2019). Citations to external documents and publications should be indicated in the text by citing the author and year within parentheses. For example, the in-text citation for the reference included would be (Hatcher et al, 2007).
- References may be in the master protocol and the ISAs as applicable.

<Start of example text>

Hatcher RA, Trussell J, Nelson AL, Cates W Jr, Stewart F, Kowal D, eds. Contraceptive technology. 19th edition. New York: Ardent Media, 2007(a): 24. Table 3-2.

<End of example text>

<Start of suggested text>

EU‑PEARL D2.1. Report on Terminology, References and Scenarios for Platform Trials and Master Protocols.

IMI2 Call15-01: <https://ec.europa.eu/info/funding-tenders/opportunities/portal/screen/opportunities/topic-details/imi2-2018-15-01>

CTFG: Recommendation paper on the initiation and conduct of complex clinical trials. <https://www.hma.eu/fileadmin/dateien/Human_Medicines/01-About_HMA/Working_Groups/CTFG/2019_02_CTFG_Recommendation_paper_on_Complex_Clinical_Trials.pdf>

TransCelerate BIOPHARMA INC., Clinical Content & Reuse Solutions. <https://www.transceleratebiopharmainc.com/assets/clinical-content-reuse-solutions/>

Woodcock J and LaVange LM. Master Protocols to Study Multiple Therapies, Multiple Diseases, or Both. N Engl J Med. 2017;377:62-70.

<End of suggested text>
