# Supplementary material for: Developing generic templates to shape the future for conducting integrated research platform trials
Source: Trials. 2024 Mar 21;25:204. doi: 10.1186/s13063-024-08034-8 (PMC10956223; doi:10.1186/s13063-024-08034-8)
Supplement: Supplementary file 2 — Additional file 2. EU-PEARL Intervention Specific Appendix template. [file 13063_2024_8034_MOESM2_ESM.docx]

| 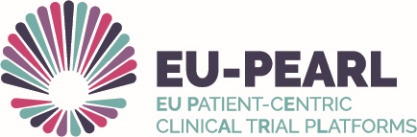 | 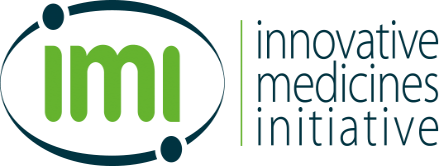 |
| --- | --- |

**Document title:**

**Intervention Specific Appendix**

**Protocol Template**

| **Document history and plan** |
| --- |
| This updated document (V4 25April2023) is based on the **EU-PEARL** **D2.3** ‘Provisional Generic Master Protocol Template and Appendix for IRPs’ (V2 30 April 2021). |
| D2.3 was based on TransCelerate Common Protocol Template CPT V8.0, copyright TransCelerate Biopharma Inc. 2015 – 2020. |
| Input received from the review committee was assessed for incorporation in the final deliverable D2.6 which is publicly available at the end of the project (April 2023). |

| **Authors** | Kathy Hersh, Cecile Spiertz, Heidi De Smedt, Ingela Larsson, Nick Di Prospero (Janssen); Madhavi Gidh-Jain (Sanofi); Peter Mesenbrink, Ekkehard Glimm (Novartis); Tom Parke (Berry); Franz Koenig (MUW), Juan Espinoso Pereiro (VHIR) |
| --- | --- |
| **Contact person EU-PEARL** | ECRIN; Peter Mesenbrink (Novartis) |
| **Document version** | Version 4 |
| **Date** | 25April2023 |

The EU-PEARL project has received funding from the Innovative Medicines Initiative 2 Joint Undertaking (JU) under grant agreement No 853966. The JU receives support from the European Union’s Horizon 2020 research and innovation programme and EFPIA and CHILDREN'S TUMOR FOUNDATION, GLOBAL ALLIANCE FOR TB DRUG DEVELOPMENT NON PROFIT ORGANISATION, SPRINGWORKS THERAPEUTICS INC.

**Disclaimer**

These materials are provided AS IS WITHOUT WARRANTY OF ANY KIND, EITHER EXPRESSED OR IMPLIED, INCLUDING, BUT NOT LIMITED TO, THE IMPLIED WARRANTIES OF MERCHANTABILITY, FITNESS FOR A PARTICULAR PURPOSE, OR NONINFRINGEMENT.

EU-PEARL and its members do not accept any responsibility for any loss of any kind including loss of revenue, business, anticipated savings or profits, loss of goodwill or data, or for any indirect consequential loss whatsoever to any person using these materials or acting or refraining from action as a result of the information contained in these materials. Any party using these materials bears sole and complete responsibility for ensuring that the materials, whether modified or not, are suitable for the particular use and are accurate, current, commercially reasonable under the circumstances, and comply with all applicable laws and regulations.

Nothing in this template should be construed to represent or warrant that persons using this template have complied with all applicable laws and regulations. All individuals and organizations using this template bear responsibility for complying with the applicable laws and regulations for the relevant jurisdiction

| 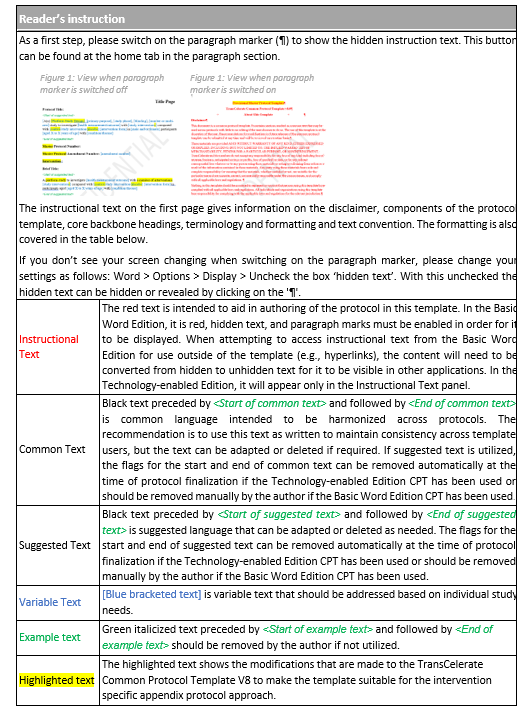 |
| --- |

**Provisional Intervention Specific Appendix Protocol Template**

**TransCelerate Common Protocol Template v8.0**

**About This Template**

**Disclaimer**

This document is a common protocol template. It contains sections marked as common text or text that may be used across protocols with little to no editing if the user chooses to do so. The use of this template is at the discretion of the user. Recommendations for modifications in future releases of the common protocol template can be submitted at any time and will be reviewed on a routine basis.

These materials are provided AS IS WITHOUT WARRANTY OF ANY KIND, EITHER EXPRESSED OR IMPLIED, INCLUDING, BUT NOT LIMITED TO, THE IMPLIED WARRANTIES OF MERCHANTABILITY, FITNESS FOR A PARTICULAR PURPOSE, OR NONINFRINGEMENT. TransCelerate and its members do not accept any responsibility for any loss of any kind including loss of revenue, business, anticipated savings or profits, loss of goodwill or data, or for any indirect consequential loss whatsoever to any person using these materials or acting or refraining from action as a result of the information contained in these materials. Any party using these materials bears sole and complete responsibility for ensuring that the materials, whether modified or not, are suitable for the particular use and are accurate, current, commercially reasonable under the circumstances, and comply with all applicable laws and regulations.

Nothing in this template should be construed to represent or warrant that persons using this template have complied with all applicable laws and regulations. All individuals and organizations using this template bear responsibility for complying with the applicable laws and regulations for the relevant jurisdiction.

Throughout this protocol template, the yellow highlighted text shows the modifications that are made to the TransCelerate Common Protocol Template V8 to make the template suitable for the intervention specific appendix protocol approach.

**Components of the Protocol Template**

- The **Core Backbone** contains protocol information common to all phases, study populations, and therapeutic areas. The core backbone is streamlined and focused on the sites’ needs.
- **Libraries** group and store content that will be inserted into the core backbone and contain specific information related to therapeutic area, study intervention, country, and study population (eg, participant, healthy volunteer). For pediatric or adult/pediatric studies, include the content contained in the pediatric library.
- **Appendices** provide additional information that can be accessed when needed (eg, abbreviations, standard content regarding adverse event [AE] definitions).

**Core Backbone Headings**

- Level 1 and 2 headings should be consistent across protocols that use the CPT for reference and mapping purposes. The structure of this template aligns with the structure of the United States National Institutes of Health (NIH) and Food and Drug Administration (FDA) Clinical Trials Protocol Template
- Level 1 and 2 headings should not be deleted. If they are not relevant to the study, not applicable should be inserted so that the numbering of subsequent sections is not changed.
- Level 3 and subsequent headings are suggested and can be deleted/added/modified as needed with the exception of those in Section 8.3 relating to Adverse Events which are International Council on Harmonisation (ICH)/regulatory agency required wording and must be included.

**Terminology**

- The following terminology has been selected for use within TransCelerate common templates (protocol, statistical analysis plan [SAP], and clinical study report [CSR]) and is considered to be appropriate for all phases, study populations, and therapeutic areas.
  - *Participant* is used rather than subject, healthy volunteer, or patient.
  - *Study intervention* is used rather than study drug. Study intervention covers all types of investigational and noninvestigational products including medical devices and vaccines.
    - Study intervention is defined as investigational intervention(s), marketed product(s), placebo, or medical device(s) intended to be administered to a study participant per protocol.
  - *Effectiveness* is used for medical device studies whereas for drugs, *efficacy* is used. Template should be updated as appropriate.

**Formatting and Text Conventions**

- Common Text: Black font preceded by <*Start of common text*> and followed by <*End of common text*> is common language intended to be harmonized across protocols. The recommendation is to use this text as written to maintain consistency across template users, but the text can be adapted or deleted if required. If suggested text is utilized, the flags for the start and end of common text can be removed automatically at the time of protocol finalization if the Technology-enabled Edition CPT has been used or should be removed manually by the author if the Basic Word Edition CPT has been used..
- Suggested Text: Black textpreceded by *<Start of suggested text>* and followed by *<End of suggested text>* is suggested language that can be adapted or deleted as needed. The flags for the start and end of suggested text can be removed automatically at the time of protocol finalization if the Technology-enabled Edition CPT has been used or should be removed manually by the author if the Basic Word Edition CPT has been used.
- Variable Text: Blue bracketed text is variable text that should be addressed based on individual study needs.
- Example Text: Green italicized text preceded by *< Start of example text>* and followed by *< End of example text >* should be removed by the author if not utilized.
- Instructional Text: Is intended to aid in authoring of the protocol in this template. In the Basic Word Edition, it is red, hidden text, and paragraph marks must be enabled in order for it to be displayed. When attempting to access instructional text from the Basic Word Edition for use outside of the template (eg, hyperlinks), the content will need to be converted from hidden to unhidden text for it to be visible in other applications. In the Technology-enabled Edition,it will appear only in the Instructional Text panel.

Title Page

**Intervention Specific Appendix Title:**

Intervention Specific Appendix Title: The Intervention Specific Appendix should have a descriptive title that identifies it sufficiently to ensure it is immediately evident what it is investigating and on whom, and to allow retrieval from literature or internet searches. It does not have to duplicate the title of the master protocol but should provide a logical link to the title of the master protocol.

<Start of suggested text>

[Intervention Specific Appendix Protocol ## to Master Protocol ####-####].

<End of suggested text>

**Example:**

Intervention Specific Appendix Protocol to Master Protocol ####-####]: A parallel group treatment, Phase 2, participant and investigator masked, two-arm study to assess the safety and effectiveness of Addiryn tablets compared to placebo tablets for decreasing agitation in male and female participants aged 60 to 85 inclusive with Alzheimer's Disease.

A structured title should contain details of participants, interventions (and acronyms if relevant), intervention forms, comparison groups, outcomes and study design. Use of the terms below will ensure alignment with Clinical Trials Registry Data Element Definitions.

Enter values from the list given for each of the indicated fields to complete.

**Intervention Model:**

- Single Group: Clinical studies with a single arm
- Parallel Group: Participants are assigned to 1 of 2 or more groups in parallel for the duration of the study
- Crossover: Participants receive 1 of 2 (or more) alternative interventions during the initial phase of the study and receive the other intervention during the second phase of the study
- Factorial: Two or more interventions, each alone and in combination, are evaluated in parallel against a control group
- Sequential: Groups of participants are assigned to receive interventions based on prior milestones being reached in the study, such as in some dose escalation and adaptive design studies

**Primary Purpose:**

- Treatment: One or more interventions are being evaluated for treating a disease, syndrome, or condition.
- Prevention: One or more interventions are being assessed for preventing the development of a specific disease or health condition.
- Diagnostic: One or more interventions are being evaluated for identifying a disease or health condition.
- Supportive Care: One or more interventions are evaluated for maximizing comfort, minimizing side effects, or mitigating against a decline in the participant's health or function.
- Screening: One or more interventions are assessed or examined for identifying a condition, or risk factors for a condition, in people who are not yet known to have the condition or risk factor.
- Health Services Research: One or more interventions for evaluating the delivery, processes, management, organization, or financing of healthcare.
- Basic Science: One or more interventions for examining the basic mechanism of action (for example, physiology or biomechanics of an intervention).
- Other: None of the other options applies.

**Study Phase:**

See definitions under heading for Study Phase and enter same phase in each place.

**Blinding**

Insert/copy definition from Overall Design section.

**Number of Arms**:

Numeric value for the number of arms in the study.

**Health Measurement/Outcome:**

What is the primary outcome as given in the objectives being examined to determine the effect from the intervention? This should be included in the protocol title written in lay language, eg, measure the reduction in bad cholesterol; other potential terms: treat, delay, confirm, predict, identify, reduce, correct, reverse, lower, decrease, increase or improve. Ensure this is written as an action/verb.

**Intervention Name**:

Enter a generic (international nonproprietary name [INN]) or trade name if required as per chemistry, manufacturing, and controls (CMC), if applicable.

**Intervention Form:**

eg, tablet, ampule, capsule, pill, patch, cream, ointment

**Participant Sex:**

eg, male, female, male and female

**Participant Age Range:**

eg,18-65 years of age, 10-18 years of age

**Condition/Disease:**

The disease, disorder, syndrome, illness, or injury, etc, that is being studied.

**Intervention Specific Appendix Protocol Number: if applicable**

**Intervention Specific Appendix Amendment Number:** [amendment number]

**Intervention:** [number or name]

**Brief Title:**

<Start of suggested text>

Intervention Specific Appendix 1 to Therapeutics for Inpatients with COVID-19

<End of suggested text>

Short title should be sufficiently detailed to make clear to a lay reader what the study is about and suitable for use as the Brief Title in ClinicalTrials.gov and for use with informed consents and ethics committee submissions.

Based on NIH expectations and participant preferences, the optimal Brief Title on ClinicalTrials.gov includes the following data elements: condition/disease, health measurements/observation, intervention name, intervention form, participant age range, and participant sex.

Definitions of these terms are in the guidance following the additional details section.

Additional details:

- Reference to “*participants*” as the preferred term
- All abbreviations are defined
- Does not end with a period
- Technical study design terms are avoided.
- Limited to 300 characters

Study Phase: [study phase]

-Please select one of the values for this field:

- N/A: for studies without phases (eg, studies of behavioral interventions)

- Early Phase 1 exploratory studies, involving very limited human exposure, with no therapeutic or diagnostic intent (eg, screening studies, microdose studies)

- Phase 1: includes initial studies to determine the metabolism and pharmacologic actions of interventions in humans, the side effects associated with increasing doses, and to gain early evidence of effectiveness; may include healthy participants and/or participants

- Phase 1/Phase 2: for studies that are a combination of Phases 1 and 2

- Phase 2: includes controlled clinical studies conducted to evaluate the effectiveness of the intervention for a particular indication or indications in participants with the disease or condition under study and to determine the common short-term side effects and risks

- Phase 2/Phase 3: for studies that are a combination of Phases 2 and 3

- Phase 3: studies conducted after preliminary evidence suggesting effectiveness of the intervention has been obtained, and are intended to gather additional information to evaluate the overall benefit-risk relationship of the intervention.

- Phase 4: studies of FDA-approved interventions to delineate additional information including risks, benefits, and optimal use

**[Acronym]**:

Acronym or abbreviation used publicly to identify the clinical study, if any.
Limit: 14 characters. Delete if not applicable.

**Intervention Owner Name:**

Legal Registered Address:

The intervention owner name and legal registered address must be included.

In some countries, the intervention owner may be the local affiliate company (or designee). If applicable, the details of the alternative intervention owner and contact person in the territory should be provided to the relevant regulatory authority as part of the clinical study application and should not be included in the protocol.

**Regulatory Agency Identifier Number(s)**

Include all numbers that are applicable for the study and available at the time of protocol or amendment finalization eg, investigational new drug (IND) number (include the center number, IND/ide number, serial number), World Health Organization (WHO) universal trial number, European Clinical Trials Database (EudraCT) number, ClinicalTrials.gov. Add type and number as applicable.

| **Registry** | **ID** |
| --- | --- |
|  |  |

**Approval Date:**

Intervention owner Signatory:

| **[Name]**  **[Title]** |  | **Date** |
| --- | --- | --- |

Medical Monitor Name and Contact Information [will be provided separately OR can be found in XX]

The investigator signature page is generated internally as a standalone document and should be provided to the investigator for signature alongside the final protocol. The investigator should retain the original in the site study files and return a copy to the intervention owner for archiving in the trial master file (TMF). In case of a protocol amendment, ensure that the protocol version is noted on the investigator signature page.

ISA Protocol Amendment Summary of Changes Table

**Delete this section if this is not an amendment.**

Note that the master protocol and the Intervention Specific Appendix (ISA) protocol can be amended separately. Therefore, the amendment numbers for the companion protocols may not align. Further, the amendment history for the ISA protocol will be in the ISA protocol and the amendment history for the master protocol will be in the master protocol.

Protocols should not be developed with the intent to amend; however, if an amendment is required, the following process and template is recommended. Companies should modify this process as appropriate (eg, naming conventions, designation of substantial/nonsubstantial amendment status) to ensure alignment with their internal processes and systems.

**GENERAL INSTRUCTIONS:**

- Protocols should be amended by making the changes directly within the protocol.
- In addition to the summary of changes table, incorporate the changes made as a result of the amendment into the respective CPT sections and create
  - a new clean version.
  - a new version with the changes highlighted (ie, tracked changes) to be provided to the health authorities, if required.
- NOTE: For substantial amendments: use the tracked‑changes version of the protocol to create a separate document with a tabular listing detailing section changed, initial wording, amended or new wording, reason/justification for change and reason for substantial amendment as this is now required by many health authorities.
- Include the heading: Protocol Amendment Summary of Changes in the table of contents (TOC) as a non-numbered heading.
- Modify the protocol number as appropriate throughout the document as specific to the company (eg, title page, page headers) to designate status as an amendment.
- See Appendix 10, ISA Protocol Amendment History for further instructions and examples for completing this section.
- The common text section titled Document History should be completed for each amendment.
- Amendments should appear in reverse chronological order with the most recent at the top (eg, Amendment 3, 2, 1).
- The ISA Protocol Amendment Summary of Changes Table for the current amendment should be maintained directly in front of the TOC.
- The ISA Protocol Amendment Summary of Changes Table for the previous amendment(s) should be moved to Appendix 10, ISA Protocol Amendment History.
- Group changes by rationale and list rationales by order of importance, with the rationale for the most important study design changes listed first. Under each rationale, list changes in order of occurrence in the protocol.

Relevant changes may have been made to the protocol template since the original protocol or last amendment was issued. Check the template change control documentation and discuss with the team to ensure all relevant changes have been added to the protocol and included in the Protocol Amendment Summary of Changes Table.

***NAMING CONVENTIONS*** for differentiation of types of amendments (eg, global, country-specific, site‑specific):

Use International Organization for Standardization (ISO)-Alpha 3 Codes from the United Nations Statistics Department for 3-letter codes to represent country or area name in country-specific amendments: https://www.nationsonline.org/oneworld/countrycodes.htm**.**

Examples can be found in Appendix 10, ISA Protocol Amendment History.

***NUMBERING CONVENTIONS***

- Global amendments should be sequentially numbered (eg, Amendment 1, Amendment 2, Amendment 3, etc).
- Country-specific amendments should list the 3-digit ISO-Alpha 3 Codes (link above) with sequential numbering (eg, for France, the 3-digit code is FRA. The first country-specific amendment for France should be numbered Amendment FRA-1. If a second amendment is required with content specific to France, it would be Amendment FRA-2.).
  When adding an amendment ensure that the country-specific changes are maintained with each global update.
  - A country-specific amendment to a global amendment

or

- - A global amendment to a country-specific amendment.

Examples can be found in Appendix 10, ISA Protocol Amendment History.

***DOCUMENT HISTORY***

- The Document History table should be inserted at the beginning of each amendment and contain the document number and date for each amendment.
- Global amendments should not list the country- or site-specific amendments in the table.
- Country- and site-specific amendments should list the global amendments.
- Country-specific amendments should not list the site-specific amendments.
- Site-specific amendments should only list country-specific amendments for that specific country.
- If an amendment with identical changes is needed for multiple countries/areas/sites, they may be named as
  - Region 1 (list country/area codes as ISO-Alpha 3 Codes from the United Nations Statistics Department as noted above)
  - Region 2 (list country/area codes as ISO-Alpha 3 Codes from the United Nations Statistics Department as noted above)
  - Site-specific SS-1 (sites numbers)

The rationale for not including the entire list of amendments in the Document History table is that the global amendments apply to all countries and sites, while the country- and site-specific amendments are just that, ‘specific,’ and therefore do not apply to all.

Examples can be found in Appendix 10, ISA Protocol Amendment History.

List dates of original protocol and all amendments in reverse chronological order.

<Start of common text>

| DOCUMENT HISTORY | |
| --- | --- |
| Document | Date |
| [Amendment X] | [Day-Mon-Year] |
| [Amendment X] | [Day-Mon-Year] |
| [Amendment X] | [Day-Mon-Year] |
| Original Protocol | [Day-Mon-Year] |

Amendment [X] (Day-Month-Year)

Include the following statement if this amendment will be implemented in any European Union (EU) member state. Include the last phrase for nonsubstantial amendments only.

<Start of suggested text>

This amendment is considered to be [substantial] [nonsubstantial] based on the criteria set forth in Article 10(a) of Directive 2001/20/EC of the European Parliament and the Council of the European Union [because it neither significantly impacts the safety or physical/mental integrity of participants nor the scientific value of the study].

<End of suggested text>

Overall Rationale for the Amendment:

The overall rationale (one primary driver) for the changes implemented in the protocol amendment should be provided. In addition, provide a high-level description of the change(s) and a brief scientific rationale for specific items outlined in the table provided (eg, changes to individual inclusion/exclusion criteria). See Appendix 10, ISA Protocol Amendment History for examples of format and green text for sample content.

[INSERT rationale statement]

| Section # and Name | Description of Change | Brief Rationale |
| --- | --- | --- |
| [INSERT] | [INSERT] | [INSERT] |
| [INSERT] | [INSERT] | [INSERT] |
| [INSERT] | [INSERT] | [INSERT] |
|  |  |  |
|  |  |  |

<End of common text>

Table of Contents

[1. Protocol Summary 14](#_Toc62724856)

[1.1. Synopsis 14](#_Toc62724857)

[1.2. Schema 15](#_Toc62724858)

[1.3. Schedule of Activities (SoA) 16](#_Toc62724859)

[2. Introduction 20](#_Toc62724860)

[2.1. Study Rationale 20](#_Toc62724861)

[2.2. Background 20](#_Toc62724862)

[2.3. Benefit/Risk Assessment 21](#_Toc62724863)

[2.3.1. Risk Assessment 21](#_Toc62724864)

[2.3.2. Benefit Assessment 21](#_Toc62724865)

[2.3.3. Overall Benefit: Risk Conclusion 22](#_Toc62724866)

[3. Objectives, Endpoints and Estimands 23](#_Toc62724867)

[4. Study Design 24](#_Toc62724868)

[4.1. Overall Design 24](#_Toc62724869)

[4.2. Scientific Rationale for Study Design 24](#_Toc62724870)

[4.2.1. Participant Input into Design 24](#_Toc62724871)

[4.3. Justification for Dose 24](#_Toc62724872)

[4.4. End of Study Definition 24](#_Toc62724873)

[5. Study Population 25](#_Toc62724874)

[5.1. Inclusion Criteria 25](#_Toc62724875)

[5.2. Exclusion Criteria 26](#_Toc62724876)

[5.3. Lifestyle Considerations 26](#_Toc62724877)

[5.3.1. Meals and Dietary Restrictions 26](#_Toc62724878)

[5.3.2. Caffeine, Alcohol, and Tobacco 26](#_Toc62724879)

[5.3.3. Activity 27](#_Toc62724880)

[5.4. Screen Failures 27](#_Toc62724881)

[5.5. Criteria for Temporarily Delaying [Enrollment/Randomization/Administration of Study Intervention] 27](#_Toc62724882)

[6. Study Intervention(s) and Concomitant Therapy 28](#_Toc62724883)

[6.1. Study Intervention(s) Administered 28](#_Toc62724884)

[6.2. Preparation, Handling, Storage, and Accountability 30](#_Toc62724885)

[6.3. Measures to Minimize Bias: Randomization and Blinding 30](#_Toc62724886)

[6.4. Study Intervention Compliance 30](#_Toc62724887)

[6.5. Dose Modification 31](#_Toc62724888)

[6.5.1. Retreatment Criteria 32](#_Toc62724889)

[6.6. Continued Access to Study Intervention after the End of the Study 32](#_Toc62724890)

[6.7. Treatment of Overdose 32](#_Toc62724891)

[6.8. Concomitant Therapy 33](#_Toc62724892)

[6.8.1. Rescue Medicine 33](#_Toc62724893)

[7. Discontinuation of Study Intervention and Participant Discontinuation/Withdrawal 34](#_Toc62724894)

[7.1. Discontinuation of Study Intervention 34](#_Toc62724895)

[7.1.1. Liver Chemistry Stopping Criteria 34](#_Toc62724896)

[7.1.2. QTc Stopping Criteria 34](#_Toc62724897)

[7.1.3. Temporary Discontinuation 34](#_Toc62724898)

[7.1.4. Rechallenge 34](#_Toc62724899)

[7.2. Participant Discontinuation/Withdrawal from the Study 35](#_Toc62724900)

[7.3. Lost to Follow up 35](#_Toc62724901)

[8. Study Assessments and Procedures 36](#_Toc62724902)

[8.1. [Efficacy and/or Immunogenicity] Assessments 36](#_Toc62724903)

[8.2. Safety Assessments 36](#_Toc62724904)

[8.2.1. Physical Examinations 36](#_Toc62724905)

[8.2.2. Vital Signs 36](#_Toc62724906)

[8.2.3. Electrocardiograms 36](#_Toc62724907)

[8.2.4. Clinical Safety Laboratory Assessments 36](#_Toc62724908)

[8.2.5. Pregnancy Testing 36](#_Toc62724909)

[8.2.6. Suicidal Ideation and Behavior Risk Monitoring 36](#_Toc62724910)

[8.3. Adverse Events (AEs), Serious Adverse Events (SAEs), and Other Safety Reporting 37](#_Toc62724911)

[8.3.1. Time Period and Frequency for Collecting AE and SAE Information 37](#_Toc62724912)

[8.3.2. Method of Detecting AEs and SAEs 37](#_Toc62724913)

[8.3.3. Follow-up of AEs and SAEs 37](#_Toc62724914)

[8.3.4. Regulatory Reporting Requirements for SAEs 37](#_Toc62724915)

[8.3.5. Pregnancy 37](#_Toc62724916)

[8.3.6. Cardiovascular and Death Events 38](#_Toc62724917)

[8.3.7. Disease-related Events and/or Disease-related Outcomes Not Qualifying as AEs or SAEs 38](#_Toc62724918)

[8.3.8. Adverse Events of Special Interest 38](#_Toc62724919)

[8.4. Pharmacokinetics 38](#_Toc62724920)

[8.5. Genetics 39](#_Toc62724921)

[8.6. Biomarkers 39](#_Toc62724922)

[8.7. Immunogenicity Assessments 39](#_Toc62724923)

[8.8. [Health Economics OR Medical Resource Utilization and Health Economics] 39](#_Toc62724924)

[9. Statistical Considerations 40](#_Toc62724925)

[9.1. Statistical Hypotheses 40](#_Toc62724926)

[9.1.1. Multiplicity Adjustment 40](#_Toc62724927)

[9.2. Analysis Sets 40](#_Toc62724928)

[9.3. Statistical Analyses 40](#_Toc62724929)

[9.3.1. General Considerations 40](#_Toc62724930)

[9.3.2. Primary Endpoint(s) 41](#_Toc62724931)

[9.3.3. Secondary Endpoint(s) 41](#_Toc62724932)

[9.3.4. Tertiary/Exploratory Endpoint(s) 41](#_Toc62724933)

[9.3.5. [Other/safety] Analysis 42](#_Toc62724934)

[9.3.6. Other Analysis 42](#_Toc62724935)

[9.4. Interim Analysis 42](#_Toc62724936)

[9.5. Sample Size Determination 42](#_Toc62724937)

[10. Supporting Documentation and Operational Considerations 44](#_Toc62724938)

[10.1. Appendix 1: Regulatory, Ethical, and Study Oversight Considerations 44](#_Toc62724939)

[10.2. Appendix 2: Clinical Laboratory Tests 44](#_Toc62724940)

[10.3. Appendix 3: AEs and SAEs: Definitions and Procedures for Recording, Evaluating, Follow-up, and Reporting 44](#_Toc62724941)

[10.4. Appendix 4: Contraceptive and Barrier Guidance 44](#_Toc62724942)

[10.4.1. Definitions 44](#_Toc62724943)

[10.4.2. Contraception Guidance 44](#_Toc62724944)

[10.5. Appendix 5: Genetics 44](#_Toc62724945)

[10.6. Appendix 6: Liver Safety: Suggested Actions and Follow-up Assessments [and Study Intervention Restart/Rechallenge Guidelines] 44](#_Toc62724946)

[10.7. Appendix 7: Country-specific Requirements 44](#_Toc62724947)

[10.8. Appendix 8: Abbreviations [and Definitions] 44](#_Toc62724948)

[10.9. Appendix 9: ISA Protocol Amendment History 44](#_Toc62724949)

[11. References 47](#_Toc62724950)

# Protocol Summary

## Synopsis

The protocol synopsis is a short (1 to 2 pages) summary of the key points of the protocol. This section of the protocol should be completed after the main text to ensure consistency with the main text.

The purpose of the protocol synopsis is to provide a concise outline of the key aspects of the study. It may be used for EU clinical trial applications (CTA) and for other external bodies such as institutional review boards [IRB]/independent ethics committees [IEC]). Its level of detail should not dissuade/discourage the investigator from referring to the main text of the protocol.

**Protocol Title:**

Ensure wording here matches the title page

**Brief Title:**

Ensure wording here matches the title page

Rationale:

The synopsis text should be taken from the main text.

Objectives, Endpoints, and Estimands:

Refer to the master protocol synopsis for the objectives and endpoints. Add additional intervention-specific objectives and endpoints if applicable.

| Objectives | Endpoints |
| --- | --- |
| Primary |  |
| - Refer to master | - Refer to master |
| Secondary | Secondary |
| - Refer to master - Add any intervention-specific objectives | - Refer to master - Add any intervention-specific endpoints |
| [Tertiary/Exploratory/Other] | [Tertiary/Exploratory/Other] |
| - Add any intervention-specific objectives | - Add any intervention-specific endpoints |

Overall Design:

- Refer to the master protocol synopsis for the study design.
- Information specific to the ISA protocol may be added here.
- If not included in the master protocol, include the number of participants and intervention groups and duration for the intervention cohort.

<Start of common text>

A platform study is defined by the master protocol, which describes the overall clinical study design applicable to all related interventions. The intervention specific appendix (ISA) is the appendix to the related master protocol which describes the specific features of the intervention and treatment of participants randomized to that intervention. The master protocol together with an ISA has all the information needed to conduct an intervention cohort study.

<End of common text>

Number of Participants:

- State the expected number of participants to be screened, randomized/enrolled, when applicable. For an event driven study, state the number of events planned along with the number of participants to be randomized.
- Cross reference Section 9.5 Sample Size Determination and ensure that section clearly explains how screening failures and nonevaluable participants are defined.
- Choose 1 of the 2 options listed and modify as appropriate

<Start of suggested text>

Approximately [X] participants will be screened to achieve [X] [randomly assigned/enrolled] to study intervention.

OR

A maximum of [X] participants will be [randomly assigned/enrolled] to study intervention.

**Note**: *Enrolled* means a participant’s, or their legally acceptable representative’s, agreement to participate in a clinical study following completion of the informed consent process [and screening]. Potential participants who are screened for the purpose of determining eligibility for the study, but do not participate in the study, are not considered enrolled, unless otherwise specified by the protocol. A participant will be considered enrolled if the informed consent is not withdrawn prior to participating in any study activity after screening.

<End of suggested text>

Intervention Groups and Duration:

Briefly state:

- Total duration of study participation for each participant with sequence and duration of study periods (eg, screening, run-in, fixed dose/titration, follow-up/washout periods).
- Dose regimens in each study period and stage (if applicable) including frequency (eg, twice daily) and route of administration and criteria for individualized dosing (eg, participant weight or plasma concentrations), if applicable.
- Rules/procedures for any dose changes/adjustments including flexible dosing; dose reductions, interruptions, or tapering; temporary/permanent discontinuation; and any circumstances for resuming study intervention, as applicable.

## Schema

Refer to the master protocol for the study schema. In some situations, an intervention cohort specific study schema may be appropriate.

## Schedule of Activities (SoA)

General information:

- There may be situations where all the activities are included in the master protocol SoA and an ISA protocol SoA is not needed. In this case, include a statement to refer to the master protocol SoA.
- There may also be situations where the master protocol SoA is very high-level with a minimum of information and most activities will be in the ISA SoA. In this case, the ISA SoA should be populated accordingly. The SoA from the master protocol should be copied here. Rows that refer to the ISA should be populated with the intervention-specific cohort information. Additional rows should be added as needed after the last row of the master protocol SoA. Any intervention-specific events, procedures, assessments, can be entered in a separate column on the far left to indicate that these events are ISA specific.
- Ensure that only essential data are collected. The schedule of activities (SoA) is the primary location for specifying the timing of assessments at each stage of the study. Do not repeat the SoA schedule in the main text.
- Visit windows may be necessary for the collection of efficacy or safety data. The acceptable windows can be indicated on the SoA by adding ± days or hours/minutes to the visit day or timepoint row.
- If applicable, specify the order of assessments (eg, performing participant-recorded assessments before other assessments to reduce bias or performing electrocardiograms [ECG] or measuring vital signs before blood draws).
- Notes/footnotes (relating to specific procedures) should be minimal, brief, and include key information. If additional details are needed, the notes should refer to the section in the protocol main text where details are provided. Note that Day 0 should not be used as a timepoint.
- Combine assessments on consecutive weeks if they are identical and consider separate tables for separate periods of the study (eg, screening, intervention days, and follow-up). For a multiple-part study, one SoA table for each part of the study is recommended.
- The informed consent process may vary across different types of platform studies. Is it acknowledged that informed consent must be signed before the first study-related activity, which would typically be at the master protocol level (eg, master informed consent form [ICF]). After consent is signed for the master protocol, the informed consent process for the intervention cohort(s) may depend on the number of intervention cohort(s) in a platform study. If only a single intervention cohort is open in a platform study, the master protocol and ISA ICFs may be signed at the same time. If multiple intervention cohorts are open in a platform study and participants are to be allocated or randomized among intervention cohorts after screening at the master level, the ICFs may be signed sequentially, eg, the master ICF would be signed to permit screening procedures for allocation/randomization to an intervention cohort, followed by the applicable intervention-specific ICF. If there are specific screening or eligibility criteria that determine the intervention cohort that participants will be allocated or randomized to, these screening/eligibility criteria should be in the master protocol and the master ICF. Intervention-specific screening/eligibility criteria should only be in the ISA protocol and the ISA ICF.
- An example of an ISA SoA table is included. Modify as required.

| [Study intervention(s) *name*] specific assessments | Procedure | Screening  (up to 5 days before Day 1) | Baseline | Intervention Period [Weeks] | | | | | | E/D | Follow-up (Visit after last dose) | Notes |
| --- | --- | --- | --- | --- | --- | --- | --- | --- | --- | --- | --- | --- |
|  |  |  | Day1 | Week 1 | **Week 2** | **Week 4** | **Week 8** | **Week 12** | **Week 16** |  |  |  |
| X | Informed consent | X |  |  |  |  |  |  |  |  |  |  |
| X | Inclusion and exclusion criteria | X |  |  |  |  |  |  |  |  |  |  |
|  | Demography | X |  |  |  |  |  |  |  |  |  |  |
|  | Full physical examination including height and weight | X |  |  |  |  |  |  |  |  |  |  |
|  | Medical history (includes substance usage [and family history of premature CV disease]) | X |  |  |  |  |  |  |  |  |  | Substances: [drugs, alcohol, tobacco, and caffeine] |
|  | Past and current medical conditions | X |  |  |  |  |  |  |  |  |  |  |
|  | [Highly sensitive serum OR urine] pregnancy test (WOCBP only) | X | X |  |  |  |  |  |  | X | X | [refer to section 8.2.5 – pregnancy testing for instruction on timepoints] |
|  | [HIV, Hepatitis B, and C screening] | X |  |  |  |  |  |  |  |  |  |  |
|  | Laboratory tests (include liver chemistries) | X |  | X |  | X |  | X |  |  |  |  |
|  | 12-lead ECG | X |  |  |  | X |  |  | X |  |  |  |
|  | Vital signs | X |  | X | X | X | X | X | X | X | X |  |
|  | [Randomization] if applicable |  | X |  |  |  |  |  |  |  |  |  |
|  | Genetic sample |  | X |  |  |  | X |  | X |  |  |  |
|  | Study intervention |  | X | X | X | X | X | X | X |  |  |  |
|  | AE review |  | X | X | X | X | X | X | X |  |  |  |
|  | [Solicited Administration site events] if applicable |  | X | X | X | X | X | X | X | X | X |  |
|  | [Unsolicited AEs] if applicable |  | X | X | X | X | X | X | X | X | X | See Appendix 3 of the Master protocols for definitions  Consider separate tables for days with multiple assessments |
|  | SAE review |  | X | X | X | X | X | X | X | X | X |  |
|  | Concomitant medication review |  | X | X | X | X | X | X | X | X | X |  |
| X | Endoscopy |  | X |  |  | X |  |  | X |  |  | Endoscopies will be read by a central reader |
| X | Symptom improvement PRO |  |  | X |  |  |  | X |  |  |  | Collect from participant diaries |
|  |  |  |  |  |  |  |  |  |  |  |  |  |

# Introduction

- Overall, this section should be short (recommend 2 to 3 pages) and may be started with an overview description of the study intervention to be tested in this intervention cohort, its class, and intended use as well as the study population.
- Consider that the entire ISA protocol will be subject to public disclosure and be succinct.
- As much as possible, reference the investigator’s brochure (IB), package insert, and other relevant documents; do not duplicate information available elsewhere.
- Introduce the definition/concept of this ISA protocol and its supplementary relationship to the master protocol.
- Consider defining new terms/concepts, such an intervention cohort and [*arm naming convention*].

<Start of common text>

A platform study is defined by the master protocol, which describes the overall clinical study design applicable to all related interventions. The ISA is the appendix to the related master protocol which describes the specific features of the intervention and treatment of participants randomized to that intervention. The master protocol together with an ISA has all the information needed to conduct an intervention cohort study.

<End of common text>

<Start of suggested text>

The following terms are used throughout the master protocol and this ISA and are defined below.:

- Sponsor is the organization with overall responsibility for the platform study.
- Intervention owner is the organization that provides the investigational treatment and possibly the matching control for a interventional cohort
- articipant refers to the common term subject. (TransCelerate)
- Study intervention refers to common term study agent. (TransCelerate)
- A platform study is a study with multiple targeted therapies investigated in a single disease in a perpetual manner, with therapies allowed to enter or leave the platform on the basis of a decision algorithm. (Woodcock and Lavange, 2017) A platform study will typically consist of a master protocol and 1 or more ISAs.
- The master protocol is the document which describes the overall clinical study design applicable to all related interventions, such as the clinical study rationale, objectives, endpoints, benefit-risk assessment, shared procedures regarding safety monitoring and reporting, and a common screening platform dictating participant eligibility and/or treatment allocation. (CTFG, 2019)
- The ISA is the appendix to the related master protocol which describes the specific features of the intervention and treatment of participants randomized to that intervention. (IMI call) Each intervention will have a separate ISA. Together, a master protocol and an ISA define all the elements needed to conduct a study.
- Intervention cohort refers to the group of participants who receive a specific investigational intervention or an intervention-specific comparator (ie, placebo and/or an active comparator) and in whom that intervention is evaluated. In the event of a shared comparator group described in the master protocol, the intervention cohort refers to the group of participants who receive the investigational intervention. (EU-PEARL) The intervention cohort is described in the ISA.
- The investigational treatment arm refers to the group of participants in an intervention cohort who receive the investigational intervention of interest being evaluated in the ISA. (EU-PEARL)

<End of suggested text>

<Start of example text>

[Investigational intervention(s) name] is a novel, potent, and selective long-acting inhaled β2 adrenoreceptor agonist that is being developed for once-daily treatment of asthma and COPD.

<End of example text>

## Study Rationale

- The rationale for the platform study of the disease should be described in the master protocol. In the ISA, present the scientific rationale for the intervention being evaluated in the intervention cohort. For example, include any key issues for the compound that are being addressed (eg, variable exposure addressed with a new formulation or dosing with food).
- This section should be aligned with the overall development plan for the compound.
- This rationale should be based on the results of previous studies (if relevant) and the characteristics of the disease entity and should be of scientific merit.

## Background

Platform-level background information (eg, disease-specific information, unmet medical need, etc) should be in the master protocol. This section should contain information specific to the intervention being tested in the intervention cohort.

This section should be brief (1/2 to 1 page) as the majority of the information is available in existing documents. Include a 1 to 2 sentence description of why the study intervention is being developed for the disease (eg, unmet medical need, easier administration, better efficacy expected, better side effect profile). State whether this is a novel class of compounds or a new compound within an established class, and whether this class of compounds has been used before in the therapeutic area. Briefly refer to literature and data relevant to the study.

For studies using an unlicensed study intervention: include a very brief summary of key nonclinical/clinical data relevant to the development of the compound and pharmacodynamic/efficacy findings that support development for the indication. Do not duplicate data already summarized in the IB/package insert; a reference to the specific IB/package insert section is sufficient. When referencing information in the IB/package insert or other relevant documents provide a reference to the section or table where the data are presented.

For studies using marketed compounds or comparators: see the manufacturer’s label (include as a reference in Section 11) or provide a brief description of relevant information. To avoid copyright infringements, do not include a copy of the approved product label in the protocol.

<Start of example text>

[Study intervention name] belongs to a novel structural class of antibiotics: bacterial type II topoisomerase inhibitors (BTIs). The BTIs selectively inhibit bacterial DNA gyrase and topoisomerase IV (homologous type II topoisomerases), which are clinically-validated antibacterial targets inhibited by the quinolone family of antibiotics. The BTIs and quinolones bind to a similar region of the same target proteins; however, they recognize distinctly different amino acids. Therefore, they inhibit different stages of the catalytic cycle of the target proteins.

A detailed description of the chemistry, pharmacology, efficacy, and safety of [study intervention name] is provided in the [investigator’s brochure/package insert].

<End of example text>

## Benefit/Risk Assessment

- Refer to the master protocol for any platform-level benefit-risk assessments. Benefit-risk assessments related to the intervention or intervention-specific procedures or assessments should be added below.
- Study interventions information should align with the IB, package insert/prescribing information (if applicable) and investigational medicinal product dossier (IMPD) (if applicable).
- Consider the known and expected benefits and potential risks of the study intervention(s) in this intervention cohort. Cross reference Section 4 Study Design for the study intervention and dose information.
- There may be unplanned circumstances under which an intervention cohort may be terminated early, such as due to emerging safety information, poor clinical efficacy, a change in the benefit-risk assessment, or other emerging information. If desired, circumstances which would lead to the unplanned early termination of an intervention cohort may be added to the ISA in the appropriate sections. (Planned circumstances for early termination, such as futility analyses or meeting liver safety criteria, should also be addressed in the appropriate sections of the ISA.)
- If an intervention cohort is terminated early, a distinction should be made between termination of an intervention cohort and termination of the platform study. Termination of an intervention cohort does not necessarily mean termination of the platform study.

<Start of suggested text>

More detailed information about the known and expected benefits and risks and reasonably expected adverse events (AEs) of [study intervention name] may be found in the [investigator’s brochure (IB), participant information leaflet, package insert, development safety update report (DSUR) or summary of product characteristics].

<End of suggested text>

<Start of example text>

Should new information regarding the benefit and/or risk of [study intervention name] change the benefit-risk assessment for participants, the [study intervention name] cohort may be stopped early for all participants, including termination of enrollment of new participants, in consultation with safety monitors/committees, study representatives/sponsors, intervention owner, and other appropriate stakeholders. Termination of the [study intervention name] cohort does not necessarily mean termination of the platform study.

<End of example text>

### Risk Assessment

Table format given in example is recommended to address all requirements of EU reg Annex 1, Section D, 17d are met.

| **Potential Risk of Clinical Significance**  Briefly summarize only the relevant key risks for THIS intervention. | **Summary of Data/Rationale for Risk**  For applicable cells in this column, include a brief description or reference to IB Section [X].  Consider the guidance in the DSUR Evaluation of Risks Section 18.1 when taking an inventory of potential risk topics. | **Mitigation Strategy**  For applicable cells in this column, provide a brief description of strategies to mitigate identified risks or provide a crossreference to the relevant protocol Section (eg, inclusion/exclusion criteria, participant monitoring, withdrawal criteria, dose selection, comparison to nonclinical no effect levels, duration of dosing, etc). |
| --- | --- | --- |
| **Study Intervention (s) [Intervention]** | | |
| Nonclinical risks – concluding sections of the nonclinical assessment of safety (NCAS) should provide key nonclinical risks associated with the study intervention.  Clinical risks – consider DSUR (or text for marketed product) for key clinical risks  Also consider the IB, development core safety information (DCSI), as applicable. If applicable, include nonserious AEs of special interest. Crossreference Section 8.3 if appropriate |  |  |
| **Study Procedures** | | |
| Consider risks associated with the study design and procedures specific to the intervention being evaluated in this intervention cohort. |  |  |
| **Other** | | |
| Consider risks associated with comparators, challenge agents, imaging agents, etc. specific to the intervention being evaluated in this intervention cohort. |  |  |

### Benefit Assessment

The benefit assessment should be written from the perspective of an individual participant.

Benefit considerations may include:

- Potential benefit of receiving study intervention during the intervention cohort duration that may have clinical utility (if applicable)
- Contributing to the process of developing new therapies in an area of unmet need – this may be particularly relevant for Clinical Pharmacology studies
- Provision of nondrug therapy (eg, compression stockings) if applicable
- Medical evaluations/assessments associated with study procedures [eg, physical exam, ECG, labs, etc.] specific to the intervention being evaluated in this intervention cohort.

### Overall Benefit: Risk Conclusion

Provide a succinct concluding statement on the perceived balance between risks that have been identified from cumulative safety data, protocol procedures and anticipated efficacy/benefits within the context of the proposed intervention being evaluated in this intervention cohort. Risks need to be weighed against the benefits for the **individual participant.**

<Start of example text>

Taking into account the measures taken to minimize risk to participants participating in this intervention cohort, the potential risks identified in association with [study intervention] are justified by the anticipated benefits that may be afforded to participants with [indication].

<End of example text>

# Objectives, Endpoints and Estimands

The master protocol is the primary source of information for protocol endpoint registration on public registers (eg, ClinicalTrials.gov).

*Objectives*:

- Refer to the master protocol for objectives. Add additional intervention cohort specific objectives if applicable.
- Objectives and endpoints for specific therapeutic areas may be accessed in the therapeutic area libraries.

*Endpoints:*

- Refer to the master protocol for endpoints. Add additional intervention cohort specific endpoints if applicable.
- It is recommended that any intervention cohort specific objectives and endpoints be presented together in a table (see example for Platform design, where it is expected that primary endpoint is the same for all ISAs, but secondary and tertiary endpoints might differ) to ensure all endpoints are aligned with an objective. The table might have to be adapted for other design options like basket trials. \

| Objectives | Endpoints |
| --- | --- |
| Primary |  |
| - Refer to master protocol | - Refer to master protocol |
| Secondary | Secondary |
| - Refer to master protocol - Add any intervention-specific objectives | - Refer to master protocol - Add any intervention-specific endpoints |
| [Tertiary/Exploratory/Other] | [Tertiary/Exploratory/Other] |
| - Add any intervention-specific objectives | - Add any intervention-specific endpoints |

# Study Design

## Overall Design

- Do not include study schema.
- Do not include the SoA here.
- Use bullets rather than lengthy text, if possible.
- Refer to the master protocol for details of the study design.
- Study intervention assignment method (eg, randomization, stratification, both). Do NOT state block size. If assignment to intervention is by randomization, describe when randomization occurs relative to screening. Do not put sample size justification here. This is covered in Section 9 Statistical Considerations.
- Total duration of the intervention cohort participation for each participant with sequence and duration of study periods (eg, screening, run-in, fixed dose/titration, follow-up/washout periods).
- Describe any provisions for extending the study or entry to rollover studies (crossreference Section 6.6 Continued Access to Study Intervention after the End of the Study). Do not duplicate information.
- See therapeutic area libraries for additional guidance for studies in specific therapeutic areas.
- A protocol deviation is related to a data point or process identified in the protocol or documents referenced in the protocol (eg, laboratory manual). When designing the study, limit items that may generate deviations whenever possible. Reduce the number of reference documents to those essential for the conduct of the study.

## Scientific Rationale for Study Design

- Refer to the master protocol for the scientific rationale for the study design. .

### Participant Input into Design

Describe any participant involvement in the design of the clinical study and any participant suggestions implemented.

## Justification for Dose

Provide justification for the selection of the doses of all study interventions. Crossreference Section 6.5 Dose Modification as needed.

If the study design foresees dose escalation, describe the maximum allowed increase from one subset to the next and mention on which data it is based (eg, nonclinical studies).

<Start of example text>

In the phase I/II study [Study xxxx], participants with a variety of advanced solid tumors have been treated at multiple dose levels of single-agent [study intervention name ] or [study intervention name] in combination with [study intervention name] .Overall, single-agent [study intervention name] and [study intervention name] in combination with [study intervention name] were tolerated with safety profiles similar to those of other approved checkpoint inhibitors. A maximum tolerated dose was not identified for [study intervention name] . Anti-tumor activity was not observed with [study intervention name] as a single agent but was seen at several dose levels of [study intervention name] in combination with [study intervention name].There was no clear pattern between dose and anti-tumor activity. Therefore, a PK/PD modeling analysis was used to support determination of the recommended dose. The pharmacological criterion chosen to guide the selection was the ability to achieve 90% suppression of the target [TTTT] expressed in the tumor in > 90% of participants. Based on the study simulation of the PK/PD model, 2 recommended doses were declared for the combination: ### mg of [study intervention name] in combination with ### mg [study intervention name] and ### mg [study intervention name] in combination with spartalizumab 400 mg every 4 weeks.

An alternate dose regimen of ### mg [study intervention name] in combination with ### mg [study intervention name] was chosen for this study.

<End of example text>

Start of example text>

The doses and dosing regimens of [study intervention name] and [study intervention name] are chosen in this study to reflect doses and dosing regimens currently under consideration or evaluation in monotherapy NASH studies.

[Study intervention name] is currently being studied in NASH as monotherapy in Study xxxx. The doses of ### to ### µg [study intervention name] have been tested and were well tolerated in study xxxx, with an observed dose dependent reduction in liver fat content, ALT and GGT. Study xxxx biomarker data suggested that exposures of AUC > ###/mL should be explored. At a dose of ### µg for [study intervention name] 80% of NASH participants are expected to achieve this threshold and it is therefore chosen as the reference dose for the monotherapy and the combination arms in this study. The overall rate of AEs and SAEs was similar across treatment groups, with an increase in pruritus with [study intervention name] compared to placebo.

[Study intervention name] is currently being tested as ### mg and ### mg once-daily monotherapy in NASH (Study YYYY). An interim analysis showed promising reduction of ALT, AST, GGT, body weight, waist circumference, hemoglobin A1c and liver fat. The majority of AEs reported were GI in nature, specifically mild diarrhea. The incidence of diarrhea was similar between the placebo and ### mg treatment groups.

A range of doses from ### mg to ### mg of [study intervention name] have been tested in obese participants (both Japanese and non-Japanese). In obese Japanese participants over 12 weeks of treatment with placebo, ### mg, ### mg, or ### mg once-daily [study intervention name] ### mg appeared to be at the plateau of the observed dose-body weight loss curve. However, the rate of diarrhea events increased largely between ### mg and ### mg from about 14% to about 39% [Study ZZZZ]. In obese non-Japanese participants over 24 weeks of treatment with placebo, ### mg, ### mg, or ### mg once-daily [study intervention name], ### mg appeared to be near the plateau of the observed dose-body weight loss curve, and the incidence of diarrhea increased from about 16% to about 55% in the ### mg and ### mg treatment groups, respectively [Study YYYY]. Thus ### mg of [study intervention name] is expected to have a similar body weight loss with considerably lower diarrhea incidence compared to ### mg and is therefore preferred. Assuming that data from obese participants are indicative of NASH participants for both body weight loss and diarrhea events, as suggested by the ### mg dose (with diarrhea events of ##/## versus ##/## for NASH vs obese participants), ### mg is selected for this combination.

In summary, the doses of [study intervention name] for this study were selected based on the expectation of achieving increased efficacy with the combination therapy, compared to individual monotherapies, while maintaining tolerability and safety of the participants.

<End of example text>

## End of Study Definition

Include an end of intervention cohort definition.

Refer to the master protocol for the end of study definition.

Determine follow-up if participants complete the intervention cohort.

<Start of suggested text>

A participant is considered to have completed the intervention cohort if the participant has completed all periods of the intervention cohort including [the last visit] or [the last scheduled procedure shown in the intervention-specific SoA].

The end of the intervention cohort is defined at the date of the last visit of the last participant in the intervention cohort or last scheduled procedure shown in the intervention-specific SoA.

Refer to the master protocol for the end of the platform study definition.

<End of suggested text>

# Study Population

Criteria should be numbered according to company process.

Refer to the master protocol for the entry criteria.

Consider including only intervention-specific entry criteria; alternatively, the master protocol entry criteria could be repeated followed by intervention cohort specific entry criteria.

<Start of common text>

Prospective approval of protocol deviations to recruitment and enrollment criteria, also known as protocol waivers or exemptions, is not permitted.

<End of common text>

## Inclusion Criteria

General Points:

- List any additional criteria necessary for participation in the intervention cohort. Ensure that each criterion can be easily assessed on the basis of measurable data and answered with yes/no responses.
- The use of double negatives should be avoided (eg, no indication of prior noncompliance with the intervention regimen).
- Consider numbering conventions for intervention-specific inclusion and exclusion criteria. They will need to distinguish between master and intervention cohort specific criteria and among different intervention cohort inclusion and exclusion criteria.
- <Start of suggested text>

All participants must be eligible according to the master protocol inclusion criteria. Participants are eligible to be included in the intervention cohort only if all of the additional criteria below apply.

<End of suggested text>

**Intervention-specific Criteria**

Additional inclusion criteria for intervention cohort 1 are listed below and start with INCL01.

| Contraceptive/Barrier Requirements |
| --- |
| <Start of common text>  If there are no contraceptive requirements in the intervention cohort, remove the statement “Contraceptive use by men and women …”. Modify as appropriate based upon inclusion of men and women.  INCL01-01 Contraceptive use by [men and women] should be consistent with local regulations regarding the methods of contraception for those participating in clinical studies.   1. Male participants:   See participant libraries for common text to include here.   1. Female participants:   INCL01-02 A woman of childbearing potential must have a negative serum β human chorionic gonadotropin β hCG) at [*specify timepoint*]. A woman is considered of childbearing potential (fertile) from the time of menarche until becoming postmenopausal unless permanently sterile.  See participant libraries for common text to include here.  <End of common text> |
| Informed Consent |
| <Start of common text>  INCL01-03 Capable of giving signed informed consent as described in Appendix 1 of the Master protocol which includes compliance with the requirements and restrictions listed in the informed consent form (ICF) and in this ISA protocol. Participants must consent at 2 levels, and sign an ICF for both the master protocol and the applicable ISA.  <End of common text> |

## Exclusion Criteria

List any additional exclusion criteria necessary for participation in the intervention cohort.

All participants must be eligible according to the master protocol exclusion criteria. Participants are excluded from the intervention cohort if any of the additional criteria below apply:

**Intervention-specific Criteria**

Additional exclusion criteria for intervention cohort 1 are listed below and start with EXCL01.

| **Prior/Concomitant Therapy** |
| --- |
| EXCL01-01 |
| **Prior/Concurrent Clinical Study Experience** |
| EXCL01-02 |
| **Diagnostic assessments** |
| EXCL01-03 |
| **Other Exclusions**  EXCL01-04 |

## Lifestyle Considerations

Refer to the master protocol for lifestyle considerations. Add additional intervention cohort specific information if applicable. Use bullets rather than numbers to list lifestyle considerations.

If this section is not applicable, include a statement that no restrictions are required. Do not omit section.

If applicable, describe any of the lifestyle considerations (diet, smoking habits, alcohol, or recreational drug consumption, etc) that could be of relevance for the intervention cohort and any restrictions during any of the study periods. For example, include a statement about exposure to sunlight for study interventions with photosensitivity potential.

Level 3 headings may not be applicable for all studies (eg, vaccines).

### Meals and Dietary Restrictions

- Food and drink restrictions before the start of pharmacokinetic (PK) sample collections.
- Timing of meals relative to dosing.
- Ensure consistency in this section with other parts of the protocol and crossreference other sections (eg, exclusion criteria) as needed.
- If the exact timing of meals is listed in the SoA, do not repeat this information here. Instead, include a reference to the SoA.

<Start of suggested text>

Refer to the master protocol for meal and dietary restrictions. [If applicable: Additional intervention cohort specific restrictions are listed below.]

- Refrain from consumption of red wine, Seville oranges, grapefruit or grapefruit juice, [pomelos, exotic citrus fruits, grapefruit hybrids, or fruit juices] from [X days] before the start of study intervention until after the final dose.

<End of suggested text>

### Caffeine, Alcohol, and Tobacco

- Restrictions are dependent on the known metabolism of the study intervention to eliminate any potential for PK interactions and possible effects of caffeine- and xanthine-containing products on ECG results or other pharmacodynamic endpoints (eg, blood pressure).
- The possible effects of alcohol on PK, pharmacodynamic interactions, or laboratory parameters, such as liver function tests, should also be addressed by restrictions in this section.

<Start of suggested text>

Refer to the master protocol for caffeine, alcohol, and tobacco restrictions. If applicable: Additional intervention cohort specific restrictions are listed below.]

- During each dosing session, participants will abstain from ingesting caffeine- or xanthine-containing products (eg, coffee, tea, cola drinks, and chocolate) for [X hours] before the start of dosing until after collection of the final pharmacokinetic (PK) and/or pharmacodynamic sample.
- During each dosing session, participants will abstain from alcohol for 24 hours before the start of dosing until after collection of the final PK and/or pharmacodynamic sample.
- Participants who use tobacco products will be instructed that use of nicotine-containing products (including nicotine patches) will not be permitted while they are in the clinical unit. [OR] Use of tobacco products will not be allowed from [screening/the start of dosing] until after the final follow-up visit.

<End of suggested text>

### Activity

Intervention cohort specific restrictions may apply depending on the nature and frequency of assessments (eg, activity may be further restricted by ensuring participants remain in bed for 4 to 6 hours after dosing) or for studies with interventions known to cause photosensitivity, activities such as sunbeds may be restricted.

<Start of suggested text>

Refer to the master protocol for activity restrictions. [If applicable: Additional intervention cohort specific restrictions are listed below.]

- Participants will abstain from strenuous exercise for [X hours] before each blood collection for clinical laboratory tests. Participants may participate in light recreational activities during studies (eg, watching television, reading).

<End of suggested text>

## Screen Failures

Refer to the master protocol for screen failure information. Participants must meet all screening criteria in the master protocol to be screened for an intervention cohort. If a participant meets the criteria of the master protocol but does not meet the screening criteria for an intervention cohort, determine if they are eligible to screen for a different intervention cohort in the same program.

State whether rescreening is permitted. If rescreening is permitted within the same intervention cohort, state the entry criteria/parameters that can be reassessed for individuals who previously failed screening and the time period for repeating procedures/rescreening. Individual inclusion/exclusion criteria may also state whether a repeat procedure is allowed without being considered a rescreen.

<Start of suggested text>

Refer to the master protocol for screen failure considerations. Participants who meet the entry criteria for inclusion per the master protocol but do not meet the entry criteria for participation in this intervention cohort [may/may not] be rescreened to another intervention cohort.

<End of suggested text>

## Criteria for Temporarily Delaying [Enrollment/Randomization/Administration of Study Intervention]

Insert suggested text from participant or therapeutic libraries if relevant.

# Study Intervention(s) and Concomitant Therapy

<Start of common text>

Study intervention is defined as any investigational intervention(s), marketed product(s), or placebo, intended to be administered to a study participant according to the ISA protocol.

<End of common text>

## Study Intervention(s) Administered

- It is preferred that interventions are described in a table and that text be minimized.
- The precise interventions or diagnostic agents to be administered in each arm of the study and for each period of the study should be described including route and mode of administration, dose, and dosage regimen and duration of intervention.
- Include information for all study interventions (eg, placebo, comparators, background medication, rescue medication). If any interventions or diagnostic agents will be provided by the intervention owner, consider adding details.
- All interventions must be designated as an investigational medicinal product (IMP) or NIMP/AxMP. If uncertain as to whether an intervention is an IMP or an NIMP/AxMP, refer to definitions provided below or to current EU guidance on IMP and NIMP/AxMP.
  - An IMP is a pharmaceutical form of an active substance or placebo being tested or used as a reference in a clinical study, including products already with a marketing authorization but used or assembled (formulated or packaged) in a way different from the authorized form, or when used for an unauthorized indication, or when used to gain further information about the authorized form. Medicinal products with a marketing authorization are IMPs when they are to be used as the test substance, reference substance, or comparator in a clinical study, provided the requirement(s) in the definition is/are met.
  - An NIMP/AxMP is a medicinal product that is not classified as an IMP in a study, but may be taken by participants during the study, eg, concomitant or rescue/escape medication used for preventative, diagnostic, or therapeutic reasons or medication given to ensure that adequate medical care is provided for the participant during a study.

**The tables should be modified as needed. Instructions for the tables are as follows:**

Table 1:

- Intervention Label: Unique identifier to represent the intervention and its related characteristics. It will be used to populate the Associated Intervention Labels cell in the Arms table.
- Intervention Name: Enter a generic (INN) or trade name if required as per CMC, if applicable.
- Intervention Description: Include details that can be made public about the intervention, sufficient to distinguish the intervention from other similar interventions studied in the same or another clinical study. For example, interventions involving drugs may include dosage form, dosage, frequency, and duration. Limit: 1000 characters.
- Type: Select one option from this list:
  - Drug: Including placebo
  - Biological/Vaccine
  - Procedure/Surgery
  - Radiation
  - Behavioral: For example, psychotherapy, lifestyle counseling
  - Genetic: Including gene transfer, stem cell and recombinant DNA
  - Dietary Supplement: For example, vitamins, minerals
  - Combination Product: Combining a drug and biological product
  - Diagnostic Test: For example, imaging, in-vitro
  - Other: Edit as appropriate
- Dose Formulation: Select one option from the list:
  - Tablet
  - Ampule
  - Capsule
- Unit Dose Strength(s): Include dose strength information
- Dosage Level(s): Include dose amount and frequency
- Route of Administration: Select one option from the list:
  - Oral
  - IM
  - IV infusion
  - IV injection
- Use: Select one of the following:
  - Experimental
  - Placebo Comparator
  - Active Comparator
  - Sham Comparator
  - Rescue Medication
  - Background Intervention
  - Challenge Agent
  - Diagnostic
  - Other.
- Definition of IMP and NIMP/AxMP is based on guidance issued by the European Commission. Regional and/or country differences in the definition of IMP/NIMP/AxMP may exist. In these circumstances, local legislation is followed.
- Sourcing: Include sourcing-related information, eg, centrally by intervention owner or locally by the study site, subsidiary, or designee.
- Packaging and Labeling: Include information on how the study intervention will be packaged and labeled.

Current/Former Names(s) or Alias(es): If applicable, add current and former name(s) or alias(es), if any, different from the intervention name(s) that the intervention owner has used publicly to identify the intervention(s), including, but not limited to, past or present names such as brand name(s) or serial number(s).

**Table 1. Study Intervention(s) Administered**

| **Intervention Label** |  |  |  |  |
| --- | --- | --- | --- | --- |
| **Intervention Name** | [Generic (or trade name if required) as per CMC, if applicable, or intervention owner number] | [Generic (or trade name if required) as per CMC, if applicable, or intervention owner number] | [Placebo] | [Any additional products provided as part of the study including rescue medications or challenge agent] |
| **Intervention Description** | [eg, dosage form, dosage, frequency] | [eg, dosage form, dosage, frequency] | [eg, dosage form, dosage, frequency] | [eg, dosage form, dosage, frequency] |
| **Type** | [drug/biologic] | [drug/biologic] | [drug/biologic] | [drug/biologic] |
| **Dose Formulation** | [tablet/ampule/capsule] | [tablet/ampule/capsule] | [tablet/ampule/capsule] | [tablet/ampule/capsule] |
| **Unit Dose Strength(s)** | [dose strength of the product ie, each unit] | [dose strength of the product ie, each unit] | [dose strength of the product ie, each unit] | [dose strength of the product ie, each unit] |
| **Dosage Level(s)** | [dose amount and frequency] | [dose amount and frequency] | [dose amount and frequency] | [dose amount and frequency] |
| **Route of Administration** | [oral/IM/IV infusion/IV injection] | [oral/IM/IV infusion/IV injection] | [oral/IM/IV infusion/IV injection] | [oral/IM/IV infusion/IV injection] |
| **Use** | [experimental, placebo, active comparator, sham comparator, rescue medication, background intervention, challenge agent, diagnostic, or other] | [experimental, placebo, active comparator, sham comparator, rescue medication, background intervention, challenge agent, diagnostic, or other] | [experimental, placebo, active comparator, sham comparator, rescue medication, background intervention, challenge agent, diagnostic, or other] | [experimental, placebo, active comparator, sham comparator, rescue medication, background intervention, challenge agent, diagnostic, or other] |
| **IMP and NIMP/AxMP** | IMP or NIMP | IMP or NIMP | IMP or NIMP | IMP or NIMP |
| **Sourcing** | [Insert/modify as appropriate: Provided centrally by the intervention owner or locally by the study site, subsidiary, or designee]. | [Insert/modify as appropriate: Provided centrally by the intervention owner or locally by the study site, subsidiary, or designee]. | [Insert/modify as appropriate: Provided centrally by the intervention owner or locally by the study site, subsidiary, or designee. | [Insert/modify as appropriate: Provided centrally by the intervention owner or locally by the study site, subsidiary, or designee. |
| **Packaging and Labeling** | Study intervention will be provided in [container]. Each [container] will be labeled as required per country requirement | Study intervention will be provided in [container]. Each [container] will be labeled as required per country requirement | Study intervention will be provided in [container]. Each [container] will be labeled as required per country requirement | Study intervention will be provided in [container]. Each [container] will be labeled as required per country requirement |
| [Current/Former Name(s) or Alias(es)] | Current/former name(s) or alias(es) | Current/former name(s) or alias(es) | Current/former name(s) or alias(es) | Current/former name(s) or alias(es) |

Table 2:

- Arm Title: Please enter 1 arm name per column (this name should be used consistently across all related documents).
- Arm Type: Role of each arm in the clinical study. Select one option from this list:
  - Experimental
  - Placebo Comparator
  - Active Comparator
  - Sham Comparator
  - No Intervention
- Arm Description: If needed, additional descriptive information (including which interventions are administered in each arm) can be added to differentiate each arm from other arms in the clinical study. Limit: 1000 characters. Delete row if not required
- Associated Intervention Labels: List all the interventions to be administered in each arm exactly as listed in the first table.

**Table 2. Study Arm(s)**

| **Arm Title** | Enter Arm name | Enter Arm name | Enter Arm name |
| --- | --- | --- | --- |
| **Arm Type** | [experimental, placebo, active comparator, sham comparator, no intervention, or other] | [experimental, placebo, active comparator, sham comparator, no intervention, or other] | [experimental, placebo, active comparator, sham comparator, no intervention, or other] |
| **[Arm Description]** | [eg, Participants will receive [X] 20 mg BID on Day 1 of each 21-day cycle. [Z] will be administered on Day 1 for 4 cycles.] | [eg, Participants will receive [X] 20 mg BID on Day 1 of each 21-day cycle. [Z] will be administered on Day 1 for 4 cycles.] | [eg, Participants will receive [X] 20 mg BID on Day 1 of each 21-day cycle. [Z] will be administered on Day 1 for 4 cycles.] |
| **Associated Intervention Labels** |  |  |  |

## Preparation, Handling, Storage, and Accountability

Instructions for the preparation of study interventions, including assembly of devices, should be provided (eg, reconstitution, mixing). If the instructions are lengthy or complicated, it is acceptable to reference the label (if applicable) or include them as an appendix to the protocol or as a separate document(s) provided to the site (eg, pharmacy manual). If provided to the site as a separate document(s), this should be noted in this section.

<Start of common text>

- The investigator or designee must confirm appropriate temperature conditions have been maintained during transit for all study intervention received, and any discrepancies are reported and resolved before use of the study intervention.
- Only participants enrolled in this intervention cohort may receive study intervention, and only authorized site staff may supply or administer study intervention. All study intervention must be stored in a secure, environmentally controlled, and monitored (manual or automated) area in accordance with the labeled storage conditions with access limited to the investigator and authorized site staff.
- The investigator, institution, or the head of the medical institution (where applicable) is responsible for study intervention accountability, reconciliation, and record maintenance (ie, receipt, reconciliation, and final disposition records).
- Further guidance and information for the final disposition of unused study interventions are provided in the [study reference manual or other specified location].

<End of common text>

## Measures to Minimize Bias: Randomization and Blinding

- Refer to the master protocol for randomization and blinding considerations. If an intervention cohort has multiple treatment arms and there is a second level of randomization, describe here.

## Study Intervention Compliance

- The measures that will be taken to ensure and document intervention compliance should be described (eg, intervention accountability records, diary cards, intervention concentration measurements, or medication event monitoring). May include the use of electronic data capture.
- Consider any implications of under/overdosing and cross reference Section 8.4 Pharmacokinetics if required.
- Teams should choose the appropriate wording from the options provided and delete the wording not used.

<Start of suggested text for studies using bulk supplies>

When the individual dose for a participant is prepared from a bulk supply, the preparation of the dose will be confirmed by a second member of the study site staff.

<End of suggested text for studies using bulk supplies>

<Start of suggested text when participants are dosed at the site>

When participants are dosed at the site, they will receive study intervention directly from the investigator or designee, under medical supervision. The date and time of each dose administered in the clinic will be recorded in the source documents. The dose of study intervention and study participant identification will be confirmed at the time of dosing by a member of the study site staff other than the person administering the study intervention. [Study site staff will examine each participant’s mouth to ensure that the study intervention was ingested.]

<End of suggested text when participants are dosed at the site>

<Start of suggested text for study intervention(s) administered at home>

When participants self-administer study intervention(s) at home, compliance with study intervention will be assessed at each visit. Compliance will be assessed by [direct questioning, counting returned tablets/capsules, etc] during the site visits and documented in the source documents and relevant form. Deviation(s) from the prescribed dosage regimen should be recorded.

A record of the quantity of [insert study intervention(s)] dispensed to and administered by each participant must be maintained and reconciled with study intervention and compliance records. Intervention start and stop dates, including dates for intervention delays and/or dose reductions will also be recorded.

<End of suggested text for study intervention(s) administered at home>

## Dose Modification

- Procedures to be used for selecting/modifying each participant's dose of study intervention should be described. Cross reference Section 4.3 Justification for Dose as needed and do not repeat information already provided in that section. These procedures can vary from simple random assignment to a selected fixed intervention/dosage regimen to the use of a specified titration procedure or more elaborate response/toxicity-determined dose modification procedures (eg, dose is titrated upward at intervals until intolerance or some specified endpoint is achieved).
- Do not include information on stopping study intervention for individual participants due to safety/other reasons as this is detailed in Section 7 Discontinuation of Study Intervention and Participant Discontinuation/Withdrawal.
- If dose selection/modification decisions are dependent upon review by a committee, include details in Appendix [10.1.5 Committees Structure] of the master protocol and make a cross reference here.
- Consider providing information in tabular format for simplicity.

<Start of example text that can be used with study designs that incorporate dose adjustment decisions>

This protocol allows some alteration from the currently outlined dosing schedule, but the [maximum daily dose and/or (predicted) maximum/cumulative exposure] will not exceed [X].

OR

The decision to proceed to the next dose level of [X] (either an increase or a decrease) will be made by the study team [and the investigator] based on safety, tolerability, and preliminary [PK and/or pharmacodynamic] data obtained in at least [X] participants at the prior dose level.

OR

The dosing schedule may be adjusted to expand a dosing cohort to further evaluate [safety, PK and/or pharmacodynamic] findings at a given dose level or to add cohorts to evaluate [up to X] additional dose levels. The study procedures for these additional participant(s)/cohort(s) will be the same as that described for other study participants/cohorts.

OR

Dose escalation will be temporarily halted and no further participants will be dosed until completion of a full safety review if:

- Moderate or severe AEs are consistently observed across participants in a cohort
- Unacceptable pharmacological effects that are reasonably attributable to [study intervention] in the opinion of the investigator are observed in more than [X]% of the participants in a cohort

Relevant reporting and discussion with the medical monitor, relevant [X] personnel, and the IRB/IEC will take place before resumption of dosing.

OR

If the same SAE occurs in more than [X] participants in a cohort, then dose escalation will be temporarily halted and no further participants will be dosed until a full safety review of the data has taken place. Relevant reporting and discussion with the medical monitor, relevant [X] personnel, and the IRB/IEC will take place before resumption of dosing.

The above criteria will apply even if measured PK parameters are below the prespecified PK stopping criteria, and every effort will be made to take a blood sample at the time of the AE for PK analysis.

<End of example text that can be used with study designs that incorporate dose adjustment decisions >

If applicable, procedures for back-titration or dose reductions for toxicity should be described.

If a dose reduction is necessary, the study intervention will be administered as follows: [insert text or a table describing changes].

### Retreatment Criteria

This section may be required in certain types of studies, eg, medical aesthetics. Any retreatment criteria needed after temporary discontinuation should be addressed in Section 7.1.4 Rechallenge.

<Start of suggested text>

All participants entered into the study will be treated at [Day X]. A participant may receive additional study interventions if the participant meets retreatment criteria as determined by the investigator and agrees to be retreated. Throughout the study, study intervention will be [blinded/unblinded].

After [Day X], the participant must meet all of the following criteria to be eligible for retreatment:

- [Criterion 1]
- [Criterion 2]

<End of suggested text>

## Continued Access to Study Intervention after the End of the Study

Include planned extension studies or possibilities for continued access to study intervention, if any, beyond completion of the intervention cohort. Continued access should be clearly defined – differentiate between intervention cohort-level and participant-level access.

If there is no intervention following the end of the intervention cohort, then text should be included to state that this is the case. Describe any additional care that will be provided to participants after they complete or discontinue the intervention cohort if this differs from what is normally expected for their condition.

## Treatment of Overdose

- Specify what is meant by study intervention overdose and any known antidote or nondrug therapies (see suggested text).
- Although clinical experience with overdose is often limited in early phases of development, provide any available project-specific guidance and information; however, ensure consistency with and avoid unnecessary duplication with any overdose information in the IB/package insert. Crossreference these documents if appropriate.
- Refer the investigator to the approved product label of the comparator (as applicable) for advice on overdose.

<Start of suggested text>

For this intervention cohort, any dose of [study intervention] greater than [insert daily dose of study intervention] within a [24-hour] time period [± X hours] will be considered an overdose.

[Intervention owner] does not recommend specific treatment for an overdose.] OR [The antidote to study intervention is X] and may be used in case of an overdose.

<End of suggested text>

<Start of common text>

In the event of an overdose, the [investigator/treating physician] should:

- Contact the medical monitor immediately.
- Evaluate the participant to determine, in consultation with the medical monitor, whether study intervention should be interrupted or whether the dose should be reduced.
- Closely monitor the participant for any AE/SAE and laboratory abnormalities [until [study intervention] can no longer be detected systemically (at least [X] days)].
- [Obtain a plasma sample for PK analysis within [X] days from the date of the last dose of study intervention if requested by the medical monitor (determined on a case-by-case basis)].
- [Document the quantity of the excess dose as well as the duration of the overdose.]

<End of common text>

## Concomitant Therapy

- Refer to the master protocol for concomitant therapy considerations. Add additional intervention cohort specific considerations if applicable.
- Describe which interventions or procedures will be allowed before and during the study and any other specific rules and procedures related to permitted or prohibited concomitant therapy. If this list is lengthy consider including details in an appendix and cross-referencing here.

### Rescue Medicine

If rescue therapy is permitted, consider using the suggested text provided.

The efficacy section should address when endpoints (eg, pain scores) are to be assessed with respect to dosing of rescue medication if relevant.

<Start of suggested text>

The study site [will/will not] supply [specify type] rescue medication that will be [provided by the intervention owner/obtained locally]. The following rescue medications may be used:

- [X]
- [X]

Although the use of rescue medications is allowable [at any time during the study], the use of rescue medications should be delayed, if possible, for at least [insert timeframe] following the administration of study intervention. The date and time of rescue medication administration as well as the name and dosage regimen of the rescue medication must be recorded.

<End of suggested text>

# Discontinuation of Study Intervention and Participant Discontinuation/Withdrawal

Details should be kept at a minimum here. Include any actions to be taken if certain events are observed in an appendix and cross reference that appendix as needed. Use schematics/algorithms if possible.

## Discontinuation of Study Intervention

Refer to the master protocol for discontinuation of study intervention. Add additional intervention cohort specific considerations if applicable.

Add additional intervention cohort specific considerations if applicable.

### Liver Chemistry Stopping Criteria

Refer to the master protocol for liver chemistry stopping criteria. Add additional intervention cohort specific criteria if applicable. Protocol authors should carefully evaluate if the liver-related stopping criteria are appropriate for the participant population and class of therapy evaluated and modify them, if needed.

<Start of common text for liver injury>

Discontinuation of study intervention for abnormal liver tests is required by the investigator when a participant meets one of the conditions outlined [in the algorithm] or in the presence of abnormal liver chemistries not meeting protocol-specified stopping rules if the investigator believes that it is in best interest of the participant.

Insert appropriate algorithm from relevant library.

<End of common text for liver injury>

### QTc Stopping Criteria

Refer to the master protocol for QTc stopping criteria. Add additional intervention cohort specific guidance if applicable. Insert appropriate text from relevant library

<Start of common text for cardiac changes>

If a clinically significant finding is identified (including, but not limited to changes from baseline in QT interval corrected using [Bazett’s formula [QTcB] or Fridericia’s formula [QTcF]]) after enrollment, the investigator or qualified designee will determine if the participant can continue in the study and if any change in participant management is needed. This review of the ECG printed at the time of collection must be documented. Any new clinically relevant finding should be reported as an AE.

<End of common text for cardiac changes>

### Temporary Discontinuation

Include specifics around criteria for interrupting study intervention, what to do if the participant needs to stop study intervention, whether they will continue in the intervention cohort, and whether all or specify which evaluations will be performed for the stated duration of the intervention cohort. Details of any rechallenge after a safety related event should be included in Section 7.1.4 Rechallenge.

### Rechallenge

Include specifics around rechallenge, criteria for restarting study intervention, what to do if the participant needs to stop study intervention, whether they will continue in the intervention cohort, number of rechallenges allowed during the intervention cohort, and whether all or specify which evaluations will be performed for the stated duration of the intervention cohort. Example of language to use for rechallenge after a liver event are provided below and should be used if required.

#### Study Intervention Restart or Rechallenge After Liver Stopping Criteria Are Met

This section does not apply to single dose studies.

<Start of common text if restart/rechallenge is NOT allowed>

Study intervention restart or rechallenge after liver chemistry stopping criteria are met by any participant in this study are not allowed.

<End of common text if restart/rechallenge is NOT allowed>

<Start of common text if restart/rechallenge IS allowed>

Study intervention [restart/rechallenge] after liver chemistry stopping criteria are met is allowed in this intervention cohort. If the participant meets liver chemistry stopping criteria do not [restart/rechallenge] the participant with study intervention unless:

- [Intervention owner board] approval **is granted**
- Ethics and/or IRB approval is obtained, if required, and
- Separate consent for intervention [restart/rechallenge] is signed by the participant

NOTE: If study intervention was interrupted for suspected intervention-induced liver injury, the participant should be informed of the risk of death, liver transplantation, hospitalization, and jaundice and reconsented before resumption of dosing.

Refer to Appendix 6: Liver Safety: Suggested Actions and Follow-up Assessments [and Study Intervention Rechallenge Guidelines] in the master protocol for details on the [restart/rechallenge] process.

If [intervention owner board] approval to restart/rechallenge the participant with study intervention is **not granted**, then the participant must permanently discontinue study intervention and may continue in the study for protocol-specified follow‑up assessments.

<End of common text if restart/rechallenge IS allowed>

## Participant Discontinuation/Withdrawal from the Study

<Start of common text>

Refer to the master protocol for considerations regarding participant discontinuation/withdrawal from the study. For considerations regrading discontinuation of study intervention, refer to Section 7.1.

<End of common text>

## Lost to Follow-up

<Start of common text>

Refer to the master protocol for considerations regarding participants lost to follow-up.

<End of common text>

# Study Assessments and Procedures

- Refer to the master protocol for study assessments and procedures. Add additional intervention cohort specific assessments and procedures if applicable.

<Start of common text>

Refer to the master protocol for study assessments and procedures. Study procedures and their timing are summarized in the SoA, including any additional intervention cohort specific assessments and procedures.

<End of common text>

## [Efficacy and/or Immunogenicity] Assessments

- Refer to the master protocol for efficacy/immunogenicity assessments. Add additional intervention cohort specific assessments if applicable.

## Safety Assessments

- Refer to the master protocol for safety assessments. Add additional intervention cohort specific assessments if applicable.

### Physical Examinations

- Refer to the master protocol for physical examination assessments. Add additional intervention cohort specific assessments if applicable.

### Vital Signs

- Refer to the master protocol for vital sign assessments. Add additional intervention cohort specific assessments if applicable.

### Electrocardiograms

- Refer to the master protocol for ECG assessments. Add additional intervention cohort specific assessments if applicable.

### Clinical Safety Laboratory Assessments

- Refer to the master protocol for clinical safety laboratory assessments. Add additional intervention cohort specific assessments if applicable.

### Pregnancy Testing

- Refer to the master protocol for pregnancy testing. Add additional intervention cohort specific pregnancy testing if applicable.

### Suicidal Ideation and Behavior Risk Monitoring

Clinical studies meeting either of the following 2 criteria must include appropriate assessments (eg, Columbia-Suicide Severity Rating Scale [C-SSRS]) to enable the prospective monitoring of suicidal ideation and behavior (SIB) in individual participants:

1. Participant or healthy volunteer studies using compounds that:

- are known to be active in the human central nervous system (CNS), or
- are being studied for CNS activity, or
- are being developed for any psychiatric or neurologic indication, or
- may affect mood, cognition, or behavior via their effects on the CNS (directly or indirectly), or
- are pharmacologically similar to medicines that have had SIB reported in association with their use, which is considered to be at least possibly causally associated (eg, isotretinoin and other tretinoins, beta blockers, reserpine, smoking cessation medicines and medicines for weight loss).

2. Studies including any participant population with an elevated risk of SIB, which may manifest during the study, and for which monitoring of SIB is considered to be in the best interest of participant safety and/or science.

Notes:

- Determination and documentation is made at a program level on a company-specific basis according to their practices. Assessment of SIB is difficult in participants with cognitive impairment of a degree that interferes with understanding of the concept of suicide (eg, Alzheimer’s disease, other dementias, learning disability, autism), and in participants who are terminally/critically ill.  It is therefore reasonable to omit in these circumstances. If omission of SIB assessment is being considered for studies in challenging populations that would otherwise meet the criteria for monitoring, regulatory authority approval should be sought prior to protocol approval.
- Young children may not have reached sufficient cognitive maturity to understand the concept of death. As there is also no validated instrument for the prospective monitoring of SIB in children less than 7 years of age, all proposed studies in children less than 7 years that meet the criteria for monitoring of SIB should therefore be referred for regulatory and company’s internal review/advisory board for approval prior to protocol approval.
- It should be recognized that in uncontrolled studies, scientific interpretation of the results of monitoring of SIB may be difficult or impossible. Even so, if monitoring is important for participant safety it may be included.

<Start of example text>

[STUDY INTERVENTION/ACTIVE COMPARATOR] is considered to be a CNS-active intervention.

AND/OR:

[STUDY INTERVENTION/ACTIVE COMPARATOR] is related to products with an increased risk of suicidal ideation or behavior.

AND/OR:

Participants with [CONDITION] may occasionally develop suicidal ideation or behavior.

<End of example text>

<Start of suggested text>

Participants being treated with [study intervention X] should be monitored appropriately and observed closely for suicidal ideation and behavior (SIB) or any other unusual changes in behavior, especially at the beginning and end of the course of intervention, or at the time of dose changes, either increases or decreases. Participants who experience signs of SIB should undergo a risk assessment. All factors contributing to SIB should be evaluated and consideration should be given to discontinuation of the study intervention.

If study design calls for family and caregiver input, specify the need to communicate to these parties. For wording to be used in pediatric studies see the pediatric participant library.

Specify how, if in the event of suicidal ideation or behavior, information will be shared with the legal guardian or others, including mental health professionals (local regulations should be followed). Address in the informed consent and assent forms as appropriate.

When informed consent or assent has been given, families and caregivers of participants being treated with [study intervention X] should be alerted about the need to monitor participants for the emergence of unusual changes in behavior, as well as the emergence of suicidal ideation and behavior and to report such symptoms immediately to the study investigator.

[Baseline assessment of suicidal ideation and behavior/intervention emergent suicidal ideation and behavior] will be monitored during [study identifier] using [name of scale].

<End of suggested text>

## Adverse Events (AEs), Serious Adverse Events (SAEs), and Other Safety Reporting

- Refer to the master protocol for safety reporting. Add additional intervention cohort specific information if applicable.

### Time Period and Frequency for Collecting AE and SAE Information

- Refer to the master protocol. Add additional intervention cohort specific information if applicable.

### Method of Detecting AEs and SAEs

- Refer to the master protocol. Add additional intervention cohort specific information if applicable.

### Follow-up of AEs and SAEs

- Refer to the master protocol. Add additional intervention cohort specific information if applicable.

### Regulatory Reporting Requirements for SAEs

- Refer to the master protocol. Add additional intervention cohort specific information if applicable.

### Pregnancy

- Define the time period for collecting pregnancy information for female participants or female partners of male participants as appropriate. This should align with the time period for postintervention contraception as described in Section 5.1.
- Do not collect pregnancy information for female participants known to be pregnant during the screening phase or before exposure to study intervention unless these participants enter the study in which case consider whether pregnancy history needs to be collected.
- Specify any additional actions required (discontinuation of study intervention, withdrawal from the study), and any assessments that need to be performed.

<Start of common text>

- Details of all pregnancies in [female participants and, if indicated, female partners of male participants] will be collected after the start of study intervention and until [time period for reporting pregnancies should align with the time period for postintervention contraception determined in Section 5.1].
- If a pregnancy is reported, the investigator will record pregnancy information on the appropriate form and submit it to the intervention owner [within 24 hours] of learning of the [female participant or female partner of male participant (after obtaining the necessary signed informed consent from the female partner)] pregnancy.
- While pregnancy itself is not considered to be an AE or SAE, any pregnancy complication or elective termination of a pregnancy for medical reasons will be reported as an AE or SAE.
- Abnormal pregnancy outcomes (eg, spontaneous abortion, fetal death, stillbirth, congenital anomalies, ectopic pregnancy) are considered SAEs and will be reported as such.
- The [participant/pregnant female partner] will be followed to determine the outcome of the pregnancy. The investigator will collect follow-up information on the [participant/pregnant female partner] and the neonate and the information will be forwarded to the intervention owner.
- Any post-study pregnancy-related SAE considered reasonably related to the study intervention by the investigator will be reported to the intervention owner as described in Section 8.3.4. While the investigator is not obligated to actively seek this information in former study participants [/pregnant female partner], the participant may learn of an SAE through spontaneous reporting.
- Any female participant who becomes pregnant while participating in the study [will discontinue study intervention or be withdrawn from the study] OR [may request continuation of study intervention.]

<End of common text>

Should a female participant become pregnant during the course of a study, under certain circumstances the study design may allow for the continuation of study intervention. In these instances, ICH guidelines and local regulations must be observed, and appropriate justification given in Section 4.2 – Scientific Rationale for Study Design. In the absence of such justification female participants who become pregnant must be discontinued from study intervention.

Justification for continuation of study intervention may include the following circumstances:

1. The study intervention has an approved label that indicates it can be used safely in pregnant females

OR

1. The participant has a high mortality disease and the investigator determines the participant is benefiting from study participation and there is no other alternative treatment for her.

If continuation of study intervention following pregnancy is justified, the protocol should include details regarding what must occur prior to allowing continuation for that participant:

<Start of suggested text>

Prior to continuation of study intervention following pregnancy, the following must occur:

- The intervention owner and the relevant IRB/IEC give written approval.
- The participant gives signed informed consent.
- The investigator agrees to monitor the outcome of the pregnancy and the status of the participant and her offspring.

<End of suggested text>

### Cardiovascular and Death Events

- Refer to the master protocol. Add additional intervention cohort specific information if applicable.

### Disease-related Events and/or Disease-related Outcomes Not Qualifying as AEs or SAEs

- Refer to the master protocol. Add additional intervention cohort specific information if applicable

### Adverse Events of Special Interest

Refer to the master protocol for AEs of special interest. Add additional intervention cohort specific information if applicable.

Consult the appropriate medically qualified team member if unsure if this section is applicable for a particular protocol.

The description should include the following:

- The definition of the event.
- Is it a measurable quantity? If yes, how will the measurement be done?
- If it is a clinical event, how will it be confirmed?

### Overdose, medication errors, misuses or abuse of medicinal product

Refer to the master protocol for overdose, errors, and misuses or abuse of the medicinal product. Add additional intervention cohort specific information if applicable.

## Pharmacokinetics

If PK assessments are applicable across all intervention cohorts, refer to the master protocol Add additional intervention cohort specific assessments if applicable.

- Insert text as appropriate for this study. If population PK will be included, provide appropriate text. If PK will not be part of the study, include a statement to this effect.
- Describe any study intervention concentrations to be measured and the sample collection times relative to dosing. Samples of plasma, urine, or other fluids may be taken for the purpose of measuring compliance, adjusting dose, or determining if a therapeutic window exists. This section of the protocol will be written in collaboration with the appropriate PK representatives and will contain information about sampling times, sample volume, sample handling procedures, assay methods, etc. Specific sample collection and processing including retention time instructions can be described in an appendix and cross-referenced.
- Indicate definitions for the PK parameters (eg, area under the curve [AUC], maximum observed concentration [C_max_], time to C_max_ [T_max_], half-life [T_½_], volume of distribution [V_d_], clearance [CL]) of interest and how they will be calculated. Consult with the PK representative for this information.
- Describe sampling time relative to ingestion of food, posture, and possible effects of concomitant medications/alcohol/caffeine/nicotine.
- Describe the biological sample(s) collected (blood, urine, or other such as breath, saliva, biopsies, etc), the handling of samples, and the assay method including references to published and/or internal assay validation documentation.
- Specify other factors that are important in assessing the PK of the study intervention (eg, soluble circulating receptors, renal or hepatic function) and the plan for measuring these factors.
- Do not reiterate the details given in the SoA or other sections of the protocol. Use cross references as needed.

<Start of suggested text>

- [PK parameters are not evaluated in this study].

<End of suggested text>

OR

<Start of suggested text>

- [Plasma/serum/whole blood/urine] samples of approximately [X] mL will be collected for measurement of [plasma/serum/whole blood/urine] concentrations of [study intervention/other] as specified in the SoA (Section 1.3) [specify timepoints only if not obvious from the SoA].
- A maximum of [X] samples may be collected at additional timepoints during the study if warranted and agreed upon between the investigator and the intervention owner. The timing of sampling may be altered during the course of the study based on newly available data (eg, to obtain data closer to the time of peak plasma concentrations) to ensure appropriate monitoring.
- Instructions for the collection and handling of biological samples will be provided by the intervention owner. The actual date and time (24-hour clock time) of each sample will be recorded.
- Samples will be used to evaluate the PK of [study intervention]. Each [plasma/serum/whole blood] sample will be divided into [X] aliquots (1 each for [PK, other analyses, and a backup]). Samples collected for analyses of [study intervention (plasma/serum/whole blood)] concentration may also be used to evaluate safety or efficacy aspects related to concerns arising during or after the study.
- Genetic analyses will not be performed on these [plasma/serum/whole blood] samples [unless consent for this was included in the informed consent]. Participant confidentiality will be maintained. At visits during which [plasma/serum/whole blood/etc] samples for the determination of [multiple aspects] of [study intervention] will be taken, one sample of sufficient volume can be used.

If there are blinded study intervention concentration results, consider adding the relevant suggested text.

- Intervention concentration information that [may/would] unblind the study will not be reported to investigative sites or blinded personnel [until the study has been unblinded].

<End of suggested text>

## Genetics

- Refer to the master protocol for genetics assessments Add additional intervention cohort specific assessments if applicable.

## Biomarkers

- Refer to the master protocol for biomarker assessments. Add additional intervention cohort specific assessments if applicable.

## Immunogenicity Assessments

If immunogenicity assessments are applicable across all intervention cohorts, refer to the master protocol Add additional intervention cohort specific assessments if applicable.

If immunogenicity assessments are included as an efficacy or safety objective, then cross reference Sections 8.1 or a subsection of Section 8.2 and mark this section as not applicable. For other assessments to be used for research purposes, use the suggested text.

<Start of suggested text>

Antibodies to [study intervention] will be evaluated in [plasma/serum] samples collected from all participants according to the SoA. Additionally, [plasma/serum] samples should also be collected at the final visit from participants who discontinued study intervention or were withdrawn from the study. These samples will be tested by the intervention owner or the intervention owner's designee.

[Plasma/Serum] samples will be screened for antibodies binding to [study intervention] and the titer of confirmed positive samples will be reported. Other analyses may be performed to verify the stability of antibodies to [study intervention] and/or further characterize the immunogenicity of [study intervention].

The detection and characterization of antibodies to [study intervention] will be performed using a validated assay method by or under the supervision of the intervention owner. [All samples collected for detection of antibodies to study intervention] will also be evaluated for [study intervention] serum concentration to enable interpretation of the antibody data. Antibodies may be further characterized and/or evaluated for their ability to neutralize the activity of the study intervention(s). Samples may be stored for a maximum of [X] years (or according to local regulations) following the last participant’s last visit for the study at a facility selected by the intervention owner to enable further analysis of immune responses to [study intervention].

<End of suggested text>

## [Health Economics OR Medical Resource Utilization and Health Economics]

- Refer to the master protocol for health economics and medical resource utilization information. Add additional intervention cohort specific assessments if applicable.

# Statistical Considerations

<Start of suggested text>

A statistical analysis plan (SAP) that is applicable to [study intervention] is available in Appendix ## to this ISA. Statistical analysis for each intervention cohort [#] will be performed by the intervention owner or under the authority of the intervention owner. A general description covering the aspects of the statistical analysis relevant to this ISA is given below. Any analyses that are consistent across the platform study will cross reference back to the master protocol for this platform study. For [study intervention], the SAP will be finalized before the first unblinding of efficacy data (as required by ICH E9).

<End of suggested text>

## Statistical Hypotheses

- In general, the primary estimand should be the same for all intervention cohorts across the platform study.
- Any deviations from or intervention-specific modifications in the statistical hypotheses linked with the primary endpoint will be described here. If there are no difference from the master protocol, refer to Section 9.1 of the master protocol
- Clearly articulate the study hypotheses which will be the subject of statistical testing. In case no hypotheses are planned to be tested state so.
- Describe the multiplicity control strategy, if any.

### Multiplicity Adjustment

<Start of example text>

Example 1 (If key secondary endpoints are different than other interventions in the platform study):

The statistical comparisons for the primary efficacy endpoint (described in Section 9.1 of the master protocol for the platform study) and the key secondary endpoints will be carried out in the hierarchical order as indicated in Section 9.1. This means that statistically significant results for the comparison in the higher rank (primary, then ranked secondary variables) are required to initiate the testing of the next comparison in the lower rank. Since a step-down procedure is used, each comparison will be tested at a significance level of 0.05 and an overall alpha level of 0.05 will be preserved.

<End of example text>

## Analysis Sets

- If the analysis sets are defined the same as in the master protocol, refer to Section 9.2 of the master protocol.
- Any deviations from or intervention-specific analysis set definitions in Section 9.2 of the master protocol should be described here.

The definition for each participant analysis set should be defined at the participant level. Optionally, to implement the estimand framework, defined analysis data sets may be included here in a separate table that specify the set of data used in the analysis for a given estimand (or family of estimands that differ only in endpoint) based on how key intercurrent events will be handled for that estimand.

<Start of example text>

*Refer to the master protocol for analysis sets considerations.*

<End of example text>

## Statistical Analyses

<Start of suggested text>

The statistical analysis plan for [study intervention] will be finalized prior to [unblinding/FPFV/DBL] and it will include a more technical and detailed description of the statistical analyses that will apply to [study intervention] described in this section. This section is a summary of the planned statistical analyses of the most important endpoints including primary and key secondary endpoints specific to [study intervention].

<End of suggested text>

### General Considerations

- Refer to the master protocol for general consideration for the platform study. Add additional intervention cohort specific considerations if applicable.
- Specify decision criteria, such as nominal significance levels, one- or two-sided tests, and confidence interval probabilities.
- Common definitions of baseline
- Statistical methods
- Site pooling
- Handling of missing baseline values, if this is planned to be handled in the same way across analyses
- Definition of study periods if needed

<Start of suggested text>

- The statistical analysis will be performed in alignment with the following standards and regulations: [fill in as appropriate, for example ICH-E9 standards]. All available data will be used in the analysis [otherwise, specify here the exceptions]. The following paragraphs describe the statistical analysis for those endpoints that different from the endpoints described in Section 9.3.2 through 9.3.5 of the master protocol. the evaluation of the primary and secondary endpoints and other analysis that will be performed at the end of the study. Interim analysis specific to [study intervention] are described in Section 9.4.
- <End of suggested text>

### Primary Endpoint(s)

- State how the primary endpoint(s) will be defined/calculated/derived and used to address the primary objective.
- Refer to estimand(s) in Section 3 and ICH E9 (R1) if applicable and in case of more than one estimand, describe the analysis of primary endpoint for all estimands. If no estimands are defined, please describe how important anticipated protocol deviations will be handled
- Describe the main analytical approach including description of assumptions including assumptions on the missing data mechanism. Describe how missing data will be handled and describe (if applicable) factors, covariates, stratification factors etc. to be included in the analysis model
- Describe the planned sensitivity analyses and how the sensitivity analyses will target the assumptions behind the main analytical approach
- Describe any supplementary analyses. If estimands that are not mentioned in section 3 are planned to be addressed, they should be defined in this section. Consider if this can be moved to SAP.

*<Start of suggested text>*

See Section 9.3.2 of the master protocol.

<End of suggested text>

### Secondary Endpoint(s)

Key secondary endpoints eg, for which a label claim is pursued, are part of the confirmatory hypotheses for which the type 1 error is controlled and should be described to the same level of detail as the section on primary endpoint(s), but if the same methodology/analytical approach is taken for these endpoints it will be sufficient to make a cross reference to Section 9.3.2

Other (supportive) secondary endpoints need not be described with the same level of detail as the key secondary endpoints.

- If the secondary endpoints match the secondary endpoints described in the master protocol the following text is recommended

*<Start of suggested text>*

See Section 9.3.3 of the master protocol.

<End of suggested text>

- If the first or any secondary endpoint deviates from what is described in the master protocol, the following text is recommended.

Start of suggested text>

- The [first] secondary endpoint for [study intervention] is [cite the first secondary endpoint with its formal definition in terms of type of variables (eg, presence/absence outcome or survival outcome or continuous endpoints)]. The evaluation of this endpoint will be done by [summarize here the statistical method used, specifying the variables and the eventual considered covariates, the eventual needed assumptions, whether the analysis will be done also on specific subsets, how missing data will be handled].
- [Add a similar paragraph for any additional secondary endpoint that differs from what is presented in the master protocol].
- [If applicable, discuss how the issue of multiple testing will be handled, as applicable if it differs from what is described in the master protocol.]

*<End of suggested text>*

### Tertiary/Exploratory Endpoint(s)

A description can be omitted from the protocol section and a reference made to the SAP. No sensitivity or supplementary analyses need to be specified for tertiary/exploratory endpoints.

Add any additional intervention cohort specific tertiary/exploratory here. If there are no additional endpoints, refer to Section 9.3.4 of the master protocol.

### [Other/safety] Analysis

Add any additional intervention cohort specific other/safety analyses here. If there are no additional analyses, refer to Section 9.3.5 of the master protocol.

All safety analyses will be made on the Safety Population.

Describe how the safety data not defined as primary or secondary endpoints will be analyzed.

<Start of suggested text>

All safety analyses across [study intervention] will be based on the safety analysis dataset including all randomized participants who are exposed to study intervention(s). Participants will be analyzed according to the intervention(s) they actually receive.

*<End of suggested text>*

### Other Analysis

Add any additional intervention cohort specific other analyses here. If there are no additional analyses, refer to Section 9.3.6 of the master protocol.

Other analyses may include analyses of assessments, which are not defined as endpoints, that need to be prespecified and not necessarily be reported in the clinical study report such as, but not limited to, immunogenicity, biomarkers, population pharmacokinetics, health care utilization endpoints and health technology assessment related endpoints. State if these will be reported in a separate document.

Define any subgroups and the analysis of these.

## Interim Analysis

Add any additional intervention cohort specific interim analyses here.

If an interim analysis is planned, describe if any type of assessment committee will be established to evaluate the interim analyses (the safety data, and/or the critical effectiveness endpoints) in accordance with ICH E9. Also describe the role of the committee (eg, to recommend to the intervention owner whether to continue, modify, or stop a study). Full details of the committee including any charters should be included in Appendix 10.1.5 Committees Structure of the master protocol.

The following information belongs in this section:

- Reason for conducting interim analyses and their impact on the conduct of the study
- Variables to be included in the interim analyses
- The timing of analyses (eg, number of participants entered, number of participants completing certain number of visits, number of events, calendar time)
- Any actions resulting from an interim analysis such as sample size re-estimation, stopping rules or any adjustments to nominal significance level for final analyses
- Unblinded or blinded information
- Adaptive) making criteria and stopping rules (eg, for efficacy, binding/unbinding futility rules) and impact on operating characteristics. Discuss if stopping rules have been addressed in the sample size calculation), if unique to the intervention described in this ISA.
- If adaptations are envisaged: type of adaptive design with details of the pre-planned study adaptations and the statistical information informing the adaptations (eg, will adaptations be based on primary endpoint and/or secondary endpoints)

<Start of suggested text>

[Insert summary of interim analysis]

The Statistical Analysis Plan for this ISA will describe the planned interim analyses in greater detail

<End of suggested text>

## Sample Size Determination

- The sample size for a specific ISA will depend also on operational aspects of the study design (eg, time of entry of ISA). Simulations will help to evaluate different scenarios. In this case, a high-level summary of simulation results may be added to the ISA, referring for detailed results to an appendix. Alternatively, simplified calculations may allow one to approximate the required sample size.
- Potential stratification factors of importance, defining subpopulations of interest might need to be included. This is of relevance, if powering of subgroups is considered.
- Corrections for re-randomization of participants might need to be added, if not included into simulations or ballpark sample size calculation.
- Simulations involved in determining sample size will be defined in <SAP/DMC Charter/Standalone Appendix>
- When applicable, ensure this section clearly explains how screening failures and nonevaluable participants are defined, if different than what has been defined in the master protocol.
- In the Bayesian framework of most platform studies, any initial estimates are likely to be adapted over time. Therefore, please add the word ‘approximately’ in the text when stating the target sample size. This ensures that the protocol covers the potential to slightly over- or under-enroll.
- Include power calculations and level of significance to be used as appropriate and any posterior probabilities that are being used to demonstrate effectiveness of the intervention being evaluated, if applicable to the intervention being studied.

<Start of suggested text>

Endpoint to be used/Decision rule:

Decision making for an ISA in this platform study will be based on (pick one of the below)

- Frequentist testing at one-sided significance level alpha of [X]

- "Bayesian inference", targeting the posterior probability of no effects below alpha

- The location of 1-sided 1-alpha-confidence/credibility intervals relative to targeted effects

- Other

On the endpoint "x", as described in the section [X] of the master protocol.

Approximate sample size text (Based on worst case scenario):

Assuming a minimum clinically relevant effect of [X] and a randomization ratio of [X] [and an inflation of the control group size per ISA by [X] due to the use of concurrent control data], up to [X] participants will need to be evaluable to assert a (pick one of the below)

- probability of success of at least [X] for the ISA

- probability of inconclusive results of at most [X] for the ISA.

A total sample size for the platform study of [X] evaluable participants will allow evaluation of [X] ISAs under these assumed common effect assumptions.

Simulation guided sample size text

The required sample size of this ISA has been estimated based on clinical study simulations. Given the simulation results, at most [X] participants are required to be randomized for the evaluation of [intervention X] ISAs. Sample size and operation characteristics of the platform study will depend on the actual entry times of ISAs, enrollment into the ISAs and on the treatment effects. The simulation report summarizes operating characteristics of the study design under various scenarios.

If subpopulations of special interest exist / Enrichment

The following stratification factors will be considered in the randomization:

To allow for testing of subgroup effects in stratum [X], at least [X]% of the enrolled participants per ISA shall be enrolled from stratum [X].

# Supporting Documentation and Operational Considerations

Information that is too lengthy and could detract from the reader’s comprehension if included in the body of the protocol should be included in an appendix.

The order of the sections is determined by the order in which they are first referenced in the protocol text.

Modify, delete, or add sections as needed.

## Appendix 1: Regulatory, Ethical, and Study Oversight Considerations

<Start of common text>

Refer to the master protocol for Appendix 1, Regulatory, Ethical, and Study Oversight Considerations.

<End of common text>

## Appendix 2: Clinical Laboratory Tests

- Refer to the master protocol for Appendix 2, Clinical Laboratory Tests. Add additional intervention cohort specific assessments if applicable.

<Start of common text>

Refer to the master protocol for Appendix 2, Clinical Laboratory Tests.

<End of common text>

## Appendix 3: AEs and SAEs: Definitions and Procedures for Recording, Evaluating, Follow-up, and Reporting

<Start of common text>

Refer to the master protocol for Appendix 3, AEs and SAEs: Definitions and Procedures for Recording, Evaluating, Follow-up, and Reporting.

<End of common text>

## Appendix 4: Contraceptive and Barrier Guidance

Content for this appendix may be in the master protocol or the ISA as applicable. If this content is in the master protocol, add the statement under common text below.

Delete appendix if not required.

Insert content for this appendix from the participant libraries as appropriate based upon the decision trees in Section 5.1.

<Start of common text>

Refer to the master protocol for Appendix 4 Contraception and Barrier Guidance.

<End of common text>

### Definitions

See participant libraries for common text to include here.

### Contraception Guidance

See participant libraries for common text to include here.

## Appendix 5: Genetics

Delete appendix if not required.

<Start of common text>

Refer to the master protocol for Appendix 5, Genetics.

<End of common text>

## Appendix 6: Liver Safety: Suggested Actions and Follow-up Assessments [and Study Intervention Restart/Rechallenge Guidelines]

Delete appendix if not required.

<Start of common text>

Refer to the master protocol for Appendix 6, Liver Safety: Suggested Actions and Follow-up Assessments.

<End of common text>

## Appendix 7: Country-specific Requirements

Content for this appendix may be in the master protocol or the ISA as applicable. If this content is in the master protocol, add the statement under common text below.

<Start of common text>

Refer to the master protocol for Appendix 7: Country-specific Requirements.

<End of common text>

Delete appendix if not required.

Do not use this appendix to create extensive lists of country-specific differences. Protocol requirements and specifications outlined in the body of the protocol should be authored using flexible language to accommodate local variation where permissible and within the parameters of the study design; this appendix should be used for requirements that cannot be addressed by flexible language.

Discuss with local regulatory groups whether country-specific requirements need to be included in the appendix. The country-specific appendix may include a list (by country) of country-specific requirements in order that any requirements for a given country can be seen in one location.

Country-specific requirements listed in the appendix should also be clearly cross-referenced within the body of the document, within the sections they refer to, but details should not be included.

Countries where contraception requirements may differ: Australia, Japan

Korea: Local intervention owner should be identified in addition to company intervention owner on protocol agreement page.

## Appendix 8: Abbreviations [and Definitions]

This appendix may be in the master protocol and each ISA.

Delete appendix if not required.

- Generate a list while drafting the protocol to reflect the abbreviations used in the protocol.
- Only include those that are used more than once in the document. Once a term is abbreviated, it should be abbreviated in the rest of the document.
- Abbreviations are defined where first used in the document. If suggested text contains an abbreviation, the author can choose to retain the abbreviation or substitute the whole word(s).

## Appendix 9: ISA Protocol Amendment History

Note that the master protocol and the ISA protocol can be amended separately. Therefore, the amendment numbers for the companion protocols may not align. Further, the amendment history for the ISA protocol will be in the ISA protocol and the amendment history for the master protocol will be in the master protocol.

Delete appendix if not required.

Example text is included in this appendix for the Protocol Amendment History located here and the Protocol Amendment Summary of Changes Table located before the table of contents.

<Start of common text>

The Protocol Amendment Summary of Changes Table for the current amendment is located directly before the table of contents (TOC).

See the instructions in the Protocol Amendment Summary of Changes Table located before the table of contents. Move all Protocol Amendment Summary of Changes Tables for previous amendments to this appendix.

Amendment [amendment number]: ([date])

This amendment is considered to be [substantial/nonsubstantial] based on the criteria set forth in Article 10(a) of Directive 2001/20/EC of the European Parliament and the Council of the European Union.

**Overall Rationale for the Amendment**

[Rationale]

| Section # and Name | Description of Change | Brief Rationale |
| --- | --- | --- |
|  |  |  |
|  |  |  |
|  |  |  |

<End of common text>

<Start of example text>

**Amendment 3: 30 March 2016**

This amendment is considered to be substantial based on the criteria set forth in Article 10(a) of Directive 2001/20/EC of the European Parliament and the Council of the European Union.

**Overall Rationale for the Amendment**

Current literature supports use of this class of interventions in a higher age range for this participant population.

| **Section # and Name** | **Description of Change** | **Brief Rationale** |
| --- | --- | --- |
| 5.1. Inclusion Criteria | Removed maximum age range | To better reflect the age of the participant population |
| Throughout | Minor editorial and document formatting revisions | Minor, therefore have not been summarized |

Example of Numbering Global and Country-specific Protocol Amendments

| Type of Protocol Amendment | Numbering | Type of changes |
| --- | --- | --- |
| Country-specific | Amendment 3/FRA-2 | Same changes specific to France added to global Amendment 3 (no new changes for France) |
| Global | Amendment 3 | New changes for all |
| Country-specific | Amendment 2/FRA-2 | Additional changes specific to France added to global Amendment 2 |
| Country-specific | Amendment 2/FRA-1 | Same changes specific to France added to global Amendment 2 (no new changes for France) |
| Global | Amendment 2 | New changes for all |
| Country-specific | Amendment 1/FRA-1 | Same changes specific to France added to global Amendment 1 (no new changes for France) |
| Global | Amendment 1 | New changes for all |
| Country-specific | Amendment FRA-1 | Changes specific to France added to original protocol |

Example of Numbering a Site-specific Protocol Amendment

| Type of Protocol Amendment | Numbering | Type of changes |
| --- | --- | --- |
| Site-specific | Amendment 2/SS-1 <<Insert Site Number(s)>> | Same changes specific to site(s) added to global Amendment 2 (no new changes for site[s]) |
| Global | Amendment 2 | New changes for all |
| Site-specific | Amendment 1/SS-1 <<Insert Site Number(s)>> | Changes specific to site(s) added to global amendment |
| Global | Amendment 1 | New changes for all |

Example of Document History Table for Global and Country-specific Protocol Amendments

| DOCUMENT HISTORY | |
| --- | --- |
| Document | Date of Issue |
| Amendment 2/FRA-1 | 1-Feb-2016 |
| Amendment 2 | 1-Feb-2016 |
| Amendment 1/FRA-1 | 1-Jan-2015 |
| Amendment 1 | 01-Dec-2015 |
| Original Protocol | 01-Oct-2015 |

Example of Document History Table for Site-specific Amendments to a Global Amendment

| DOCUMENT HISTORY | |
| --- | --- |
| Document | Date of Issue |
| Amendment 2/SS-1 | 1-Feb-2016 |
| Amendment 2 | 1-Feb-2016 |
| Amendment 1/SS-1 | 1-Jan-2015 |
| Amendment 1 | 01-Dec-2015 |
| Original Protocol | 01-Oct-2015 |

<End of example text>

# References

- See therapeutic libraries for key references to include.
- References to both internal and external documents and publications should be listed in alphabetical order. Do not reference internal reports in preparation.
- In the reference list, use the style and format published by the International Committee of Medical Journal Editors [ICMJE, 2019]. Citations to external documents and publications should be indicated in the text by citing the author and year within parentheses. For example, the in-text citation for the reference included would be (Hatcher et al, 2007).
- References may be in the master protocol and the ISAs as applicable.

<Start of example text>

Hatcher RA, Trussell J, Nelson AL, Cates W Jr, Stewart F, Kowal D, eds. Contraceptive technology. 19th edition. New York: Ardent Media, 2007(a): 24. Table 3-2.

<End of example text>

<Start of suggested text>

EU‑PEARL D2.1. Report on Terminology, References and Scenarios for Platform Trials and Master Protocols.

IMI2 Call15-01: <https://ec.europa.eu/info/funding-tenders/opportunities/portal/screen/opportunities/topic-details/imi2-2018-15-01>

CTFG: Recommendation paper on the initiation and conduct of complex clinical trials. <https://www.hma.eu/fileadmin/dateien/Human_Medicines/01-About_HMA/Working_Groups/CTFG/2019_02_CTFG_Recommendation_paper_on_Complex_Clinical_Trials.pdf>

TransCelerate BIOPHARMA INC., Clinical Content & Reuse Solutions. <https://www.transceleratebiopharmainc.com/assets/clinical-content-reuse-solutions/>

Woodcock J and LaVange LM. Master Protocols to Study Multiple Therapies, Multiple Diseases, or Both. N Engl J Med. 2017;377:62-70.

<End of suggested text>
