# Supplementary material for: Developing generic templates to shape the future for conducting integrated research platform trials
Source: Trials. 2024 Mar 21;25:204. doi: 10.1186/s13063-024-08034-8 (PMC10956223; doi:10.1186/s13063-024-08034-8)
Supplement: Supplementary file 3 — Additional file 3. EU-PEARL Statistical Analysis Plan template. [file 13063_2024_8034_MOESM3_ESM.docx]

| **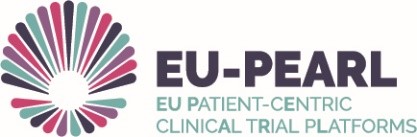** | **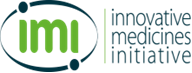** |
| --- | --- |

**Document title:**

**Statistical Analysis Plan Template**

| **Document history and plan** |
| --- |
| This updated SAP document (V3 25April2023) is based on the **EU-PEARL** **D2.3** ‘Provisional Generic Master Protocol Template and Appendix for IRPs’ (V2 30 April 2021). |
| The SAP of D2.3 was based on TransCelerate Statistical Analysis Plan SAP V3.0, copyright TransCelerate Biopharma Inc. 2018 – 2020. |
| Input received from the review committee was assessed for incorporation in the final deliverable D2.6 which is publicly available at the end of the project (April 2023). |

| **Authors** | Peter Mesenbrink (Novartis), Franz Koenig (MUW), Tobias Mielke (Janssen), Dan Evans (Pfizer), Ekkehard Glimm (Novartis), Paola Rancoita (USR), Yingwen Dong (Sanofi) |
| --- | --- |
| **Contact person EU-PEARL** | ECRIN; Peter Mesenbrink (Novartis) |
| **Document version** | Version 3 |
| **Date** | 25April2023 |

The EU-PEARL project has received funding from the Innovative Medicines Initiative 2 Joint Undertaking (JU) under grant agreement No 853966. The JU receives support from the European Union’s Horizon 2020 research and innovation programme and EFPIA and CHILDREN'S TUMOR FOUNDATION, GLOBAL ALLIANCE FOR TB DRUG DEVELOPMENT NON- PROFIT ORGANISATION, SPRINGWORKS THERAPEUTICS INC.

**Disclaimer**

These materials are provided AS IS WITHOUT WARRANTY OF ANY KIND, EITHER EXPRESSED OR IMPLIED, INCLUDING, BUT NOT LIMITED TO, THE IMPLIED WARRANTIES OF MERCHANTABILITY, FITNESS FOR A PARTICULAR PURPOSE, OR NONINFRINGEMENT.

EU-PEARL and its members do not accept any responsibility for any loss of any kind including loss of revenue, business, anticipated savings or profits, loss of goodwill or data, or for any indirect consequential loss whatsoever to any person using these materials or acting or refraining from action as a result of the information contained in these materials. Any party using these materials bears sole and complete responsibility for ensuring that the materials, whether modified or not, are suitable for the particular use and are accurate, current, commercially reasonable under the circumstances, and comply with all applicable laws and regulations.

Nothing in this template should be construed to represent or warrant that persons using this template have complied with all applicable laws and regulations. All individuals and organizations using this template bear responsibility for complying with the applicable laws and regulations for the relevant jurisdiction


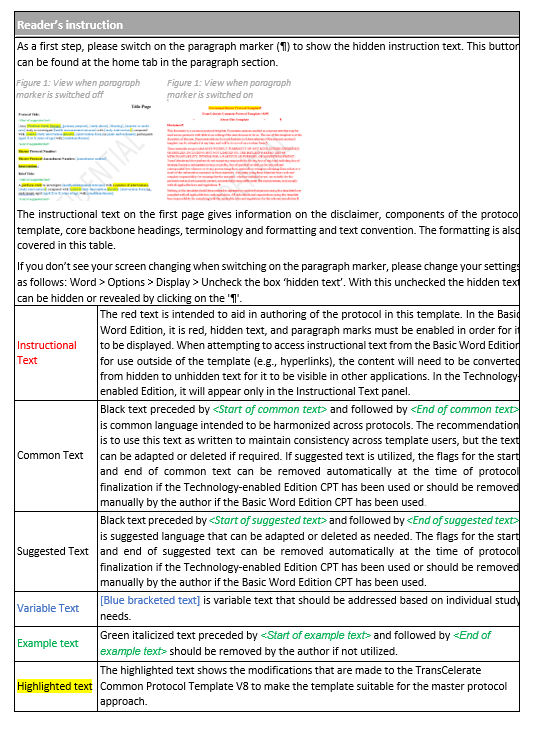


**Master Protocol Statistical Analysis Plan Template V1**

**About This Template**

**Disclaimer**

This document is a template for a statistical analysis plan (SAP) for platform studys that can be utilized in the core Master Protocol as Master SAP (mSAP) as well as in its intervention-specific appendices (ISAs) as ISA SAP. It contains sections marked as common text that may be used across SAPs with little to no editing if the user chooses to do so. The use of this template is at the discretion of the user.

These materials are provided AS IS WITHOUT WARRANTY OF ANY KIND, EITHER EXPRESSED OR IMPLIED, INCLUDING, BUT NOT LIMITED TO, THE IMPLIED WARRANTIES OF MERCHANTABILITY, FITNESS FOR A PARTICULAR PURPOSE, OR NONINFRINGEMENT. TransCelerate and its members do not accept any responsibility for any loss of any kind including loss of revenue, business, anticipated savings or profits, loss of goodwill or data, or for any indirect consequential loss whatsoever to any person using these materials or acting or refraining from action as a result of the information contained in these materials. Any party using these materials bears sole and complete responsibility for ensuring that the materials, whether modified or not, are suitable for the particular use and are accurate, current, commercially reasonable under the circumstances, and comply with all applicable laws and regulations.

Nothing in this template should be construed to represent or warrant that persons using this template have complied with all applicable laws and regulations. All individuals and organizations using this template bear responsibility for complying with the applicable laws and regulations for the relevant jurisdiction.

Please note that currently there are comments which may apply either mainly to the mSAP or the ISA SAP. When drafting this first version focus was given mainly on the context for a mSAP.

If the analyses are exactly the same for all ISAs and no deviations are expected, then it might be sufficient to have a single master SAP (mSAP). If there are (small) deviations, e.g., additional subgroup or sensitivity analyses for some of the ISA, then individualized SAPs for each ISA might be prepared as standalone documents in addition. However, in such case the mSAP defines the main analysis strategy for Master Protocol (including definition of estimands including analysis populations, analysis model (including definition of priors if applicable), multiple testing strategy,...). In such cases the mSAP serves as a template/blueprint of the standalone SAPs for each ISA of the platform trial concerned. If there are there separate Statistical Analysis Plan (SAP) for each new ISA, a stable draft SAP for the new ISA should be available at First Patient In (FPI) and a final one at least one week prior to unblinded DBL (and earlier, if the ISA will be used for registration.

**Components of the SAP Template**

- The **Core Backbone** and **Appendices** are designed to cover, at minimum, the needs for regulatory reporting and the clinical study report (CSR). Levels 1 and 2 headings are aligned with Section 9 Statistical Considerations in the common protocol template (CPT). Level 3 and lower headings can be deleted/added/modified if additional planned analyses need to be described.
  - The **Core Backbone** contains analysis plan information common to all phases, study populations, and therapeutic areas. The core backbone is streamlined and focused on the regulatory reporting recommendations and/or requirements.
  - **Appendices** provide additional information that can be accessed when needed, eg, abbreviations, data handling conventions, CSR items not critical for regulatory reporting.
    - This information may also be contained in separate documentation and referenced.

**Core Backbone Headings**

- Levels 1 and 2 headings should be consistent across analysis plans that use the TransCelerate common SAP template for reference purposes. The structure of this template aligns with the structure of the common protocol template, which is aligned with the United States National Institutes of Health (NIH) and Food and Drug Administration (FDA) Clinical Trials Protocol Template.
- Levels 1 and 2 headings should not be deleted. If they are not relevant to the study, not applicable should be inserted so that the numbering of subsequent sections is not changed.
- Level 3 and subsequent subheadings are suggested and can be deleted/added/modified as needed.

**Terminology**

- The terminology used in the SAP should match the protocol.
- The following terminology has been selected for use within TransCelerate common templates (CPT, SAP, and CSR) and is considered to be appropriate for all phases, study populations, and therapeutic areas.
  - *Participant* is used rather than subject, healthy volunteer, or patient.
  - *Study intervention* is used rather than study drug. Study intervention covers all types of investigational and noninvestigational products including medical devices and vaccines.
    - Study intervention is defined as investigational intervention(s), marketed product(s), placebo, or medical device(s) intended to be administered to a study participant per protocol.

**Formatting and Text Conventions**

- Common Text: Black font preceded by <Start of common text> and followed by <End of common text> is common language intended to be harmonized across SAPs. The recommendation is to use this text as written to maintain consistency across template users, but the text can be adapted or deleted, if required. The flags for the start and end of common text can be removed automatically at the time of SAP finalization if the Technology-enabled Edition SAP has been used or should be removed manually by the author if the Basic Word Edition SAP has been used.
- Suggested Text: Black text preceded by <Start of suggested text> and followed by <End of suggested text> is suggested language that can be adapted or deleted as needed.
- Variable Text: Blue bracketed text is variable text that should be addressed based on individual study needs.
- Example Text: Green italicized text preceded by <*Start of example text*> and followed by <*End of example text*> should be removed by the author if not utilized.
- Instructional Text: Intended to aid in the authoring of the SAP. In the basic Word edition, it is red, hidden text, and paragraph marks must be enabled in order for it to be displayed. In the technology-enabled edition, it will appear only in the instructional text panel.

**Disclosure**

- Users are reminded that many regulators have begun to require increased public disclosure of clinical trial documents. For recommendations related to using this template given the disclosure requirements, please refer to the best practices for disclosure in the implementation toolkit available on TransCelerate’s website.

Title Page

**Protocol Title:**

**Protocol Number:**

**Compound Number:**

**Short Title:**

[Acronym:]

**Sponsor Name:**

**Legal Registered Address:**

**Regulatory Agency Identifier Number(s):**

| **Registry** | **ID** |
| --- | --- |
| Enter Registry Name | Enter Registry ID |

Table of Contents

[Title Page 3](#_Toc52192283)

[Table of Contents 4](#_Toc52192284)

[Version History 5](#_Toc52192285)

[1. Introduction 6](#_Toc52192286)

[1.1. Objectives, Endpoints, and Estimands 7](#_Toc52192287)

[1.2. Study Design 8](#_Toc52192288)

[2. Statistical Hypotheses 11](#_Toc52192289)

[2.1. Multiplicity Adjustment 11](#_Toc52192290)

[3. Analysis Sets 13](#_Toc52192291)

[4. Statistical Analyses 16](#_Toc52192292)

[4.1. General Considerations 16](#_Toc52192293)

[4.2. Primary [Endpoint(s)/Estimand(s)] Analysis 17](#_Toc52192294)

[4.2.1. Definition of Endpoint(s) 17](#_Toc52192295)

[4.2.2. Main Analytical Approach 18](#_Toc52192296)

[4.2.3. Sensitivity [Analysis/Analyses] 19](#_Toc52192297)

[4.2.4. Supplementary Analyses 19](#_Toc52192298)

[4.3. Secondary [Endpoint(s)/Estimand(s)] Analysis 20](#_Toc52192299)

[4.3.1. [Key/Confirmatory] Secondary [Endpoint(s)/Estimand(s)] 20](#_Toc52192300)

[4.3.2. Supportive Secondary [Endpoint(s)/Estimand(s)] 22](#_Toc52192301)

[4.4. [Tertiary/Exploratory/Other] [Endpoint(s)/Estimand(s)] Analysis 22](#_Toc52192302)

[4.5. [Other] Safety Analyses 23](#_Toc52192303)

[4.5.1. Extent of Exposure 23](#_Toc52192304)

[4.5.2. Adverse Events 24](#_Toc52192305)

[4.5.3. Additional Safety Assessments (if applicable) 25](#_Toc52192306)

[4.6. Other Analyses 26](#_Toc52192307)

[4.6.1. Other Variables and/or Parameters 27](#_Toc52192308)

[4.6.2. Subgroup Analyses 27](#_Toc52192309)

[4.7. Interim Analysis 28](#_Toc52192310)

[4.8. Changes to Protocol-planned Analyses 30](#_Toc52192311)

[5. Sample Size Determination 31](#_Toc52192312)

[6. Supporting Documentation 33](#_Toc52192313)

[6.1. Appendix 1: List of Abbreviations 34](#_Toc52192314)

[7. References 36](#_Toc52192315)

Version History

This section should include high-level, concise documentation of any changes from previous versions of the statistical analysis plan (SAP) for the Master Protocol. Include a sentence at the beginning of this section that references the current version of the Master Protocol with the version date.

If this is version 1 of the Master Protocol SAP, include the information provided in the example table with only the first row completed.

For amendments to the Master Protocol SAP, describe the reason the Master Protocol SAP is amended such as: Master Protocol amendment, changes following blinded data review that impact all interventions in the Master Protocol, request from the data monitoring committee, etc. Details on the major changes and rationale for these changes for each Master Protocol SAP amendment are included in this section. A tabular format like the example table provided below may be used to present this information. Additional rows may be added to the table for the SAP amendment to describe the changes.

<Start of example text>

This statistical analysis plan (SAP) for Study XXXXXX is based on the Master Protocol dated ddMMMyyyy.

| **Master Protocol SAP Version** | **Date** | **Change** | **Rationale** |
| --- | --- | --- | --- |
| 1 |  | Not applicable | Original version |
| <add rows as necessary> |  |  |  |

<End of example text>

# Introduction

- Indicate the type of data included, eg, efficacy, safety, immunogenicity, pharmacokinetics [PK], pharmacodynamics [PD].
- Indicate if changes to protocol-planned analyses exist, and if so, reference the SAP section documenting these changes, eg, Section 4.8 Changes to Protocol-planned Analyses in this section. If there are no changes to the analyses described in the protocol, include a statement in the SAP to address this information.
- If non-key analysis specifications are not contained in this document (either main body or appendices), indicate what this Master Protocol SAP contains and that these non-key items are documented elsewhere with reference(s).
  - Non-key analysis specifications may include, but are not limited to, summaries/analyses of baseline characteristics and demographics, important protocol deviations, exploratory endpoints, derivations, and items not critical for regulatory decision-making.
    - These may be included as an appendix to the SAP. If this information is included in an appendix to the Master ProtocolSAP, then there is no need to reference a separate document.
  - Indicate table, figure, and listing specifications are contained in a separate document.
- Only those principles that apply to all interventions in the platform study should be described in this SAP. Any analysis details which are specific to a specific intervention-specific appendix (ISA) should be described in the analysis plan that supports the relevant ISA.

<Start of example text>

*XXX is a project to develop an environment for studying XXX disease and then test multiple different interventions for the secondary prevention of XX. The XXX project is running across Europe with multiple partners from academia and the commercial sector.*

*The project is designed as a perpetual platform study. This implies that there is a single Master Protocol dictating the conduct of the trial. The specific protocol for each intervention or combination of interventions that enters the platform study is summarized by an Intervention Specific Appendix (ISA) to the Master Protocol. Each ISA is inferentially separate. This implies that any conclusions and reports will be produced separately even though some placebo treated participants may contribute to several of the reports. Thus, by default, there will not be considerations to control for familywise type I error across ISAs.*

*The overarching statistical analysis considerations of the trial are dictated in a single Master Statistical Analysis Plan (mSAP) – this document. The specific SAP for each intervention specific trial is summarized by an appendix to the master SAP labelled as ISA-SAP. In case of conflicting information between the Master SAP and the ISA-SAP, the ISA SAP should be followed.*

*An overview of the terminology is given in the table below*

| *Generic name* | *Name in platform study* | *Name in sub-study* |
| --- | --- | --- |
| *Protocol* | *Master Protocol* | *Intervention-specific Appendix (ISA)* |
| *Statistical Analysis Plan (SAP)* | *Master SAP (mSAP)* | *Intervention-specific Appendix Statistical Analysis Plan (ISA-SAP)* |
| *Subject* | *Participant* | *Participant* |
| *Population* |  |  |
| *Interim analysis* |  |  |

[Changes to the Master Protocol-planned analyses are described in Section 4.8.]

OR

[There are no changes to the analyses described in the Master Protocol.]

<END of example text>

## Objectives, Endpoints, and Estimands

Objectives, Estimands, and Endpoint information should be copied directly from Section 3 of the Master Protocol. Additional estimands may be added to this section.

**Primary estimand/coprimary estimands**

<Start of example text>

The primary clinical question of interest is: What is the treatment difference in [health measurement/outcome] after [duration] of treatment in participants with [condition/disease] regardless of intervention discontinuation for any reason and regardless of initiation of rescue intervention or change in background intervention (dose and product)?

The estimand is described by the following attributes:

Population: participants with [condition/disease]. Further details can be found in Section 5 of the Master Protocol.

Endpoint: change from baseline to [timepoint] in [health measurement/outcome]

Treatment condition: the randomized treatment with or without [rescue medication or change in background medication] (treatment policy strategy). Further details on study interventions and concomitant, including rescue, interventions can be found in Section 6 of the Master Protocol.

The two intercurrent events “treatment discontinuation for any reason” and “initiation of rescue treatment or change in background treatment (dose and product)” are both addressed by the treatment condition of interest attribute. There are no remaining intercurrent events.

Population-level summary: difference in mean changes between treatment conditions

Rationale for estimand: [rationale].

Given the lack of approved therapies any changes to treatment are believed to be in the interest of the participant it is unknown if any of the intercurrent events will impact the disease trajectory this justifies the treatment policy strategy for this estimand.

**Secondary estimand(s)**

The clinical question of interest is for the secondary objective regarding [target of intervention]: What is the treatment difference in the percentage of participants achieving [health measurement/outcome] after [duration] of treatment in participants with [condition/disease] regardless of treatment discontinuation for any reason and regardless of initiation of any interventions affecting [outcome], eg, [medication and/or surgery]?

The estimand is described by the following attributes:

Population: participants with [condition/disease]. Further details can be found in Section 5 of the Master Protocol.

Endpoint: achievement of [health measurement/outcome] at [timepoint]

Treatment condition: the randomized treatment with or without any other [interventions] (treatment policy strategy). Further details on study interventions and concomitant, including rescue, interventions can be found in Section 6 of the Master Protocol.

*<End of example text>*

## Study Design

Include a brief description to clarify the schema details or add more information not included in the schema. This information should be enhanced from the Overall Design section of the MPT (Section 4.1).

- Recommend not duplicating information already in schema.
- Use bullets rather than lengthy text, if possible.
- Items to consider:
  - Study phase and study population, e.g., patient, healthy volunteer.
  - Design type, e.g., parallel, crossover, factorial, single group, SMART (sequential multiple assignment randomized study), and other design elements as required, e.g., dose-escalation, multicenter, adaptation.
  - Control method, e.g., placebo, active comparator, low dose, historical, or none (i.e., uncontrolled).
  - Blind level, e.g., open-label, single-blind, double-blind, double-blind (sponsor unblinded), matching placebos, double-dummy.
  - Method of assignment to treatment, e.g., randomization scheme including randomization ratio, stratification
  - Total duration of study participation for each participant with sequence and definition of study periods, e.g., screening, run-in, fixed dose/titration/maintenance/withdrawal, follow-up/washout periods.
  - Description of any provisions for extending the study or entry to rollover studies.
  - Rules/procedures for dose changes/adjustments including flexible dosing; dose reductions, interruptions, tapering, or rescue; temporary/permanent discontinuation; and any circumstances for resuming study intervention, as applicable.
  - Planned interim analyses.
  - Describe when the primary analysis will be performed, e.g., at database release after last participant last visit, at 100% primary endpoint data availability. Also describe final analysis timing if there will be subsequent reporting events, e.g., after long-term follow-up.
- <Start of suggested text>
- This is a [Phase 2a, Phase 2b, Phase 2a/b, Phase 3, Phase 2/3] platform study to investigate the [purpose (eg, efficacy and safety)] of multiple study interventions simultaneously or sequentially in cohorts of [provide high-level description of the study population].
- The Master Protocol describes the framework for the general study population and the common study elements of the platform study. The accompanying ISA(s) will present the intervention-specific information, including intervention-specific objectives, the justification for the study intervention dose(s), the number of participants to be assigned to an intervention cohort, any additional inclusion and exclusion criteria, and any additional study elements for the study intervention(s) as applicable.
- The study will be conducted in several phases: [provide high-level description of the study phases]. The duration of individual participation will be approximately [specify number of weeks or months].
- [Randomization across ISAs will be performed if at site level there is overlapping recruitment for at least 2 ISAs, the ISAs are focused on recruiting the same population (ie, no conflicting in/exclusion criteria), and the interventions have a similar benefit-risk ratio.]
- [Participants who do not meet all screening criteria for a particular ISA and agree to participate in another ISA may need to repeat some of the screening activities for the Master Protocol if they are outside the screening window as detailed in the applicable ISA.]
- [In general, participants who have enrolled in a given ISA will have the option to re-enroll in a different or subsequent ISA (if available) only after they have completed participation to the first ISA, and if they meet the new intervention-specific eligibility criteria.]
- Throughout the study [efficacy, safety, pharmacokinetics, pharmacodynamics, pharmacogenomics, biomarkers, immunogenicity, health economics] will be assessed at the timepoints indicated in the applicable ISA Schedule of Activities.
- A database lock is planned to occur after the completion of each intervention cohort.
- [Additional database locks may be added for an individual intervention cohort if appropriate and will be specified in the ISA].
- [A Data Monitoring Committee will be commissioned to review safety data periodically.]
- [Within each intervention cohort, interim analyses maybe performed, if applicable, at pre-specified time points. An Interim Analysis/Adaptations Committee will review the interim efficacy analysis results.]
- [An intervention arm within a given ISA or the complete ISA may be terminated if it is determined to be efficacious or futile based on interim analysis results or due to safety concerns].

<End of suggested text>

# Statistical Hypotheses

The information should be derived from the protocol (Section 9.1). Additional details may be added.

## Multiplicity Adjustment

- Please refer to both the Master Protocol and Master Protocol SAP (mSAP) and if applicable to ISAs whether a multiplicity adjustment is applied across ISAs.
- Please state if multiplicity shall be controlled for a certain family of hypotheses, for example for a certain set of hypotheses linked to a specific ISA or across the platform study.
- In a platform study, different sets of families of hypotheses might be defined, e.g., for each intervention-control comparison including a pre-defined set of primary and secondary endpoints. A rationale must be given. If the same adjustment methods are applied across all intervention cohorts in the Master Protocol, the overarching scheme is to be described in the Master Protocol itself and should be copied from there to the mSAP. In the ISA-SAP, additional details might be given how the adjustment is implement specifically for a specific intervention.
- If multiplicity adjustment strategies differ between intervention cohorts (due to different populations/treatments), this section should relate to the description given in Section 9.1.1 of the corresponding ISA. Please copy the text from the ISA and expand, if necessary, in the ISA specific SAP. In the master SAP refer to the ISA SAP.
- If in the platform study, multiplicity is being controlled only in specific ISAs, please refer in the master SAP to the ISAs and give more details in the ISA specific SAP.
- Clearly state the method for controlling overall type I error.
- Another complexity is if the FWER shall be controlled for a certain family of hypotheses in an ISA and adaptive interim analyses are conducted as well. Then special methods will be applied to address both multiple hypotheses, repeated significance testing and platform study adaptations. E.g., use of adaptive combination tests for both primary and secondary endpoints. Then the combination function (e.g., inverse normal with pre-specified weights) and adjusted significance levels (e.g., spending function with O´Brien & Fleming type of boundaries) will be pre-specified for each (intersection) hypotheses.
- If Bayesian decision rules are used, multiplicity could be discussed in term of frequentist error rates.
- State not applicable if multiplicity control is not relevant for this SAP platform study /ISA.

*<Start Example text>*

For the mSAP the high level overview of methods applying to all (or most) ISAs should be copied directly from the Multiplicity Adjustment section of the master protocol (Section 9.1.1). Additional details may be added.

**Example 1:**

*The statistical comparisons for the primary efficacy endpoint and the key secondary endpoints will be carried out in the hierarchical order as indicated in Section 9.1 for each intervention cohort. This means that statistically significant results for the comparison in the higher rank (primary, then ranked secondary variables) are required to initiate the testing of the next comparison in the lower rank. Since a step-down procedure is used, each comparison will be tested at a significance level of 0.05 and an overall alpha level of 0.05 will be preserved.*

**Example 2:**

*The type I error will be controlled in the strong sense using a hierarchical (fixed sequence) testing procedure. This is based on priority ordering of the null hypotheses and testing them in this order using the 2-sided 95% confidence interval approach until an insignificant result appears. Consequently, the second null hypothesis will only be tested if the first null hypothesis has been rejected in favor of* [*intervention x*].

The steps in the hierarchical testing procedure are as follows:

*Step 1:* [health measurement or observation] *non-inferiority of* [intervention x] *versus* [intervention y]

*Step 2:* [health measurement or observation] *superiority of* [intervention x] *versus* [intervention y]

# Analysis Sets

- This information should be obtained from the Analysis Sets section of the protocol (Section 9.2). Additional analysis sets may be added (if there are additional sets, then this information should be summarized in the version history and in Section 4.8 Changes to Protocol-planned Analyses).
- The definition of each analysis set need to include a temporal definition of which data will be utilized (e.g. using only concurrent control within the same ISA or also non-concurrent data from the platform study). This has to be aligned with definition in the master protocol template section 9.3.1.
- If additional estimands are defined in the SAP (e.g., in supplementary analyses sections, tertiary/exploratory/other endpoint[s]/estimand[s], other safety analyses, or other analyses sections), specify which analysis sets are used to estimate them and if necessary, define new analysis sets.
- In studies with estimands explicitly defined (mandatory for confirmatory studies), the analysis sets can be derived from the estimand description. However, for transparency, it is useful to specify in detail which participants and which data points are to be included in each analysis set used to estimate each estimand defined in the protocol. Note, naming of the sets of selected participants and not only the full data set (participants and data points) is recommended for ease of programming of the ADaM data set.
- In studies without estimands explicitly defined, the analysis sets should at minimum be defined at the participant level, and the data points to be included can be specified, if deemed relevant.
- If necessary, reference any team decisions that impact participant exclusions, e.g., if sites are excluded because of Good Clinical Practice violations. These decisions can be documented in the meeting minutes, if they are made after SAP finalization.
- Definitions that apply to entire platform study should be described in the Master Protocol and the master SAP.
- If there are any definitions that are intervention-specific this should be described in the ISA-SAP and the text should be aligned with Section 9.2 of the relevant ISA.

Three examples are provided below for illustration purposes only. Other formats, other definitions, and different naming conventions can be used. The first example is for a study with no estimands explicitly defined and the second and the third examples address a study with estimands explicitly defined.

<Start of example text>

Example 1:

For the purposes of analysis, the following analysis sets are defined:

| **Participant Analysis Set** | **Description** |
| --- | --- |
| Full analysis set | - All randomized participants. Participants will be included in the analyses according to the planned intervention. |
| Safety analysis set | - All participants who are exposed to study intervention. Participants will be included in the analyses according to the intervention they actually received. |

The full analysis set is used to analyze endpoints related to the efficacy objectives, and the safety analysis set is used to analyze the endpoints and assessments related to safety.

Example 2:

The following analysis data sets are defined to estimate the estimands defined in the protocol and to address safety.

| **Analysis Data Sets** | **Description** |
| --- | --- |
| Analysis set 1 for the primary estimand and for the secondary estimand for the secondary objective 1 | - PAS1: All randomized participants. Participants will be included in the analyses according to the planned intervention - For participants who discontinue study intervention and/or receive rescue therapy, post-discontinuation or post-rescue observations will not be included. |
| Analysis set 2 for the additional estimand for the primary objective | - PAS1: All randomized participants. Participants will be included in the analyses according to the planned intervention - For participants who discontinue study intervention and/or receive rescue therapy, all post-discontinuation or post-rescue observations will be included in the analysis set. |
| Safety analysis set 1 to be used for safety assessments with a long lag-time | - PAS2: All participants who are exposed to study intervention. Participants will be included in the analyses according to the intervention they actually received. - All observed data will be included in the analysis set. |
| Safety analysis set 2 to be used for safety assessments with an acute onset | - PAS2: All participants who are exposed to study intervention. Participants will be included in the analyses according to the intervention they actually received. - All observed data until discontinuation of intervention will be included in the analysis set. |

PAS = participant analysis set

Example 3:

The following participant analysis sets are defined:

| **Participant Analysis Set** | **Description** |
| --- | --- |
| Full analysis set (FAS) | - All randomized participants. Participants will be included in the analyses according to the planned intervention. |
| Safety analysis set | - All participants who are exposed to study intervention. Participants will be included in the analyses according to the intervention they actually received. |

The following data points sets are defined:

| **Data Points Sets** | **Description** |
| --- | --- |
| DPS1 | - For participants who discontinue study intervention and/or receive rescue therapy, post-discontinuation or post-rescue observations will not be included. |
| DPS2 | - For participants who discontinue study intervention and/or receive rescue therapy, all post-discontinuation or post-rescue observations will be included in the analysis set. |
| DPS3 | - All observed data will be included in the analysis set. |
| DPS4 | - All observed data until discontinuation of intervention will be included in the analysis set. |

FAS and DPS1 are used to estimate the primary estimand and the secondary estimand for secondary objective 1.

FAS and DPS2 are used to estimate the additional estimand for the primary objective.

Safety analysis set and DPS3 are used to present safety data with a long lag-time.

Safety analysis set and DPS4 are used to present safety data with an acute onset.

<End of example text>

# Statistical Analyses

## General Considerations

This information should be taken from the master protocol (Section 9.3.1). Additional general considerations may be added.

This section should describe general methods and definitions that do not need to be repeated in the subsequent sections. For example, a general statement that all treatment group comparisons for all categorical analyses will be tested using Fisher’s exact test does not need to be repeated for each categorical analysis described in later subsections. The same would be true for analysis of variance information and the model used. If different definitions are required for specific analyses, these should be stated in the relevant section. Suggested topics to be included in this section, if appropriate for the study, are provided below. If subsections are used, this section can include the section heading only, with no text required.

- Decision criteria, such as nominal significance levels, 1- or 2-sided tests, and confidence interval probabilities, if not already specified in Section 2
- Common definitions of baseline
- For randomized studies, describe stratification factors if applicable and if not specified in Section 1.2
- General methods, such as handling of wrong stratification, wrong intervention assignment, handling of values below lower limit of quantification, continuous variables will be summarized with min, max, mean, median, std, quantiles, etc.
- General choice of analysis sets for analyses
- Pooling strategies for countries/regions, sites, etc.
- Intervention grouping strategy, e.g., combining all active dose arms versus control
- Handling of missing baseline values, if this is planned to be handled in the same way across analyses
- Definition of study periods if needed
- For studies with estimands, describe how intercurrent events will be summarized (number by intervention group and timing)
- Definition of which study intervention contrasts will be provided
- Details of software used for the analyses
- Definition of how the control group will be defined across all of the interventions, any adaptations or sharing that will be broadly required should be outlined here. Intervention grouping strategy, e.g., combining all active dose arms versus control

<Start of suggested text>

- *The statistical analysis will be performed in alignment with the following standards and regulations:* [*fill in as appropriate, for example ICH-E9 standards, the GDPR*]. All *available data* [Platform study or ISA] *will be used in the analysis* [otherwise, specify here the exceptions, e.g., if in an ISA SAP only concurrent control data will be used]. *The following paragraphs describe the statistical analysis for the evaluation of the primary and secondary endpoints and other analysis that will be performed at the end of the study. Interim analysis are described in Section 4.7.*
- *Patient demographics and baseline characteristics will be summarized on the XXX set, overall and by randomized treatment group, by means of summary descriptive statistics.*
- *For qualitative variables (e.g. sex), absolute (n= ) and relative frequencies will be calculated per treatment group. Data will be visualized by bar plots. For quantitative data (e.g. age), the number of valid observations (n = ), mean, standard deviation, standard error, median, interquartile range, minimum and maximum will be calculated for each randomized group.*
- *Data will be visualized by [write here the more relevant graphical representation with respect to the aim of the descriptive statistics (e.g. histogram or boxplot) and eventually described whether reference/normative values will be plotted].*

<End of suggested text>

## Primary [Endpoint(s)/Estimand(s)] Analysis

This information should be copied directly from the protocol (Section 9.3.2). Additional details may be added.

### Definition of Endpoint(s)

- State how the primary endpoint(s) will be defined/calculated/derived and used to address the primary objective.
- Describe if the primary endpoint will be transformed, such as square-root and logarithm, before analysis. It is recommended to include the rationale/justification for transformation and the interpretation.

<Start of suggested text>

The primary endpoint is the Repeatable Battery for the Assessment of Neuropsychological Status (RBANS)™ Total Scale Index Score.

The RBANS™ Total Scale Index Score is comprised of the total mean score of the following 5 indexes themselves comprised of 12 subtests:

1. Immediate Memory – List Learning and Story Memory

2. Visuospatial/Constructional – Figure Copy and Line Orientation

3. Language – Picture naming and Semantic Fluency

4. Attention – Digit Span and Coding

5. Delayed Memory – List Recall, List Recognition, Story Memory, and Figure Recall

*The rate of decline during the treatment period of the intervention will be compared to placebo using the proportional disease progression model.*

*If the intervention owner requires an additional endpoint for decision making for an intervention, it will be specified in the Intervention Specific Appendix. This will not change the primary endpoint required in the Master Protocol and the Master SAP.*

<End of suggested text>

### Main Analytical Approach

- Refer to estimand(s) in Section 1.1 and ICH E9 (R1), if applicable. In case of more than one primary estimand due to different requirements across different regulatory agencies, describe the analysis of the primary endpoint for all primary estimands and indicate which estimands are required by which authorities if this is not clear from Section 1.1. If no estimands are defined, please describe how important anticipated protocol deviations will be handled.
- In sequential and/or adaptive designs, describe analysis methodology relevant to the sequential and/or adaptive design (e.g. corrected p-values, confidence limits, critical values, combination weights in combination tests).
- Refer to the analysis set to be used in the main analytical approach(es).
- Refer to the Statistical Hypotheses section (Section 2 of this template) regarding the hypothesis to be tested, if applicable.
- Describe the main analytical approach(es) (aligned to the primary estimand[s], if applicable). Describe how missing data will be handled. If imputation methods differ across different intercurrent events, describe how. In case of multiple imputation, state imputation model, number of datasets, and seed. Specify how datasets will be combined.
- Describe whether the analysis of the primary endpoints will require longitudinal analysis and, in case, which are the parameters that should be estimated (e.g. slope, time point differences; plateau of eventual non-linear trend) based on which model (e.g., specifying the variance-covariance matrix in a mixed model for repeated measurements)
- Describe (if applicable) prognostic factors, covariates, stratification factors, etc, to be included in the analysis model.
- Describe underlying assumption(s) of the main analytical approach(es) including assumptions on the missing data mechanism.
- In case of more than 2 study intervention groups, define which study intervention contrasts will be provided, if not fully described in the General Considerations section (Section 4.1 of this template).
- Describe how analysis results will be presented, such as estimated study intervention difference or a more general effect size, xx% confidence intervals, p-values, forest plots, etc.
- Recommended to describe how the endpoint(s) will be summarized descriptively including how missing data patterns will be presented.
- In case of transformation of the endpoint(s), describe whether the back transformation of the results will be also presented
- Describe diagnostics, if relevant.

### Sensitivity [Analysis/ Analyses]

- Describe the planned sensitivity analyses and how the sensitivity analyses will target the assumptions behind the main analytical approach(es). Pay special attention to assumptions regarding the missing data mechanism. A sensitivity analysis targets the same estimand / endpoint as the main analysis, but under different assumptions, e.g. regarding the behavior of patients in whom an observation of the primary endpoint is missing or with respect to covariate which have an impact on the primary endpoint.
- State the estimand which is targeted and specify in what way the sensitivity analysis assumptions will deviate from the main analysis assumptions, For example, you may state: “The main analysis followed participants who discontinued treatment and use the observed 12-week-response irrespective of whether a discontinuation of the randomized treatment occurred prior to this timepoint or not, This sensitivity analysis is a tipping point analysis which uses multiple imputation and a range of effect-modifying factors delta to impute the 12-week response of participants who discontinued randomized treatment prior to 12 weeks.
- If intercurrent events have been defined in the protocol, state how the sensitivity analysis will modify the handling of these intercurrent events relative to the main analysis. For example, you may modify the handling of missing data caused by an intercurrent event from an assumption of “missing at random” to assumption of “jump to reference” in the test treatment arm.
- If an alternative model is used to analyse the primary endpoint, describe how it is related to the main analysis. For example: “The primary analysis compared the response to treatment in the two arms by a stratified CMH test stratified by smoking status. This sensitivity analysis uses a logistic regression model including treatment, smoking status, disease severity score, gender and age group as covariates to analyze the response.”
- Describe how you plan to compare the results of the sensitivity analysis with results from the main analysis, e.g. under what outcomes you would label the results from the analysis as “robust” and when you would label them as “sensitive to assumptions”.

### Supplementary Analyses

- Describe any supplementary analyses. A supplementary analysis of the primary estimand / primary endpoint is an additional analysis of the primary endpoint that does not qualify as a sensitivity analysis. For example, it may be an analysis of a post-randomization subpopulations such as the per-protocol-set or patients who achieved a certain biomarker value during the course of the trial.Estimands to be estimated, but not defined in Section 3 of the protocol or in Section 1.1 of the SAP, should be defined in this section.
- If not defined in Section 3 of the SAP, describe which participants and data points are included in the analysis set(s) to be used to estimate each of the estimand(s) related to supplementary analyses.
- If the SAP is updated during study conduct and new intercurrent events that are not part of the predefined estimand(s) have emerged during study conduct, it should be addressed here. If the new intercurrent events result in a change of primary estimand, a protocol amendment should be considered.

## Secondary [Endpoint(s)/Estimand(s)] Analysis

This information should be taken from the master protocol (Section 9.3.3). Additional details may be added.

### [Key/Confirmatory] Secondary [Endpoint(s)/Estimand(s)]

Key/confirmatory secondary endpoint(s)/estimand(s) (eg, for which a label claim is pursued) are part of the confirmatory hypotheses where the type 1 error is controlled (via multiplicity adjustment). It is recommended to describe the analysis of such endpoints to the same level of detail as the primary endpoint(s)/estimand(s) being described in, Section 4.2. If the same methodology/analytical approach is taken for these endpoints, it will be sufficient to add a crossreference to Section 4.2 to avoid redundancy.

#### Definition of Endpoint(s)

- State how the secondary endpoint(s) will be defined/calculated/derived and used to address the primary objective. In particular it is suggested to specify whether a secondary endpoint is binary, categorical (with proportional categories or not) or continuous.
- Describe if the secondary endpoint will be transformed, such as square-root and logarithm, before analysis. It is recommended to include the rationale/justification for transformation and the interpretation.

#### Main Analytical Approach

- Refer to estimand(s) in Section 1.1 and ICH E9 (R1), if applicable. In case of more than one secondary estimands due to different requirements across different regulatory agencies, describe the analysis of the secondary endpoints for all secondary estimands and indicate which estimands are required by which authorities if this is not clear from Section 1.1. If no estimands are defined, please describe how important anticipated protocol deviations will be handled.
- In sequential and/or adaptive designs, describe analysis methodology relevant to the sequential and/or adaptive design (e.g. corrected p-values, confidence limits, critical values, combination weights in combination tests). This is mostly relevant for confirmatory secondary endpoints.
- Refer to the analysis set to be used in the main analytical approach(es).
- Refer to the Statistical Hypotheses section (Section 2 of this template) regarding the hypothesis to be tested, if applicable.
- Describe the main analytical approach(es) (aligned to the secondary estimand[s], if applicable). Describe how missing data will be handled. If imputation methods differ across different intercurrent events, describe how. In case of multiple imputation, state imputation model, number of datasets, and seed. Specify how datasets will be combined.
- Describe whether secondary endpoints will require longitudinal analysis and, in case, which are the parameters that should be estimated (e.g. slope, time point differences; plateau of eventual non-linear trend)
- Describe (if applicable) prognostic factors, covariates, stratification factors, etc, to be included in the analysis model.
- Describe any factors that will be used to evaluate the heterogeneity of the population for which the endpoint(s) are being evaluated and if any adjustments will be made.
- Describe underlying assumption(s) of the main analytical approach(es) including assumptions on intercurrent events/missing data
- In case of time-to-event data, specify the nature of censoring events (e.g. informative and non-informative censoring)
- In case of more than 2 study intervention groups, define which study intervention contrasts will be provided, if not fully described in the General Considerations section (Section 4.1 of this template).
- Recommended to describe how the endpoint(s) will be summarized descriptively including how missing data patterns will be presented. Describe how analysis results will be presented, such as estimated study intervention difference or more general effect size, xx% confidence intervals, p-values, forest plots, etc. To set hypothesis testing for evaluating clinical efficacy a “clinically relevant change” should be defined to be able to provide a clinical definition of effect size.
- Describe diagnostics, if relevant.
- Describe how data and/or results will be presented graphically, for example, dot plots (if small sample size), bar plots or box plot or error bars in univariate, or multivariate plots.
- In case of transformation of the endpoint(s), describe whether the back transformation of the results will be also presented.

#### Sensitivity [Analysis/Analyses]

- Describe the planned sensitivity analyses and how the sensitivity analyses will target the assumptions behind the main analytical approach(es). Pay special attention to assumptions regarding the missing data mechanism, if applicable, evaluating also possible bias in the analysis of longitudinal or time-to-event outcomes due to drop outs. Supplementary Analyses.

#### Supplementary Analyses

- Describe any supplementary analyses in particular when endpoints or observe data deviate from main statistical approaches above described.
- Estimands to be estimated, but not defined in Section 3 of the protocol or in Section 1.1 of the SAP, should be defined in this section.
- If not defined in Section 3 of the SAP, describe which participants and data points are included in the analysis set(s) to be used to estimate each of the estimand(s) related to supplementary analyses.
- If the SAP is updated during study conduct and new intercurrent events that are not part of the predefined estimand(s) have emerged during study conduct, it should be addressed here. If the new intercurrent events result in a change of secondary estimand a protocol amendment should be considered.

### Supportive Secondary [Endpoint(s)/Estimand(s)]

It is recommended to describe the analyses of other (supportive) secondary endpoints in sufficient detail (including details on handling of missing data), in order for an independent statistician to be able to redo the analyses. The following topics should be considered:

- State how the endpoints will be defined/calculated/derived.
- Describe if the endpoint will be transformed, such as square-root and logarithm, before analysis including rationale/justification and interpretation.
- Refer to (or define, if not in Section 1.1) relevant estimand(s).
- Specify the analysis set(s) to be used, if not clear from Section 3.
- Specify analysis methods including handling of missing data.
- Describe (if applicable) factors, covariates, stratification factors, etc to be included in the analysis model.
- Specify how results will be presented.
- Sensitivity and supplementary analyses are not in scope for these endpoints but can be added, if deemed necessary

## [Tertiary/Exploratory/Other] [Endpoint(s)/Estimand(s)] Analysis

If specified in the protocol, this information can be taken from the master protocol (Section 9.3.4). Additional details may be added.

If no description is provided in the protocol, then it is recommended to describe the analyses to the same level of detail as the supportive secondary endpoints. For example, no sensitivity or supplementary analyses need to be specified for tertiary/exploratory/other endpoints/estimands.

## [Other] Safety Analyses

The high-level overview of the overarching principles and methods applying to all (or most) ISAs should be taken from the protocol (Section 9.3.5). Additional details on the statistical analyses may be added.

Consider describing how standard and any special safety endpoints analyses, that have not already been described in Sections 4.2 Primary Endpoint(s) Analysis, 4.3 Secondary Endpoint(s) Analysis, or 4.4 Tertiary/Exploratory [Endpoint(s)/Estimand(s)] Analysis, will be performed.

Suggested topics, specific to safety analyses to be included in this section or subsections, are provided below. If potentially clinically significant criteria are used for adverse events (AEs), laboratory assessments, vital signs, or electrocardiograms (ECGs), then describe how these criteria are determined and how they relate to clinical relevance or include a reference to standards, appendix, or other documentation.

- Specify estimand(s), if applicable and not defined in Section 1.1.
- Specify analysis set(s) to be used if not clear from Section 3.
- Describe general methods for summarizing and analyzing safety data.
- Include special safety topics relevant to the given study and its rationale.
- Specify how to handle unscheduled visits and/or duplicate measurements.
- If running categorical analyses for participants with a potentially clinically significant change, describe criteria in the appendix or refer to referenced documentation or standards.
- For categorical analyses, describe in detail the participants in the denominator and numerator. Pay particular attention to baseline requirements for denominators. For instance, to assess who goes high, usually participants need to be low or normal at baseline. Specify whether baseline includes all baseline visits, just the last baseline visit, or some other criteria.
- Describe methods used to compute any derived data, eg, reporting absolute change or percent change from baseline, transformation of data, or use of categorical cut-points for safety scales

<Start of suggested text>

*All safety analyses across all interventions in the platform study will be based on the Safety analysis dataset including all randomized participants who are exposed to study intervention(s). Participants will be analyzed according to the intervention(s) they actually receive.*

[*In addition, similar analyses will be performed on the Expanded Safety analysis dataset including all randomized participants who are exposed to the investigational intervention and participants who are randomized and exposed to the control(s) across available intervention cohorts.*]

<End of suggested text>

### Extent of Exposure

- Describe the summaries that will be provided for extent of exposure to study intervention, which may include the number of participants exposed, the duration of exposure, the dose(s) to which they were exposed, and dose modifications.
- If applicable, also describe how to calculate the total daily dose, total years of exposure, etc.
- The wording should be adjusted to account for oral agents, infusions, injections, or other nonoral administrations.
- If applicable, duration of exposure to any treatment can be summarized with descriptive statistics.
- It might also be helpful to describe the number of participants exposed for specified periods of time, such as for 1 day or less, 2 days to 1 week, more than 1 week to 1 month, more than 1 month to 6 months.
- This information will be intervention-specific

*<Start of example text>*

*For each intervention that is evaluated as part of the platform study, exposure will be calculated based on total daily dose over treatment duration in months/years. Details of the summary of any exposure assessments required for a specific trial drug will be reported in the ISA.*

*<End of example text>*

### Adverse Events

Suggested topics to be included in this section or as subsections are provided below:

- The summaries that will be presented, such as overall, by drug relationship, by intensity/grade, by special interest, or by study period, and with reported terms being mapped to preferred terms according to dictionary.
- Methods to be used for analysis of AEs such as relative risk, exposure-adjusted incidence rate.
- Details on how treatment-emergent AEs are defined.
- Description of whether counts (proportions) of participants and/or counts (rates) of events will be summarized.
- Presentation of AEs from coding, such as summarizing by primary system organ class and preferred term.
- Presentation of fatal serious AEs (SAEs) and nonfatal SAEs.
- Presentation of AEs leading to discontinuation of treatment and/or withdrawal from the study.
- Presentation of other significant AEs, if applicable.

*<Start of example text>*

*The verbatim terms used in the CRF by investigators in the platform study to identify adverse events will be coded using the Medical Dictionary for Regulatory Activities* *(MedDRA)*. *Intervention-emergent adverse events are adverse events with onset during the* [*lead-in, intervention, follow-up*] *phase or that are a consequence of a pre-existing condition that has worsened since baseline. All reported adverse events will be included in the analysis. For each adverse event, the number* *(percentage) of participants who experience at least 1 occurrence of the given event will be summarized by intervention arm. AEs of special interest to any specific study intervention will be evaluated.*

*Summaries, listings, datasets, or participant narratives may be provided, as appropriate, for those participants who die, who discontinue intervention due to an adverse event, or who experience a severe or a serious adverse event.*

[*Parameters with predefined National Cancer Institute Common Terminology Criteria for Adverse Events (NCI-CTCAE) toxicity grades will be summarized. Change from baseline to the worst adverse event grade experienced by the participant during the study will be provided as shift tables.]*

*<End of example text>*

### Additional Safety Assessments (if applicable)

- Add other sections such as Laboratory Data, Vital Signs, and ECGs, if applicable to the study. Delete this section, if it is not applicable.

#### Clinical Laboratory Tests

*<Start of example text>*

*Laboratory data will be summarized by type of laboratory test. Reference ranges will be used in the summary of laboratory data. Descriptive statistics will be calculated for each laboratory test at baseline and for observed values and changes from baseline at each scheduled time point. Results will be presented in pre- versus post-intervention cross-tabulations (with classes for below, within, and above normal ranges). Frequency tabulations of the abnormalities will be made. A listing of participants with any laboratory results outside the reference ranges will be provided.*

[*The laboratory abnormalities will be determined according to the criteria specified in the DAIDS Toxicity Grading Scale3 and in accordance with the normal ranges of the clinical laboratory if no gradings are available.]*

*[Markedly abnormal laboratory results will be summarized and a listing of participants with any markedly abnormal laboratory results will be provided]*

Any analyses specific to an intervention in the platform study should be described in an appendix to this statistical analysis plan.

*<End of example text>*

#### Electrocardiogram

*<Start of example text>*

*Electrocardiogram data will be summarized by ECG parameter. Descriptive statistics will be calculated at baseline and for observed values and changes from baseline at each scheduled time point. Frequency tabulations of the abnormalities will be made.*

*The ECG variables that will be analyzed are heart rate, PR interval, QRS interval, QT interval, and corrected QT (QTc) interval using the following correction method:* [*QT corrected according to Bazett's formula (QTcB), QT corrected according to Fridericia's formula (QTcF)*.]

[*Descriptive statistics of QTc intervals and changes from baseline will be summarized at each scheduled time point. The number (percentage) of participants with QTc interval >450 milliseconds, >480 milliseconds, or >500 milliseconds will be summarized, as will the number (percentage) of participants with QTc interval increases from baseline >30 milliseconds or >60 milliseconds.]*

Any analyses specific to an intervention in the platform study should be described in an appendix to this statistical analysis plan.

*<End of example text>*

#### Vital Signs

*<Start of example text>*

*Descriptive statistics of* [*temperature, pulse/heart rate, respiratory rate, and blood pressure (systolic and diastolic)*] *values and changes from baseline will be summarized at each scheduled time point. The number (percentage) of participants with values beyond clinically important limits will be summarized.*

Any analyses specific to an intervention in the platform study should be described in an appendix to this statistical analysis plan.

*<End of example text>*

#### Physical Examination

*<Start of example text>*

*Physical examination results and findings will be summarized.*

Any analyses specific to an intervention in the platform study should be described in an appendix to this statistical analysis plan.

*<End of example text>*

#### Suicidal Ideation and Behavior Risk Monitoring

This subsection should only be retained in those master protocols where this assessment is actually being performed. If it is not being performed, the header can be removed.

*<Start of example text>*

[Columbia Suicide Severity Rating scale (C-SSRS) data from subjects with suicidal ideation or behavior at any time during the study will be summarized.]

Any analyses specific to an intervention in the platform study should be described in the ISA.

<*End of suggested text*>

## Other Analyses

This information will be taken from the Other Analyses section of the protocol (Section 9.3.6). Additional details may be added.

### Other Variables and/or Parameters

Other analyses may include analyses of assessments or derived parameters, which are not defined as endpoints but need to be prespecified in either the protocol or the SAP. Examples include but are not limited to: immunogenicity, biomarkers, PK/PD/population PK parameters, health care utilization variables, and health technology assessment-related variables.

Subsections may be used for different topics.

It is recommended that the variables used in the analyses should be clearly defined and the analyses should be described at the same level of detail as supportive secondary endpoints being described in, Section 4.3.2 of the SAP.

The definition and derivation may be specified in a table format.

Specify estimands not defined in Section 1.1 of the SAP, if applicable.

### Subgroup Analyses

Note: Often individual standard clinical studies (excluding large outcomes or safety studies) are not designed to allow for statistically meaningful subgroup analyses because of too small sample sizes. Also, subgroup analyses are not commonly included in the set of multiplicity-controlled analyses and are therefore subject to multiplicity issues.

It is recommended to consider addressing the following topics, if applicable:

- Define the endpoints subject to subgroup analysis – can be for either efficacy and safety, or both.
- Define subgroups (may include stratification factor, if relevant).
- Provide the purpose (consistency, hypothesis) of each subgroup analysis. The subgroup analyses should preferably be further substantiated, eg, biological plausibility of anticipated differential effect, regulatory/payer requirement.
- Specify any rules to define the minimum size of a subgroup in order to carry out the analysis.
- Specify analysis sets/estimand, as applicable.
- Specify the subgroup analysis methods, including how missing data are handled.
- Specify the level of significance for the test of the treatment-by-subgroup interaction.
- Assess consistency across regions and subpopulation(s) for multi-regional clinical studies, c.f. ICH E17.
- Describe how results will be presented. It is recommended to focus on estimates and confidence intervals rather than p-values. It is often useful to display the results in a forest plot.

<Start of example text>

Subgroup analyses of the primary endpoint and confirmatory secondary endpoints will be made to assess consistency of the intervention effect across the following subgroups:

- Age group: < 65 vs ≥ 65 years
- Sex: female vs male
- Race: white vs black vs other

If the number of participants is too small (less than [10%]) within a subgroup, then the subgroup categories may be redefined prior to unblinding the study.

The treatment effect and its associated X% confidence interval will be estimated for each subgroup. A forest plot summarizing the results will be provided.

<End of example text>

## Interim Analysis

Please take (and if applicable) expand text from Section 9.12 from the master protocol if interim analyses were planned for each intervention cohort of the platform study.

- From section x.xx of the corresponding ISAs if interim analyses are intervention-specific
- Text should be aligned with technical details provided in the applicable DMC charter given in Appendix 10.1.5 Committees Structure.

The following information belongs in this section:

- Reason for conducting interim analyses and their impact on the conduct of the study
- Timing of interim analysis relative to platform studies (e.g. will it be milestone-based or time frequency based as such that certain criteria are met (events, number of patients, follow-up time) this would trigger specific interventions to be included as part of the interim analysis
- Variables to be included in the interim analyses
- Unblinded or blinded information
- The timing of analyses (e.g., approximate number of participants entered, approximate number of participants completing certain number of visits, number of events, calendar time)
- (Adaptive) making criteria and stopping rules (e.g., for efficacy, binding/unbinding futility rules) and impact on operating characteristics. Discuss if stopping rules have been addressed in the sample size calculation), if applicable to all interventions
- Any actions resulting from an interim analysis such as sample size re-estimation, stopping rules or any adjustments to nominal significance level for final analyses
- If adaptations are envisaged: type of adaptive design with details of the pre-planned study adaptations and the statistical information informing the adaptations (e.g., will adaptations be based on primary endpoint and/or secondary endpoints)
- If response adaptive designs are used: give details on burn-in period and if and how long control group allocation is fixed, algorithm to modify allocation ratio and information to be used for updates, if this can be done generically at the platform study level. Otherwise, such text should be described in the ISA (and related SAPs)
- Discuss how interim analysis affects statistical testing and estimation across all interventions being evaluated in the platform study

This information will be copied directly from the protocol (Section 9.4). Additional details may be added.

If an interim analysis is planned, describe if any type of data monitoring committee will be established to evaluate the interim analyses (the safety data, and/or the critical efficacy endpoints) in accordance with ICH E9. Also describe the role of the committee (e.g., making recommendation to the sponsor whether to continue, modify, or stop a study).

Include the following information in this section:

- Reason for conducting interim analyses and the impact on the conduct of the study
- Endpoints to be included in the interim analyses
- Timing of the interim analyses (eg, number of participants enrolled, number of participants completing a certain number of visits, number of events, calendar time)
- Any actions resulting from an interim analysis, such as sample size re-estimation, or stopping rules
- Multiplicity considerations relating to the interim and final analyses
- Blinding/unblinding strategy

<Start of example text>

An interim analysis of the primary endpoint will be performed by the independent data monitoring committee (IDMC), consisting of [X] clinicians and 1 statistician who are independent experts not otherwise involved in the study, when approximately [X] primary events have occurred. The analysis method for the primary efficacy endpoint described in Section 4.2 Primary Endpoints/Estimands Analysis will be used for the interim analysis. Based on the group sequential design with the [O’Brian Fleming] alpha spending approach, a 2-sided alpha of [X] will be allocated to the interim analysis. In addition, if the conditional power for the final analysis (based on the original assumption for the remaining study) is [X] or lower, the study may be stopped for futility.

The interim analysis will be conducted such that the ongoing study integrity is maintained. Only the independent statistical support group, who is responsible for providing the interim analysis results to the IDMC will be unblinded to the individual treatment group assignments. Interim analysis results will not be shared with investigators, participants, or the study team who are involved in the conduct of the study before the final database lock.

<End of example text>

<Start of example text 2:>

*This POC platform study as such will not control for the Type I error. For each ISA, the sample size and the stopping criteria are set in consideration of the Type I error, which is evaluated by simulation.*

*The interim analyses will be conducted every three months. It will be the same time for all ISAs, but the analyses will be made separately. The actual analysis will follow the principles for the primary efficacy analysis described in Section XX.*

*For each evolution analysis conducted in which an intervention has reached a minimum exposure to research participants, an analysis of graduation (success) will be conducted. With the same constraints, an analysis of futility will be conducted. The default criterion is at least XXX subjects with xx months of follow up in an intervention cohort, but this can be specified within an ISA. The details of these analyses are:*

1. *If the posterior probability of event Y is greater than YY then the intervention meets graduation criteria and will be labelled a success. This analysis concludes a super superiority, meaning there is high probability of at least a 10% slowing in the rate of decline is met for the intervention*
2. *If the posterior probability of a event Y is less than ZZ then the futility criteria is met for that intervention. Thus, if there is high probability of event Y, the intervention will be declared futile.*

The sponsor will only be informed of the decision to continue or stop the trial and will not be unblinded to the results until the study has ended.

<End of example text>

## Changes to Protocol-planned Analyses

The purpose of this section is to document changes from the planned analyses specified in the protocol. The changes themselves should be incorporated into the relevant sections of this document.

This section may be included in the first version of the SAP, ie, not an amendment, if such changes were decided after the initial protocol was finalized. If this section is part of an SAP amendment, then there will be some overlap with the information presented in this section and the version history. Additional justifications may be provided in this section (e.g., for regulatory reasons). Changes made after database lock are out of scope for the SAP.

If there are changes to protocol-planned analyses that were not documented in the final protocol or latest protocol amendment at the time of SAP development, summarize these important changes with rationale, according to ICH E3, Section 9.8. Additional analyses that do not replace planned analyses, or clarifications are not considered changes. If there were no important changes to the protocol-planned analyses, write “Not applicable”.

# Sample Size Determination

This information should be copied directly from the Sample Size Determination section of the CPT (Section 9.5). Additional details may be added to further describe the sample size methods.

<Start of suggested text>

*The sample size of this platform study is driven by the total number of ISAs included into the study, the individual effect assumptions and the design specifics of the underlying ISAs.*

*A maximum of [x] participants will be [randomly assigned/enrolled] to each study intervention in the platform study.*

If data between ISAs to be shared, e.g., concurrent control

*Due to the sharing of concurrent data, operating characteristics of the platform study will depend on the time of inclusion of ISAs and the enrollment rate into the different ISAs.*

Clause adding flexibility on effect assumptions due to new information

The available information on the endpoints used for decision making is expected to change over the duration of the platform study and will be considered in the confirmation of the required sample size of newly added ISAs.

Endpoint to be used/Decision rule:

*Unless specified differently in the specific ISAs, decision making for an ISA in this platform study will be based on (pick one of the below)*

*- frequentist (superiority / non-inferiority) testing at one-sided significance level alpha of [X]*

*- "Bayesian inference", targeting the posterior probability of no effects below alpha*

*- the location of 1-sided 1-alpha-confidence/credibility intervals relative to targeted effects*

*- other*

*on the endpoint "x", as described in the section [X] of the Master Protocol.*

Approximate sample size text (Based on worst case scenario):

*Assuming a minimum clinically relevant effect of* [X] *and a randomization ratio of* [X] [*and an inflation of the control group size per ISA by [X] due to the use of concurrent control data*], *up to* [X] *subjects will need to be evaluable per ISA to assert a* (pick one of the below)

- *probability of success of at least* [X] for the ISA

- *probability of inconclusive results of at most* [X] *for the ISA*.

*A total sample size for the platform study of* [X] *evaluable participants will allow evaluation of* [X] *ISAs under these assumed common effect assumptions*.

*Based on the common effect assumptions and assuming the sample size of [X] per ISA and a total number of* [X] *ISAs, the probability to detect at least one intervention as effective is:* [X].

Simulation guided sample size text

*The required sample size of this platform study has been estimated based on clinical study simulations. Given the simulation results, at most* [X] *subjects are required to be randomized for the evaluation of* [X] *ISAs. A total sample size of* [X] *is expected to be required for the platform study. Sample size and operation characteristics of the platform study will depend on the actual entry times of ISAs, enrollment into the ISAs and on the treatment effects. The simulation report summarizes operating characteristics of the study design under various scenarios.*

No sample size text – defer to ISA

*The required sample size of the platform study will be driven by the design and the objectives of the specific ISAs and the predicted performance of the planned analyses on the endpoints of interest, including effect assumptions for the ISAs.*

*Details on the sample size calculation will be provided in the respective intervention cohorts.*

If subpopulations of special interest exist / Enrichment

*The following stratification factors will be considered in the randomization:*

*To allow for testing of subgroup effects in stratum* [X], *at least* [X]% *of the enrolled participants per ISA shall be enrolled from stratum* [X]. *Any enrichment for a specific intervention will be described in this section of the ISA*.

Corrections for evaluable patients / re-randomizations

*Approximately* [X] *participants will be randomly assigned to each ISA such that approximately* [X] *evaluable participants will complete the study*.

*Study participants may be eligible of sequentially randomization into other open ISAs, upon completion of previous ISAs. Assuming that* [X*]% of participants will be re-randomized to multiple ISAs, the total number of required participants for the platform is* [X].

<*End of suggested text*>

# Supporting Documentation

The subsections within this section are recommended to be named Appendix XXX in line with the CPT.

The appendix/appendices can include important information not included in the body text of the SAP, for example:

- Participant disposition
- Baseline characteristics and demographics
- Protocol deviations
- Medical history
- Prior/concomitant/follow-up medications (including dictionary)
- Data derivation rules
- Adverse events of special interest
- Potentially clinically significant criteria for safety endpoints
- Simulation report

## Appendix 1: List of Abbreviations

- Generate a list or table while drafting the SAP to reflect the abbreviations and acronyms used in the SAP.
- Only include those that are used more than once in the document. Once a term is abbreviated or an acronym is introduced, the abbreviation or acronym should be used in the rest of the document.
- Abbreviations and acronyms are defined where first used in the document or per sponsor company policy.
- Delete this appendix if not required.

<Start of example text>

| **Abbreviation** | **Definition** |
| --- | --- |
| AE | adverse event |
| ANCOVA | analysis of covariance |
| BMI | body mass index |
| CI | confidence interval |
| DMC | data monitoring committee |
| EMA | European Medicines Agency |
| FDA | US Food and Drug Administration |
| ICH | International Council on Harmonization |
| IDMC | Independent Data Monitoring Committee |
| ISA | Intervention-specific Appendix |
| ISA-SAP | Intervention-specific Appendix Statistical Analysis Plan |
| LLoQ | lower limit of quantification |
| mSAP | Master SAP |
| MedDRA | medical dictionary for regulatory activities |
| SAP | Statistical Analysis Plan |
| SMQs | standardized MedDRA queries |

<End of example text>

# References

- See therapeutic libraries for key references to include.
- References to both internal and external documents and publications should be listed in alphabetical order. Do not reference internal reports in preparation.
- In the reference list, use the style and format published by the International Committee of Medical Journal Editors (ICMJE 2019). Citations to external documents and publications should be indicated in the text by citing the author and year within parentheses. For example, the in-text citation for the reference included would be (Hatcher et al, 2007).

<Start of example text>

Hatcher RA, Trussell J, Nelson AL, Cates W Jr, Stewart F, Kowal D, eds. Contraceptive technology. 19th edition. New York: Ardent Media, 2007(a): 24. Table 3-2.

<End of example text>
