# Supplementary material for: Developing generic templates to shape the future for conducting integrated research platform trials
Source: Trials. 2024 Mar 21;25:204. doi: 10.1186/s13063-024-08034-8 (PMC10956223; doi:10.1186/s13063-024-08034-8)
Supplement: Supplementary file 4 — Additional file 4. EU-PEARL Guidance to CTR Cover letter. [file 13063_2024_8034_MOESM4_ESM.docx]

|  |  |
| --- | --- |

**Document title:**

**Guidance* for supplementary information to the CTR Cover Letter**

***The guidance applies to the companion protocol templates named Generic Master Protocol Template and Intervention Specific Appendix Template documents prepared in the EU-PEARL consortium**

**Document history and plan**

This updated document (V4 25April2023) is based on the **EU-PEARL** **D2.3** ‘Provisional Generic Master Protocol Template and Appendix for IRPs’ (V2 30 April 2021). This version is the version after the review by the CTCG.

D2.3 was based on TransCelerate Common Protocol Template V8, copyright TransCelerate Biopharma Inc. 2019 – 2021.

Input received from the review committee was assessed for incorporation in the final deliverable D2.6 which is planned to be made publicly available at the end of the project (April 2023).

| **Authors** | Sal Morello, Siard Houtzager, Cecile Spiertz (24 - Janssen), Sabina Hernandez Penna, Peter Mesenbrink (25 - Novartis), Tom Parke (14 - Berry), Franz Koenig (4 - MUW), Benjamin Hofner, Katharina Hees (19 - PEI), |
| --- | --- |
| **Contact person EU-PEARL** | ECRIN; Peter Mesenbrink (Novartis) |
| **Document version** | Version 4 |
| **Date** | 25April2023 |

The EU-PEARL project has received funding from the Innovative Medicines Initiative 2 Joint Undertaking (JU) under grant agreement No 853966. The JU receives support from the European Union’s Horizon 2020 research and innovation programme and EFPIA and CHILDREN'S TUMOR FOUNDATION, GLOBAL ALLIANCE FOR TB DRUG DEVELOPMENT NON PROFIT ORGANISATION, SPRINGWORKS THERAPEUTICS INC.

**Disclaimer**

These materials are provided AS IS WITHOUT WARRANTY OF ANY KIND, EITHER EXPRESSED OR IMPLIED, INCLUDING, BUT NOT LIMITED TO, THE IMPLIED WARRANTIES OF MERCHANTABILITY, FITNESS FOR A PARTICULAR PURPOSE, OR NONINFRINGEMENT.

EU-PEARL and its members do not accept any responsibility for any loss of any kind including loss of revenue, business, anticipated savings or profits, loss of goodwill or data, or for any indirect consequential loss whatsoever to any person using these materials or acting or refraining from action as a result of the information contained in these materials. Any party using these materials bears sole and complete responsibility for ensuring that the materials, whether modified or not, are suitable for the particular use and are accurate, current, commercially reasonable under the circumstances, and comply with all applicable laws and regulations.

Nothing in this template should be construed to represent or warrant that persons using this template have complied with all applicable laws and regulations. All individuals and organizations using this template bear responsibility for complying with the applicable laws and regulations for the relevant jurisdiction

**Abbreviation list**

| **Acronym / Abbreviation** | **Meaning** |
| --- | --- |
| **CTA** | Clinical Trial Applications |
| **CTCG** | Clinical Trial Coordination Group |
| **CTR** | Clinical Trial Regulation |
| **CT#** | Clinical Trial Number |
| **D** | Deliverable |
| **IMP** | Investigational Medicinal Products |
| **IRPs** | Integrated Research Platform |
| **ISA** | Intervention Specific Appendix |
| **MP** | Master Protocol |
| **V** | Version |

1. **Purpose and use of this document**

In a platform trial, sub-protocols (or Intervention Specific Appendices - ISAs) can be added or closed throughout the lifetime of the trial, resulting in a complex trial design. It is often unknown at the beginning (i.e., planning stage) how many sub-protocols/ISAs will eventually be included in a platform trial. As such, it is important (and may be required by national regulation) to inform a regulatory agency when key changes occur (e.g. protocol amendments) including the impact of the change to the ongoing platform trial and sub-protocols/ISAs.

The purpose of this guidance document is to aid Applicant’s in providing clear communication to the regulatory agencies through a cover letter. It will provide the reviewer with background on the scope of the trial and the current submission and address how the different sub-protocols/ ISAs will interact with a Master Protocol, which IMPs (Investigational Medicinal Products) are studied and other relevant information for the reviewer.

In addition, this guidance document provides two examples of an overview table to be included in a cover letter for a clinical trial application submitted under the Clinical Trial Regulation (CTR) EU No536/2014. The recommendations in this document supplement the cover letter requirements as laid out in Annex I B (and Annex II B) of the regulation. More specifically, it is stated herein that the cover letter “shall draw attention to any features which are particular to the clinical trial.” The EU-PEARL working group is recommending using the guidance below to organize and summarize the key features of a complex clinical trial design both at the time of study initiation and substantial amendment requests.

Please note that this document is intended as guidance for the user and cannot cover every possible situation or scenario.

2. **Proposed additional information to be included in the CTR cover letter, when submitting a complex clinical trial**

Sponsors should follow the CTR cover letter description as per the Clinical Trials Regulation (Regulation (EU) No 536/2014, Annex I B, Annex II b as well as CTR Article 81.9 for non-substantial modifications). The following points are additional considerations to facilitate the review of complex clinical trials.

2.1 Complex Innovative Design

It is recommended to clearly state that the application concerns a *complex clinical trial* with the description of the main characteristics of the design, and a short rationale of the strategic decisions made.

Clearly indicate the key features of the Master Protocol complex clinical trial design both at the time of initial clinical trial applications (CTAs) and substantial modification requests and include the relationship between the “*Master Protocol*” and the different sub-protocols/ ISAs. Highlight the protocol elements which are ‘in common’ in the Master protocol (for example: the overall study design, general study population, main inclusion and exclusion criteria, common elements of the randomization scheme, informed consent process, primary and secondary outcomes, statistical methodology and the planned analyses that are common across all sub-protocols/ISAs).

In addition, include the intervention specific features in the sub-protocol/ISA; for example, introduction of a new IMP or population, as well as the use of companion diagnostics, if applicable.

Together, a master protocol and a sub-protocol/ISA define all the elements needed to conduct a study. It is expected that the Master Protocol and sub-protocol/ISA are reviewed in parallel. A clear guidance is intended to facilitate reviewers to extract the relevant information for a set of sub-protocols/ISAs. It will help a reviewer to focus on and assess the relevant pages in the whole protocol, which are relevant for the approval of a new sub-protocol/ISA.

2.2 Sub-protocol/ISA study design

To aid the reviewer it may be helpful to provide additional sub-protocol/ISA specific details and background on the sub-protocol/ISA study design. For example: primary objective of the sub-protocol/ISA, specific interventions, a rationale for the test medication, additional entry criteria, concomitant medication use and safety assessments, a schedule of events and study duration.

2.3 Modular structure

To provide an overview of the relationship of the modular structure of the platform study, we propose that the cover letter could include a table or a figure to indicate the connections between the Master Protocol and the individual sub-protocols/ISAs as described in the appendix of report of the EU-PEARL Deliverable [D2.6 “Final Generic Master Protocol Template and Appendix for IRPs”](https://eu-pearl.eu/deliverables-patient-centric-clinical-trial-platforms/)

.

**Examples for supportive tables highlighting the conduct of trial / regulatory submission history**

The examples provided below for inclusion in the cover letter are derived from the CTFG [recommendation paper on the initiation and conduct of complex clinical trials](https://www.hma.eu/fileadmin/dateien/Human_Medicines/01-About_HMA/Working_Groups/CTFG/2019_02_CTFG_Recommendation_paper_on_Complex_Clinical_Trials.pdf), dated 12 February 2019.

.

1) One trial with sub-protocols/ISAs added as substantial amendments

*Table 1: Platform trial overview for inclusion in the cover letter*

| Submission type | Key documents | Countries^a^ | Reason for submission |
| --- | --- | --- | --- |
| Initial CTA | Master Protocol (MP) Original  Approved <insert date> | [All] | Submission of Master Protocol and Sub-protocols 1, 2, and 3 under EuCT 2018-XXXXXX-XX |
| CTA-A | MP Amendment 1  Approved <insert date> | [All] | Substantial Amendment |
| CTA^b^ | MP Amendment 2  Approved <insert date> | [All] | Submission of Sub-protocol 4 |
| CTA^b^ | MP Amendment 3  For review | [All] | Closing of Sub-protocol 2 |
| … | … | … | … |

^a^ Optional: countries where the trials are planned to be run. Paying attention to specific geographic differences.

^b^ Addition of a sub-protocol/ISA to an ongoing clinical trial may not require an amendment of the MP. The MP and sub-protocol/ISA are written and amended as separate, companion documents^.^

2) Separate trials with a similar Master Protocol – submitted as individual ‘stand-alone trials’ (with multiple EUCT numbers).

*Table 2: Platform trial overview for inclusion in cover letter*

| **Submission type** | **Clinical Trial number ^a^** | **Key documents** | | | | **Countries^b^** | **Reason for submission** |  |
| --- | --- | --- | --- | --- | --- | --- | --- | --- |
|  |  |  |  |  |  |  |  |  |
| Initial CTA | 2020-XXXXXX-XX  Approved <insert date> | Master Protocol (MP) Original Sub-protocol/ISA 1 Original | | | | [All] | Submission of Master Protocol and Drug A |  |
| CTA^c^ | 2021-XXXXXX-XX  Approved <insert date> | MP with Sub-protocol/ISA 2 | | | | [All] | Master Protocol and introduction of Drug B and D |  |
|  |  |  |  |  |  |  |  |  |
| CTA^c^ | 2022-XXXXXX-XX  Approved <insert date> | MP with Sub-protocol/ISA 3 | | | | [All] | Master Protocol and introdcution of Drug E |  |
|  |  |  |  |  |  |  |  |  |
| … |  |  | | | |  | … |  |
| End of Trial | 2020-XXXXXX-XX  Approved <insert date> | … | | | | … | Closing of Trial Notification for Drug A |  |
|  |  |  |  |  |  | | |  |

^a^ CT # = Clinical trial number (previously referred to as EudraCT #)

^b^ Optional: countries where the trials are planned to be run. Paying attention to specific geographic differences.

^c^ Addition of a sub-protocol/ISA as a ‘stand-alone trial’ with a new CT number
